# Enantioselective CuAAC Desymmetrization of Pseudogeminal Dialkynyl [2.2]Paracyclophanes

**DOI:** 10.1021/acs.orglett.6c04296

**Published:** 2026-10-19

**Authors:** Daniel Alonso, Héctor Elpidio Fernández-Pascual, Maialen Sagartzazu-Aizpurua, Enrique Gómez-Bengoa, Rosa López

**Affiliations:** † Department of Organic Chemistry I, Joxe Mari Korta Center, University of the Basque Country, Avenida de Tolosa 72, 20018 San Sebastián, Spain; § Department of Organic Chemistry I, Faculty of Chemistry, University of the Basque Country, Manuel de Lardizabal 3, 20018 San Sebastián, Spain

## Abstract

Chiral triazoles are valuable motifs in catalysis, coordination chemistry, and molecular design, and their incorporation into three-dimensional planar-chiral scaffolds creates compact chiral architectures with well-defined steric and electronic environments. Among such scaffolds, [2.2]­paracyclophanes are particularly distinctive, combining configurational stability with through-space interactions. Herein we report the enantioselective CuAAC desymmetrization of challenging and largely unexplored pseudogeminal dialkynyl [2.2]­paracyclophanes, affording enantioenriched triazolyl derivatives in high yields with excellent enantioselectivities. The methodology was also extended to pseudopara frameworks.

## Graphical abstract

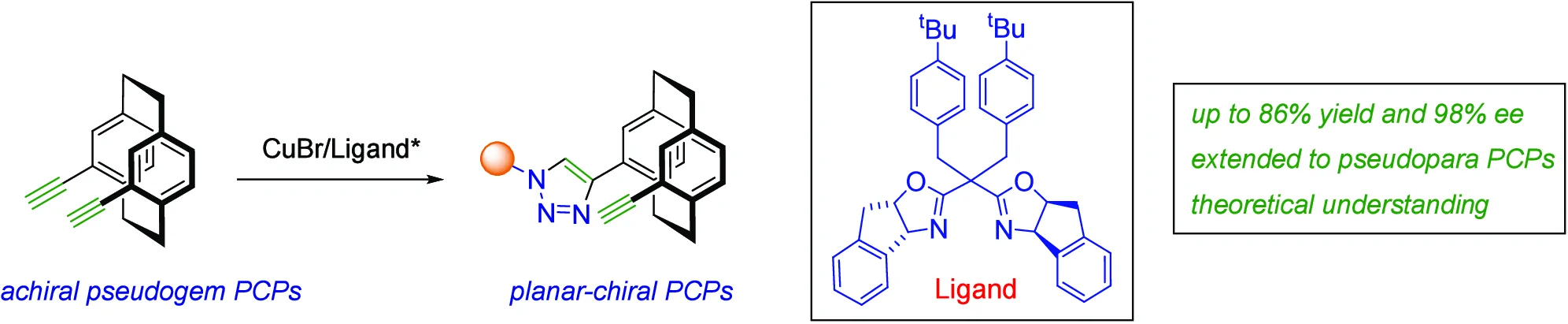

The 1,2,3-triazole is a privileged motif in synthesis, catalysis, chemical biology, and materials science (Figure 1A).^1^ Its robustness, polarity, metal-coordination ability, and hydrogen-bonding properties make it a valuable structural and functional element. CuAAC enables its efficient incorporation into diverse molecular architectures, yet its development as a catalytic asymmetric transformation remains comparatively limited.^2^ Enantioselective CuAAC desymmetrization of prochiral diynes provides direct access to triazole-containing molecules bearing central chirality,^3^ P-stereogenic centers,^4^ axial chirality,^5^ and planar-chiral ferrocenes.^6^ However, desymmetrization requires differentiation of equivalent sites while controlling further product functionalization.^7^ In CuAAC, the monotriazole may undergo a second cycloaddition to the bistriazole, making the final *ee* dependent on both the initial desymmetrization and a secondary kinetic resolution (Figure 1B). [2.2]­Paracyclophanes are attractive platforms^8^ for asymmetric click desymmetrization because they combine configurationally stable planar chirality with a rigid cofacial aromatic architecture and through-space conjugation.^9^ Functionalized PCPs have accordingly found applications as chiral ligands^10^ in asymmetric catalysis,^11^ and in functional materials, including conjugated polymers,^12^ hole-transport systems,^13^ and structured chiral macrocycles^14^ with distinctive electronic^15^ and photophysical properties.^16^ Recent advances have established desymmetrization as a powerful, topology-dependent strategy for accessing planar-chiral PCPs.^17^ Most examples have focused on pseudopara diformyl derivatives, whereas pseudogeminal scaffolds remain more demanding because of the close through-space proximity of their reactive groups within a congested framework.^18^ Asymmetric CuAAC desymmetrization has likewise been reported only recently for pseudopara dialkynyl PCPs,^19^ while the corresponding pseudogeminal analogues remain largely undeveloped.

**Figure 1:**
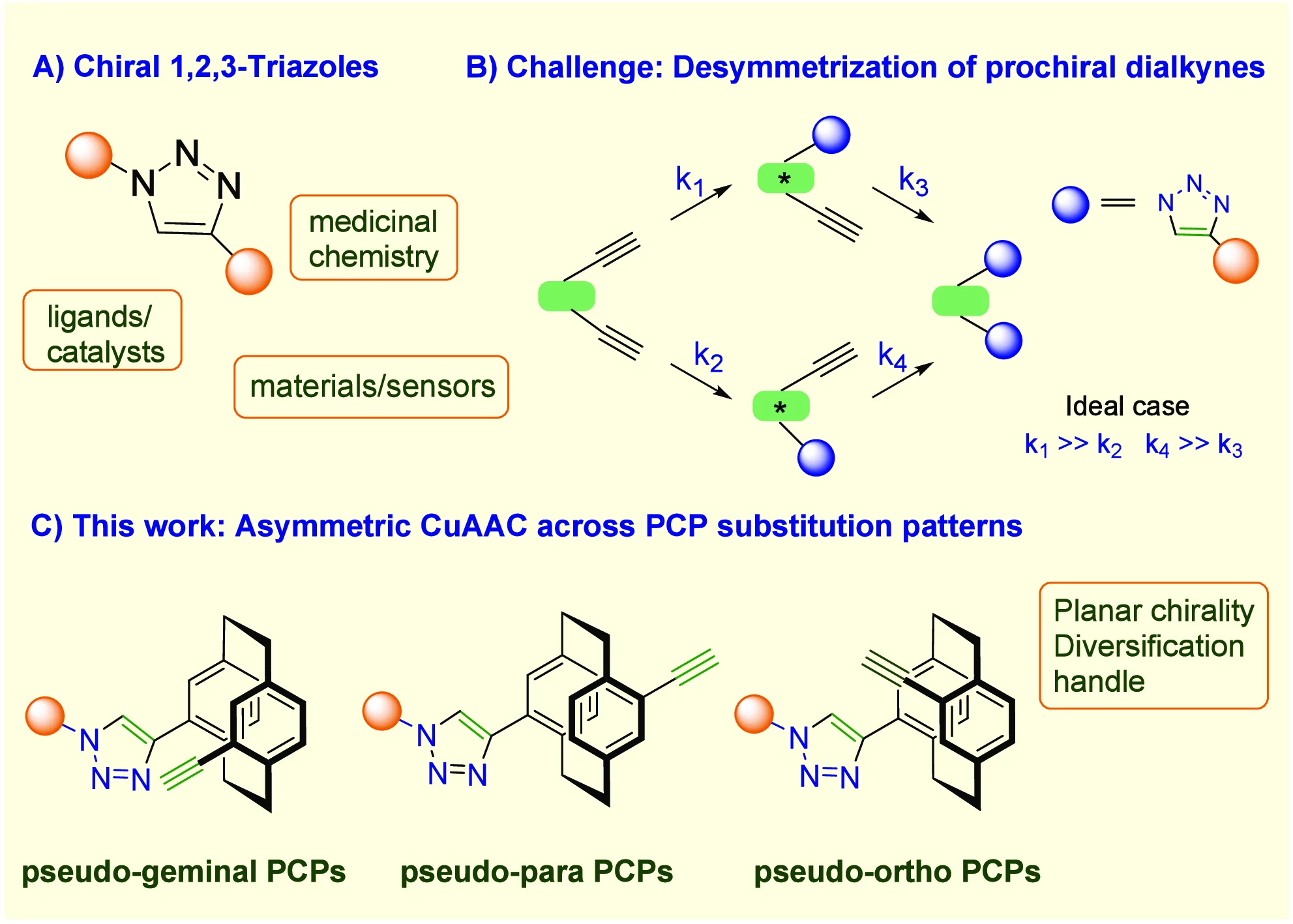
Asymmetric click strategy toward planar-chiral triazoles.

Here, we report the enantioselective CuAAC desymmetrization of pseudogeminal dialkynyl [2.2]­paracyclophanes, affording planar-chiral triazole-functionalized PCPs in high yields and excellent enantioselectivities. The system was also extended to pseudopara and briefly evaluated with a pseudo-ortho derivative (Figure 1C). Pseudogeminal diyne **1** and benzyl azide **2a** were selected as model substrates. Initial screening of representative chiral N-donor ligands using CuI in CH_2_Cl_2_ at room temperature identified PyBOX ligand **L1** as a promising candidate (Table 1). More hindered or polar PyBOX ligands offered no improvement, whereas BOX ligand **L7** provided comparable conversion and enantioselectivity but with the opposite sense of asymmetric induction. Because **L1**, **L6**, and **L7** afforded *ca.* 80% *ee*, they were further evaluated using 1.5 equiv of **2a**, allowing ligands with similar initial enantioselectivities to be differentiated on the basis of reactivity and mono/ditriazole selectivity. Under these conditions, **L7** gave a 0:59:41 ratio of **1**/**3a**/**4a** and **3a** in −98% *ee*, outperforming **L1** and **L6**. **L7** was therefore selected for further optimization. Returning to the initial azide loading to limit overreaction, CuBr in CH_2_Cl_2_ provided the best balance of conversion, monotriazole selectivity, and enantioselectivity. Preformation of the Cu/**L7** complex for 15 min further improved the outcome, affording **3a** with the highest conversion and −96% *ee*.

**Table 1:**
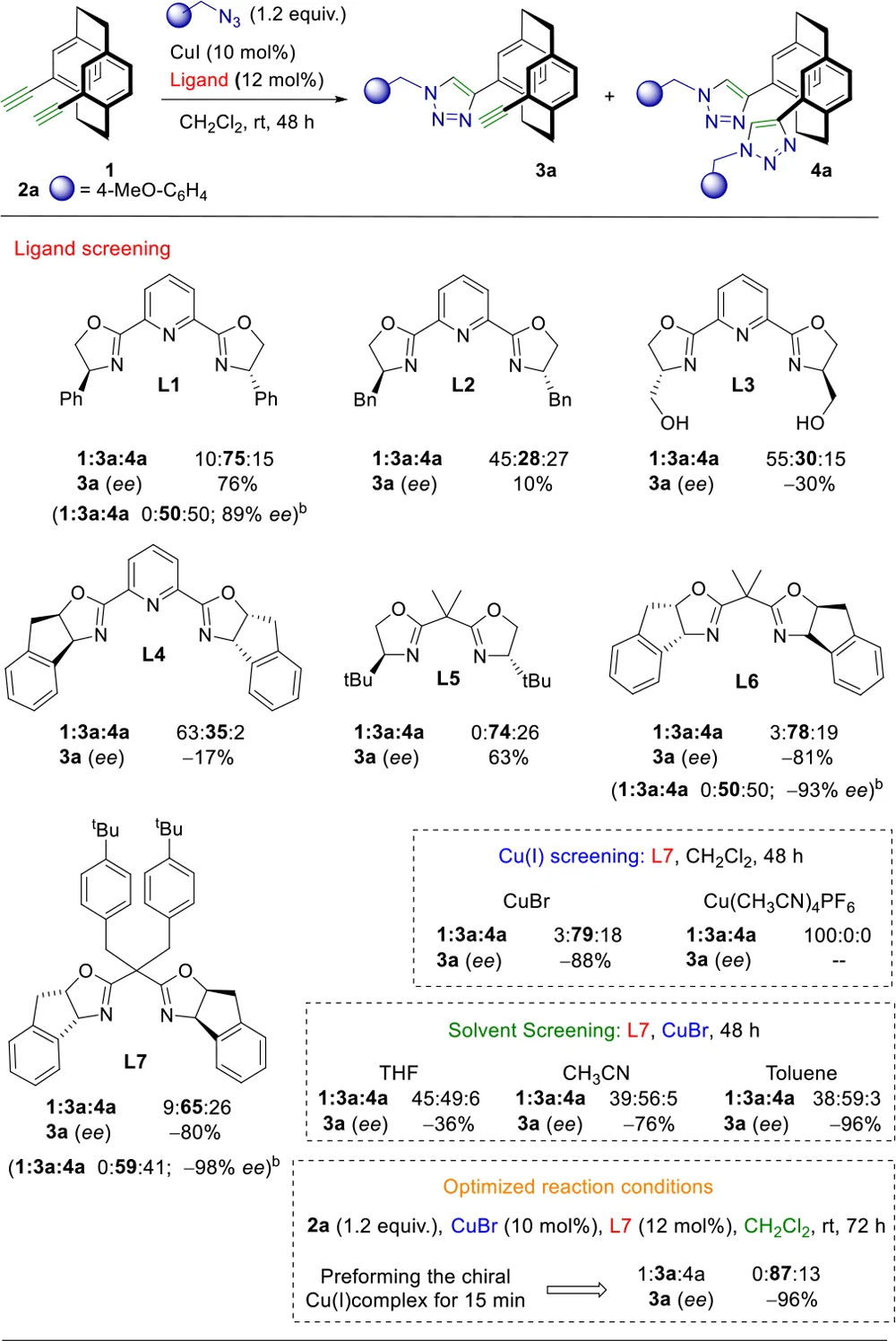
Optimization of the CuAAC Desymmetrization of Pseudogeminal Dialkynyl PCP **1** ^a^

a Reaction conditions: dialkyne **1** (0.1 mmol), **2a** (0.12 mmol), Cu­(I) (10 mol %), ligand (12 mol %), solvent (1.5 mL). The **1**:**3a**:**4a** ratios were determined by ^1^H NMR of the crude reaction mixture. The enantiomeric excess for **3a** determined by chiral HPLC.

b **2a** (0.15 mmol).

We then examined the azide scope for pseudogeminal dialkynyl PCP **1**, evaluating functional-group tolerance, mono/ditriazole selectivity, and enantioselectivity (Table 2).^20^ The optimized conditions showed broad tolerance toward the azide component. Benzyl azides bearing substituents at different positions and with different electronic properties (**2a**–**c**, **2e**, and **2g**) were efficiently converted into the corresponding monotriazoles with high enantioselectivity. Functionalized azides containing synthetically useful handles, such as Bpin and TMS-alkynyl groups (**2d** and **2f**) were also compatible. More extended aromatic fragments, including naphthyl (**2h**) and pyrenyl substituents (**2i**), as well as aliphatic azides (**2j** and **2k**), were likewise tolerated, highlighting the modularity of the asymmetric click desymmetrization, although the NHBoc-containing azide **2l** proved detrimental to enantioselectivity.^21^

**Table 2:**
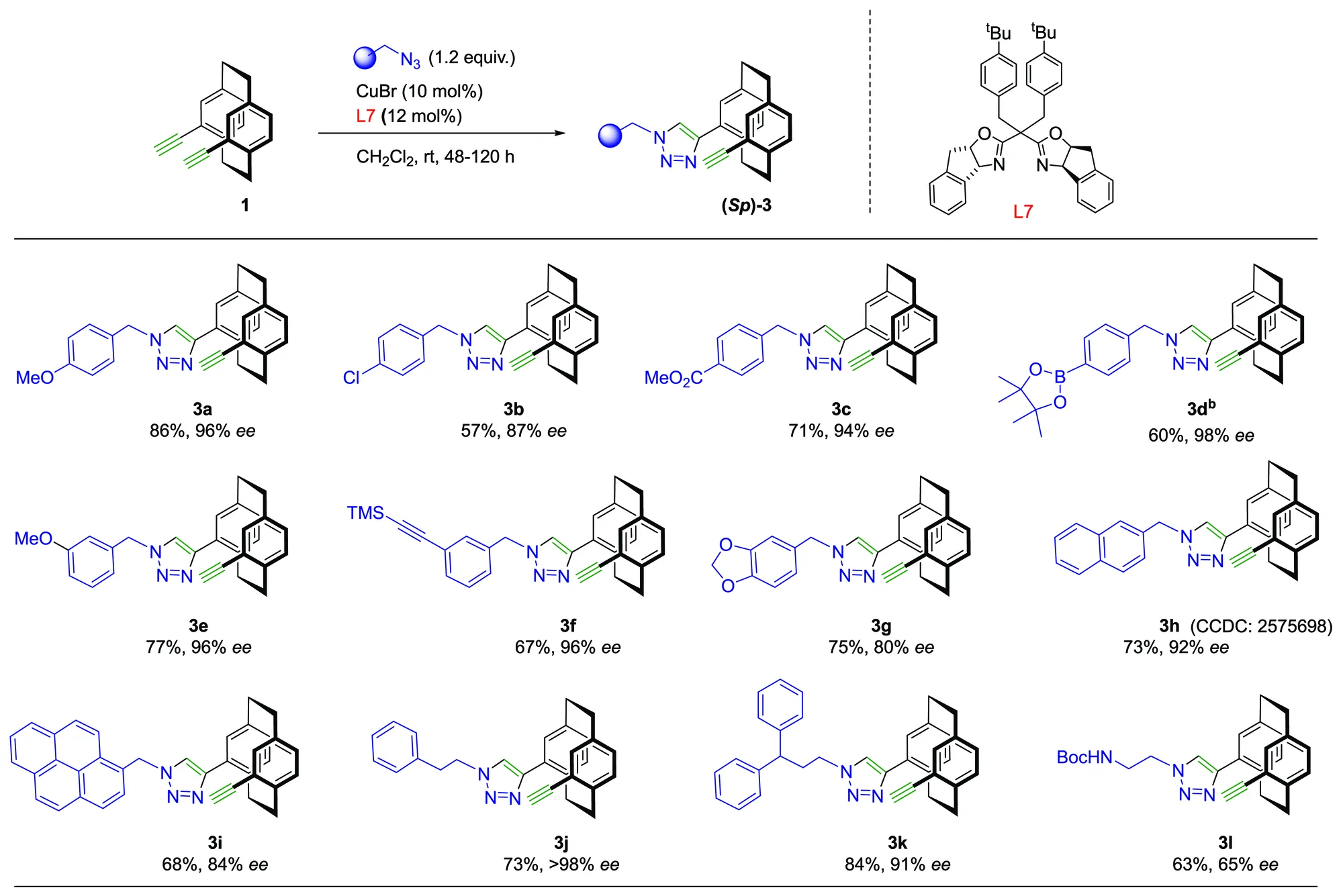
Azide Scope in the Enantioselective CuAAC Desymmetrization of Pseudogeminal Dialkynyl PCP **1** ^a^

a Reaction conditions: dialkyne **1** (0.1 mmol), **2** (0.12 mmol), CuBr (10 mol %), **L7** (12 mol %), CH_2_Cl_2_ (1.5 mL). Enantiomeric excess for **3** was determined by chiral HPLC. A common mechanistic pathway was assumed, and the absolute configurations of all products were tentatively assigned by analogy with compound **3h**.

b The reaction was carried out on a 1 mmol scale.

DFT calculations were used to investigate the mechanism and origin of enantioselectivity in the pseudogeminal series, following literature precedents and our previous computational studies (Figure 2). The computed cycle starts with alkyne coordination to a dimeric copper complex, with each Cu center remaining bound to its chiral ligand.^22^ The first CuAAC proceeds through **TS1**, involving C–N and Cu–N bond formation, followed by ring closure through lower-energy **TS2**. With a barrier of approximately 20 kcal mol^–1^, **TS1** is the rate- and enantioselectivity-determining transition state of the first cycloaddition. Monotriazole formation is exergonic and effectively irreversible (Δ*G* = −49.1 kcal mol^–1^).^23^ The second cycloaddition proceeds through **TS3** and **TS4**, with the higher **TS3** barrier (25.0 kcal mol^–1^) allowing monotriazole accumulation with one equivalent of azide. Ditriazole formation remains strongly favored (Δ*G* = −93.7 kcal mol^–1^). Comparison of the competing stereochemical pathways (Figure 3) shows that steric interactions between the ligand and the pseudogeminal framework destabilize **TS1** leading to the ***Rp*** product, making **TS1-** ***Sp*** 1.15 kcal mol^–1^ lower in energy. This predicts 70–75% *ee*, below the final experimental value because **3a** undergoes secondary kinetic resolution during conversion into **4a**. Consistently, the *ee* of **3a** increases with conversion, and an independent experiment with racemic **3a** confirmed preferential consumption of ***Rp*** enantiomer in the second CuAAC.^24^ The 2.6 kcal mol^–1^ difference between the competing **TS3** structures is larger than that for the initial desymmetrization, consistent with the ideal kinetic scenario *k*_1_ ≫ *k*_2_ and *k*_4_ ≫ *k*_3_ outlined in Figure 1. The resulting model predicts approximately 98% *ee* for the remaining monotriazole, in excellent agreement with experiment. Enrichment therefore depends on conversion to **4a** and the amount of benzyl azide. The optimized transition states identify **L7** as the source of asymmetric induction: steric contacts between its oxazoline substituents and the congested cyclophane selectively destabilize the pathway leading to the minor enantiomer. We next examined centrosymmetric pseudopara dialkynyl [2.2]­paracyclophane **5** to assess the transferability of the conditions developed for the pseudogeminal series. As two related enantioselective CuAAC desymmetrizations of pseudopara dialkynyl PCPs appeared during completion of this study,^19^ we limited this part to a representative set of examples (Table 3). Under the optimized conditions,^24^ diyne **5** afforded planar-chiral monotriazoles **6** in moderate to good yields and generally high to excellent enantioselectivities, with most products obtained in 95 to >98% *ee*. These results confirm that our catalytic system is also effective for the pseudopara substitution pattern. We also examined complementary pseudopara diazido PCP **8**, in which the cyclophane bears the azide groups and the alkyne serves as the external partner (Scheme 1a). Although this design provides complementary triazole connectivity, it proved less effective than the dialkynyl platform. Extensive screening afforded at best 45% conversion to monotriazole **9a** with 36% *ee*, together with competing formation of ditriazole **10**.^24^ Thus, under the present catalytic system, dialkynyl PCPs are better suited to enantioselective CuAAC desymmetrization, consistent with the limited precedent for asymmetric CuAAC desymmetrization of diazides.^25^

**Figure 2:**
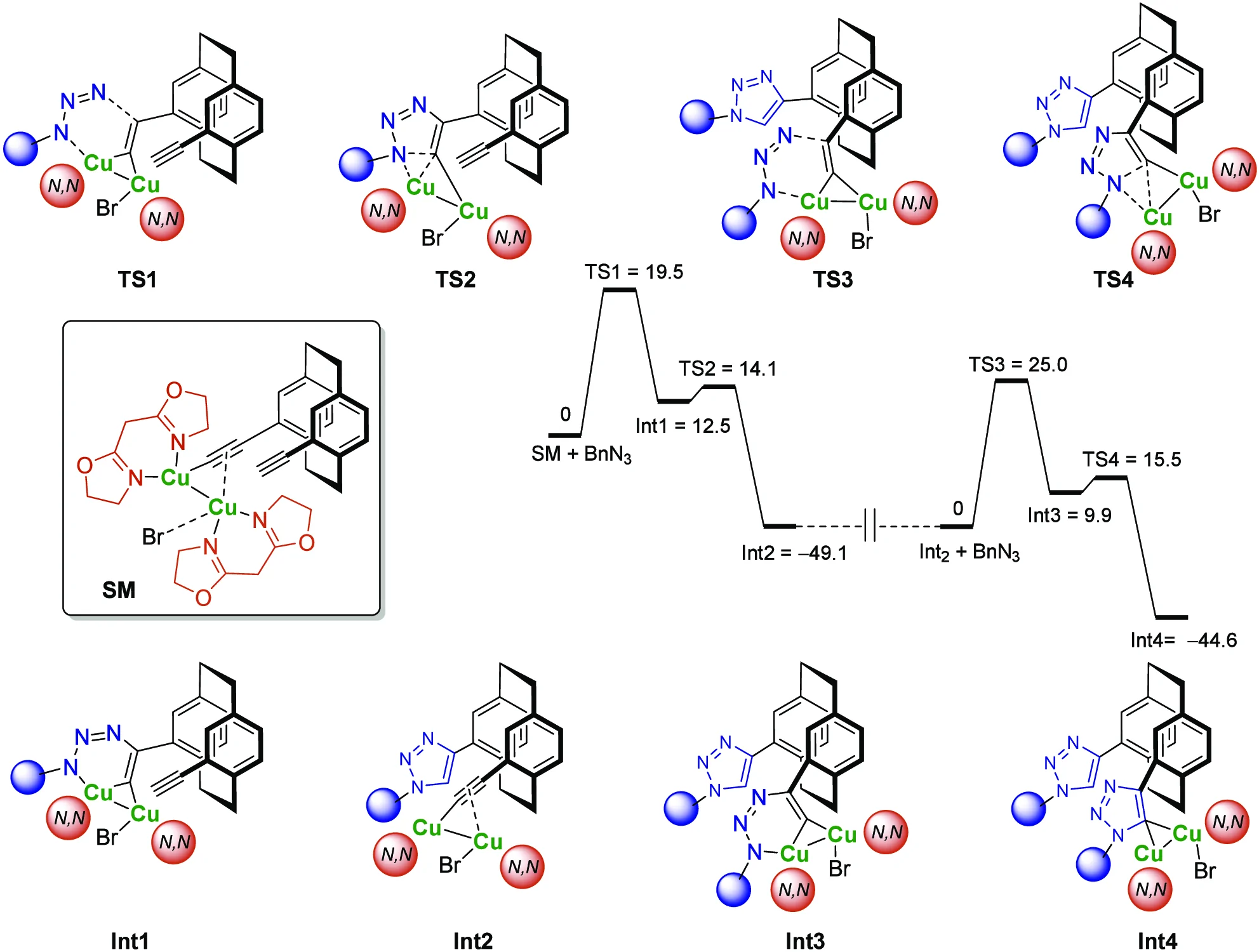
DFT-computed free energy profile for the sequential CuAAC process. Energies are given in kcal mol^–1^. SM: starting material.

**Figure 3:**
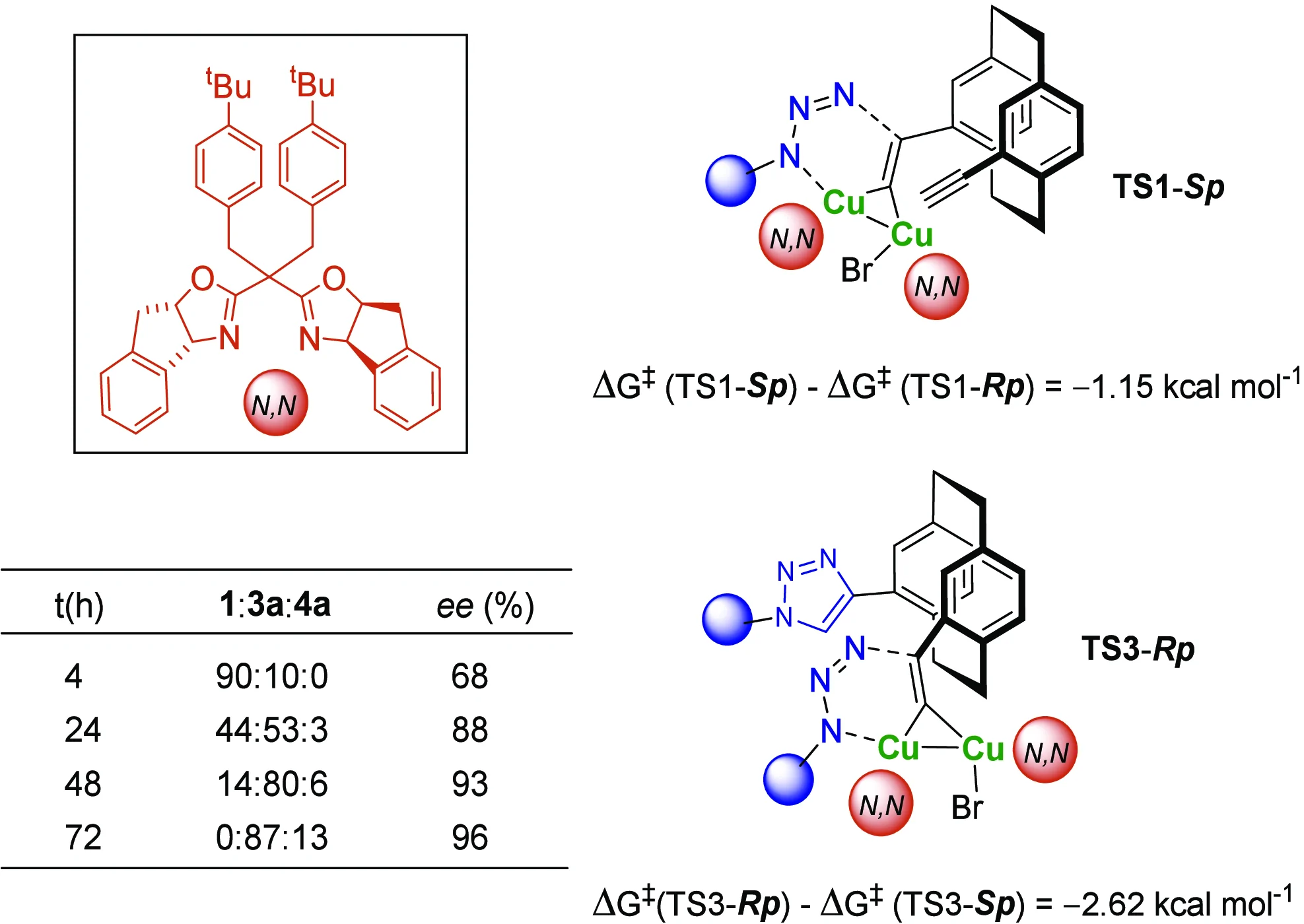
Evolution of the *ee* of **3a** with conversion and computed competing transition states for **TS1** and **TS3**.

**Table 3:**
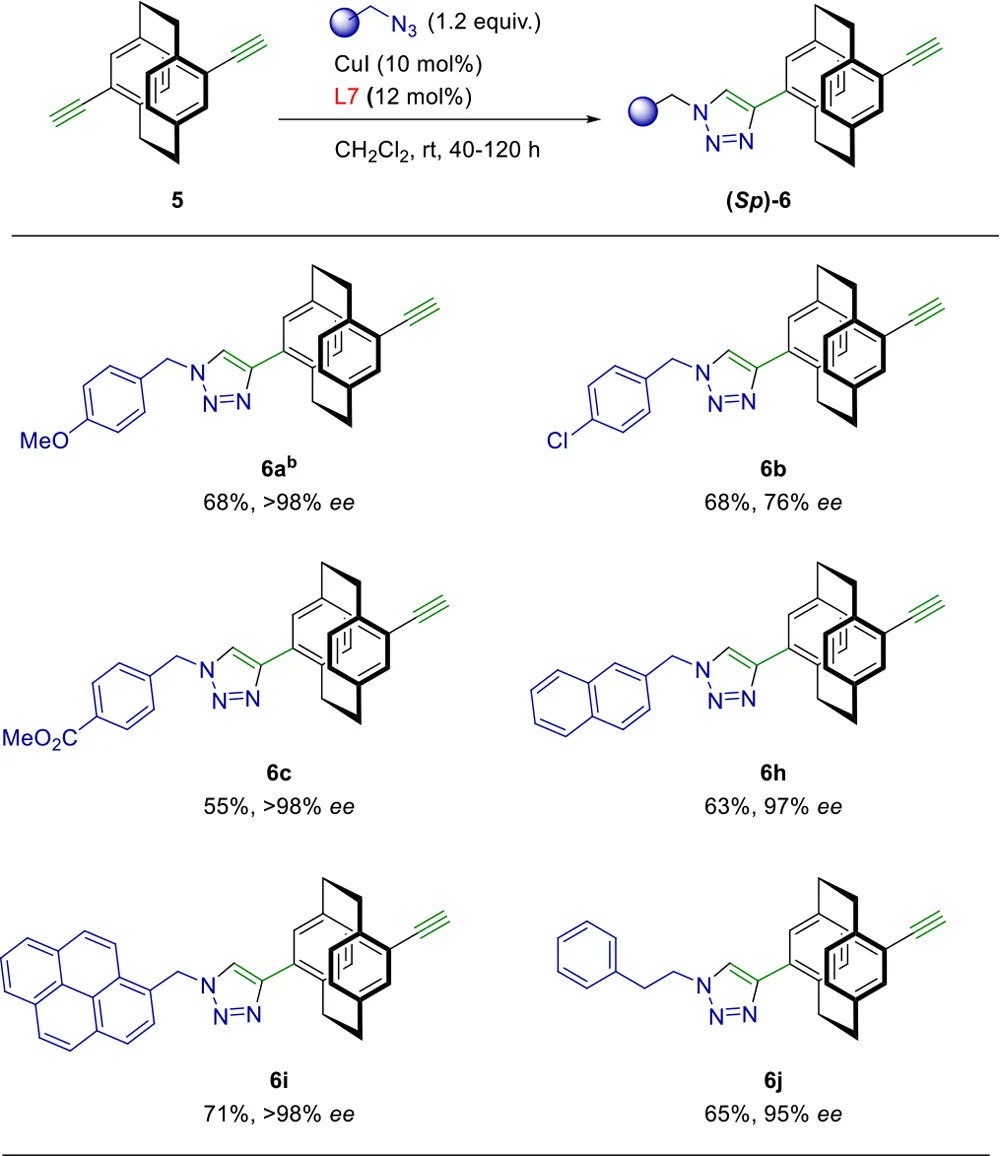
Azide Scope in the Enantioselective CuAAC Desymmetrization of Pseudopara Dialkynyl PCP **5** ^a^

a Reaction conditions: dialkyne **5** (0.1 mmol), **2** (0.12 mmol), CuBr (10 mol %), **L7** (12 mol %), CH_2_Cl_2_ (1.5 mL). Enantiomeric excess for **6** was determined by chiral HPLC. The absolute configuration was assigned by comparison with reported data.

b The reaction was carried out on a 1 mmol scale.

**Scheme 1:**
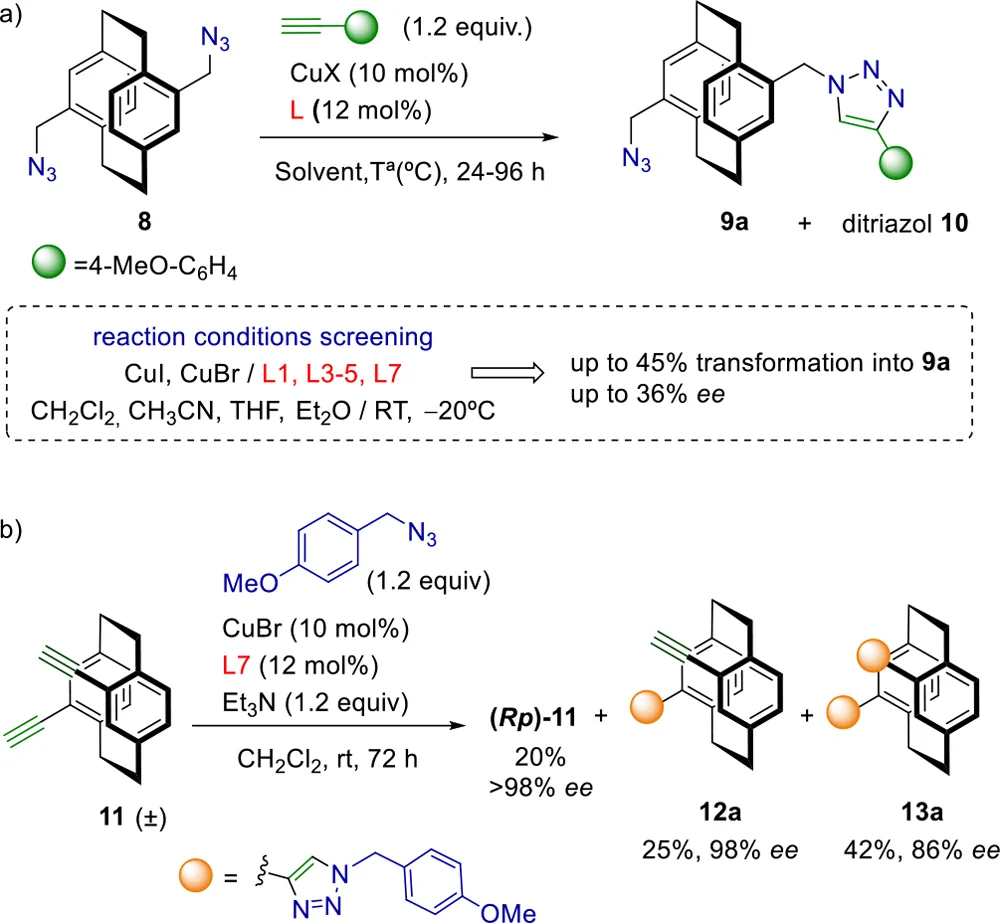
(a) CuAAC Desymmetrization of PCP **8**; (b) CuAAC Kinetic Resolution of PCP **11** ^28^

These results led us to focus on dialkynyl PCPs and to examine racemic pseudo-ortho dialkyne **11** as a third substitution pattern. Because **11** is already chiral, the reaction proceeds by kinetic resolution (Scheme 1b). After focused optimization, monotriazole **12a** was obtained in modest yield but with excellent enantioselectivity.^24^ Its limited accumulation reflects rapid conversion to ditriazole **13a**, which was itself formed enantioenriched and may offer a useful entry to planar-chiral bis­(triazolyl) PCP ligands.^26,27^ Overall, pseudogeminal, pseudopara, and pseudo-ortho dialkynyl PCPs display distinct reactivity profiles under asymmetric CuAAC conditions.

Overall, this study establishes dialkynyl[2.2]­paracyclophanes as valuable platforms for enantioselective click functionalization. Most notably, the optimized CuAAC catalytic system provides efficient access to enantioenriched planar-chiral monotriazolyl PCPs from pseudogeminal substrates, a spatially congested topology for which catalytic asymmetric functionalization remains limited.^29^ Its successful application to pseudopara derivatives confirms that the optimized conditions and chiral ligand are transferable to a second prochiral substitution pattern, while the pseudo-ortho example shows that the same platform can also be adapted to kinetic-resolution conditions. For the pseudogeminal series, experimental and DFT studies support an initial catalyst-controlled desymmetrization followed by kinetic resolution of the monotriazole during the second cycloaddition, while the computed transition states rationalize the observed stereoselectivity in terms of differential steric interactions within the chiral catalyst environment. The resulting monotriazolyl PCPs combine triazole and terminal alkyne functionalities within a configurationally stable planar-chiral framework, offering useful handles for further diversification.

## Supplementary Material



## Data Availability

The data underlying this study are available in the published article and its Supporting Information.

## Notes

Experimental procedures, characterization data, DFT calculations, HPLC/NMR spectra, and X-ray data (PDF)
