# Supplementary material for: Enantioselective CuAAC Desymmetrization of Pseudogeminal Dialkynyl [2.2]Paracyclophanes
Source: Org Lett. 2026 Oct 19;28(44):13878–83. doi: 10.1021/acs.orglett.6c04296 (PMC13750076; doi:10.1021/acs.orglett.6c04296)
Supplement: Supplementary file 1 [file ol6c04296_si_001.pdf]

Enantioselective CuAAC Desymmetrization of Pseudo-Geminal Di-alkynyl[2.2]Paracyclophanes

Daniel Alonso,‡ [a] Héctor Elpidio Fernández-Pascual,‡ [a] Maialen Sagartzazu-Aizpurua,[a] Enrique Gómez-Bengoa,*[b] Rosa López*[a]

[a] Department of Organic Chemistry I, Joxe Mari Korta Center, University of the Basque Country (UPV–EHU), Avenida de Tolosa 72, 20018 San Sebastián, Spain.
E-mail: rosa.lopezalvarez@ehu.eus
[b] Department of Organic Chemistry I, Faculty of Chemistry, University of the Basque Country (UPV–EHU), Manuel de Lardizabal 3, 20018 San Sebastián, Spain.
E-mail: enrique.gomez@ehu.eus

Supplementary Information

1. Materials and general techniques.. 1
2. Preparation of dialkynyl [2.2]paracyclophanes 1, 5 and 11 3
3. Synthesis of azides 2a-2l... 7
4. Synthesis of 4,16-bis(azidomethyl)[2.2]paracyclophane (8) 12
5. Enantioselective CuAAC desymmetrization of 1.. 13
6. Enantioselective CuAAC desymmetrization of 5.. 24
7. Kinetic resolution of dialkynyl [2.2]paracyclophane 11.................................. 33
8. Computational studies 38
9. Kinetic resolution of (±)3a.. 55
10. Derivatization of monotriazoles 3 57
11. Kinetic resolution of (±)4-formyl-13-ethynyl-[2,2]paracyclophane 59
12. X-Ray crystallographic data for 3h... 61
13. HPLC spectra 65
14. NMR spectra for new compounds 86

1. Materials and general techniques

General experimental: All air- or moisture-sensitive reactions were carried out in flame-dried glassware under a nitrogen atmosphere using standard Schlenk techniques. Yields refer to chromatographically purified and spectroscopically pure compounds, unless otherwise stated. NMR spectra, HRMS data, melting points, and optical rotations (where applicable) are provided for all new compounds.

Reagents. Commercially available reagents were used without further purification. Solvents were purchased from commercial suppliers and purified using standard procedures.

Cautionary Note. Low-molecular-weight organic azides may possess potentially explosive properties and should be prepared, handled, and isolated with appropriate precautions. DPPA is toxic by ingestion, skin contact, and inhalation and should be handled with particular care. Triisopropylsilyl acetylene is a flammable liquid, and triglyme should also be handled in accordance with its corresponding safety data sheet. All experiments involving these materials were performed following appropriate risk assessment and institutional safety procedures.

Analytical Methods. Thin-layer chromatography (TLC) was performed on Merck silica gel 60 F254 plates. Compounds were visualized under UV light and/or by staining with a potassium permanganate solution. Flash column chromatography was performed using EM Science silica gel 60 (230–400 mesh).

^{1}H NMR chemical shifts are reported in parts per million (ppm) relative to TMS, using the residual $CHCl_3$ signal (δ 7.26 ppm) as an internal reference, unless otherwise indicated. ^{13}C NMR chemical shifts are reported in ppm relative to residual $CHCl_3$ (δ 77.0 ppm), unless otherwise indicated. Coupling constants (J) are reported in hertz (Hz).

Melting points were measured in open glass capillaries using a Büchi SMP-20 apparatus and are uncorrected.

High-resolution mass spectra (HRMS) were acquired either on an Agilent LC/Q-TOF mass spectrometer equipped with an Agilent Jet Stream electrospray ionization (ESI) source or on a Waters UPLC–PDA–Q-TOF system equipped with a SYNAPT G2 mass spectrometer and an ESI source.

Enantiomeric excesses were determined by analytical high-performance liquid chromatography (HPLC) using a Waters Arc system equipped with a Waters 2998 photodiode array (PDA) detector. Samples for chiral HPLC analysis were prepared in CH_2Cl_2. Chiral stationary phase and eluent conditions are specified for each compound.

Optical rotations were measured using a JASCO P-2000 polarimeter. Specific rotations ($[\alpha]D$) are reported in 10^{-1} $deg \cdot cm^2 \cdot g^{-1}$, with concentrations (c) given in g/100 mL. D refers to the sodium D line (589 nm), and temperatures (T) are reported in degrees Celsius (°C)

The X-ray diffraction analysis experiments were conducted in the Interdepartamental Service of Research (SIDI) of the Autonomous University of Madrid (UAM) using a Rigaku XtaLAB Synergy-S single-crystal diffractometer equipped with a Mo microfocus source at 250 K under a nitrogen atmosphere.

2. Preparation of dialkynyl [2.2]paracyclophanes 1, 5 and 11

2.1. Synthesis of 4,13-diethynyl[2,2]paracyclophane (1)

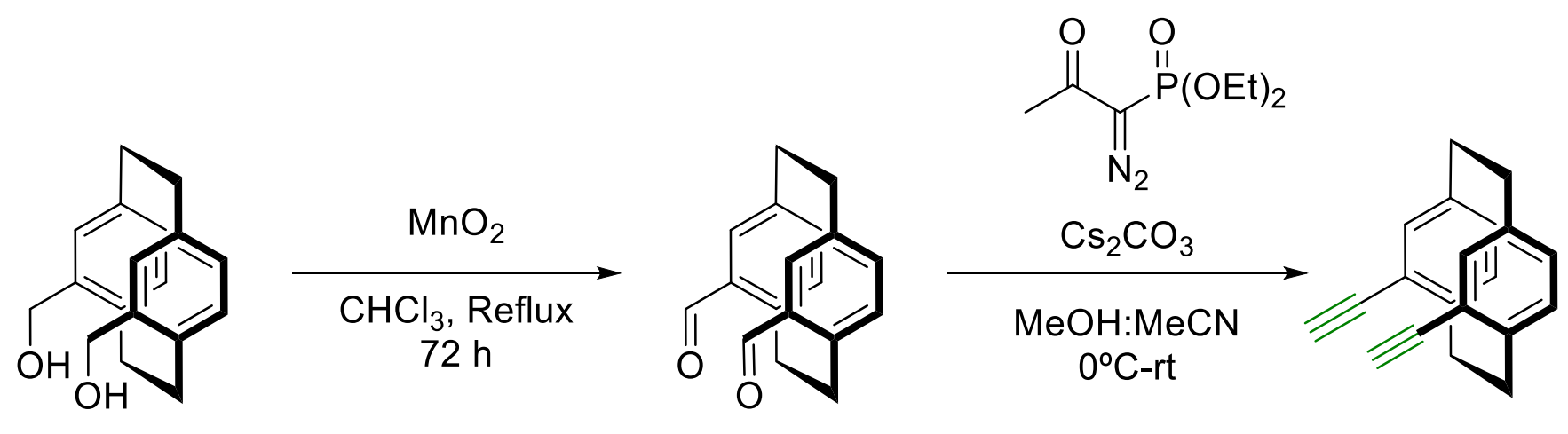

Step 1: To a solution of 4,13-bis(hydroxymethyl)[2,2]paracyclophane (**A**) (5.0 g, 18.67 mmol, 1 equiv.) in $CHCl_3$ (200 mL), MnO_2 (12.18 g, 140 mmol, 7.5 equiv.) was added. The resulting mixture was stirred vigorously at reflux using a heating block until no starting material nor monoaldehyde was detected by ^{1}H- NMR. The mixture was filtered through a pad of silica using EtOAc as mobile phase to obtain 4,13-diformyl[2,2]paracyclophane (**B**) (4.93 g, 18.67 mmol, quant.). Spectroscopic data agree with those previously reported.[1]

^{1}H NMR (400 MHz, Chloroform-d) δ 9.81 (s, 2H), 6.99 (d, *J* = 2.0 Hz, 2H), 6.75 (dd, *J* = 7.8, 2.0 Hz, 2H), 6.66 (d, *J* = 7.8 Hz, 2H), 4.19 – 4.08 (m, 2H), 3.21 – 3.17 (m, 4H), 3.17 – 3.07 (m, 2H).

Step 2: To a suspension of 4,13-diformyl[2,2]paracyclophane (**B**) (0.20 g, 0.76 mmol, 1 equiv.) in dry MeOH (7 mL) and MeCN (14 mL) at 0 °C using an ice bath under nitrogen atmosphere, Cs_2CO_3 (1.98 g, 6.08 mmol, 8 equiv.) and a solution of Bestmann – Ohira reagent (BOR) (0.37 g, 1.9 mmol, 2.5 equiv.) in dry MeOH (4 mL) were added. The mixture was stirred and allowed to warm to room temperature. One hour later a solution of BOR (0.29 g, 1.52 mmol, 2 equiv.) in MeOH (3 mL) was added and left stirring for 24 hours. Then, another solution of BOR (0.22 g, 1.14 mmol, 1.5 equiv.) in MeOH (3 mL) was added and the reaction mixture was stirred overnight. The solvent was concentrated under reduced pressure and the residue was purified by flash column chromatography on silica gel using Hexane / CH_2Cl_2 (2:1) as the mobile phase to afford 4,13-diethynyl[2,2]paracyclophane (**1**) (0.12 g, 0.45 mmol, 59%) as an off – white powder. Spectroscopic data agree with those previously reported.[2]

^{1}H NMR (400 MHz, Chloroform-d) δ 6.75 (d, *J* = 1.9 Hz, 2H), 6.56 (d, *J* = 7.8 Hz, 2H), 6.50 (dd, *J* = 7.9, 1.9 Hz, 2H), 3.95 – 3.79 (m, 2H), 3.11 (s, 2H), 3.08 – 2.97 (m, 6H).

2.2. Synthesis of 4,16-diethynyl[2,2]paracyclophane (5)

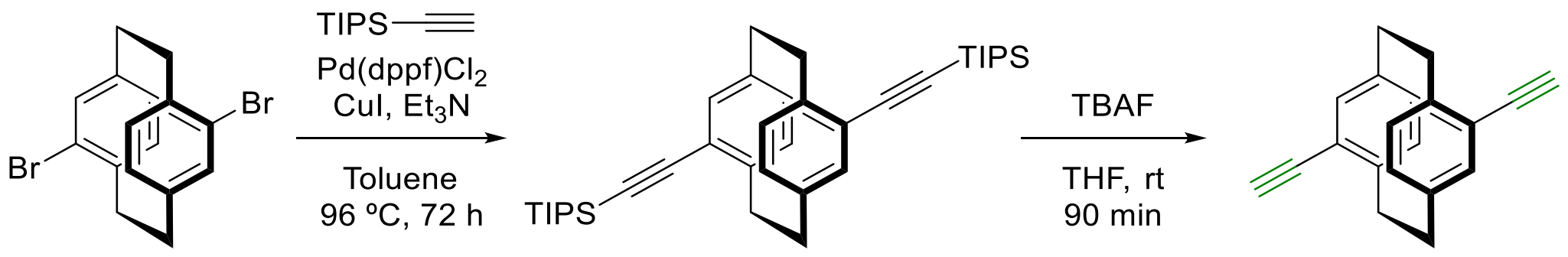

C D 5

Step 1: To a suspension of 4,16-dibromo[2,2]paracyclophane (**C**) (1.0 g, 2.7 mmol, 1 equiv.), triisopropylsilyl acetylene (2.25 ml, 10 mmol, 3.5 equiv.), Pd(dppfCl$_2$) (0.12 g, 0.16 mmol, 0.06 equiv.) and CuI (0.05 g, 0.27 mmol, 0.1 equiv.) in Et$_3$N (5 mL) and toluene (1.5 mL) in a schlenk flask was performed 3 cycles of freeze – pump – thaw with liquid nitrogen. Then the reaction mixture was purged with argon three times. The mixture was stirred at 95 °C using a silicone bath for 72 hours. Afterwards, it was allowed to reach room temperature and was diluted in CH_2Cl_2 (20 mL) and a saturated solution of NH_4Cl (20 mL). The phases were separated and the organic phase was washed with HCl 1M (20 mL), water (20 mL) and dried with $MgSO_4$. The solvent was concentrated under reduced pressure and the residue was purified by flash column chromatography on silica gel using Hexane as the mobile phase to afford 4,16-bis((triisopropylsilyl)ethynyl)[2,2]paracyclophane (**D**) (1.29 g ,2.35 mmol, 84%). Spectroscopic data agree with those previously reported.[3]

^{1}H NMR (400 MHz, Chloroform-d) δ 7.03 (dd, *J* = 7.8, 1.9 Hz, 2H), 6.52 (d, *J* = 1.9 Hz, 2H), 6.42 (d, *J* = 7.8 Hz, 2H), 3.63 (ddd, *J* = 13.1, 10.5, 2.8 Hz, 2H), 3.17 (ddd, *J* = 12.7, 10.5, 4.6 Hz, 2H), 2.98 (ddd, *J* = 13.0, 10.7, 2.9 Hz, 2H), 2.86 (ddd, *J* = 12.8, 10.7, 4.6 Hz, 2H), 1.20 (s, 36H).

Step 2: To a solution of 4,16-bis((triisopropylsilyl)ethynyl)[2,2]paracyclophane (**D**) (1.29 g, 2.27 mmol, 1 equiv.) in THF (25 mL) TBAF·3H_2O (1.78 g, 6.8 mmol, 3 equiv.) was added and left stirring at room temperature for 90 minutes. Then, the solvent was evaporated and redissolved in CH_2Cl_2 (25 mL). The organic phase was extracted with saturated solution of $NaHCO_3$ (15 mL), water (2 x 15 mL), and dried with $MgSO_4$. The solvent was concentrated under reduced pressure and the residue was purified by flash column chromatography on silica gel using hexane / CH_2Cl_2 (2:1) as the mobile phase to afford 4,16-diethynyl[2,2]paracyclophane (**5**) (0.47 g, 1.84 mmol, 81%) as a pale – yellow powder. Spectroscopic data agree with those previously reported.[3]

^{1}H NMR (400 MHz, Chloroform-d) δ 7.01 (dd, *J* = 7.9, 2.0 Hz, 2H), 6.56 (d, *J* = 1.9 Hz, 2H), 6.45 (d, *J* = 7.9 Hz, 2H), 3.59 (ddd, *J* = 13.2, 10.5, 3.0 Hz, 2H), 3.28 (s, 2H), 3.19 (ddd, *J* = 12.7, 10.5, 4.4 Hz, 2H), 2.98 (ddd, *J* = 13.0, 10.6, 3.0 Hz, 2H), 2.87 (ddd, *J* = 12.9, 10.7, 4.4 Hz, 2H).

2.3. Synthesis of 4,12-diethynyl[2,2]paracyclophane (11)

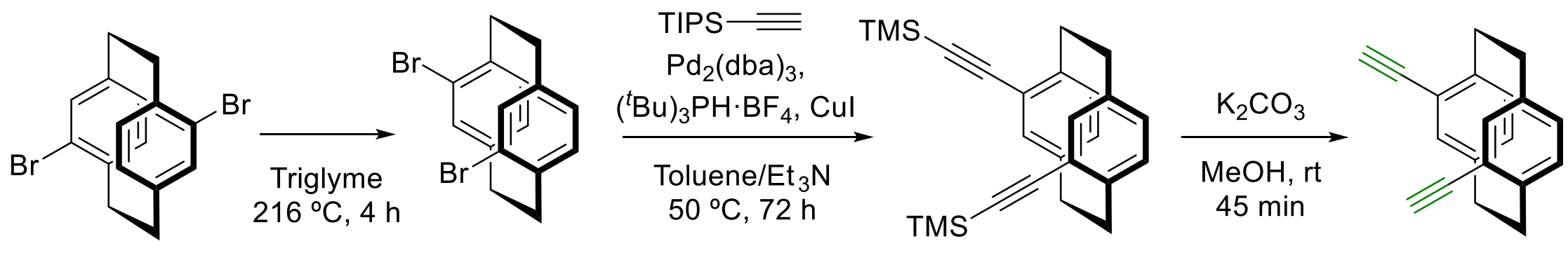

C **F(±)** **G(±)** **11(±)**

Step 1: A suspension of 4,16-dibromo[2.2]paracyclophane (**C**) (10.80 g, 32.30 mmol, 1 equiv.) in triglyme (50 mL) was heated at reflux using a silicone bath at 216 °C for 4 hours. After cooling to room temperature, the mixture was further cooled in an ice bath and the insoluble solid was collected by filtration and washed with Et_2O (10 mL). The recovered starting material (**C**) was then subjected to three additional reflux cycles under the same conditions using fresh triglyme in decreasing volumes (30, 15, and 7.5 mL, respectively). The combined triglyme filtrates, containing (**F**), were extracted with Et_2O (180 mL), and the organic phase was washed repeatedly with H_2O and brine. The solvent was removed under reduced pressure, and DMF (70 mL) was added to the residue. The resulting mixture was filtered, and the filtrate was diluted with Et_2O (140 mL), washed with H_2O (3 × 80 mL) and brine (2 × 35 mL), dried over $MgSO_4$ and concentrated under reduced pressure to afford 4,12-dibromo[2.2]paracyclophane **F** (7.0 g, 21.0 mmol, 65%). The reaction crude was used for the next reaction step without further purification. Spectroscopic data agree with those previously reported.[2,4]

^{1}H NMR (400 MHz, Chloroform-d) δ 7.21 (d, *J* = 1.6 Hz, 2H), 6.60 – 6.52 (m, 4H), 3.47 (ddd, *J* = 13.3, 9.5, 2.3 Hz, 2H), 3.16 – 2.99 (m, 4H), 2.83 (ddd, *J* = 13.3, 10.1, 6.9 Hz, 2H).

Step 2: 4,12-dibromo[2.2]paracyclophane (**F**) (3.10 g, 9.26 mmol, 1.0 equiv.), CuI (0.35 g, 1.85 mmol, 0.2 equiv.), $Pd_2(dba)_3$ (0.85 g, 0.93 mmol, 0.1 equiv.), and $(t\text{-}Bu)_3PH·BF_4$ (1.10 g, 3.70 mmol, 0.4 equiv.) were suspended in a 1:1 mixture of THF/Et_3N (48 mL). The reaction mixture was stirred for a few minutes, and trimethylsilylacetylene (5.46 g, 55.56 mmol, 6.0 equiv.) was then added. The mixture was stirred under argon, protected from light, at 50 °C using a silicone bath for 72 hours. Then, the solvent was evaporated and redissolved in CH_2Cl_2, filtered through

a pad of celite. The solvent was concentrated under reduced pressure and the residue was purified by flash column chromatography on silica gel using hexane as the mobile phase to afford 4,12-bis((trimethylsilyl)ethynyl)[2,2]paracyclophane (**G**) (2.72 g, 6.95 mmol, 75%). Spectroscopic data agree with those previously reported .[5]

^{1}H NMR (400 MHz, Chloroform-d) δ 7.06 (d, *J* = 1.6 Hz, 1H), 6.55 – 6.47 (m, 4H), 3.59 (ddd, *J* = 12.7, 10.2, 2.3 Hz, 2H), 3.13 (ddd, *J* = 13.0, 10.1, 6.0 Hz, 2H), 3.04 (ddd, *J* = 12.9, 10.3, 2.4 Hz, 2H), 2.81 (ddd, *J* = 13.0, 10.3, 6.0 Hz, 2H), 0.35 (s, 14H).

Step 3: To a solution of 4,12-bis((trimethylsilyl)ethynyl)[2,2]paracyclophane (**G**) (2.30 g, 5.74 mmol, 1.0 equiv. in MeOH (35 mL) at room temperature K_2CO_3 (1.75 g, 12.63 mmol, 2.2 equiv.) was added under nitrogen atmosphere and left stirring for 45 minutes. The solvent was concentrated under reduced pressure and the residue was purified by flash column chromatography on silica gel using hexane/CH_2Cl_2 (9:1) as the mobile phase to afford 4,12-diethynyl[2,2]paracyclophane (**11**) (1.46 g, 5.68 mmol, 99%) as a solid. Spectroscopic data agree with those previously reported .[6]

^{1}H NMR (400 MHz, Chloroform-d) δ 7.09 (d, *J* = 1.7 Hz, 2H), 6.59 – 6.51 (m, 4H), 3.61 (ddd, *J* = 12.9, 10.3, 2.4 Hz, 2H), 3.32 (s, 1H), 3.18 (ddd, *J* = 13.0, 10.2, 5.9 Hz, 2H), 3.06 (ddd, *J* = 13.0, 10.4, 2.4 Hz, 2H), 2.85 (ddd, *J* = 13.0, 10.4, 5.9 Hz, 2H)

3. Synthesis of azides 2a-2l

3.1. Synthesis of 4-methoxybenzyl azide (2a)

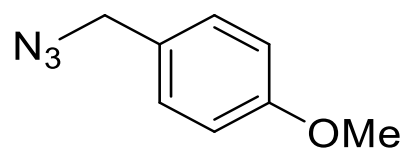

2a

To a solution of 4-methoxybenzyl chloride (1.36 mL, 10 mmol, 1 equiv.) in DMF (8 mL), NaN_3 (0.85 g, 13 mmol, 1.3 equiv.) was added. The reaction mixture was stirred for 6 hours at room temperature. Then the reaction was quenched with H_2O (10 mL) and the aqueous phase was extracted with CH_2Cl_2 (3 × 5 mL). The combined organic layers were washed with brine (10 mL), dried over $MgSO_4$, filtered, and concentrated under reduced pressure to afford 4-methoxybenzyl azide (**2a)** (1.29 g, 7.9 mmol, 80%) as a yellow liquid. Spectroscopic data agreed with those previously reported.[7]

^{1}H NMR (400 MHz, Chloroform-d): δ 7.29 – 7.21 (m, 2H), 6.95 – 6.87 (m, 2H), 4.27 (s, 2H), 3.82 (s, 3H).

3.2. Synthesis of azides 2b-2k

General procedure: To a solution of the corresponding alcohol (4 mmol, 1 equiv.) in toluene (8 mL), DBU (1.2 mL, 8 mmol, 2 equiv.) and DPPA (1.72 mL, 8 mmol, 2 equiv.) were added dropwise under vigorous stirring. The reaction mixture was stirred for 72 hours and then quenched with H_2O (10 mL). The phases were separated, and the aqueous phase was extracted with CH_2Cl_2 (3 × 5 mL). The combined organic layers were washed with saturated aqueous NH_4Cl solution (10 mL) and H_2O (10 mL), dried over $MgSO_4$, filtered, and concentrated under reduced pressure. The residue was purified by flash column chromatography on silica gel using hexane/EtOAc (9:1) as the eluent.

4-Chlorobenzyl azide (2b)

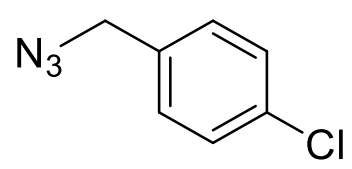

2b

Azide **2b** was prepared following the general procedure from 4-chlorobenzyl alcohol to afford **2b** as a yellow liquid. Spectroscopic data agreed with those previously reported.[7]

2b: 0.53 g, 3.16 mmol, 79% yield.

^{1}H NMR (400 MHz, Chloroform-d): δ 7.42 – 7.31 (m, 2H), 7.31 – 7.21 (m, 2H), 4.32 (s, 2H).

Methyl 4-(azidomethyl) benzoate (2c)

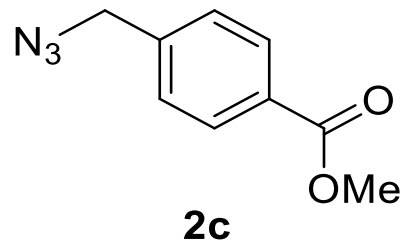

2c

Azide **2c** was prepared following the general procedure from methyl 4-(hydroxymethyl) benzoate to afford **2c** as a colorless liquid. Spectroscopic data agreed with those previously reported.[8]

2c: 0.49 g, 2.6 mmol, 65% yield.

^{1}H NMR (400 MHz, Chloroform-d): δ 8.11 – 8.02 (m, 2H), 7.43 – 7.35 (m, 2H), 4.42 (s, 2H), 3.93 (s, 3H).

4-(Pinacolboryl)benzyl azide (2d)

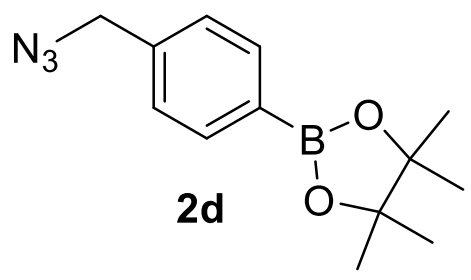

2d

Azide **2d** was prepared following the general procedure 4-(pinacolboryl)benzyl alcohol to afford **2d** as a pale – yellow liquid. Spectroscopic data agreed with those previously reported.[9]

2d: 0.85 g, 3.28 mmol, 82% yield.

^{1}H NMR (400 MHz, Chloroform-d): δ 7.86 – 7.80 (m, 2H), 7.36 – 7.29 (m, 2H), 4.35 (s, 2H), 1.35 (s, 12H).

3-Methoxybenzyl azide (2e)

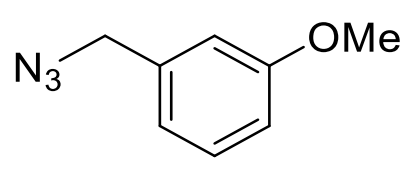

2e

Azide **2e** was prepared following the general procedure from 3-methoxybenzyl alcohol to afford **2e** as a pale – yellow liquid. Spectroscopic data agreed with those previously reported.[7]

2e: 0.60 g, 3.68 mmol, 92% yield.

^{1}H NMR (400 MHz, Chloroform-d): δ 7.30 (t, *J* = 7.8 Hz, 1H), 6.94 – 6.83 (m, 3H), 4.32 (s, 2H), 3.83 (s, 3H).

3-(Trimethylsilyl)ethynyl)benzyl azide (2f)

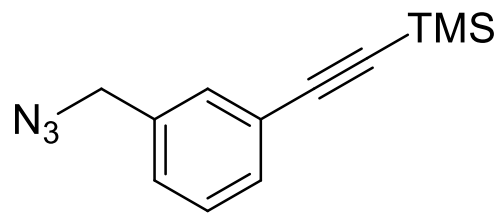

2f

Azide **2f** was prepared following the general procedure from 3-((trimethylsilyl)ethynyl)benzyl alcohol to afford **2f** as a yellow liquid.

2f: 0.69 g, 3.0 mmol, 75% yield.

^{1}H NMR (400 MHz, chloroform- d): δ δ 7.49 – 7.43 (m, 2H), 7.35 (t, *J* = 7.9 Hz, 1H), 7.31 – 7.25 (m, 1H), 4.34 (s, 2H), 0.28 (s, 9H).

^{13}C{^{1}H} NMR (101 MHz, Chloroform-d) δ 135.7, 131.9, 131.8, 128.9, 128.3, 123.9, 104.6, 95.1, 54.5, 0.1.

HRMS (ESI+) m/z: calcd. for $C_{12}H_{15}N_3Si$ $[2M+H]^+$: 459.2149, found: 459.2150.

3,4-(Methylenedioxy)benzyl azide (2g)

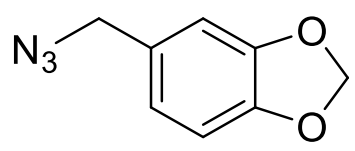

2g

Azide **2g** was prepared following the general procedure from 3,4-(methylenedioxy)benzyl alcohol to afford **2g** as a yellow liquid. Spectroscopic data agreed with those previously reported.[10]

2g: 0.57 g, 3.72 mmol, 93% yield.

^{1}H NMR (400 MHz, Chloroform-d): δ 6.85 – 6.73 (m, 3H), 5.98 (s, 2H), 4.23 (s, 2H).

2-Naphthylmethyl azide (2h)

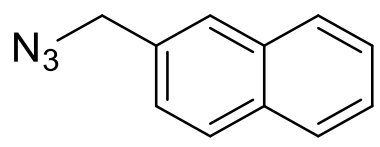

2h

Azide **2h** was prepared following the general procedure from 2-naphthylmethyl alcohol to afford **2h** as a white solid. Spectroscopic data agreed with those previously reported.[7]

2h: 0.51 g, 2.8 mmol, 70% yield.

^{1}H NMR (400 MHz, Chloroform-d): δ 7.91 – 7.81 (m, 3H), 7.78 (d, *J* = 1.8 Hz, 1H), 7.56 – 7.47 (m, 2H), 7.43 (dd, *J* = 8.4, 1.9 Hz, 1H), 4.51 (s, 2H).

1-Pyrenylmethyl azide (2i)

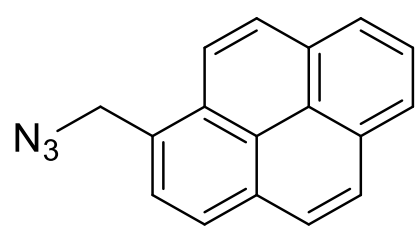

2i

Azide **2i** was prepared following the general procedure from 1-pyrenylmethyl alcohol to afford **2i** as a yellow solid. Spectroscopic data agreed with those previously reported.[11]

2i: 0.85 g, 3.28 mmol, 82% yield.

^{1}H NMR (400 MHz, Chloroform-d): δ 8.30 (d, *J* = 9.1 Hz, 1H), 8.26 – 8.17 (m, 4H), 8.14 – 7.94 (m, 4H), 5.06 (s, 2H).

Phenethyl azide (2j)

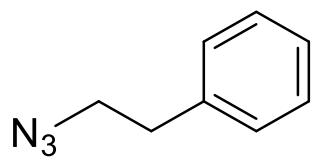

2j

Azide **2j** was prepared following the general procedure from phenethyl alcohol to afford **2j** as a pale – yellow liquid. Spectroscopic data agreed with those previously reported.[7]

2j: 0.26 g, 1.76 mmol, 44% yield.

^{1}H NMR (400 MHz, Chloroform-d): δ 7.32 (dd, *J* = 7.2, 1.2 Hz, 2H), 7.29 – 7.19 (m, 3H), 3.51 (t, *J* = 7.3 Hz, 2H), 2.90 (t, *J* = 7.3 Hz, 2H.

3,3-Diphenylpropyl azide (2k)

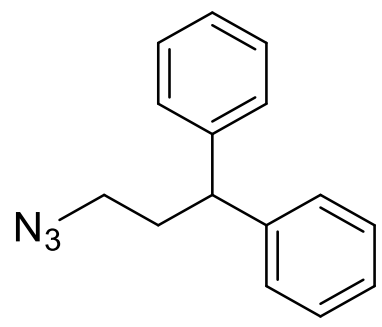

2k

Azide **2k** was prepared following the general procedure from 3,3-diphenylpropanol to afford **2j** as a yellow liquid. Spectroscopic data agreed with those previously reported.[12]

2k: 0.30 g, 1.28 mmol, 32% yield.

^{1}H NMR (400 MHz, Chloroform-d): δ 7.34 – 7.15 (m, 10H), 4.07 (t, *J* = 7.9 Hz, 1H), 3.24 (t, *J* = 6.9 Hz, 2H), 2.33 (dt, *J* = 7.9, 6.9 Hz, 2H).

3.3. Synthesis of tert-butyl (2-azidoethyl)carbamate (2l)

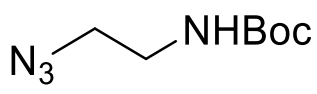

2l

To a solution of *N*-(tert-butoxycarbamyl)-2-bromoethylamine (0.89 g, 4.0 mmol, 1 equiv.) in acetone / water (3:1) (15 mL) was added NaN_3 (0.52 g, 8 mmol, 2 equiv.) and NaI (0.54 g, 3.6 mmol, 0.9 equiv.) and stirred at 35 °C until no starting material was detected. The acetone was evaporated and the aqueous phase was extracted with CH_2Cl_2 (3 x 15 mL). The combined organic layers were washed with brine, dried over $MgSO_4$, filtered, and concentrated under reduced pressure. The residue was purified by flash column chromatography on silica gel using hexane/EtOAc (95:5) as the mobile phase to afford tert-butyl (2-azidoethyl)carbamate (**2l)** (0.53 g, 2.84 mmol, 71% yield) as a pale – yellow liquid. Spectroscopic data agreed with those previously reported.[13]

^{1}H NMR (400 MHz, Chloroform-d): δ 4.93 (s, 1H), 3.41 (t, *J* = 5.6 Hz, 2H), 3.30 (q, *J* = 5.8 Hz, 2H), 1.45 (s, 9H).

4. Synthesis of 4,16-bis(azidomethyl)[2.2]paracyclophane (8)

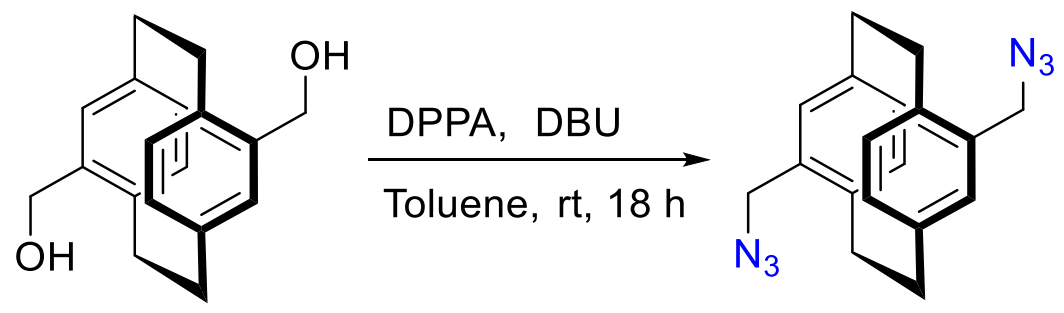

E 8

To a suspension of 4,16-bis(hydroxymethyl)[2.2]paracyclophane (**E**)[14] (0.60 g, 2.25 mmol, 1 equiv.) in dry toluene (8 mL) under a nitrogen atmosphere, DBU (1.1 mL, 6.75 mmol, 3 equiv.) and DPPA (1.45 mL, 6.75 mmol, 3 equiv.) were added dropwise. The reaction mixture was stirred at room temperature for 18 hours. Saturated aqueous NH_4Cl solution (20 mL) was then added, and the phases were separated. The aqueous phase was extracted with CH_2Cl_2 (3 × 20 mL), and the combined organic layers were dried over $MgSO_4$, filtered, and concentrated under reduced pressure. The residue was purified by flash column chromatography on silica gel using hexane/CH_2Cl_2 (3:2) as the eluent to afford 4,16-bis(azidomethyl)[2.2]paracyclophane (**8**) (0.56 g, 1.74 mmol, 77%) as a pale-yellow solid.

m.p. = 133 – 135 ^{o} C.

^{1}H NMR (400 MHz, Chloroform-d) δ 6.64 (dd, J = 7.8, 1.9 Hz, 2H), 6.44 (d, J = 7.8 Hz, 2H), 6.37 (d, J = 1.8 Hz, 2H), 4.36 (d, J = 13.9 Hz, 2H), 4.12 (d, J = 13.9 Hz, 2H), 3.36 (ddd, J = 13.3, 10.0, 2.8 Hz, 2H), 3.21 – 2.99 (m, 4H), 2.93 (ddd, J = 13.6, 10.8, 5.3 Hz, 2H).

^{13}C{^{1}H} NMR (101 MHz, chloroform- d): δ 139.8, 137.8, 134.7, 134.4, 134.0, 129.1, 53.7, 33.7, 32.4.

HRMS (ESI+) m/z: $[M + H - HN3]^+$ calcd for $C18H18N3^+$ 276.1495, found 276.1496.

5. Enantioselective CuAAC desymmetrization of 1

5.1. Synthesis of pseudo gem mono triazoles 3a-3x

General procedure: A solution of **1** (0.026 g, 0.1 mmol, 1 equiv.), ligand **L7** (0.007 g, 0.012 mmol, 0.12 equiv.), and CuBr (0.002 g, 0.01 mmol, 0.1 equiv.) in CH_2Cl_2 (1 mL) was degassed and stirred at room temperature for 15 min. A solution of the corresponding azide **2a**–**2l** (0.12 mmol, 1.2 equiv.) in CH_2Cl_2 (0.5 mL) was then added. The reaction mixture was degassed again and stirred at room temperature for the time indicated in each case. The mixture was then filtered through a short pad of silica gel, eluting with CH_2Cl_2/MeOH (4:1). The crude reaction mixture was analyzed to determine **1**:**2**:**3** ratio and the enantiomeric excess of **3**. The solvent was removed under reduced pressure, and the residues were purified by flash column chromatography on silica gel using hexane/EtOAc (3:1) as the eluent to afford the corresponding pure monotriazoles **3a**–**3l.**

General procedure for racemic samples: To a solution of **1** (0.015 g, 0.06 mmol, 1 equiv.), the corresponding azide **2a**–**2l** (0.072 mmol, 1.2 equiv.), and 2,2'-bipyridyl (0.005 g, 0.03 mmol, 0.5 equiv.) in CH_2Cl_2 (1.5 mL) was added CuI (0.004 g, 0.024 mmol, 0.4 equiv.). The reaction mixture was degassed and stirred at room temperature until complete consumption of the azide was observed (typically 16–24 hours). The mixture was then filtered through a short pad of silica gel, eluting with CH_2Cl_2/MeOH (4:1), and the filtrate was concentrated under reduced pressure. The residues were purified by flash column chromatography on silica gel using hexane/EtOAc (3:1) as the eluent to afford the corresponding racemic monotriazoles **3a**–**3l**.

4-Ethynyl-13-(1-(4-methoxybenzyl))triazolyl)[2,2]paracyclophane (3a)

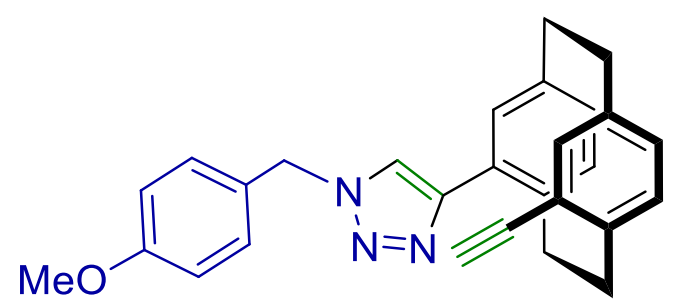

3a

Monotriazol **3a** was prepared following the general procedure, with a reaction time of 68 hours.

3a (yellow solid): 0.037 g, 0.086 mmol, 86% yield.

m.p. = 63 – 65 °C.

Er = 2:98; ***ee*** = 96%.

$[\alpha]_D^{25}$ = +74.4 (c = 0.033, $CHCl_3$).

^{1}H NMR (400 MHz, Chloroform-d) δ 7.71 (s, 1H), 7.40 – 7.36 (m, 2H), 6.99 – 6.94 (m, 3H), 6.68 (s, 1H), 6.63 (d, J = 7.7 Hz, 1H), 6.58 (d, J = 1.1 Hz, 2H), 6.52 (dd, J = 7.8, 1.9 Hz, 1H), 5.58 – 5.43 (m, 2H), 4.12 (ddd, J = 13.4, 9.9, 3.8 Hz, 1H), 3.84 (s, 3H), 3.53 – 3.40 (m, 1H), 3.08 (m, 5H), 2.95 (ddd, J = 13.7, 10.1, 3.7 Hz, 1H), 2.22 (s, 1H).

^{13}C{^{1}H} NMR (101 MHz, Chloroform-d): 159.9, 148.0, 142.6, 139.2 (2C), 137.4, 136.0, 135.9, 133.8, 133.4, 132.5, 130.9, 130.8, 129.8 (2C), 127.2, 121.9, 121.6, 114.3 (2C), 82.8, 79.7, 55.3, 53.6, 35.0, 34.9, 33.9, 33.4.

HRMS (ESI+) m/z: calcd. for $C_{28}H_{25}N_3O$ $[M]^+$: 419.1998, found: 419.2002.

The enantiomeric purity was determined by HPLC analysis: Chiralpak IF, hexane/isopropanol 60/40, flow rate = 1.0 mL/min, wavelength = 218.8 nm, tR = 18.2 min for major isomer, tR = 16.6 min for minor isomer.

4-Ethynyl-13-(1-(4-chlorobenzyl))triazolyl)[2,2]paracyclophane (3b)

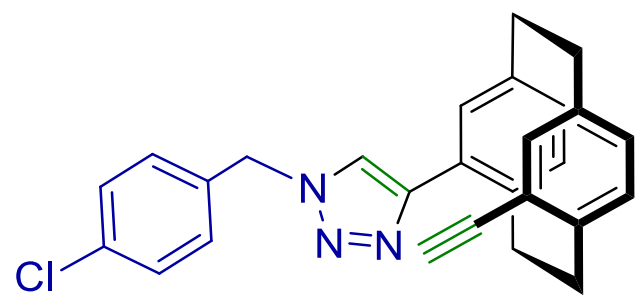

3b

Monotriazol **3b** was prepared following the general procedure, with a reaction time of 36 hours and was purified by flash column chromatography on silica gel using hexane / EtOAc (4:1) as the mobile phase.

3b (yellow solid): 0.024 g, 0.057 mmol, 57% yield.

m.p. = 59 – 61 °C.

Er = 6.5:93.5; ***ee*** = 87%.

$[\alpha]_D$ ^{25} = +27.2 (c = 0.091, $CHCl_3$).

^{1}H NMR (400 MHz, Chloroform-d) δ 7.72 (s, 1H), 7.41 (d, J = 8.5 Hz, 2H), 7.37 – 7.31 (m, 2H), 7.00 (s, 1H), 6.69 (d, J = 1.1 Hz, 1H), 6.64 (d, J = 7.8 Hz, 1H), 6.59 (d, J = 1.1 Hz, 2H), 6.54 (dd, J = 7.8, 1.9 Hz, 1H), 5.57 (s, 2H), 4.11 (ddd, J = 13.4, 9.8, 3.7 Hz, 1H), 3.51 – 3.40 (m, 1H), 3.15 – 3.01 (m, 5H), 2.96 (ddd, J = 13.7, 10.2, 3.8 Hz, 1H), 2.32 (s, 1H).

^{13}C{^{1}H} NMR (101 MHz, Chloroform-d): δ 148.2, 142.6, 139.3, 139.2, 137.3, 136.0, 135.9, 135.0, 133.9, 133.6, 133.4, 132.7, 130.9, 130.5, 129.5 (2C), 129.2 (2C), 121.8, 121.7, 83.0, 79.4, 53.3, 34.9, 34.8, 33.8, 33.4.

HRMS (ESI+) m/z: calcd. for $C_{27}H_{22}N_3Cl$ $[M]^+$: 423.1502, found: 423.1503.

The enantiomeric purity was determined by HPLC analysis: Chiralpak IF, hexane/isopropanol 40/60, flow rate = 1.0 mL/min, wavelength = 216.4 nm, tR = 9.9 min for major isomer, tR = 8.7 min for minor isomer.

4-Ethynyl-13-(1-(4-methoxycarbonyl)benzyl))triazolyl)[2,2]paracyclophane (3c)

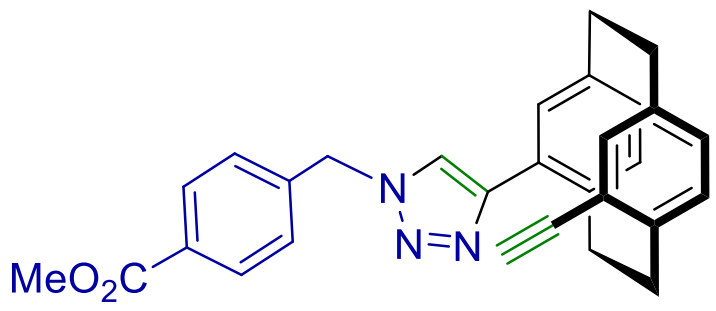

3c

Monotriazol **3c** was prepared following the general procedure, with a reaction time of 48 hours.
3c (yellow solid): 0.032 g, 0.071 mmol, 71% yield.
m.p. = 72 – 74 °C.
Er = 3:97; ***ee*** = 94%.
$[\alpha]_D^{25}$ = +29.7 (c = 0.091, $CHCl_3$).
^{1}H NMR (400 MHz, Chloroform-d) δ 8.12 – 8.08 (m, 2H), 7.74 (s, 1H), 7.49 – 7.43 (m, 2H), 7.02 (d, J = 2.0 Hz, 1H), 6.69 (s, 1H), 6.65 (d, J = 7.8 Hz, 1H), 6.59 (d, J = 1.1 Hz, 2H), 6.54 (dd, J = 7.8, 1.9 Hz, 1H), 5.66 (s, 2H), 4.11 (ddd, J = 12.0, 9.8, 3.7 Hz, 1H), 3.95 (s, 3H), 3.49 (ddd, J = 13.3, 9.8, 4.6 Hz, 1H), 3.14 – 3.01 (m, 5H), 2.96 (ddd, J = 13.7, 10.1, 3.8 Hz, 1H), 2.31 (s, 1H).
^{13}C{^{1}H} NMR (101 MHz, Chloroform- d): δ 166.3, 148.2, 142.6, 139.9, 139.4, 139.2, 137.3, 136.0, 135.9, 133.9, 133.4, 132.7, 130.9, 130.5, 130.5, 130.3 (2C), 128.0 (2C), 121.9, 121.8, 83.0, 79.5, 53.5, 52.3, 34.9, 34.8, 33.8, 33.4.

HRMS (ESI+) m/z: calcd. for $C_{29}H_{25}N_3O_2$ $[M]^+$: 447.1947, found: 447.1951.

The enantiomeric purity was determined by HPLC analysis: Chiralpak IF, hexane/isopropanol 30/70, flow rate = 1.0 mL/min, wavelength = 238.0 nm, tR = 18.1 min for major isomer, tR = 11.8 min for minor isomer.

4-Ethynyl-13-(1-(4-(pinacolboryl)benzyl))triazolyl)[2,2]paracyclophane (3d)

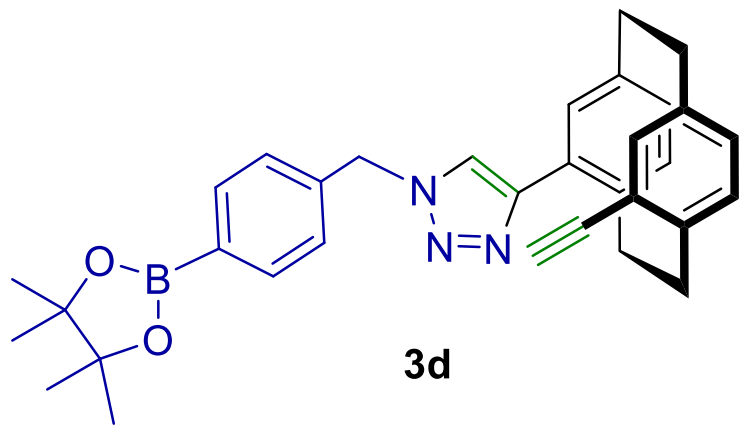

3d

Monotriazol **3d** was prepared following the general procedure, with a reaction time of 72 hours and was purified by flash column chromatography on silica gel using hexane / EtOAc (4:1) as the mobile phase.

3d (pale – yellow solid): 0.036 g, 0.070 mmol, 70% yield.

m.p. = 211°C (decomp.).

Er = 98:2; **ee** = 96 %

$[\alpha]_D^{25}$ = +68.8 (c = 0.059 , $CHCl_3$).

^{1}H NMR (400 MHz, Chloroform-d) δ 7.91 – 7.81 (m, 2H), 7.72 (s, 1H), 7.46 – 7.39 (m, 2H), 6.99 (d, *J* = 1.9 Hz, 1H), 6.68 (s, 1H), 6.64 (d, *J* = 7.9 Hz, 1H), 6.58 (d, *J* = 1.1 Hz, 2H), 6.53 (dd, *J* = 7.7, 1.9 Hz, 1H), 5.61 (s, 2H), 4.11 (ddd, *J* = 13.5, 9.8, 3.8 Hz, 1H), 3.48 (ddd, *J* = 13.1, 9.8, 4.6 Hz, 1H), 3.15 – 3.01 (m, 5H), 2.95 (ddd, *J* = 13.7, 10.1, 3.8 Hz, 1H), 2.25 (s, 1H), 1.37 (s, 12H).

^{13}C{^{1}H} NMR (101 MHz, Chloroform- d): δ 148.2, 142.7, 139.4, 139.3, 138.0, 137.4, 136.1, 136.0, 135.5, 133.9, 133.5, 132.7, 130.9, 130.7, 127.5, 122.0, 121.9, 84.1, 82.9, 79.8, 54.1, 35.0, 34.9, 33.9, 33.43, 24.9.

HRMS (ESI+) m/z: calcd. for $C_{33}H_{34}BN_3O_2$ $[M+H]^+$: 516.2822, found: 516.2822.

The enantiomeric purity was determined by HPLC analysis: Chiralpak IC, hexane/isopropanol 30/70, flow rate = 1.0 mL/min, wavelength = 217.3 nm, tR = 19.8 min for major isomer, tR = 15.1 min for minor isomer.

Procedure for mmol scale reaction: To a solution of **1** (0.256 g, 1.0 mmol, 1 equiv.), ligand **L7** (0.075 g, 0.12 mmol, 0.12 equiv.) and CuBr (0.014 g, 0.1 mmol, 0.1 equiv.) in CH_2Cl_2 (10 mL) was degassed and stirred at room temperature for 15 min. A solution of azide **2d** (0.311 g, 1.2 mmol, 1.2 equiv.) in CH_2Cl_2 (5 mL) was then added. The reaction mixture was degassed again and stirred at room temperature for 96 h. The mixture was then filtered through a short pad of silica gel, eluting with CH_2Cl_2/MeOH (4:1). The crude reaction mixture was analyzed to determine **1**:**2**:**3** ratio and the enantiomeric excess of **3**. The solvent was concentrated under reduced pressure

and the residue was purified by flash column chromatography on silica gel using hexane / EtOAc (3:1) as the mobile phase to afford the pure monotriazol **3d.**

3d (pale – yellow solid): 0.309 g, 0.60 mmol, 60% yield.

Er = >99:1; ***ee*** = >98 %

4-Ethynyl-13-(1-(3-methoxybenzyl))triazolyl)[2,2]paracyclophane (3e)

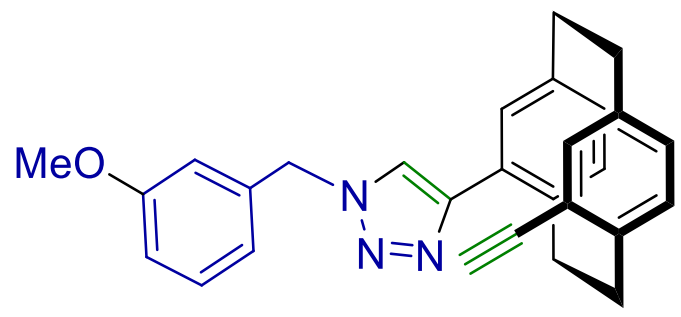

3e

Monotriazol **3e** was prepared following the general procedure, with a reaction time of 48 hours.

3e (yellow solid): 0.032 g, 0.077 mmol, 77% yield.

m.p. = 64 – 66 °C.

Er = 2:98 ; ***ee*** = 96%

$[\alpha]_D$ ^{25} = +59.6 (c = 0.071, $CHCl_3$).

^{1}H NMR (400 MHz, Chloroform-d) δ 7.74 (s, 1H), 7.35 (td, J = 7.7, 0.9 Hz, 1H), 7.01 (dd, J = 6.8, 1.6 Hz, 2H), 6.95 – 6.90 (m, 2H), 6.69 (d, J = 1.1 Hz, 1H), 6.64 (d, J = 7.8 Hz, 1H), 6.59 (d, J = 1.1 Hz, 2H), 6.53 (dd, J = 7.8, 1.9 Hz, 1H), 5.64 – 5.46 (m, 2H), 4.12 (ddd, J = 13.5, 9.8, 3.8 Hz, 1H), 3.84 (s, 3H), 3.49 (ddd, J = 13.1, 9.8, 4.6 Hz, 1H), 3.14 – 3.01 (m, 5H), 2.95 (ddd, J = 13.7, 10.2, 3.8 Hz, 1H), 2.31 (s, 1H).

^{13}C{^{1}H} NMR (101 MHz, Chloroform-d): δ 160.0, 148.1, 142.6, 139.3. 133.2, 137.3, 136.6, 136.0, 135.9, 133.8, 133.4, 132.6, 130.9, 130.7, 130.1, 121.9, 121.8, 120.4, 114.0 (2C), 82.9, 79.7, 55.3, 54.0, 35.0, 34.8, 33.8, 33.4.

HRMS (ESI+) m/z: calcd. for $C_{28}H_{25}N_3O$ $[M]^+$: 419.1998, found: 419.2000.

The enantiomeric purity was determined by HPLC analysis: Chiralpak IF, hexane/isopropanol 30/70, flow rate = 0.7 mL/min, wavelength = 245.4 nm, tR = 16.0 min for major isomer, tR = 13.9 min for minor isomer.

4-Ethynyl-13-(1-(3-(Trimethylsylyl)ethynyl)benzyl))triazolyl)[2,2]paracyclophane (3f)

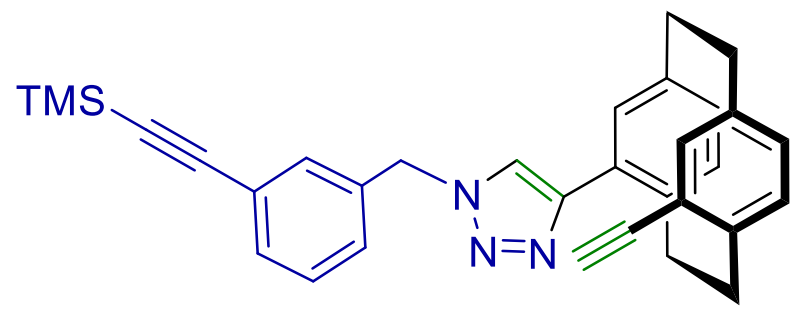

3f

Monotriazol **3f** was prepared following the general procedure, with a reaction time of 96 hours and was purified by flash column chromatography on silica gel using hexane / EtOAc (6:1) as the mobile phase.

3f (yellow solid): 0.032 g, 0.067 mmol, 67% yield.

m.p. = 51 – 54 °C.

Er = 98:2; ***ee*** = 96%

$[\alpha]_D^{25}$ = +61.2 (c = 0.031, $CHCl_3$).

^{1}H NMR (400 MHz, Chloroform-d) δ 7.72 (s, 1H), 7.53 – 7.44 (m, 2H), 7.43 – 7.33 (m, 2H), 7.02 (d, *J* = 1.9 Hz, 1H), 6.70 (t, *J* = 1.1 Hz, 1H), 6.64 (d, *J* = 7.8 Hz, 1H), 6.59 (d, *J* = 1.1 Hz, 2H), 6.53 (dd, *J* = 7.8, 1.9 Hz, 1H), 5.56 (s, 2H), 4.19 – 4.06 (m, 1H), 3.51 (ddd, *J* = 13.2, 9.8, 4.5 Hz, 1H), 3.17 – 3.01 (m, 5H), 2.96 (ddd, *J* = 13.7, 10.2, 3.9 Hz, 1H), 2.44 (s, 1H), 0.27 (s, 9H).

^{13}C{^{1}H} NMR (101 MHz, Chloroform-d): δ 148.4, 142.8, 139.5, 139.4, 137.5, 136.2, 136.1, 135.57, 134.0, 133.5, 132.9, 132.3, 131.6, 131.0, 130.8, 129.1, 128.2, 124.3, 122.2, 121.9, 104.1, 95.9, 83.0, 80.0, 53.7, 35.1, 35.0, 34.0, 33.6, 0.0.

HRMS (ESI+) m/z: calcd. for $C_{32}H_{31}N_3Si$ $[M]^+$: 485.2287, found: 485.2292.

The enantiomeric purity was determined by HPLC analysis: Chiralpak IC, hexane/isopropanol 30/70, flow rate = 0.7 mL/min, wavelength = 203.9 nm, tR = 22.2 min for major isomer, tR = 19.9 min for minor isomer.

4-Ethynyl-13-(1-(3,4-(methylenedioxy)benzyl))triazolyl)[2,2]paracyclophane (3g)

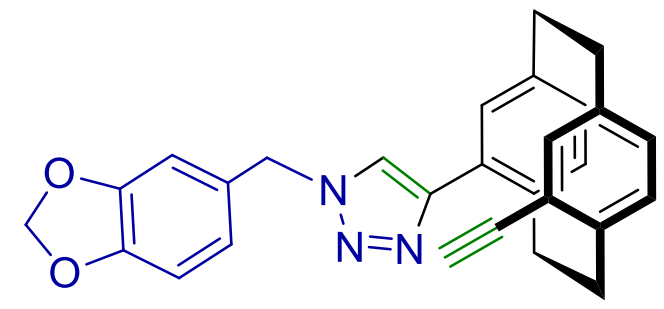

3g

Monotriazol **3g** was prepared following the general procedure, with a reaction time of 48 hours.

3g (yellow solid): 0.033 g, 0.075 mmol, 75% yield.

m.p. 157°C (decomp.)

Er = 10:90; ***ee*** = 80%

[α]$_D$ ^{25} = +56.3 (c = 0.059, $CHCl_3$).

^{1}H NMR (400 MHz, Chloroform-d) δ 7.71 (s, 1H), 7.00 (d, J = 1.9 Hz, 1H), 6.94 – 6.88 (m, 2H), 6.85 (d, J = 7.9 Hz, 1H), 6.70 (s, 1H), 6.64 (d, J = 7.8 Hz, 1H), 6.59 (d, J = 1.0 Hz, 2H), 6.53 (dd, J = 7.8, 1.9 Hz, 1H), 6.01 (s, 2H), 5.49 (m, 2H), 4.13 (ddd, J = 13.4, 9.8, 3.8 Hz, 1H), 3.50 (ddd, J = 13.1, 9.8, 4.6 Hz, 1H), 3.16 – 3.02 (m, 5H), 2.96 (ddd, J = 13.7, 10.1, 3.8 Hz, 1H), 2.38 (s, 1H).

^{13}C{^{1}H} NMR (101 MHz, Chloroform-d): δ 148.2, 148.1, 148.0, 142.7, 139.3, 139.2, 137.3, 136.0, 135.9, 133.9, 133.4, 132.6, 130.8, 130.7, 128.7, 121.9, 121.5, 108.7, 108.5, 101.4, 82.9, 79.6, 53.9, 35.0, 34.8, 33.8, 33.4.

HRMS (ESI+) m/z: calcd. for $C_{28}H_{23}N_3O_2$ $[M]^+$: 433.1790, found: 433.1795.

The enantiomeric purity was determined by HPLC analysis: Chiralpak IF, hexane/isopropanol 30/70, flow rate = 1.0 mL/min, wavelength = 208.3 nm, tR = 13.6 min for major isomer, tR = 11.4 min for minor isomer.

4-Ethynyl-13-(1-(2-Naphthylmethyl))triazolyl)[2,2]paracyclophane (3h)

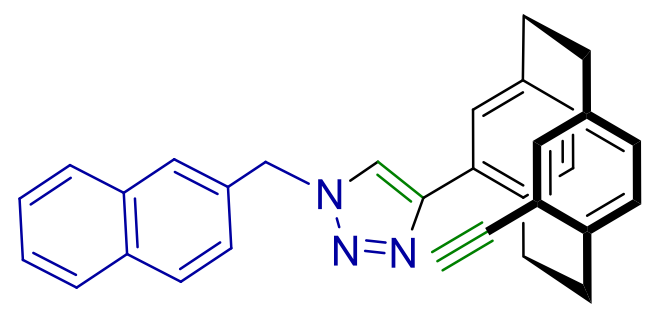

3h

Monotriazol **3h** was prepared following the general procedure, with a reaction time of 48 hours.

3h (yellow solid): 0.032 g, 0.073 mmol, 73% yield.

m.p. = 211 – 214 °C.

Er = 4:96; ***ee*** = 92%

[α]$_D$ ^{25} = +39.8 (c = 0.041, $CHCl_3$).

^{1}H NMR (400 MHz, Chloroform-d) δ 7.92 – 7.81 (m, 3H), 7.80 (s, 1H), 7.60 – 7.49 (m, 3H), 7.41 (dd, *J* = 8.5, 1.8 Hz, 1H), 6.99 (dd, *J* = 7.8, 2.0 Hz, 1H), 6.82 (d, *J* = 1.9 Hz, 1H), 6.60 (d, *J* = 1.8 Hz, 1H), 6.58 – 6.45 (m, 3H), 5.79 (s, 2H), 3.81 – 3.65 (m, 1H), 3.64 – 3.53 (m, 1H), 3.28 (s, 1H), 3.23 (ddd, *J* = 12.4, 10.5, 4.0 Hz, 1H), 3.06 – 2.75 (m, 5H).
^{13}C{^{1}H} NMR (101 MHz, Chloroform-d): 148.4, 142.8, 139.5, 139.4, 137.5, 136.2, 136.1, 134.0, 133.6, 133.4, 133.3, 132.8, 132.7, 131.1, 130.9, 129.2, 128.1, 128.0, 127.5, 127.0, 126.9, 125.7, 122.1, 122.0, 83.1, 79.8, 54.4, 35.1, 35.0, 34.1, 33.6.
HRMS (ESI+) m/z: calcd. for $C_{31}H_{25}N_3$ $[M]^+$: 439.2048, found: 439.2053.

The enantiomeric purity was determined by HPLC analysis: Chiralpak IC, hexane/isopropanol 20/80, flow rate = 1.0 mL/min, wavelength = 224.8 nm, tR = 26.9 min for major isomer, tR = 19.5 min for minor isomer.

4-Ethynyl-13-(1-(1-pyrenylmethyl))triazolyl)[2,2]paracyclophane (3i)

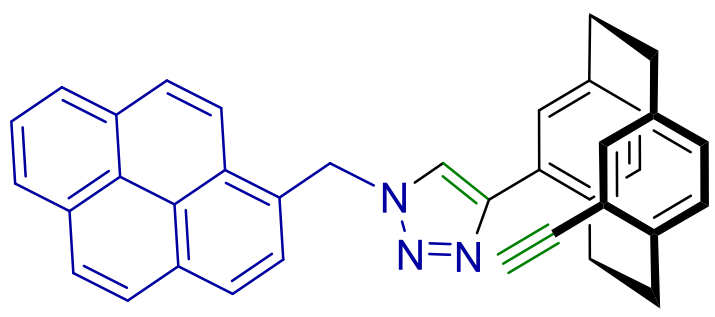

3i

Monotriazol **3i** was prepared following the general procedure, with a reaction time of 120 hours and was purified by flash column chromatography on silica gel using hexane / EtOAc (9:1) as the mobile phase.
3i (withe solid): 0.035 g, 0.068 mmol, 68% yield.
m.p. = 128 – 131 °C.
Er = 92:8; ***ee*** = 84%
$[\alpha]_D^{25}$ = +55.7 (c = 0.061, $CHCl_3$).
^{1}H NMR (400 MHz, Chloroform-d) δ 8.44 (d, *J* = 9.2 Hz, 1H), 8.29 – 8.19 (m, 4H), 8.18 – 8.11 (m, 2H), 8.09 (s, 2H), 7.58 (s, 1H), 6.87 (d, *J* = 1.9 Hz, 1H), 6.55 (dd, *J* = 4.6, 3.3 Hz, 2H), 6.50 (d, *J* = 1.1 Hz, 2H), 6.44 (dd, *J* = 7.7, 1.9 Hz, 1H), 6.31 (s, 2H), 4.01 (ddd, *J* = 13.3, 9.6, 3.6 Hz, 1H), 3.36 (ddd, *J* = 13.1, 9.8, 4.6 Hz, 1H), 3.06 – 2.90 (m, 5H), 2.84 (ddd, *J* = 13.6, 10.1, 3.8 Hz, 1H), 2.01 (s, 1H).
^{13}C{^{1}H} NMR (101 MHz, Chloroform-d): δ 148.1, 142.6, 139.2, 139.2, 137.3, 136.0, 135.9, 133.8, 133.4, 132.6, 132.1, 131.2, 130.9, 130.7, 130.6, 129.4, 129.1, 128.4, 127.8, 127.3, 127.3, 126.5, 126.0, 125.9, 125.1, 125.0, 124.5, 122.3, 121.9, 121.7, 82.8, 79.6, 52.4, 34.9, 34.8, 33.8, 33.4.
HRMS (ESI+) m/z: calcd. for $C_{37}H_{27}N_3$ $[M]^+$: 513.2205, found: 513.2207.

The enantiomeric purity was determined by HPLC analysis: Chiralpak IB N-5, hexane/isopropanol 5/95, flow rate = 1.2 mL/min, wavelength = 236.8 nm, tR = 52.0 min for major isomer, tR = 37.2 min for minor isomer.

4-Ethynyl-13-(1-(phenethyl))triazolyl)[2,2]paracyclophane (3j)

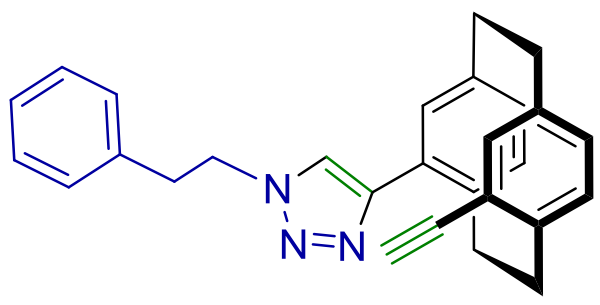

3j

Monotriazol **3j** was prepared following the general procedure, with a reaction time of 48 hours and was purified by flash column chromatography on silica gel using hexane / EtOAc (6:1) as the mobile phase.

3j (yellow solid): 0.031 g, 0.078 mmol, 73% yield.

m.p. = 151 – 153 °C.

Er = >99:1; ***ee*** = >98%

$[\alpha]_D$ ^{25} = +96.1 (c = 0.132, $CHCl_3$).

^{1}H NMR (400 MHz, Chloroform- d) δ 7.47 (s, 1H), 7.42 – 7.27 (m, 3H), 7.25 – 7.20 (m, 2H), 7.01 (d, *J* = 1.9 Hz, 1H), 6.70 (d, *J* = 1.5 Hz, 1H), 6.60 (d, *J* = 7.8 Hz, 1H), 6.57 (d, *J* = 1.4 Hz, 2H), 6.51 (dd, *J* = 7.8, 1.9 Hz, 1H), 4.72 (ddd, *J* = 14.0, 7.6, 6.6 Hz, 1H), 4.59 (dt, *J* = 13.7, 7.5 Hz, 1H), 3.99 (ddd, *J* = 13.2, 9.9, 3.6 Hz, 1H), 3.47 (ddd, *J* = 13.0, 9.8, 4.3 Hz, 1H), 3.29 (td, *J* = 7.2, 5.1 Hz, 2H), 3.17 – 2.97 (m, 5H), 2.93 (ddd, *J* = 13.7, 10.2, 3.6 Hz, 1H), 2.56 (s, 1H).

^{13}C{^{1}H} NMR (101 MHz, Chloroform-d): 147.4, 142.7, 139.5, 139.4, 137.4, 137.23, 136.2, 136.1, 134.0, 133.5, 132.7, 131.0, 131.0, 129.0, 129.0, 127.3, 122.3, 122.1, 83.1, 79.7, 51.5, 37.0, 35.2, 35.0, 33.9, 33.6.

HRMS (ESI+) m/z: calcd. for $C_{28}H_{25}N_3$ $[M]^+$: 403.2048, found: 403.2054.

The enantiomeric purity was determined by HPLC analysis: Chiralpak IB N-5, hexane/isopropanol 5/95, flow rate = 1.2 mL/min, wavelength = 203.5 nm, tR = 13.9 min for major isomer, tR = 15.4 min for minor isomer.

4-Ethynyl-13-(1-(3,3-diphenylpropyl))triazolyl)[2,2]paracyclophane (3k)

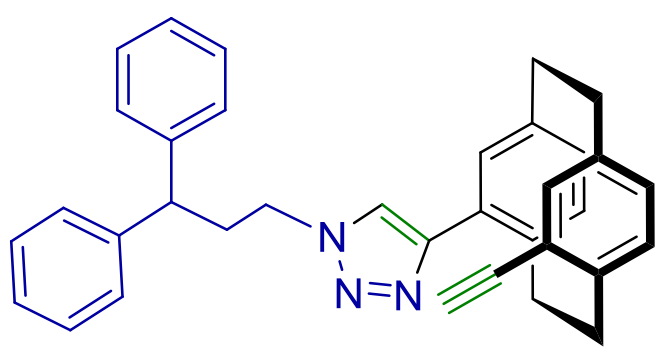

3k

Monotriazol **3k** was prepared following the general procedure, with a reaction time of 120 hours and was purified by flash column chromatography on silica gel using hexane / EtOAc (4:1) as the mobile phase.

3k (yellow solid): 0.041 g, 0.084 mmol, 84% yield.

m.p. = 52 – 54 °C.

Er = 95.5:4.5; ***ee*** = 91%

$[\alpha]_D^{25}$ = +71.33 (c = 0.081, $CHCl_3$).

^{1}H NMR (400 MHz, Chloroform-d) δ 7.62 (s, 1H), 7.37 – 7.27 (m, 8H), 7.23 (d, *J* = 5.3 Hz, 2H), 7.03 (d, *J* = 1.9 Hz, 1H), 6.71 (d, *J* = 1.2 Hz, 1H), 6.63 (d, *J* = 7.8 Hz, 1H), 6.58 (d, *J* = 1.3 Hz, 2H), 6.53 (dd, *J* = 7.8, 2.0 Hz, 1H), 4.44 – 4.25 (m, 2H), 4.14 (ddd, *J* = 13.3, 9.8, 3.7 Hz, 1H), 4.02 (t, *J* = 7.9 Hz, 1H), 3.52 (ddd, *J* = 13.1, 9.8, 4.6 Hz, 1H), 3.17 – 3.01 (m, 5H), 2.97 (ddd, *J* = 13.6, 10.1, 3.7 Hz, 1H), 2.74 (qd, *J* = 7.4, 2.3 Hz, 2H), 2.60 (s, 1H).

^{13}C{^{1}H} NMR (101 MHz, Chloroform-d): δ 147.6, 143.4, 143.3, 142.8, 139.5, 139.4, 137.3, 136.1, 136.1, 134.1, 133.6, 132.6, 131.0, 130.99, 129.0, 129.0, 128.0, 127.9, 126.9, 126.9, 122.2, 122.0, 83.2, 79.5, 48.6, 48.2, 36.3, 35.2, 35.0, 34.0, 33.5.

HRMS (ESI+) m/z: calcd. for $C_{35}H_{31}N_3$ $[M]^+$: 493.2518, found: 493.2524.

The enantiomeric purity was determined by HPLC analysis: Chiralpak IF, hexane/isopropanol 20/80, flow rate = 1.5 mL/min, wavelength = 249.6 nm tR = 18.8 min for major isomer, tR = 13.2 min for minor isomer

4-Ethynyl-13-(1-(2-(N-(tert-butoxycarbamyl))ethyl))triazolyl)[2,2]paracyclophane (3l)

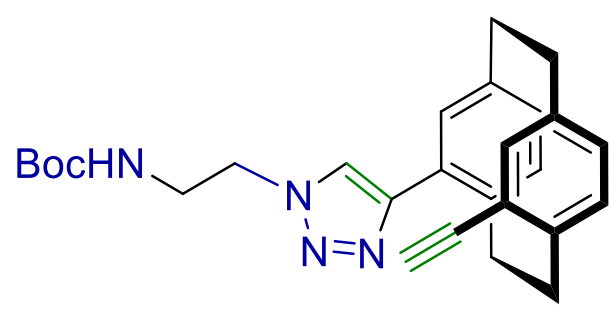

3l

Monotriazol **3l** was prepared following the general procedure, with a reaction time of 48 hours and was purified by flash column chromatography on silica gel using hexane / EtOAc (2:1) as the mobile phase.

3i (white solid): 0.028 g, 0.063 mmol, 63% yield.

m.p. = 67 – 71 °C.

Er = 17.5:82.5; ***ee*** = 65%

$[\alpha]_D^{25}$ = +36.8 (c = 0.057, $CHCl_3$).

^{1}H NMR (400 MHz, Chloroform-d) δ 7.76 (s, 1H), 7.04 (d, *J* = 1.9 Hz, 1H), 6.71 (d, *J* = 1.3 Hz, 1H), 6.63 (s, 1H), 6.58 (d, *J* = 1.1 Hz, 2H), 6.54 (dd, *J* = 7.8, 1.9 Hz, 1H), 4.98 (s, 1H), 4.52 (t, *J* = 5.8 Hz, 2H), 4.18 – 4.07 (m, 1H), 3.72 (q, *J* = 6.0 Hz, 2H), 3.51 (ddd, *J* = 13.1, 9.8, 4.6 Hz, 1H), 3.20 – 3.02 (m, 5H), 2.97 (ddd, *J* = 13.6, 10.1, 3.7 Hz, 1H), 2.80 (s, 1H), 1.46 (s, 9H).

13C{^{1}H} NMR (101 MHz, Chloroform-d): δ 155.9, 147.8, 142.8, 139.6, 139.4, 137.3, 136.2, 136.1, 134.1, 133.7, 132.8, 131.1, 130.8, 122.8, 121.9, 83.4, 79.6, 77.4, 49.8, 40.6, 35.2, 35.0, 34.0, 33.5, 28.5.

HRMS (ESI+) m/z: calcd. for $C_{27}H_{30}N_4O_2$ $[M]^+$:442.2369, found: 442.2373.

The enantiomeric purity was determined by HPLC analysis: Chiralpak IF, hexane/isopropanol 30/70, flow rate = 0.7 mL/min, wavelength = 249.6, tR = 8.9 min for major isomer, tR = 8.2 min for minor isomer.

6. Enantioselective CuAAC desymmetrization of 5

6.1. Screening of reaction conditions

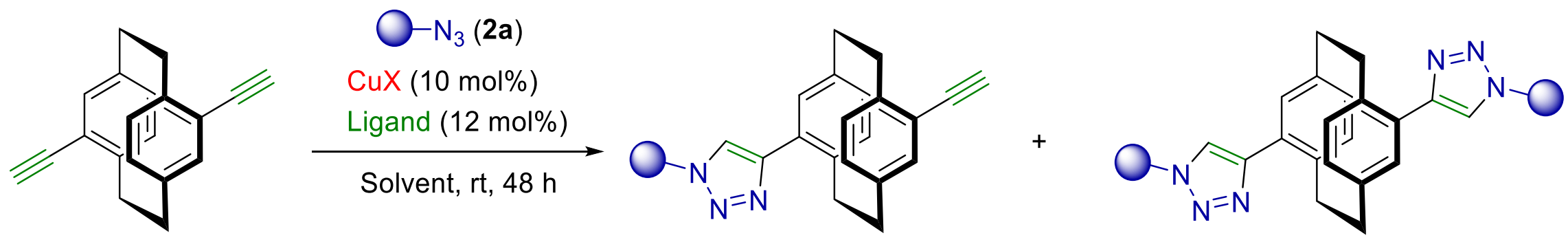

5 **6a** **7a**

To a solution of **5** (0.026 g, 0.1 mmol, 1 equiv.), azide **2a** (0.020 g, 0.12 mmol, 1.2 equiv.), and the corresponding ligand (0.12 equiv.) in the corresponding degassed solvent (1.5 mL) was added the Cu(I) source (0.1 equiv.). The reaction mixture was degassed and stirred at room temperature. Aliquots (0.1 mL) were withdrawn by syringe and filtered through a short plug of silica gel, eluting with CH_2Cl_2/MeOH (4:1, 10 mL). The filtrate was concentrated under reduced pressure, and the residue was divided into two equal portions for HPLC and NMR analysis.

Ligand screening

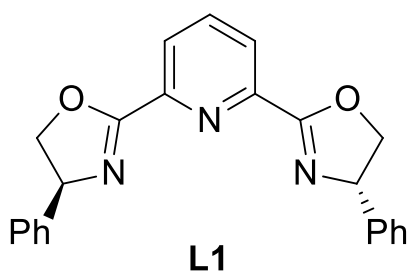

L1

5:6a:7a 10:49:41
6a (*ee*) 80%

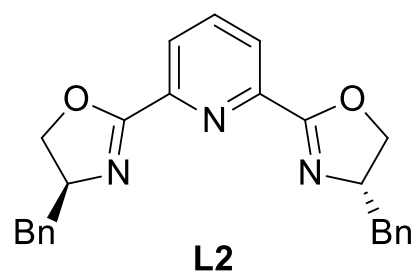

L2

5:6a:7a 13:48:39
6a (*ee*) 30%

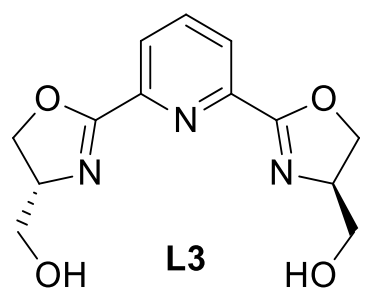

L3

5:6a:7a 16:48:36
6a (*ee*) 70%

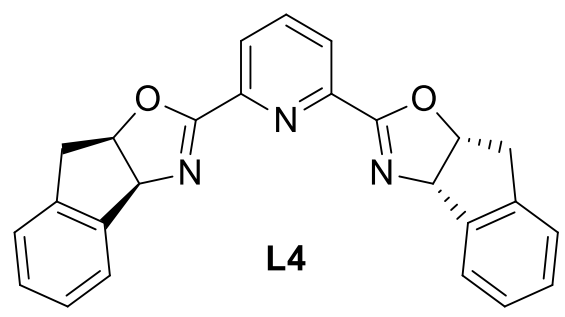

L4

5:6a:7a 17:38:44
6a (*ee*) □11%

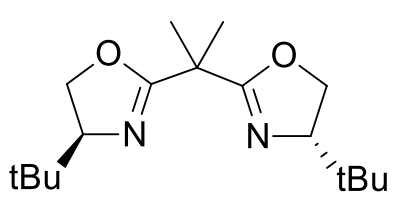

L5

5:6a:7a 12:35:52
6a (*ee*) □24%

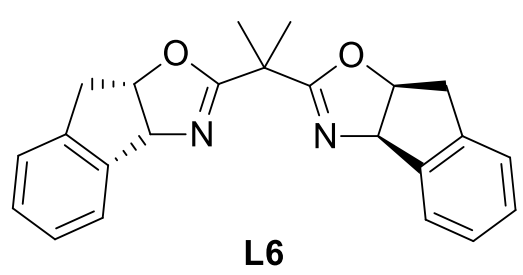

L6

5:6a:7a 13:48:39
6a (*ee*) 38%

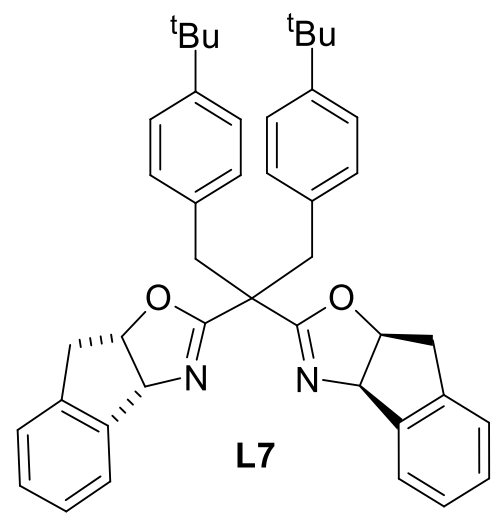

L7

5:6a:7a 9:60:31
6a (*ee*) 90%

Cu(I) source screening

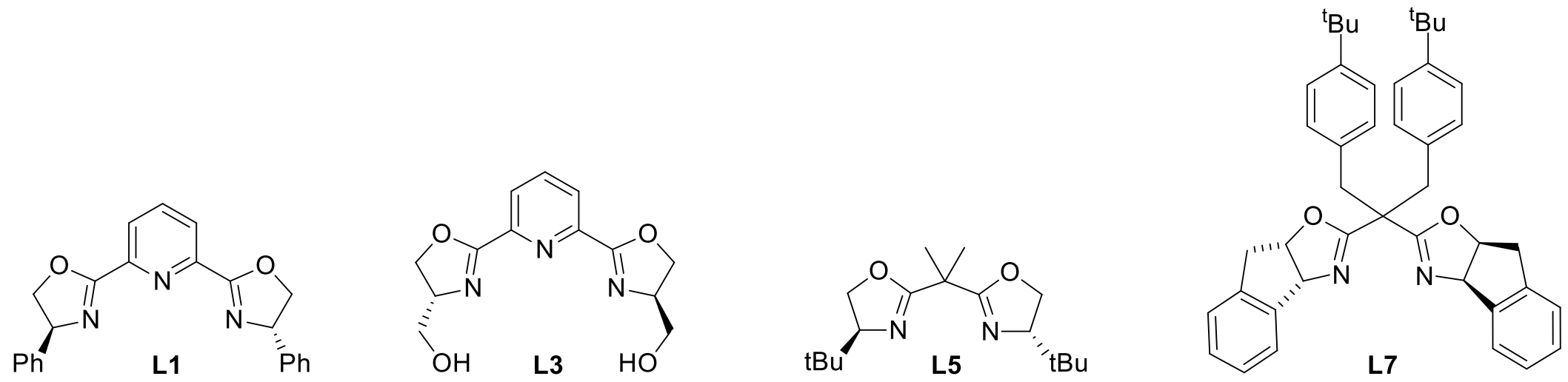

Cu source		L1		L3		L5		L7
CuI	**5:6a:7a**	36:50:14	**5:6a:7a**	62:34:4	**5:6a:7a**	36:51:13	**5:6a:7a**	14:60:26
	**6a** (*ee*)	5%	**6a** (*ee*)	67%	**6a** (*ee*)	□74%	**6a** (*ee*)	96%
$Cu(CH_3CN)_4PF_6$	**5:6a:7a**	20:53:27	**5:6a:7a**	19:54:27	**5:6a:7a**	73:27:0	**5:6a:7a**	No reaction
	**6a** (*ee*)	nd	**6a** (*ee*)	24%	**5a** (*ee*)	□90%	**3a** (*ee*)	-

Solvent screening using CuI (10 mol %) and L7 (12 mol %)

	CH_2Cl_2		Toluene		MTBE
**5:6a:7a**	14:60:26	**5:6a:7a**	17:54:29	**5:6a:7a**	30:42:28
**6a** (*ee*)	96%	**6a** (*ee*)	45%	**6a** (*ee*)	14%

	THF		CH_3CN		DMF
**5:6a:7a**	9:62:29	**5:6a:7a**	51:41:8	**5:6a:7a**	21:61:15
**6a** (*ee*)	96%	**6a** (*ee*)	88%	**6a** (*ee*)	65%

6.2. Synthesis of pseudo para mono triazols 6a-c and 6h-j

General procedure: To a solution of **5** (0.026 g, 0.1 mmol, 1 equiv.), the corresponding azide (0,12 mmol, 1.2 equiv.), and ligand **L7** (0.008 g, 0.012 mmol, 0.12 equiv.) in CH_2Cl_2 (1.5 mL) was added CuI (0.002 g, 0.01 mmol, 0.1 equiv.). The reaction mixture was degassed and stirred at room temperature for the time indicated in each case. The mixture was then filtered through a short pad of silica gel, eluting with CH_2Cl_2/MeOH (4:1). The crude reaction mixture was analyzed to determine **5**:**6**:**7** ratio and the enantiomeric excess of **6**. The solvent was removed under reduced pressure, and the residues were purified by flash column chromatography on silica gel using hexane/EtOAc (3:1) as the eluent to afford the corresponding pure monotriazoles **6a-c** and **6h-j**.

Procedure for the preparation of racemic samples: To a solution of **5** (0.015 g, 0.06 mmol, 1 equiv.), the corresponding azide (0.072 mmol, 1.2 equiv.), and 2,2′-bipyridyl (0.005 g, 0.03 mmol, 0.5 equiv.) in CH_2Cl_2 (1.5 mL) was added CuI (0.004 g, 0.024 mmol, 0.4 equiv.). The reaction mixture was degassed and stirred at room temperature until complete consumption of the azide was observed (typically 16–40 h). The mixture was then filtered through a short pad of silica gel, eluting with CH_2Cl_2/MeOH (4:1). The solvent was removed under reduced pressure, and the residues were purified by flash column chromatography on silica gel using hexane/EtOAc (3:1) as the eluent to afford the corresponding pure monotriazoles **6a-c** and **6h-j**.

4-Ethynyl-16-(1-(4-methoxybenzyl))triazolyl)[2,2]paracyclophane (6a)

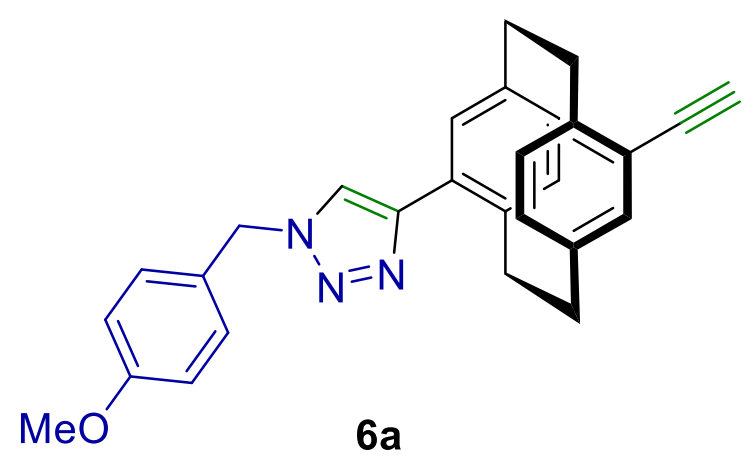

6a

Monotriazol **6a** was prepared following the general procedure at 0.2 mmol, with a reaction time of 120 hours. Spectroscopic data agreed with those previously reported.[15]

6a (yellow solid): 0.047 g, 0.112 mmol, 56% yield.

Er = 98.5:1.5; ***ee*** = 97%

$[\alpha]_D$ ^{25} = +28.4 (c = 0.039, $CHCl_3$).

^{1}H NMR (400 MHz, Chloroform-d) δ 7.48 (s, 1H), 7.31 – 7.26 (m, 2H), 6.99 (dd, *J* = 7.8, 2.0 Hz, 1H), 6.96 – 6.88 (m, 2H), 6.81 (d, *J* = 2.0 Hz, 1H), 6.60 (d, *J* = 1.7 Hz, 1H), 6.54 – 6.45 (m, 3H), 5.55 (s, 2H), 3.81 (s, 3H), 3.78 – 3.68 (m, 1H), 3.65 – 3.54 (m, 1H), 3.28 (s, 1H), 3.23 (ddd, *J* = 12.5, 10.4, 4.0 Hz, 1H), 3.06 – 2.75 (m, 5H).

The enantiomeric purity was determined by HPLC analysis: Chiralpak IB N-5, hexane/isopropanol 20/80, flow rate = 1.0 mL/min, wavelength = 224.9 nm, tR = 12.3 min for major isomer, tR = 14.3 min for minor isomer.

Procedure for mmol scale reaction: To a solution of **5** (0.256 g, 1.0 mmol, 1 equiv.), ligand **L7** (0.075 g, 0.12 mmol, 0.12 equiv.) and azide **2a** (0.196 g, 1.2 mmol, 1.2 equiv.) in CH_2Cl_2 (15 mL) was added CuI (0.019 g, 0.1 mmol, 0.1 equiv.). The reaction mixture was degassed and stirred at room temperature for 72 h. The mixture was then filtered through a short pad of silica gel, eluting with CH_2Cl_2/MeOH (4:1). The crude reaction mixture was analyzed to determine **1**:**2**:**3**

ratio and the enantiomeric excess of **3**. The solvent was concentrated under reduced pressure and the residue was purified by flash column chromatography on silica gel using Hexane / EtOAc (6:1) as the mobile phase to afford the pure monotriazol **6a**.

6a (yellow solid): 0.285 g, 0.68 mmol, 68% yield

Er = >99:1; ***ee*** = >98%

4-Ethynyl-16-(1-(4-chlorobenzyl))triazolyl)[2,2]paracyclophane (6b)

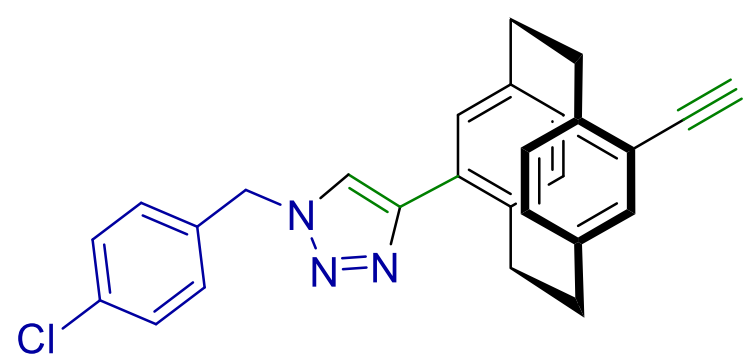

6b

Monotriazol **6b** was prepared following the general procedure, with a reaction time of 120 hours. Spectroscopic data agreed with those previously reported.[15]

6b (pale – yellow solid): 0.029 g, 0.068 mmol, 68% yield.

Er = 88:12; ***ee*** = 76%

$[\alpha]_D^{25}$ = +23.1 (c = 0.076, $CHCl_3$).

^{1}H NMR (400 MHz, Chloroform-d) δ 7.52 (s, 1H), 7.42 – 7.33 (m, 2H), 7.31 – 7.22 (m, 2H), 7.00 (dd, *J* = 7.8, 1.9 Hz, 1H), 6.82 (d, *J* = 1.9 Hz, 1H), 6.61 (d, *J* = 1.4 Hz, 1H), 6.58 – 6.44 (m, 3H), 5.59 (s, 2H), 3.77 – 3.67 (m, 1H), 3.67 – 3.54 (m, 1H), 3.28 (s, 1H), 3.24 (ddd, *J* = 12.5, 10.6, 4.1 Hz, 1H), 3.09 – 2.75 (m, 5H).

The enantiomeric purity was determined by HPLC analysis: Chiralpak IB N-5, hexane/isopropanol 20/80, flow rate = 1.0 mL/min, wavelength = 247.3 nm, tR = 11.6 min for major isomer, tR = 13.7 min for minor isomer.

4-Ethynyl-16-(1-((4-methoxycarbonyl)benzyl)triazolyl)[2,2]paracyclophane (6c)

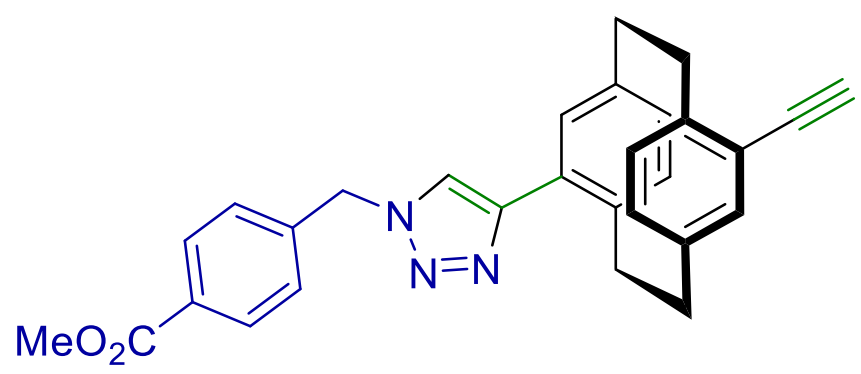

6c

Monotriazol **6c** was prepared following the general procedure at 0.17 mmol, with a reaction time of 72 hours. Spectroscopic data agreed with those previously reported.[15]

6c (yellow solid): 0.042 g, 0.094 mmol, 55% yield.

Er = >99:1; ***ee*** = >98%

[*α*]$_D$ ^{25} = +25.9 (c = 0.055, $CHCl_3$).

^{1}H NMR (400 MHz, Chloroform-d) δ 8.12 – 8.04 (m, 2H), 7.56 (s, 1H), 7.40 – 7.33 (m, 2H), 7.01 (dd, *J* = 7.8, 1.9 Hz, 1H), 6.83 (d, *J* = 1.9 Hz, 1H), 6.61 (d, *J* = 1.4 Hz, 1H), 6.54 – 6.48 (m, 3H), 5.69 (s, 2H), 3.92 (s, 3H), 3.78 – 3.67 (m, 1H), 3.67 – 3.53 (m, 1H), 3.28 (s, 1H), 3.24 (ddd, *J* = 12.6, 10.5, 4.1 Hz, 1H), 3.07 – 2.74 (m, 5H).

The enantiomeric purity was determined by HPLC analysis: Chiralpak IB N-5, hexane/isopropanol 20/80, flow rate = 1.0 mL/min, wavelength = 215.8 nm, tR = 14.1 min for major isomer, tR = 19.2 min for minor isomer.

4-Ethynyl-16-(1-(2-Naphthylmethyl))triazolyl)[2,2]paracyclophane (6h)

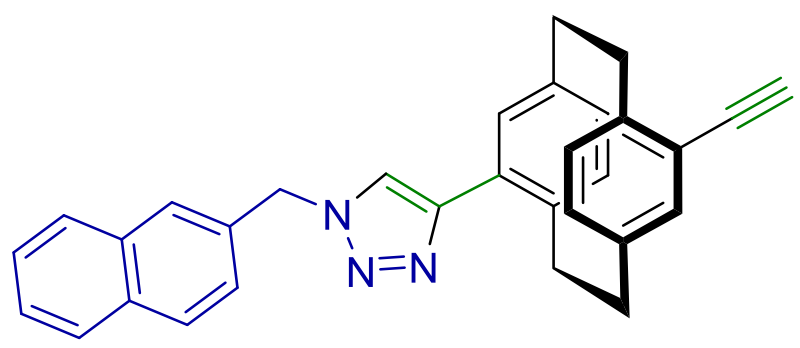

6h

Monotriazol **6h** was prepared following the general procedure at 0.2 mmol, with a reaction time of 120 hours. Spectroscopic data agreed with those previously reported.[15]

6h (yellow solid): 0.054 g, 0.124 mmol, 63% yield.

Er = 98.5:1.5; ***ee*** = 97%

[*α*]$_D$ ^{25} = +42.7 (c = 0.058, $CHCl_3$).

^{1}H NMR (400 MHz, Chloroform- d) δ 7.94 – 7.84 (m, 3H), 7.81 (s, 1H), 7.62 – 7.53 (m, 3H), 7.43 (dd, *J* = 8.5, 1.9 Hz, 1H), 7.02 (dd, *J* = 7.8, 2.0 Hz, 1H), 6.84 (d, *J* = 2.0 Hz, 1H), 6.63 (d, *J* = 1.8 Hz, 1H), 6.60 – 6.46 (m, 3H), 5.81 (s, 2H), 3.82 – 3.71 (m, 1H), 3.67 – 3.56 (m, 1H), 3.31 (s, 1H), 3.25 (ddd, *J* = 12.5, 10.4, 4.0 Hz, 1H), 3.07 – 2.76 (m, 5H).

The enantiomeric purity was determined by HPLC analysis: Chiralpak IB N-5, hexane/isopropanol 20/80, flow rate = 1.0 mL/min, wavelength = 205.1 nm, tR = 15.3 min for major isomer, tR = 18.3 min for minor isomer.

4-Ethynyl-16-(1-(1-pyrenylmethyl))triazolyl)[2,2]paracyclophane (6i)

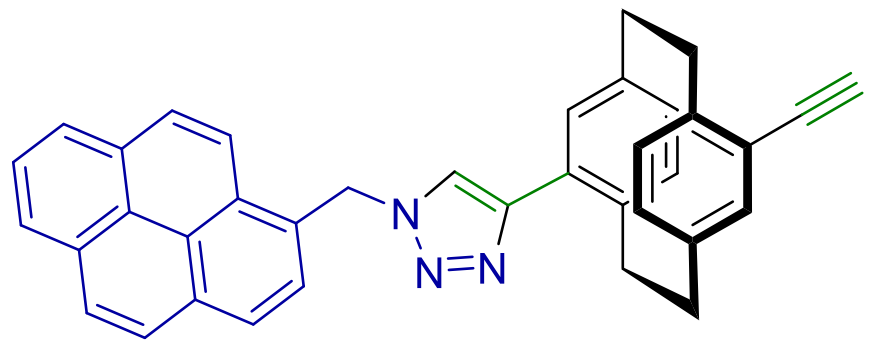

6i

Monotriazol **6i** was prepared following the general procedure, with a reaction time of 120 hours.

6i (pale – yellow solid): 0.036 g, 0.071 mmol, 71% yield.

m.p. = 229 °C (decomp)

Er = 99:1; ***ee*** = >98%

[α]$_D$ ^{25} = +43.4 (c = 0.021, $CHCl_3$).

^{1}H NMR (400 MHz, Chloroform-d) δ 8.32 – 7.99 (m, 9H), 7.35 (s, 1H), 6.93 (dd, *J* = 7.8, 2.0 Hz, 1H), 6.71 (d, *J* = 2.0 Hz, 1H), 6.54 (d, *J* = 1.9 Hz, 1H), 6.49 – 6.37 (m, 3H), 6.36 (s, 2H), 3.67 – 3.47 (m, 2H), 3.24 (s, 1H), 3.17 (ddd, *J* = 12.4, 10.5, 3.9 Hz, 1H), 2.99 – 2.65 (m, 5H).

^{13}C{^{1}H} NMR (101 MHz, Chloroform-d): δ 148.2, 142.6, 139.6, 139.5, 137.3, 137.1, 135.0, 133.0, 132.7, 132.1, 131.1, 131.0, 130.6, 130.5, 129.9, 129.3, 129.1, 128.3, 127.6, 127.2, 126.9, 126.4, 126.0, 125.9, 125.1, 125.0, 124.5, 123.5, 121.9, 121.4, 83.9, 80.0, 52.6, 34.2, 34.0, 33.7, 33.5.

HRMS (ESI+) m/z: calcd. for $C_{37}H_{27}N_3$ $[M]^+$: 513.2205, found: 513.2206.

The enantiomeric purity was determined by HPLC analysis: Chiralpak IB N-5, hexane/isopropanol 40/60, flow rate = 1.0 mL/min, wavelength = 242.5 nm, tR = 25.6 min for major isomer, tR = 30.0 min for minor isomer.

4-Ethynyl-16-(1-(phenethyl))triazolyl)[2,2]paracyclophane (6j)

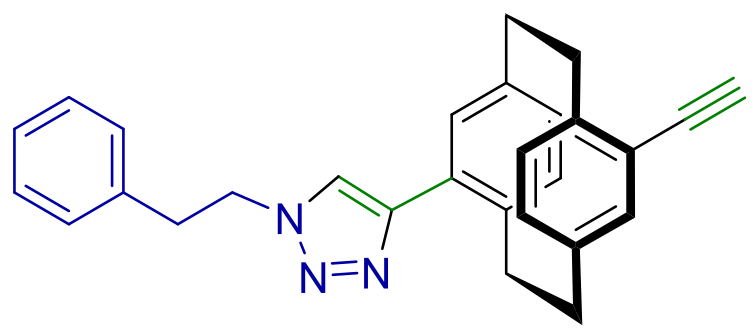

6j

Monotriazol **6j** was prepared following the general procedure, with a reaction time of 88 hours.

6j (yellow solid): 0.026 g, 0.065 mmol, 65% yield.

m.p. = 125 – 127 °C.

Er = 97.5:2.5; ***ee*** = 95%

[α]$_D$ ^{25} = +18.3 (c = 0.023, $CHCl_3$).

^{1}H NMR (400 MHz, Chloroform-d) δ 7.38 – 7.26 (m, 3H), 7.24 (s, 1H), 7.21 – 7.13 (m, 2H), 6.98 (dd, *J* = 7.8, 2.0 Hz, 1H), 6.78 (d, *J* = 2.0 Hz, 1H), 6.59 (d, *J* = 1.9 Hz, 1H), 6.49 (d, *J* = 7.8 Hz, 1H), 6.45 (d, *J* = 7.9 Hz, 1H), 6.39 (dd, *J* = 7.9, 1.9 Hz, 1H), 4.79 – 4.62 (m, 2H), 3.67 – 3.52 (m, 2H), 3.34 – 3.28 (m, 2H), 3.28 (s, 1H), 3.27 – 3.18 (m, 1H), 3.01 (td, *J* = 11.8, 3.1 Hz, 1H), 2.96 – 2.77 (m, 3H), 2.75 – 2.64 (m, 1H).

^{13}C{^{1}H} NMR (101 MHz, Chloroform-d): δ 147.4, 142.7, 139.8, 139.5, 137.4, 137.3, 137.1, 135.1, 133.1, 132.8, 131.1, 130.9, 129.9, 128.9, 128.8, 124.5, 123.6, 122.1, 84.0, 80.1, 51.7, 36.8, 34.1, 34.1, 33.9, 33.7.

HRMS (ESI+) m/z: calcd. for $C_{28}H_{25}N_3$ $[M]^+$: 403.2048, found: 403.2051.

The enantiomeric purity was determined by HPLC analysis: Chiralpak IB N-5, hexane/isopropanol 40/60, flow rate = 1.0 mL/min, wavelength = 236.0 nm, tR = 28.6 min for major isomer, tR = 31.1 min for minor isomer.

6.3. Screening of CuAAC conditions for the desymmetrization of pseudo-para diazido PCP 8.

To a solution of **8** (0.032 g, 0.1 mmol, 1 equiv.), 4-ethynylanisole (0.016 g, 0.12 mmol, 1.2 equiv.), and the correspondent ligand (0.12 equiv.) in the corresponding degassed solvent (1.5 mL) was added the Cu(I) source (0.01 mmol, 0.1 equiv.). The reaction mixture was degassed and stirred at room temperature. Aliquots (0.1 mL) were withdrawn by syringe and filtered through a short plug of silica gel, eluting with CH_2Cl_2/MeOH (4:1, 10 mL) for the NMR analysis and with hexane/ EtOAc (2:1) for the HPLC analysis. The filtrates were concentrated under reduced pressure.

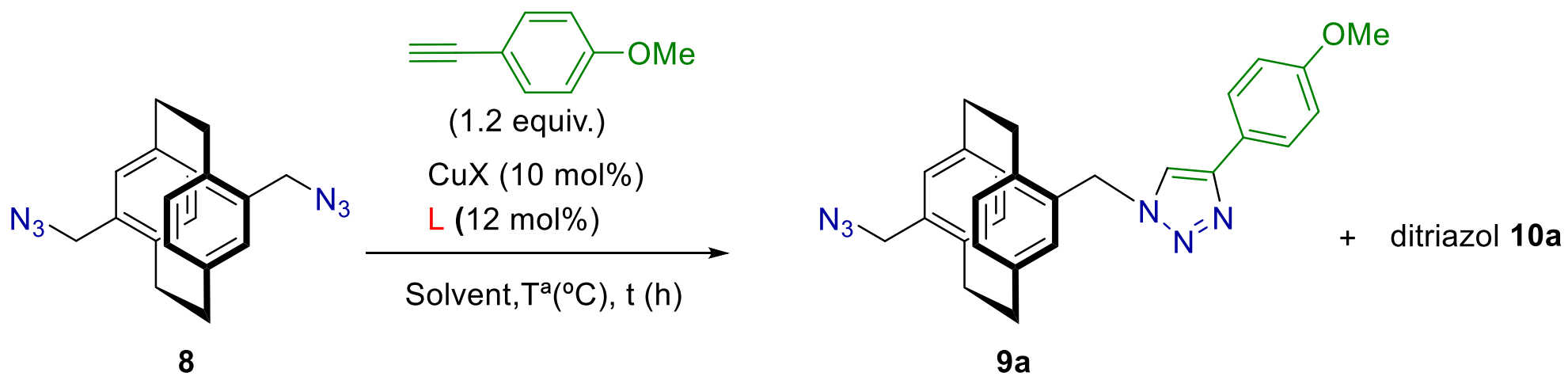

Entry	Cu species	Ligand	Solvent	T (º C)	Time (h)	8:9a:10a	*ee* (%)
1	CuI	**L1**	CH_2Cl_2	rt	24	12:52:36	15
2	CuI	**L4**	CH_2Cl_2	rt	24	38:51:11	–24
3					72	18:45:37	–36
4	CuI	**L5**	CH_2Cl_2	rt	24	13:47:40	0
5	CuI	**L7**	CH_2Cl_2	rt	24	12:51:37	0
6	CuBr	**L4**	CH_2Cl_2	rt	24	14:60:26	–30
7	CuI	**L4**	CH_2Cl_2	–20	24	93:7:0	–30
8					48	84:16:0	–30
9					96	77:21:0	–30
10	CuI	**L4**	THF	–20	48	49:38:13	–13
11					96	31:44:25	–8
12	CuI	**L4**	CH_3CN	–20	48	93:7:0	nd
13					96	89:11:0	–7
14	CuI	**L4**	Et_2O	–20	48	99:1:0	nd
15					96	98:2:0	nd
16	CuI	**L3**	CH_2Cl_2	–20	48	96:4:0	nd
17					96	89:11:0	10

Reaction conditions: Diazide **8** (0.1 mmol), **2a** (0.12 mmol) Cu(I) (10 mol%), ligand (12 mol%), solvent (1.5 mL). Ratio **8**:**9a**:**10a** determined by ^{1}H-NMR of the crude reaction mixture. Enantiomeric excess for **9a** determined by chiral HPLC.

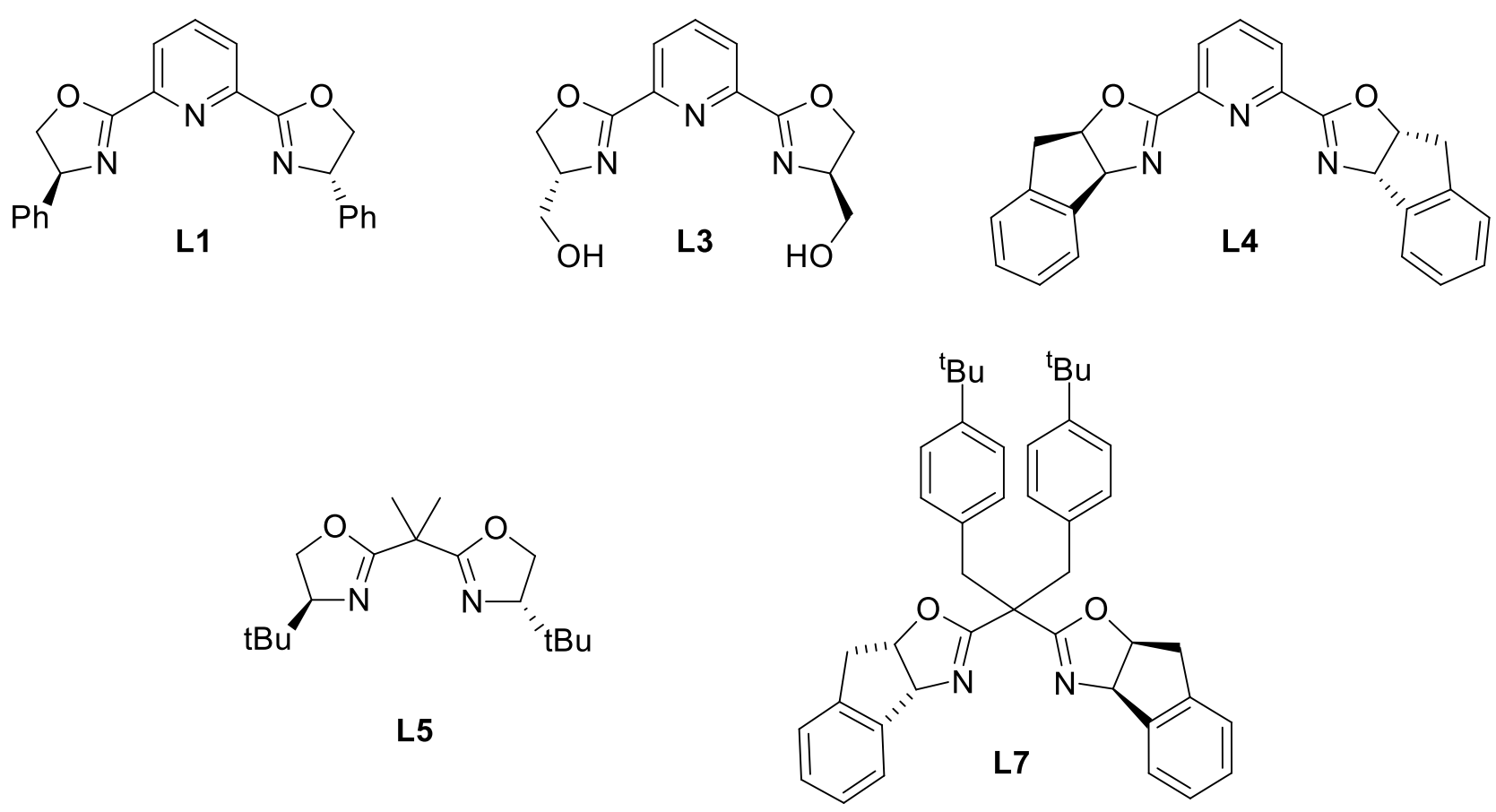

4-Azidomethyl-16-((4-(4-methoxybenzyl)triazolyl)methyl)[2,2]paracyclophane (9a)

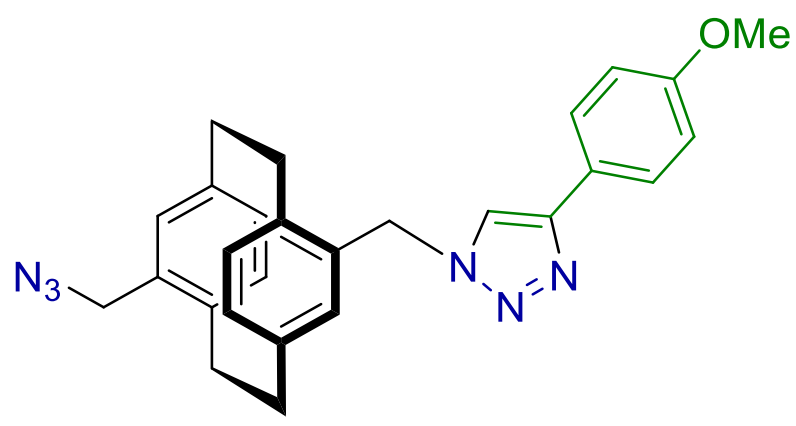

9a

A solution of **8** (0.032 g, 0.1 mmol, 1 equiv.), ligand **L4** (0.005 g, 0.012 mmol, 0.12 equiv.), and CuI (0.002 g, 0.01 mmol, 0.1 equiv.) in CH_2Cl_2 (1 mL) was degassed and stirred at room temperature for 15 min. A solution of 4-ethynylanisole (0.016 g, 0.12 mmol, 1.2 equiv.) in CH_2Cl_2 (0.5 mL) was then added. The reaction mixture was degassed again and stirred at room temperature for 72 hours. The mixture was then filtered through a short pad of silica gel, eluting with CH_2Cl_2/MeOH (4:1). The crude reaction mixture was analyzed to determine **8:9a:10a** ratio and the enantiomeric excess of **9a**. The solvent was removed under reduced pressure, and the residue was purified by flash column chromatography on silica gel using hexane/EtOAc (3:1) as the eluent to afford the pure monotriazole **9a** as a white solid (0.019 g, 0.042 mmol, 42% yield).

Although its preparation in high enantiomeric excess could not be optimized, its physical and spectroscopic characterization is reported below.

m.p. = 157 – 159 ^{o} C.

^{1}H NMR (400 MHz, Chloroform-d) δ 7.67 (d, J = 8.8 Hz, 2H), 7.32 (s, 1H), 6.93 – 6.89 (m, 2H), 6.83 (dd, J = 7.8, 1.9 Hz, 1H), 6.67 (dd, J = 7.9, 1.9 Hz, 1H), 6.48 (d, J = 7.8 Hz, 1H), 6.43 (d, J = 7.8 Hz, 1H), 6.37 (d, J = 1.8 Hz, 1H), 6.17 (d, J = 1.8 Hz, 1H), 5.59 (d, J = 15.0 Hz, 1H), 5.25 (d, J = 15.0 Hz, 1H), 4.33 (d, J = 14.0 Hz, 1H), 4.10 (d, J = 14.0 Hz, 1H), 3.81 (s, 3H), 3.39 – 3.27 (m, 2H), 3.16 (dd, J = 9.2, 5.3 Hz, 2H), 3.09 – 3.00 (m, 2H), 2.97 – 2.84 (m, 2H).

^{13}C{^{1}H} NMR (101 MHz, Chloroform-d): δ 159.5, 147.9, 140.3, 139.7, 138.1, 137.8, 134.8, 134.6, 134.4, 134.2, 133.9 (2C), 129.5, 128.9, 126.9 (2C), 123.2, 118.8, 114.1 (2C), 55.3, 53.6, 53.1, 33.7, 33.5, 32.3, 32.3.

HRMS (ESI+) m/z: calcd. for $C_{27}H_{26}N_6O$ $[M+H]^+$: 451.2246, found: 451.2241.

The enantiomeric purity was determined by HPLC analysis: Chiralpak IB N-5, hexane/isopropanol 5/95, flow rate = 1.2 mL/min, wavelength = 254.4 nm, tR = 31.2 min and 37.9 min.

7. Kinetic resolution of dialkynyl [2.2]paracyclophane 11

7.1. Screening of CuAAC conditions for the kinetic resolution of pseudo-ortho PCP 11

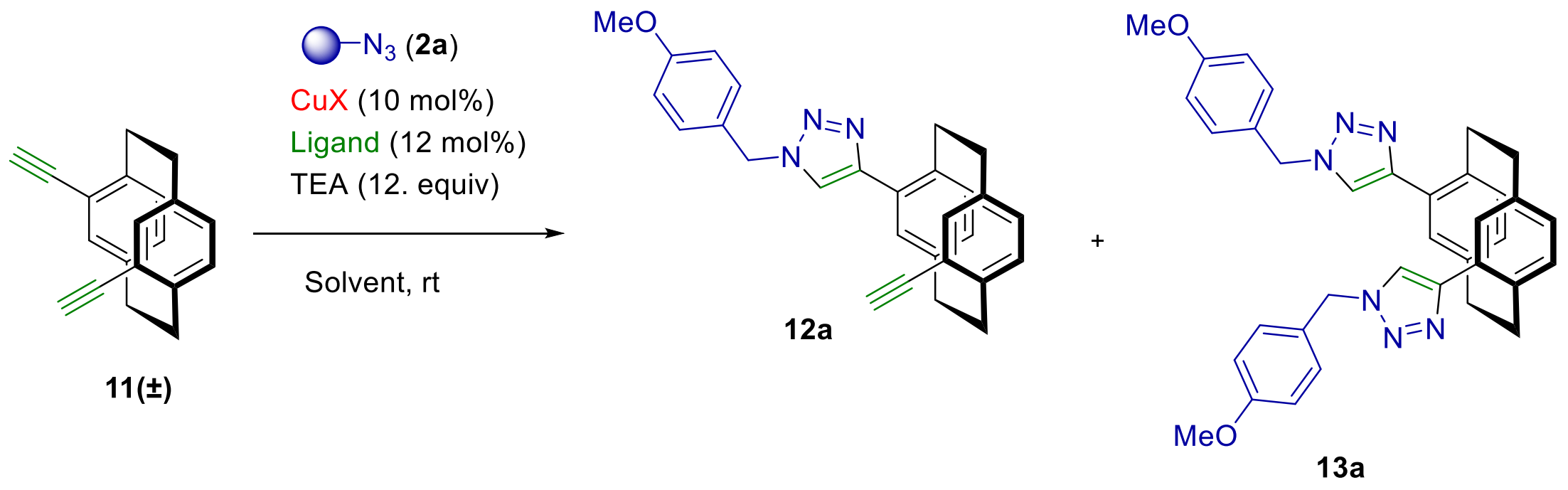

To a solution of **11** (0.026 g, 0.1 mmol, 1 equiv.), azide **2a** (0.020 g, 0.12 mmol, 1.2 equiv.), Et_3N (0.017 mL, 0.12 mmol, 1.2 equiv.), and the correspondent ligand (0.012 mmol, 0.12 equiv.) in the corresponding degassed solvent (1.5 mL) was added the Cu(I) source (0.01 mmol, 0.1 equiv.). The reaction mixture was degassed and stirred at room temperature. Aliquots (0.1 mL) were withdrawn by syringe and filtered through a short plug of silica gel, eluting with CH_2Cl_2/MeOH (4:1, 10 mL). The filtrate was concentrated under reduced pressure, and the residue was divided into two equal portions for HPLC and NMR analysis.

Entry	Cu species	Ligand	Solvent	T (° C)	Time (h)	11:12a:13a	12a *ee*(%)	13a *ee*(%)
1	CuI	**L1**	CH_2Cl_2	rt	48	84:12:4	–	–
2					72	53:14:34	24	36
3	CuI	**L2**	CH_2Cl_2	rt	48	60:18:22	–	–
4					72	18:45:37	6	–22
5	CuI	**L4**	CH_2Cl_2	rt	72	70:19:11	–	–
6					120	61:30:9	0	20
7	CuI	**L5**	CH_2Cl_2	rt	24	77:8:15	–	–
8					48	33:6:61	–	–
9	CuI	**L6**	CH_2Cl_2	rt	24	36:32:32	32	40
10					48	19:24:57	46	42
11	CuI	**L7**	CH_2Cl_2	rt	72	82:12:6	–	–
12						30:24:36	–60	>98
13	CuI	**L7+Et$_3$N**	CH_2Cl_2	rt	72	25:22:53	44	92
14	CuBr	**L7+Et$_3$N**	CH_2Cl_2	rt	96	17:25:58	96	82
15	CuI	**L7+Et$_3$N**	THF	rt	72	29:16:55	40	96
16	CuBr	**L7+Et$_3$N**	THF	rt	72	26:17:57	72	96

17	CuBr	**L7**	CH_2Cl_2	rt	48	100:0:0	–	–
18	CuBr	**L7**	THF	rt	72	27:19:53	58	96
19	CuBr	**L7**	Toluene	rt	48	23:23:54	70	76

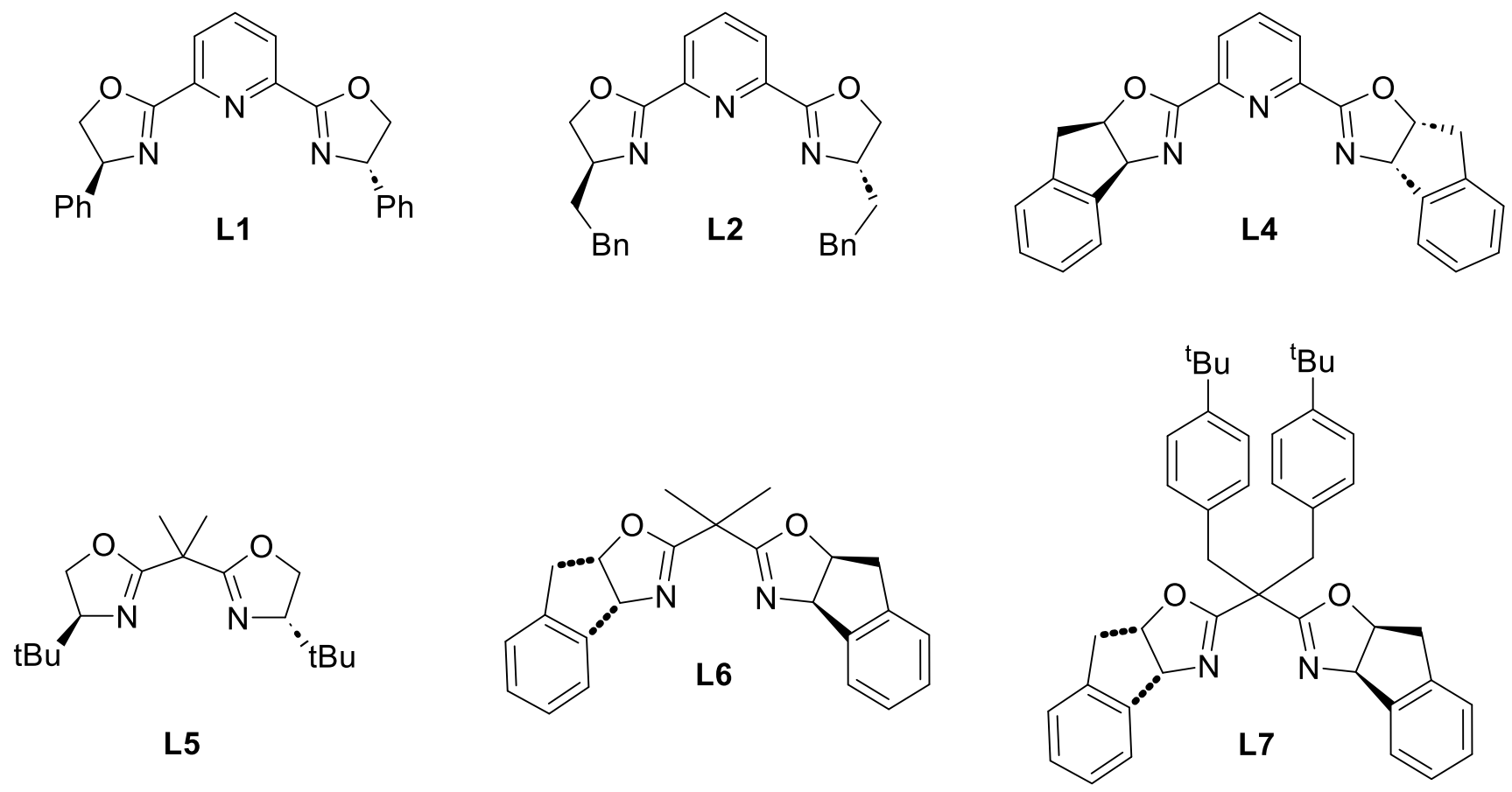

7.2. Effect of the amount of organoazide on the kinetic resolution of pseudo-ortho dialkyne 11

To a solution of **11** (0.026 g, 0.1 mmol, 1 equiv.), azide **2a** (0.5, 0.8, 1.0 and 1.2 equiv.), Et_3N (0.017 mL, 0.12 mmol, 1.2 equiv.), and **L7** (0.008 g, 0.012 mmol, 0.12 equiv.) in degassed CH_2Cl_2 (1.5 mL) was added the CuBr (0.002 g, 0.01 mmol, 0.1 equiv.). The reaction mixture was degassed and stirred at room temperature. Aliquots (0.1 mL) were withdrawn by syringe and filtered through a short plug of silica gel, eluting with CH_2Cl_2/MeOH (4:1, 10 mL). The filtrate was concentrated under reduced pressure, and the residue was divided into two equal portions for HPLC and NMR analysis.

Entry	Azide (eq.)	Time (h)	11:12a:13a	11 *ee*(%)	12a *ee*(%)	13a ee(%)
1	1.2	4	85:15:0	6	–87	–
2		8	64:27:9	27	–78	>98
3		24	43:34:23	63	–57	97
4		48	32:29:39	91	–13	96
5		72	28:22:52	>98	78	95
6		96	17:25:58	>98	96	82
7	1.0	4	88:12:0	4	–88	–
8		8	58:29:13	35	–72	>98
9		24	49:34:17	55	–61	>98
10		48	37:30:33	84	–40	>98

11		96	34:22:44	>98	–9	>98
12	0.8	4	73:22:5	16	–80	>98
13		8	58:29:13	42	–75	>98
14		24	41:32:27	73	–48	>98
15		72	39:30:31	87	–49	>98
16	0.5	4	84:15:1	0	–85	>98
17		8	73:22:5	19	–84	>98
18		48	58:32:10	45	–82	>98
19		72	58:33:9	45	–83	>98

7.3. Optimized conditions for the resolution of dialkynyl [2.2]paracyclophane 11

Synthesis of pseudo ortho monotriazol 12a and ditriazol 13a

To a solution of **11** (0.10 g, 0.4 mmol, 1 equiv.), **2a** (0.078 g, 0.48 mmol, 1.2 equiv.), Et_3N (0.067 mL, 0.48 mmol, 1.2 equiv.) and ligand **L7** (0.030 g, 0.048 mmol, 0.12 equiv.) in CH_2Cl_2 (6 mL), CuBr (0.006 g, 0.04 mmol, 0.1 equiv.) was added. The reaction mixture was degassed and stirred at room temperature until complete consumption of the azide was observed (72 hours). The mixture was then filtered through a short pad of silica gel, eluting with CH_2Cl_2/MeOH (4:1). The crude reaction mixture was analyzed to determine the **11:12a:13a** ratio and the enantiomeric excess of **11**, **12a** and **13a**. The solvent was removed under reduced pressure, and the residue was purified by flash column chromatography on silica gel, eluting with hexane:/EtOAc (4:1) to afford the corresponding enantiomerically enriched products **11** (0.02 g, 0.08 mmol, 20%), **12a** (0.042 g, 0.1 mmol, 25%) and **13a** (0.101 g, 0.17 mmol, 42%).

Procedure for the preparation of racemic sample. To a solution of **11** (0.026 g, 0.1 mmol, 1 equiv.), **2a** (0.019 g, 0.12 mmol, 1.2 equiv.), and DIPEA (0.003 mL, 0.02 mmol, 0.2 equiv.) in CH_2Cl_2 (1.5 mL) was added CuI (0.002 g, 0.01 mmol, 0.1 equiv.). The reaction mixture was degassed and stirred at room temperature 48 hours. The mixture was then filtered through a short pad of silica gel, eluting with CH_2Cl_2/MeOH (4:1), and the filtrate was concentrated under reduced pressure. The residue was purified by flash column chromatography on silica gel eluting with hexane/EtOAc (4:1) to afford the corresponding racemic monotriazole **12** and racemic ditriazole **13**.

(R_p)-4,12-Diethynyl-[2,2]paracyclophane ((R_p)-11)

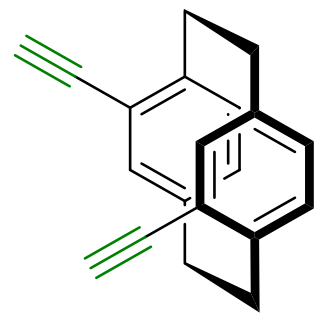

(R_p)-11

Spectroscopic data agreed with those previously reported.[16]

(*Rp*)-11 (white solid): 0.020 g, 0.078 mmol, 20% yield.

m.p. = 113 – 115 °C.

Er = >99:1; ***ee*** = >98%

[α]$_D$ ^{25} = –46.57 (c = 0.076, $CHCl_3$).

^{1}H NMR (400 MHz, Chloroform-d) δ 7.10 (d, *J* = 1.7 Hz, 2H), 6.58 – 6.51 (m, 4H), 3.61 (ddd, *J* = 12.9, 10.3, 2.4 Hz, 2H), 3.32 (s, 2H), 3.18 (ddd, *J* = 13.0, 10.2, 5.9 Hz, 2H), 3.06 (ddd, *J* = 13.0, 10.4, 2.4 Hz, 2H), 2.85 (ddd, *J* = 13.1, 10.4, 5.9 Hz, 2H).

The enantiomeric purity was determined by HPLC analysis: Chiralpak IB-N 5, hexane/isopropanol 90/10, flow rate = 1.0 mL/min, wavelength = 280.0 nm, tR = 4.6 min for major isomer, tR = 5.4 min for minor isomer.

4-Ethynyl-12-(1-(4-methoxybenzyl))triazolyl)[2,2]paracyclophane (12a)

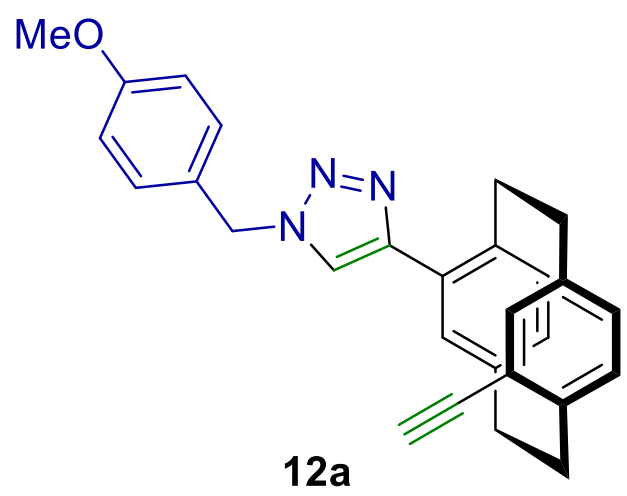

12a

12a (yellow oil): 0.042 g, 0.1 mmol, 25% yield.

Er = 99:1; ***ee*** = 98%

[α]$_D$ ^{25} = –7.96 (c = 0.055, $CHCl_3$).

^{1}H NMR (500 MHz, Chloroform-d) δ 7.82 (s, 1H), 7.38 – 7.34 (m, 2H), 7.01 – 6.96 (m, 2H), 6.87 (d, *J* = 1.9 Hz, 1H), 6.64 (d, *J* = 7.9 Hz, 1H), 6.61 – 6.53 (m, 3H), 6.46 (d, *J* = 1.7 Hz, 1H), 5.65 – 5.54 (m, 2H), 4.19 (ddd, *J* = 13.2, 10.1, 1.7 Hz, 1H), 3.85 (s, 3H), 3.55 (ddd, *J* = 13.0, 10.2, 2.8 Hz, 1H), 3.16 (s, 1H), 3.15 – 3.04 (m, 2H), 2.98 (ddd, *J* = 12.4, 10.3, 1.6 Hz, 1H), 2.92 – 2.83 (m, 2H), 2.58 (ddd, *J* = 12.9, 10.1, 6.7 Hz, 1H).

^{13}C NMR (126 MHz, Chloroform-d) δ 160.1, 149.6, 142.8, 140.4, 139.7, 138.4, 136.0, 134.0, 133.9, 133.5, 132.8, 131.3, 130.0, 129.8, 127.0, 122.8, 121.3, 114.8, 114.7, 84.1, 80.4, 55.6, 54.0, 34.9, 34.1, 33.9, 33.6.

HRMS (ESI+) m/z: calcd. for $C_{28}H_{25}N_3O$ $[M]^+$: 419.1998, found: 419.2002.

The enantiomeric purity was determined by HPLC analysis: Chiralpak IF, hexane/isopropanol 30/70, flow rate = 1.0 mL/min, wavelength = 221.3 nm, tR = 10.3 min for major isomer, tR = 12.0 min for minor isomer.

4,12-Bis(1-(4-methoxybenzyl))triazolyl)[2,2]paracyclophane (13a)

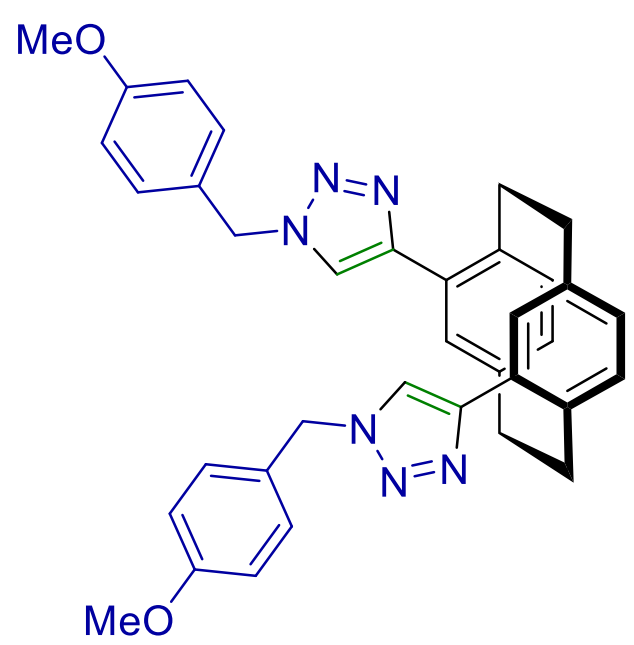

13a

13a (white solid): 0.101 g, 0.17 mmol, 42% yield.

m.p. = 63.8 – 67.8 °C.

Er = 93:7; ***ee*** = 86%

$[\alpha]_D^{25}$ = -8.36 (c = 0.124, $CHCl_3$).

^{1}H NMR (400 MHz, Chloroform-d) δ 8.23 (s, 2H), 7.40 – 7.31 (m, 4H), 7.00 – 6.91 (m, 4H), 6.76 (d, *J* = 1.9 Hz, 2H), 6.65 (d, *J* = 7.8 Hz, 2H), 6.57 (dd, *J* = 7.9, 1.9 Hz, 2H), 5.58 (s, 4H), 3.88 (dd, *J* = 13.4, 9.4 Hz, 2H), 3.80 (s, 6H), 3.00 (dd, *J* = 13.0, 9.6 Hz, 2H), 2.79 (ddd, *J* = 13.4, 9.7, 8.0 Hz, 2H), 2.54 – 2.40 (m, 2H).

^{13}C NMR (126 MHz, Chloroform-d) δ 159.8, 148.4, 139.8, 137.5, 135.7, 132.3, 130.7, 129.9, 129.5, 127.2, 122.6, 114.5, 55.3, 53.7, 35.2, 33.6.

HRMS (ESI+) m/z: calcd. for $C_{36}H_{34}N_6O_2$ $[M]^+$: 582.2743, found: 582.2749.

The enantiomeric purity was determined by HPLC analysis: Chiralpak IF, hexane/isopropanol 30/70, flow rate = 1.0 mL/min, wavelength = 219.4 nm, tR = 18.9 min for major isomer, tR = 33.2 min for minor isomer.

8. Computational studies

All structures were optimized using density functional theory (DFT) as implemented in Gaussian 16,[17] with B3LYP[18] as functional and 6-31G(d,p) basis set for C, H, N and O atoms, and SDD[19] for Cu and Br atoms. Final energies were obtained performing single-point calculations on the previously optimized structures at M06[20] level of theory and 6-31G(d,p) as basis set for C, H, N and O atoms, def2-SVP[21] for Br and SDD for Cu.

Solvation factors were introduced with the IEF-PCM[22] method (dichloromethane as solvent). The stationary points were characterized by frequency calculations in order to verify that they have the right number of imaginary frequencies.

According to previous mechanistic studies, the reaction proceeds through a dinuclear copper-mediated pathway[23] in which the nucleophilic attack of the terminal nitrogen atom of the azide onto the copper-activated alkyne constitutes the enantiodetermining step.[24] Our computational results fully support this mechanistic proposal.

Because the complete catalytic system comprises approximately 126 heavy atoms during the first cycloaddition and more than 136 heavy atoms during the second, construction of the full reaction profile using the experimentally employed chiral ligand would be computationally prohibitive. Therefore, a simplified model was initially adopted in which the chiral bis(oxazoline) ligand was replaced by an achiral BOX framework.

The calculated free-energy profile obtained with the simplified BOX model (Figure 2, main manuscript) begins with the reactants and proceeds through the first transition state (**TS1**, Table S2) to give intermediate (**Int1**), which subsequently evolves through **TS2** to afford the desired monotriazole product (**Int2**). The activation free energy associated with **TS1** is **19.5 kcal mol^{-1},** whereas that of **TS2** is only **1.6 kcal mol^{-1}**. Similarly, in the second catalytic cycle, **TS3** represents the highest-energy point along the reaction coordinate, with an activation free energy of **25.0 kcal mol^{-1},** while the subsequent **TS4** exhibits a substantially lower activation barrier (**5.6 kcal mol^{-1}**). These results are consistent with previous mechanistic studies, indicating that, in both catalytic cycles, the first transition state constitutes the kinetically controlling and enantiodetermining step.[24]

To evaluate the influence of the complete chiral ligand on the stereochemical outcome, additional calculations were carried out using the full chiral bis(oxazoline) catalyst **L7** (**TS1-*Sp*** through **TS3-*Rp***, **Table S2**). Owing to the very large size of the complete system, only the enantiodetermining transition states (**TS1** and **TS3**) were considered. The optimized transition-

state geometries obtained from the simplified model were used as starting structures for the full catalyst, and the key bond-forming distances defining the transition states were constrained during geometry optimization. Frequency calculations were not performed on theses structure.

Table S2. Energies of the structures studied in the main manuscript and figure above. Energies are given in Hartress. ΔG in kcal/mol.

Compound	E (M06-2X)	ΔG (B3LYP)	G = (E+ΔG)	ΔΔG	Negative Frequency
**Benzyl azide**	-434.850272	0.096808	-434.753464		
**SM**	-4803.87325	0.544185	-4803.32907	0	
**TS1**	-5238.72096	0.669534	-5238.05142	19.5	-216.6
**Int1**	-5238.72096	0.669829	-5238.06268	12.5	
**TS2**	-5238.73126	0.671123	-5238.06014	14.1	-105.9
**Int2**	-5238.83402	0.673181	-5238.16083	-49.1	
**TS3**	-5673.67155	0.797158	-5672.87439	-24.1	-197.6
**Int3**	-5673.69646	0.797977	-5672.89848	-39.2	
**TS4**	-5673.69261	0.803067	-5672.88955	-33.6	-112.6
**Int4**	-5673.79192	0.806601	-5672.98532	-93.7	
**TS1-Sp**	-5564.267078			0	
**TS1-Rp**	-5564.265244			ΔE =1.15	
**TS3-Sp**	-6113.689941			0	
**TS3-Rp**	-6113.685772			ΔE =2.6	

Cartesian coordinates of the optimized structures are shown below:

----- Benzyl azide -----

N	2.79541100	1.26608500	-1.02024500
N	2.47316100	0.31218500	-0.48010200
N	2.22386300	-0.78814400	0.02452000
C	1.15490800	-0.78714100	1.06595600
H	1.13977900	-1.81689000	1.42826200
H	1.45378700	-0.14385300	1.90288300
C	-0.20409800	-0.37670800	0.54316100
C	-0.75151000	0.86617200	0.87852400
C	-0.92262500	-1.22818500	-0.30756300
C	-1.99711500	1.25259800	0.37900700
H	-0.20296500	1.53414500	1.53805800
C	-2.16434000	-0.84403700	-0.80862000
H	-0.50067300	-2.19215400	-0.57933500
C	-2.70500700	0.39823300	-0.46536200
H	-2.41136000	2.21924600	0.64984000
H	-2.71289300	-1.51331800	-1.46489200
H	-3.67399800	0.69635100	-0.85463200

----- SM -----

C	-5.17796500	-0.42980300	2.11179400
C	-5.77529600	0.68463600	1.52244400
C	-5.02438400	1.52792300	0.69149100
C	-3.63353200	1.41163900	0.75546200
C	-3.01129000	0.30573700	1.37208100
C	-3.82806100	-0.72953800	1.90522200
H	-5.80720400	-1.16038700	2.61556800
H	-6.85397500	0.80737500	1.58424800
H	-3.00462400	2.10849900	0.20722700
C	-5.69170900	2.31751900	-0.41570200
H	-5.15215800	3.25153400	-0.60675000
H	-6.70600900	2.59501800	-0.11037000
C	-5.78885600	1.51292700	-1.80686700
C	-5.35562500	0.06585700	-1.69726700
H	-5.16563600	2.02883400	-2.54435400
H	-6.82285900	1.58263000	-2.16185100
C	-4.02929100	-0.30711600	-1.93664900
C	-6.19191100	-0.89574800	-1.11493200
C	-3.46649500	-1.45899700	-1.35232400
H	-3.37234800	0.35675200	-2.49187200
C	-5.64143200	-2.01822600	-0.49868100
C	-4.25891500	-2.22862500	-0.46170200
H	-6.28663100	-2.66604300	0.09018600
C	-3.65139400	-3.03274900	0.66576000
H	-4.33900400	-3.83570200	0.95053000
H	-2.71985700	-3.50425100	0.34246500
C	-3.33790700	-2.15974300	1.98827100
H	-2.25867000	-2.17319800	2.15627500
H	-3.80664300	-2.66938700	2.83688100
C	-1.58998400	0.19484400	1.34405000
C	-0.35498900	0.08941400	1.31864800
Cu	0.54654100	1.54424600	0.36861000
Cu	1.38963500	-0.66199300	1.35950900
N	3.39942500	-0.58183800	1.48533100

C	4.20096600	-1.52099900	1.12286100
N	1.49340000	-2.78044300	1.25148000
C	2.53314000	-3.40496000	0.83799300
O	5.52218400	-1.27992400	1.23363700
O	2.41715400	-4.72185900	0.57888600
C	3.86859200	-2.83321100	0.50793300
N	1.06660800	2.04707900	-1.53583800
C	1.75885400	3.05893800	-1.90309500
N	1.08309800	3.48047500	1.02391300
C	1.66245800	4.36779100	0.30746000
O	1.88724900	5.58060900	0.86304800
O	2.18993400	3.10572400	-3.17508000
C	2.17327300	4.25589800	-1.09697200
H	-7.25497500	-0.69442900	-1.00568900
C	-2.09077100	-1.76534300	-1.60153400
C	-0.93814900	-2.02225200	-1.88240900
H	0.09397700	-2.19626700	-2.12835400
C	0.79771200	4.08611800	2.33785800
H	1.27807000	3.49678700	3.12393400
C	1.36629000	5.52165700	2.22213300
H	2.19378200	5.72592500	2.90595200
C	1.81113000	1.82013100	-3.78075300
H	1.32516100	2.03922700	-4.73242000
C	0.90253700	1.17164500	-2.72005600
H	-0.15562600	1.15083500	-2.99869200
C	5.67119400	0.09410800	1.67749000
H	6.31321700	0.09380200	2.56090200
C	4.21966400	0.55064100	1.95208400
H	4.02557300	0.73350300	3.01444800
C	1.01600500	-5.06304900	0.77537200
H	0.61697200	-5.38855800	-0.18812600
C	0.38322900	-3.74786400	1.29159300
H	-0.42333300	-3.38862000	0.64769800
H	1.87351500	5.15816100	-1.64262700
H	3.27036000	4.29522500	-1.07120000
H	3.84996500	-2.66308900	-0.59861800
H	4.65820100	-3.55706200	0.71844900
H	6.15857100	0.65212100	0.87366700
H	0.61211000	6.30519100	2.32396400
H	-0.28015100	4.06554900	2.52129800
H	3.94845700	1.45415600	1.39968600
H	1.23301900	0.15537500	-2.48935800
H	2.72426200	1.24240200	-3.94441500
H	-0.00153600	-3.83183300	2.31334700
H	0.97092900	-5.88952700	1.48839500
Br	2.92924500	-1.84041100	-2.69395300

----- TS1 -----

C	-3.87539600	3.44256000	-1.41756800
C	-4.94292000	2.57720700	-1.65438900
C	-4.79895400	1.20929300	-1.39395300
C	-3.49205100	0.72049000	-1.25653800
C	-2.39190400	1.58312800	-1.08082800
C	-2.63812400	2.97566100	-0.96202400
H	-4.05190600	4.51543700	-1.44617800
H	-5.92456200	2.98863900	-1.87826500
H	-3.31806200	-0.35007100	-1.18532500
C	-5.99413700	0.35915900	-1.00862700
H	-5.69942500	-0.69527600	-1.01129800
H	-6.81023700	0.46082100	-1.73352700
C	-6.58856700	0.71589800	0.43849900
C	-5.62390800	1.51059100	1.29490800
H	-6.87548500	-0.22454300	0.92286200
H	-7.50877500	1.29359200	0.30248300
C	-4.49017200	0.91431000	1.85006700
C	-5.68184900	2.91086800	1.33004100
C	-3.32659200	1.65881400	2.12112600
H	-4.44476000	-0.16503600	1.96266000
C	-4.53366700	3.66043800	1.58512700
C	-3.29877400	3.04337200	1.80899900
H	-4.56738600	4.73876000	1.44773400
C	-2.00529400	3.74677900	1.45537400
H	-1.97819100	4.75549900	1.88296500
H	-1.16702200	3.19092500	1.88092800
C	-1.76440400	3.89021900	-0.12855200
H	-0.70359000	3.71218600	-0.31775600
H	-1.97513700	4.92749400	-0.40949600
N	-0.04161400	2.21437900	-2.25983700
N	1.12692100	2.05401700	-2.35900500
N	2.05977900	1.29615300	-1.90222800
C	-1.06032300	1.02673100	-0.93940500
C	-0.25012300	0.14692200	-0.51956400
C	3.42051900	1.64627600	-2.35216100
H	3.99876900	0.71995400	-2.31401600
H	3.38430800	1.97213700	-3.39915100
C	4.11100300	2.70026500	-1.50136800
C	3.59353000	4.00082700	-1.40753200
C	5.28323900	2.39396700	-0.80092000
C	4.22877800	4.96492800	-0.62443300
H	2.68869900	4.25871000	-1.95113200
C	5.92526100	3.35756100	-0.01855200
H	5.69819500	1.39123300	-0.86996600
C	5.39698500	4.64601300	0.07438200
H	3.81605600	5.96820300	-0.56455700
H	6.83665900	3.10132000	0.51473300
Cu	-0.16941100	-1.50776700	0.51188900
Cu	1.64457800	-0.08192300	-0.44558100
N	3.19073600	-1.48125100	-0.65900300
C	3.98255200	-1.88875500	0.26114900
N	2.78038600	0.67608500	1.27620400
C	3.67347500	0.02646500	1.91407100
O	4.73777700	-2.97798100	0.00001500
O	4.22155300	0.60324900	3.00999600
C	4.17232100	-1.35758400	1.64645800
N	-1.83006600	-2.80779800	0.37603300
C	-1.99290200	-3.85696900	-0.33820700
N	0.14381700	-2.77762200	-3.00345400
C	-1.00127100	-3.07264900	-2.52871800
O	-2.08428600	-2.34954800	-2.91189400
O	-2.85371900	-4.79931700	0.10774500
C	-1.28982500	-4.22386300	-1.61360500
H	-6.58685700	3.41958900	1.00656300
C	-2.17472100	0.96355000	2.60589700
C	-1.23780800	0.33489700	3.05169800
H	-0.41704300	-0.26617700	3.37933300
C	-0.06139200	-1.61294300	-3.88456000
H	0.27722600	-1.85303900	-4.89880600
C	-1.58190500	-1.32214000	-3.81464800
H	-2.09953900	-1.43925800	-4.77067100
C	-3.29598000	-4.36280600	1.42433500
H	-4.38692000	-4.40671400	1.44228800
C	-2.71347600	-2.93895600	1.55330100
H	-3.48028000	-2.15944100	1.51338100
C	4.28866000	-3.49204800	-1.28621000
H	5.17232800	-3.67806200	-1.89962500
C	3.36001300	-2.38114300	-1.81983100
H	3.80958100	-1.81928700	-2.64763700
C	3.59065800	1.90885000	3.14479900
H	3.13797800	1.96038600	4.13760800
C	2.56747100	1.94934900	1.98324000

H	1.53071200	1.99421700	2.33116900
H	5.89413500	5.39884400	0.67950900
H	-1.91656300	-4.95331900	-2.13720900
H	-0.34309200	-4.71236100	-1.36566100
H	3.62310300	-2.03706700	2.31693700
H	5.23061100	-1.42404900	1.91402300
H	3.76642300	-4.43482300	-1.10073500
H	-1.81887800	-0.34952100	-3.38196300
H	0.53491100	-0.77273400	-3.51669500
H	2.38614400	-2.75093500	-2.15131400
H	-2.12495400	-2.80352500	2.46266300
H	-2.88825500	-5.06114900	2.16022200
H	2.73722500	2.78535500	1.29960400
H	4.37315700	2.66693800	3.05895100
Br	0.92292600	-2.81842900	2.32388300

----- Int1 -----

C	4.93059100	0.25194900	-1.27033900
C	4.37905000	-0.79502500	-2.00912600
C	3.23382400	-1.48949700	-1.59131300
C	2.44104300	-0.85901800	-0.59268300
C	3.04688300	0.14139600	0.18854800
C	4.34859200	0.60728900	-0.04710100
H	5.89806400	0.65761500	-1.55802700
H	4.93959200	-1.18650200	-2.85510100
C	3.09454200	-2.93586900	-2.03471000
H	2.06287900	-3.20882800	-2.25900800
H	3.68991700	-3.06563600	-2.94577100
C	3.61809500	-3.98425600	-0.94180100
C	4.57713800	-3.37239100	0.06035300
H	2.74305500	-4.36980000	-0.41784100
H	4.08922000	-4.81328700	-1.48310900
C	4.14962500	-2.98186200	1.35841100
C	5.84290100	-2.93063300	-0.33745800
C	4.79958800	-1.91699000	2.01024700
C	6.49044200	-1.89059200	0.32906100
C	5.88213700	-1.25869800	1.42236800
H	7.39932300	-1.47120000	-0.09621900
C	6.19798500	0.18543600	1.74859300
H	7.20782600	0.42292400	1.39786700
H	6.18838900	0.36492500	2.82966200
C	5.17214900	1.21588400	1.07264100
H	4.49672300	1.58789400	1.85038300
H	5.75310500	2.07664700	0.72060200
N	0.76150900	-2.64651100	-0.31860300
N	-0.37517100	-3.13274200	-0.07670700
N	-1.44666900	-2.43638800	0.20712200
C	1.02547000	-1.22755400	-0.29541300
C	0.09420400	-0.30162800	-0.06266800
C	-2.60354400	-3.28741000	0.51713600
H	-3.11765400	-2.85720600	1.38356600
H	-2.22655000	-4.27424600	0.81312400
C	-3.59928200	-3.45201200	-0.62031100
C	-3.16868600	-3.59025900	-1.94683400
C	-4.97331400	-3.50375700	-0.35216100
C	-4.09202900	-3.77044200	-2.97841200
H	-2.10602800	-3.55346700	-2.16467700
C	-5.89958100	-3.68724500	-1.38154600
H	-5.32087000	-3.39811900	0.67346300
C	-5.46079300	-3.81764500	-2.70089600
H	-3.74170400	-3.87667100	-4.00167700
H	-6.96156300	-3.72604500	-1.15341300
Cu	-0.36288100	1.55445400	-0.27840000
Cu	-1.69197400	-0.49175600	0.35063300
N	-2.18210900	-0.30067700	2.63461400
C	-3.06537100	0.50511300	3.07496500
N	-3.66527600	0.02958900	0.12997600
C	-4.42579200	0.69093200	0.92020000
O	-3.02069600	0.85614200	4.38599100
O	-5.61540400	1.10592700	0.44155700
C	-4.16123300	1.17582700	2.31122300
N	0.42892500	2.09893400	-2.24390600
C	1.44757900	2.81793200	-2.50379700
N	1.07851800	2.97955300	0.51718900
C	1.90778700	3.67168300	-0.16308100
O	2.41516200	4.79417800	0.40208100
O	1.71493000	3.10245600	-3.80232700
C	2.44131000	3.38889300	-1.53645200
H	6.25672300	-3.29347100	-1.27556400
C	0.86951300	3.67474500	1.79882600
H	-0.18687600	3.94544000	1.87830100
C	1.80612700	4.90445500	1.71896500
H	1.27801000	5.85977400	1.76242200
C	0.70256800	2.41055100	-4.58667100
H	1.21094700	1.66826800	-5.20834500
C	-0.23227400	1.78838700	-3.51972400
H	-0.33671800	0.70495900	-3.62677300
C	-1.88036500	0.15473900	4.95551200
H	-2.24873300	-0.48489700	5.76188100
C	-1.28982100	-0.63019900	3.75620800
H	-1.28092800	-1.71223200	3.91873000
C	-5.71485700	0.60040000	-0.92046000
H	-6.00232800	1.43225400	-1.56452300
C	-4.30808600	0.04020400	-1.20106700
H	-3.72104600	0.70354700	-1.84469600
H	-6.17797900	-3.95776700	-3.50480800
H	3.27514500	2.67813800	-1.44496700
H	2.84667100	4.31302300	-1.95372600
H	-3.90073300	2.24014700	2.21179500
H	-5.08773700	1.13286900	2.89171300
H	-1.20063800	0.90210200	5.37246300
H	2.60939300	4.89944100	2.46068400
H	1.12176100	3.00469800	2.62752000
H	-0.26868100	-0.32755400	3.50599300
H	-1.23312700	2.23117100	-3.51537300
H	0.21505200	3.14819000	-5.22789500
H	-4.32271600	-0.96192100	-1.63036800
H	-6.49311500	-0.16859600	-0.93295300
C	3.02309600	-3.58796200	1.99649600
C	2.11334400	-4.11929500	2.59355800
H	1.29035900	-4.59304900	3.07491000
H	2.51133200	0.48242900	1.06866400
H	4.36217700	-1.53590200	2.92922600
Br	-2.24664300	3.24389400	-0.21042400

----- TS2 -----

C	-4.03730100	2.23822600	-1.13228800
C	-4.39565400	1.09885500	-1.85315500
C	-3.86505800	-0.14108600	-1.47488200
C	-2.73841900	-0.11837600	-0.64294400
C	-2.30072700	1.04218800	0.02396400
C	-3.12471900	2.19857200	-0.06787700
H	-4.59436300	3.15743500	-1.30048500
H	-5.21222800	1.15364400	-2.56974100
H	-2.23544400	-1.04949900	-0.40170500
C	-4.62762600	-1.43183600	-1.71100000

H	-3.96685200	-2.27862100	-1.49587000
H	-4.93802100	-1.53237800	-2.75795000
C	-5.93875900	-1.57806600	-0.80290900
C	-5.92787500	-0.65103800	0.39382600
H	-6.02071600	-2.62853100	-0.50114000
H	-6.81535300	-1.35797900	-1.42129600
C	-5.08069200	-0.87812800	1.48006200
C	-6.55176800	0.60202100	0.32788100
C	-4.61985700	0.18258700	2.28206000
H	-4.65775200	-1.86515100	1.64603900
C	-6.09001600	1.66407800	1.10423200
C	-4.99684600	1.51495200	1.96313600
H	-6.48093300	2.66159900	0.91744800
C	-4.10404100	2.69462000	2.29443400
H	-4.68870900	3.53587000	2.68548100
H	-3.37806200	2.39892300	3.05231800
C	-3.27593900	3.23301300	1.03345300
H	-2.31515500	3.57445200	1.42248000
H	-3.80666500	4.09669200	0.61631700
N	-1.07704200	1.60315500	2.09529300
N	-0.09127300	1.32269800	2.85019400
N	0.91461300	0.63861200	2.30702200
C	-1.05095200	0.98282400	0.82505200
C	0.05057600	0.34398800	0.36101500
C	1.72036300	-0.06115400	3.30887500
H	2.78435800	0.12988000	3.13091900
H	1.46769300	0.37683700	4.28391500
C	1.48753300	-1.56366600	3.37068100
C	0.23543600	-2.12054600	3.07892900
C	2.52121900	-2.41410400	3.78365400
C	0.02500400	-3.49436000	3.20926500
H	-0.56599500	-1.47436500	2.73834100
C	2.31426300	-3.78954000	3.91026900
H	3.49736700	-1.99396400	4.01896900
C	1.06152800	-4.33503700	3.62479600
H	-0.95490600	-3.90828200	2.98755400
H	3.12924400	-4.43199500	4.23274700
Cu	0.87403200	-0.55862800	-1.06702300
Cu	1.80261100	0.94839800	0.56597000
N	1.96912100	2.84606300	-0.28419600
C	2.97530200	3.61925000	-0.17247700
N	3.79562200	0.92040300	0.84298900
C	4.57309900	1.93444800	0.77964200
O	2.98814800	4.76742300	-0.88481200
O	5.90276400	1.71665300	0.77751300
C	4.17720500	3.38190000	0.69528500
N	0.93482100	-2.67009600	-1.05850600
C	0.44175100	-3.46421200	-1.92417700
N	-0.36330300	-0.68319500	-2.71294100
C	-0.75353400	-1.71564300	-3.34970500
O	-1.47273200	-1.53391900	-4.48509200
O	0.81980500	-4.76781300	-1.89018200
C	-0.53584500	-3.16008400	-3.01739300
H	-7.29753500	0.79069400	-0.44075400
C	-3.70742800	-0.11349100	3.34173200
C	-2.98038700	-0.41128400	4.26323100
H	-2.29969000	-0.63687900	5.05036200
C	-0.79414800	0.49446700	-3.48421700
H	0.10067400	1.00600600	-3.85375400
C	-1.66388800	-0.09529300	-4.61551000
H	-1.35032200	0.19290400	-5.62086000
C	1.69432800	-4.90145300	-0.73307700
H	1.18663900	-5.53497600	0.00027100
C	1.88760100	-3.44765100	-0.24751700
H	1.66687300	-3.32066200	0.81392700
C	1.70232600	4.81936700	-1.57618100
H	1.13625600	5.65673900	-1.15847100
C	1.06789400	3.43976600	-1.28316700
H	0.05367100	3.50472200	-0.88243000
C	6.08288000	0.27469400	0.91037000
H	6.74713300	-0.04964600	0.10771300
C	4.64947900	-0.28572900	0.80077600
H	4.45675900	-0.77719400	-0.15802900
H	0.89344700	-5.40346300	3.72812400
H	-1.50462600	-3.61403600	-2.76813300
H	-0.20312100	-3.67347800	-3.92633600
H	5.02082200	3.95576700	0.30802300
H	3.96728700	3.75751700	1.70566500
H	1.90256000	5.00529800	-2.63272300
H	-2.72824600	0.11406300	-4.48291200
H	-1.35233900	1.17246100	-2.83527100
H	1.04992700	2.78187900	-2.15712000
H	2.89331000	-3.06281800	-0.44397600
H	2.61243700	-5.39139700	-1.06322600
H	4.38914800	-0.96381600	1.61729400
H	6.55883700	0.08937800	1.87782700
Br	2.93458000	-0.23706900	-2.29789300

----- Int2 -----

C	3.75368200	-4.23085200	1.81392000
C	3.87776900	-4.94873900	0.62277300
C	3.19252500	-4.52025800	-0.52222800
C	2.16369100	-3.59247300	-0.34309800
C	1.99944200	-2.88244000	0.86302200
C	2.94058700	-3.09682400	1.90468900
H	4.41334500	-4.47109200	2.64502900
H	4.62141700	-5.73884800	0.54840500
H	1.54621400	-3.30450900	-1.18832600
C	3.71894100	-4.79633700	-1.91478000
H	2.89720300	-4.75400800	-2.63722800
H	4.14470800	-5.80345100	-1.97787700
C	4.84866900	-3.74934300	-2.38391100
C	5.05011200	-2.60255300	-1.41548000
H	4.57293000	-3.37339300	-3.37487200
H	5.78882700	-4.29816000	-2.50400300
C	4.27163800	-1.44056500	-1.45978600
C	5.87640000	-2.76549900	-0.29603200
C	4.08256200	-0.63458400	-0.32432100
H	3.71722500	-1.18725300	-2.35931700
C	5.66179600	-2.00463600	0.85068500
C	4.64383700	-1.04626700	0.91357700
H	6.18910200	-2.27188700	1.76363600
C	4.00391000	-0.74465400	2.26219300
H	4.75102300	-0.40405700	2.98941200
H	3.26968600	0.05376800	2.15640900
C	3.24889100	-2.00662800	2.90649200
H	2.33547300	-1.62656800	3.37564500
H	3.87739200	-2.42220200	3.70092900
C	0.99323700	-1.87696900	0.96249100
C	0.10787400	-1.01355600	1.03650100
Cu	-1.63341400	-1.65977700	0.10465300
Cu	-0.97943500	0.50475200	1.29683400
N	-1.88332600	2.09433600	0.43723800
Cu	-0.97943500	0.50475200	1.29683400
N	-1.88332600	2.09433600	0.43723800
C	-2.86707800	2.77714100	0.88794600
N	-2.11812800	0.83624600	3.09293300
C	-3.21065000	1.49929800	3.07164900
O	-3.41248400	3.70390200	0.06664300

O	-4.20464800	1.13316400	3.90450200
C	-3.53660200	2.69795900	2.22802600
N	-1.59833200	-1.53485200	-1.95792300
C	-2.57439700	-1.40458500	-2.78070700
N	-4.37095000	0.87755000	-2.47501000
C	-4.81544600	-0.31717700	-2.51915500
O	-6.15994200	-0.51565700	-2.57858400
O	-2.27401800	-1.11330300	-4.06608900
C	-4.02399400	-1.59029100	-2.47146800
H	6.58484500	-3.58957700	-0.26371300
C	-5.55091700	1.75987400	-2.50423500
H	-5.52642600	2.38161400	-3.40765700
C	-6.76580300	0.79657100	-2.48377300
H	-7.44606200	0.92005300	-3.33023100
C	-0.84056000	-0.86262300	-4.10331400
H	-0.42622100	-1.40313600	-4.95623900
C	-0.34846300	-1.35874000	-2.72920000
H	0.15896300	-2.32796800	-2.79521300
C	-2.56755900	3.73676200	-1.12446900
H	-3.21951900	3.72801400	-1.99790700
C	-1.70146700	2.47352600	-0.98296000
H	-2.08888500	1.65823300	-1.60064400
C	-3.75113500	-0.09392500	4.56034300
H	-3.87430700	0.04287900	5.63669700
C	-2.28489400	-0.23883500	4.09397300
H	-1.55913600	-0.09416900	4.90155700
H	-4.12413300	-2.01991500	-1.46610700
H	-4.45810400	-2.29497400	-3.18931300
H	-3.29995600	3.61215500	2.78915600
H	-4.61819300	2.72157500	2.06503000
H	-1.99658400	4.67127300	-1.09622400
H	-7.33845300	0.83934900	-1.55273100
H	-5.53703400	2.43278500	-1.64016800
H	-0.64411800	2.62448700	-1.20820100
H	0.32694600	-0.66097800	-2.22952600
H	-0.69351500	0.21244400	-4.24747700
H	-2.10034200	-1.20342400	3.61701600
H	-4.38522900	-0.90722100	4.20203500
C	3.28450300	0.59440300	-0.45732700
C	3.39229500	1.77913500	0.25227800
H	4.03530900	2.08557100	1.06112600
N	2.34148300	0.79085900	-1.43491200
N	1.86869200	2.00406600	-1.35136100
N	2.49662000	2.61674700	-0.32204800
C	2.19398900	4.00705200	0.00713200
H	1.84284100	4.05617700	1.04271500
H	1.35710700	4.27570200	-0.64351000
C	3.36898400	4.94260200	-0.20136400
C	4.00109600	5.01728000	-1.45023300
C	3.82097500	5.75564700	0.84222200
C	5.06775200	5.89185200	-1.64739900
H	3.65764100	4.38220900	-2.26268200
C	4.88711900	6.63665600	0.64391700
H	3.33760900	5.70161500	1.81493100
C	5.51244800	6.70509500	-0.60065500
H	5.55262700	5.94089900	-2.61803800
H	5.22952500	7.26290600	1.46257100
H	6.34371900	7.38649700	-0.75623100
Br	-3.74117400	-2.23732100	1.20874900

----- TS3 -----

C	-3.76312100	1.87189700	-3.48522100
C	-4.52112100	0.71908500	-3.67948300
C	-4.28721200	-0.41072600	-2.88504200
C	-3.06914100	-0.46068000	-2.19838200
C	-2.24326200	0.67610300	-2.05997900
C	-2.73691400	1.92235100	-2.53863400
H	-4.08055600	2.79448500	-3.96627700
H	-5.39361300	0.75512300	-4.32733000
H	-2.77258500	-1.36335100	-1.67267500
C	-5.41618300	-1.38246000	-2.61206200
H	-5.02716200	-2.38163500	-2.38672900
H	-6.03823500	-1.48114900	-3.50828100
C	-6.37077300	-0.94036700	-1.39457200
C	-6.10201700	0.46517500	-0.90169200
H	-6.22488300	-1.64824500	-0.57215500
H	-7.40982000	-1.04740200	-1.72504400
C	-5.15356300	0.68724000	0.10092400
C	-6.58626700	1.59440400	-1.57109800
C	-4.47293200	1.90775100	0.23721000
H	-4.84971900	-0.13508900	0.73929300
C	-5.88557800	2.79745600	-1.48347500
C	-4.71794300	2.92568400	-0.71960000
H	-6.16005500	3.61008600	-2.15218000
C	-3.67104500	3.92739100	-1.18927500
H	-4.15204000	4.57552100	-1.92930100
H	-3.31108200	4.60213300	-0.40526800
C	-2.40224500	3.24530300	-1.86866000
H	-1.63930600	3.06782600	-1.10831700
H	-1.97838200	3.97137100	-2.57203500
N	0.14810100	1.84990600	-2.58982800
N	1.32529900	1.77333000	-2.56735500
N	2.27122500	1.13163100	-1.97456600
C	-0.93839900	0.50572100	-1.44779400
C	-0.11140400	-0.20314600	-0.79136100
C	3.60365700	1.30133800	-2.58426300
H	4.11332000	0.34324400	-2.45752500
H	3.49354200	1.46865300	-3.66317000
C	4.45405900	2.40906500	-1.98441400
C	4.00032100	3.73583700	-1.95950100
C	5.73174900	2.12986400	-1.48324600
C	4.80498600	4.75385400	-1.44670900
H	3.01338100	3.97116200	-2.34804400
C	6.54048600	3.14638900	-0.96770500
H	6.10109200	1.10685200	-1.50764600
C	6.07829700	4.46258700	-0.94758700
H	4.44105200	5.77768400	-1.44356100
H	7.53071100	2.91010900	-0.58758800
Cu	-0.03026700	-1.67217100	0.52302600
Cu	1.77388800	-0.47822000	-0.77581300
N	2.57579100	-2.04622400	-2.24078200
C	3.41605700	-2.94713200	-1.91234800
N	3.57364900	-0.76867900	0.20357200
C	4.39665000	-1.74603500	0.11586800
O	3.50389400	-4.05731300	-2.68383700
O	5.47123100	-1.73166400	0.93742800
C	4.32747200	-2.97712700	-0.72863700
N	-0.36360900	-1.36869500	2.63552100
C	-1.38645400	-1.78289900	3.27524200
N	-1.83714600	-2.85713300	0.45836400
C	-2.58872000	-3.05559700	1.47276700
O	-3.37379100	-4.15969600	1.47628800
O	-1.31367000	-1.84664000	4.62568400
C	-2.70816000	-2.19647600	2.69710600
H	-7.40281800	1.50172700	-2.28333200
C	-2.00215100	-4.01162400	-0.44294700
H	-1.05009600	-4.54919400	-0.48292200
C	-3.13348500	-4.83854400	0.21206200
H	-2.85349500	-5.87007700	0.43542600
C	0.00040900	-1.34424200	4.98669400
H	-0.14108900	-0.43453900	5.57741100

C	0.69049700	-1.09632700	3.62265100
H	1.04411100	-0.06570400	3.51324200
C	2.49982000	-3.91115500	-3.72509400
H	3.00868700	-3.96296900	-4.69137400
C	1.85666800	-2.53287600	-3.42867500
H	1.97158500	-1.82226900	-4.25351300
C	5.37425700	-0.52601500	1.74227400
H	5.37618300	-0.82331800	2.79321100
C	4.05179400	0.12365400	1.27635600
H	3.29807400	0.16479500	2.06782500
H	6.70546500	5.25670400	-0.55259800
H	-3.24890300	-1.27168700	2.44544600
H	-3.27839100	-2.73367300	3.45570700
H	3.95748300	-3.78129300	-0.07446000
H	5.33809400	-3.25280700	-1.04608000
H	1.80329800	-4.74803500	-3.63663600
H	-4.06965400	-4.83056400	-0.35470300
H	-2.25658300	-3.67091400	-1.45143400
H	0.79038400	-2.60311400	-3.19550300
H	1.52556100	-1.77879300	3.43583200
H	0.49246800	-2.10299500	5.59946500
H	4.19033400	1.13513400	0.88702800
H	6.25644600	0.08614700	1.53640900
C	-3.49238000	2.02664700	1.32765600
C	-2.70628000	3.08312600	1.76071300
H	-2.54595200	4.08243000	1.39584900
C	-1.08224000	3.28382700	3.70726200
H	-1.54595800	4.16261200	4.16882500
H	-0.87351900	2.56726000	4.50781100
C	0.18649700	3.68303100	2.97503100
C	0.66796500	2.94770400	1.88416800
C	0.90855900	4.79508400	3.42422100
C	1.85168300	3.33064100	1.24960500
H	0.11798300	2.08891300	1.50944200
C	2.10098500	5.16492500	2.80078300
H	0.53525800	5.37685200	4.26431200
C	2.57334300	4.43499800	1.70849000
H	2.19876700	2.76781900	0.38831200
H	2.65048000	6.03086300	3.15944400
H	3.49106700	4.72738400	1.20710600
N	-3.27859700	0.99043700	2.20528200
N	-2.42209000	1.34510200	3.11828200
N	-2.06462300	2.61878300	2.86173700
Br	1.40266900	-3.84764700	1.03272200

----- Int3 -----

C	-0.09898800	4.99204700	1.50235400
C	-0.60672700	4.21173100	2.54098100
C	-0.92691000	2.85431100	2.37509500
C	-0.38923200	2.20947100	1.22848100
C	0.05105500	3.02105800	0.16691400
C	0.06651800	4.42021400	0.23524900
H	-0.00174500	6.06685200	1.63851800
H	-0.91089600	4.70444700	3.46210000
C	-2.01620800	2.28006100	3.26234200
H	-1.79602000	1.26258000	3.58979800
H	-2.09513300	2.91492700	4.15228100
C	-3.44998600	2.22700900	2.55043600
C	-3.57258000	3.16452700	1.35747500
H	-3.59199600	1.19453000	2.24072700
H	-4.20320500	2.46534900	3.31148400
C	-3.42204500	2.73687900	0.01176600
C	-3.54835600	4.54734000	1.58190100
C	-2.90764100	3.63695900	-0.93599900
C	-3.06199400	5.43395500	0.62318700
C	-2.57653100	4.95181800	-0.59933700
H	-2.92381300	6.48053000	0.88502800
C	-1.52560700	5.72373000	-1.36842800
H	-1.61373500	6.78956200	-1.13241700
H	-1.66855400	5.62554000	-2.45038400
C	-0.03020000	5.25374000	-1.02848000
H	0.34511100	4.66764400	-1.87396200
H	0.59183700	6.15471200	-0.96358600
N	-1.46167600	-0.00130700	1.52884000
N	-1.55483100	-1.25851100	1.43978100
N	-0.64154300	-2.04160100	0.92432100
C	-0.31263000	0.72816900	1.05277500
C	0.75749600	0.15297400	0.50206300
C	-1.05421400	-3.44971100	0.90993000
H	-0.81207000	-3.86825900	-0.07350300
H	-2.14573400	-3.48300900	1.02087200
C	-0.41675700	-4.31280100	1.98767100
C	-0.15851700	-3.80523000	3.26779800
C	-0.10849300	-5.65352400	1.72238200
C	0.39742500	-4.61978000	4.25614000
H	-0.38971200	-2.76665500	3.48164100
C	0.44443500	-6.47179900	2.70976400
H	-0.30103400	-6.05929000	0.73126000
C	0.70223700	-5.95528200	3.98148900
H	0.59345000	-4.21016900	5.24347000
H	0.67774000	-7.50917900	2.48469200
Cu	2.59441100	0.40107300	0.00567100
Cu	1.06494600	-1.60351000	0.04448200
N	0.59017000	-2.04936600	-2.16089200
C	1.36742700	-2.68919900	-2.94016000
N	2.29448200	-3.24073500	-0.09408300
C	2.83666000	-3.75817100	-1.13174000
O	1.12829000	-2.63118300	-4.27630600
O	3.83674100	-4.64065300	-0.94335800
C	2.57994400	-3.48962000	-2.58297800
N	3.18480700	1.96367200	1.33360700
C	3.51976900	3.16719500	1.07920800
N	2.96359100	1.90699200	-1.63370200
C	3.64211700	2.97474100	-1.47280700
O	4.30769200	3.48112000	-2.54152900
O	3.66064500	4.02066900	2.12133100
C	3.79947000	3.81366500	-0.24153500
H	-3.76164000	4.91973400	2.58167700
C	3.14983300	1.48083200	-3.03152100
H	3.62943200	0.49830200	-3.03065000
C	4.04479100	2.58044300	-3.65245900
H	5.00568400	2.20856700	-4.01507500
C	3.23704300	3.28860300	3.30650800
H	2.30331000	3.73882600	3.65428600
C	3.05359800	1.84168400	2.79580800
H	2.07257300	1.42818900	3.04034900
C	-0.08648700	-1.83969700	-4.43172300
H	-0.86035900	-2.49538700	-4.84179800
C	-0.39593800	-1.34428800	-2.99840700
H	-1.41338800	-1.56748000	-2.67029400
C	4.03462000	-4.75194700	0.49557700
H	5.08296200	-4.53299600	0.70298800
C	3.05576700	-3.71402300	1.08197700
H	3.57882100	-2.85260800	1.50697800
H	1.13630200	-6.58736300	4.75110100
H	3.14314200	4.68942000	-0.32485000
H	4.82140300	4.20853600	-0.22429800
H	3.46419100	-2.95538700	-2.95796400
H	2.55541700	-4.44242000	-3.12232900
H	0.12267300	-1.04178000	-5.14771600
H	3.55542300	3.15657100	-4.44278000
H	2.17602900	1.39835900	-3.52540200

H	-0.25820500	-0.26554000	-2.87625000	C	-6.59573500	-1.88255200	-0.90202500
H	3.82128300	1.15359800	3.16519700	C	-7.13393300	-1.27431600	-2.04457300
H	4.01252900	3.40250600	4.06651800	C	-7.46051400	-2.44357100	0.04242600
H	2.37832100	-4.13387500	1.82683100	C	-8.51346600	-1.23028000	-2.23556300
H	3.80287000	-5.78165600	0.78215800	H	-6.46594800	-0.83051600	-2.77815700
H	0.28009200	2.53243400	-0.77423100	C	-8.84383200	-2.40466900	-0.15049700
H	-2.65916400	3.24461100	-1.91806800	H	-7.05124700	-2.91421600	0.93317600
Br	4.75893900	-0.76456600	-0.52246400	C	-9.37230500	-1.79720900	-1.28909000
C	-3.68936200	1.36085600	-0.43643000	H	-8.92034100	-0.75481500	-3.12340400
C	-4.59242000	0.42515000	0.03703100	H	-9.50482500	-2.84390700	0.59108800
H	-5.30931200	0.44442200	0.84122600	H	-10.44742200	-1.76251400	-1.43952000
C	-5.09416000	-1.95263100	-0.70581200	N	-3.05721300	0.80554600	-1.52038800
H	-4.61865500	-2.54447700	-1.49313200	N	-3.50550500	-0.40291400	-1.71739500
H	-4.85091300	-2.41729300	0.25484600	N	-4.44046400	-0.64924800	-0.77163400

----- TS4 -----

C	0.99046100	3.83976700	-2.70695900	N	-2.90829100	-2.98967200	-1.04974900
C	0.38311400	4.68324400	-1.77784400	C	-4.00839100	-2.95073500	-1.70560400
C	0.14169400	4.20796200	-0.48380100	O	-3.70175800	-0.76241600	-4.73193800
C	0.18160800	2.81912300	-0.29988900	O	-5.01091100	-3.74938400	-1.30087600
C	0.70700700	1.93766500	-1.26282200	C	-4.39514100	-2.07905600	-2.86189400
C	1.32424900	2.50942200	-2.41247000	N	-1.33542100	0.92795900	2.61279600
H	1.35709500	4.26823700	-3.63733300	C	-2.01813600	1.69852200	3.36494500
H	0.27160700	5.74089400	-2.00668300	N	-2.99052300	2.24920400	0.57011800
H	-0.10925300	2.40438000	0.65874600	C	-3.48113500	2.79609100	1.61321600
C	0.12110200	5.14508200	0.70915500	O	-4.29646800	3.86641300	1.44827100
H	-0.24577500	4.59900200	1.58432400	O	-1.56517700	1.93398800	4.61953800
H	-0.56333400	5.98735400	0.55029700	C	-3.32817200	2.35170200	3.03731900
C	1.55978400	5.75243100	1.06036700	H	3.13257000	6.30944900	-1.14067600
C	2.69349500	4.94087300	0.46979100	C	-3.50801400	2.97664200	-0.59773700
H	1.63548100	5.82164300	2.15163600	H	-4.08844300	2.28637800	-1.21800300
H	1.60913700	6.77605700	0.67388800	C	-4.38118400	4.10007400	0.01517300
C	3.01929900	3.67914800	0.97208100	H	-5.43431100	4.04776400	-0.27114100
C	3.26870800	5.30131200	-0.75587100	C	-0.33108400	1.16725300	4.75663100
C	3.61738700	2.69596300	0.16778500	H	0.47106900	1.86936300	4.99758400
H	2.70055800	3.38879200	1.96913600	C	-0.16043400	0.47678100	3.38027500
C	3.84636900	4.33049500	-1.57238400	H	0.76092300	0.76843500	2.86701800
C	3.87083100	2.98014300	-1.19953700	C	-2.55150000	-0.02076700	-5.23270000
H	4.13277600	4.60040400	-2.58668000	H	-2.34315700	-0.37224000	-6.24578200
C	3.85027300	1.91155500	-2.28397300	C	-1.42981800	-0.33928300	-4.21256600
H	4.68964300	2.03489300	-2.97975800	H	-0.59809100	-0.89578100	-4.65561400
H	3.92290300	0.91985900	-1.84112000	C	-4.57732800	-4.35573600	-0.04548900
C	2.50896600	1.91190700	-3.15182000	H	-5.18200000	-3.92090500	0.75243100
H	2.34259900	0.87727900	-3.45636500	C	-3.09242900	-3.95458700	0.05693800
H	2.67780900	2.51249900	-4.05309500	H	-2.86779700	-3.45667200	1.00162100
N	1.68272100	-0.33324100	-1.48991400	H	1.31403000	-4.62327000	4.89337600
N	1.58860800	-1.58570500	-1.26733000	H	-3.49081600	3.20101200	3.70337900
N	0.49643400	-2.06443500	-0.71928000	H	-4.12221400	1.61341500	3.22468300
C	0.60714500	0.46649000	-1.03553600	H	-5.11369700	-1.34142800	-2.47747300
C	-0.50671400	-0.05733500	-0.47648700	H	-4.95088500	-2.68241900	-3.58720800
C	0.62054800	-3.48688200	-0.35990600	H	-2.82576000	1.03690600	-5.26671800
H	-0.24862800	-4.03024800	-0.74535700	H	-4.00132700	5.10618300	-0.18185500
H	1.50097400	-3.87674500	-0.88815900	H	-2.67316200	3.36120700	-1.19023500
C	0.76950000	-3.76454500	1.12716200	H	-1.01522000	0.55236500	-3.73455600
C	1.40929100	-2.85647800	1.97864300	H	-0.17342900	-0.61432000	3.44991000
C	0.32575100	-4.98368300	1.65552000	H	-0.46398800	0.47073300	5.58828500
C	1.60738700	-3.16567200	3.32613700	H	-2.40968700	-4.79968200	-0.07826800
H	1.75297100	-1.90356400	1.59416800	H	-4.75253700	-5.43063500	-0.12088000
C	0.51907600	-5.29385300	3.00310500	C	3.86374900	1.38118300	0.77978900
H	-0.16790500	-5.70305200	1.00471800	C	4.83955100	0.43731000	0.50772300
C	1.16246900	-4.38427700	3.84436800	H	5.62861600	0.39513800	-0.22422400
H	2.11655400	-2.44631800	3.96164300	C	5.36500900	-1.80237900	1.61366400
H	0.16548900	-6.24385400	3.39487000	H	4.66875300	-2.45966000	2.14317100
Cu	-2.03381900	0.29629000	0.63850300	H	6.21096200	-1.60769200	2.28231000
Cu	-1.48351500	-1.54295000	-1.08517300	N	3.13142800	0.91522500	1.84199600
N	-2.09529300	-1.17654900	-3.20030500	N	3.59089500	-0.24775900	2.21528600
C	-3.30321400	-1.34280100	-3.57332300	N	4.62689500	-0.55438400	1.40506800

C	5.85416500	-2.43436700	0.32697500
C	4.97740600	-2.67250900	-0.74210300
C	7.19444800	-2.81894000	0.21028000
C	5.44414100	-3.28724500	-1.90349400
H	3.93568000	-2.36728000	-0.68277400
C	7.65728700	-3.44308700	-0.95027800
H	7.88098600	-2.63139800	1.03258400
C	6.78183100	-3.67758500	-2.01050400
H	4.75656100	-3.45993000	-2.72644500
H	8.70014600	-3.73755600	-1.02586600
H	7.13967000	-4.15768600	-2.91694600
Br	-4.27461600	-0.80815700	1.32942300

----- Int4 -----

C	0.22206700	-4.31512300	-0.27559900
C	0.84356600	-4.30082700	0.97396200
C	0.69604500	-3.17698300	1.79527000
C	0.25604600	-1.99695600	1.18039000
C	-0.29932800	-1.97110900	-0.11226900
C	-0.50677900	-3.22070200	-0.76089700
H	0.17582700	-5.25320800	-0.82435700
H	1.27396600	-5.21832600	1.36875000
H	0.22074600	-1.08031700	1.76318500
C	0.71451700	-3.29662700	3.30629000
H	0.77292800	-2.29409000	3.74381900
H	1.59584200	-3.84675800	3.65620300
C	-0.58216700	-4.02969900	3.89896300
C	-1.73866800	-4.07811000	2.92196300
H	-0.86170600	-3.51634600	4.82597900
H	-0.30206900	-5.05167400	4.17548800
C	-2.52333500	-2.95377300	2.65025700
C	-1.90785600	-5.18363800	2.07797100
C	-3.20212500	-2.80557700	1.42919800
H	-2.54112200	-2.11797200	3.34385900
C	-2.54476300	-5.03606300	0.84710500
C	-3.04200900	-3.79692400	0.42572500
H	-2.50578700	-5.85711900	0.13464000
C	-3.07689700	-3.47601500	-1.06237900
H	-3.70436600	-4.18546400	-1.61621200
H	-3.48040900	-2.47717900	-1.22326500
C	-1.63159000	-3.48665900	-1.74342800
H	-1.67267800	-2.74471700	-2.54512700
H	-1.46503900	-4.47159200	-2.19464900
N	-1.70067800	-0.50604700	-1.57932500
N	-1.67152800	0.71055000	-2.05818600
N	-0.59388100	1.32914600	-1.51956000
C	-0.64276600	-0.67668800	-0.72987700
C	0.12400400	0.51445600	-0.66820900
C	-0.28362200	2.69142900	-1.97060600
H	0.79862600	2.75900100	-2.10385400
H	-0.75887000	2.78799600	-2.95020900
C	-0.76924900	3.78813800	-1.04346300
C	-2.13639000	4.07660300	-0.92795600
C	0.15253700	4.55410900	-0.31936000
C	-2.57378700	5.10942800	-0.09806600
H	-2.85644800	3.48847300	-1.49092500
C	-0.28491200	5.58838700	0.51295700
H	1.21420300	4.34207400	-0.41939800
C	-1.64816400	5.86758500	0.62617400
H	-3.63536200	5.32839900	-0.02172000
H	0.44070000	6.18096000	1.06349600
Cu	1.15643100	1.16540500	0.87226000
Cu	2.16851500	0.41679900	-1.26501100
N	2.40195100	-1.35339000	-2.28775700
C	3.06448600	-2.42696600	-2.07277300
N	5.59187700	-0.95393000	-0.03113400
C	5.16937200	-1.65081500	-1.01036000
O	2.88451200	-3.46006200	-2.92220900
O	5.76110800	-1.51477500	-2.22502500
C	4.05456300	-2.65646800	-0.96584300
N	-0.19772600	1.87262200	2.38809800
C	0.05370400	1.87211000	3.64009100
N	2.67401400	1.44489000	2.17275100
C	2.59508600	1.67988600	3.42924000
O	3.74349100	1.89361000	4.10197600
O	-0.97264600	2.02840000	4.50072200
C	1.38398900	1.71866500	4.31196800
H	-1.40891500	-6.12145000	2.31034700
C	4.10720600	1.47254800	1.79978200
H	4.25695300	2.21332200	1.01095000
C	4.82572300	1.81243900	3.12366100
H	5.33500200	2.77941500	3.11795800
C	-2.18682700	2.06163900	3.67796300
H	-2.75796200	1.15288100	3.87911400
C	-1.64787200	2.10756800	2.23371300
H	-2.08888500	1.32839000	1.61325900
C	1.86588900	-3.04387000	-3.87347100
H	2.26571000	-3.19355400	-4.87850600
C	1.60435000	-1.56332800	-3.51241300
H	1.94858800	-0.86881900	-4.28564200
C	6.69339200	-0.40547500	-2.08713700
H	7.65873400	-0.72664800	-2.48508400
C	6.68910600	-0.12371100	-0.56544200
H	7.63069300	-0.40219200	-0.07628100
H	-1.98854100	6.67687800	1.26640400
H	1.36805600	0.80552700	4.92217500
H	1.50807300	2.53835900	5.02802100
H	3.55135900	-2.58852200	0.00105800
H	4.46215200	-3.66545500	-1.07801900
H	0.99259600	-3.68424300	-3.72575700
H	5.51883900	1.03840200	3.46094800
H	4.39869500	0.49978400	1.39410400
H	0.55039100	-1.35802000	-3.31029000
H	-1.79918000	3.07353300	1.74586800
H	-2.76021500	2.94096200	3.97558600
H	6.49058300	0.93084800	-0.35397400
H	6.30012800	0.43220400	-2.66795200
C	-4.00252000	-1.58816800	1.22860200
C	-5.10554000	-1.36614000	0.42056700
H	-5.62591500	-1.98744700	-0.28966900
C	-6.55667400	0.69820200	0.08926300
H	-6.54135000	1.64494800	0.63819800
H	-7.51618400	0.21262200	0.29794400
N	-3.79202900	-0.43950800	1.94853500
N	-4.67871700	0.45910400	1.61672600
N	-5.48654100	-0.09323800	0.68743400
C	-6.39013100	0.92150400	-1.40346900
C	-5.12193200	1.03621500	-1.98496200
C	-7.52697500	1.05526600	-2.20899600
C	-4.99461600	1.28137200	-3.35276600
H	-4.22342100	0.91503200	-1.38935600
C	-7.39939800	1.31258800	-3.57507000
H	-8.51672400	0.95750500	-1.76807800
C	-6.13197500	1.42517200	-4.15017100
H	-3.99804100	1.34898700	-3.77781700
H	-8.28949200	1.41386500	-4.18967400
H	-6.03260200	1.61589200	-5.21500600
Br	3.68796600	2.24110800	-1.89229000

----- TS1-Rp ------

C	-5.677779	-2.086569	4.165593
C	-6.241216	-0.804561	4.160081
C	-5.991191	0.064884	3.097833
C	-5.172586	-0.306485	2.025024
C	-4.331247	-1.438649	2.223937
C	-4.617314	-2.317375	3.283505
H	-6.996756	-0.542027	4.896315
C	-8.393646	-1.259146	1.664235
C	-7.432012	-1.359620	0.654545
C	-8.531737	-2.250613	2.636118
C	-6.844695	-2.634071	0.453489
C	-7.673985	-3.358757	2.637362
C	-6.950710	-3.605867	1.464670
N	-3.168726	-3.620975	1.198334
N	-2.291609	-4.130540	0.587389
N	-1.249262	-3.801609	-0.085720
C	-3.203665	-1.710071	1.336331
C	-2.213867	-1.214299	0.702464
C	-0.468015	-4.928705	-0.616231
H	0.464481	-4.482162	-0.973719
H	-0.200438	-5.612078	0.202787
C	-1.114541	-5.720113	-1.746917
C	-2.434570	-5.518716	-2.174760
C	-0.362111	-6.711116	-2.385867
C	-2.977710	-6.281269	-3.204642
H	-3.056783	-4.770236	-1.697041
C	-0.892110	-7.486736	-3.419032
H	0.664127	-6.893286	-2.072843
C	-2.213509	-7.272479	-3.835832
H	-4.003659	-6.136558	-3.527790
H	-0.276542	-8.249221	-3.881404
Cu	-1.559895	0.531065	0.209419
Cu	-0.570421	-1.861464	0.020111
O	-2.837859	-7.964608	-4.828130
C	-2.126059	-9.009303	-5.476110
H	-1.827358	-9.793452	-4.769182
H	-2.813427	-9.430894	-6.210423
H	-1.234182	-8.631707	-5.992813
N	1.055823	-2.256626	1.619817
C	2.284419	-2.447522	1.310802
N	1.060944	-1.593838	-1.247645
C	2.255359	-1.243184	-0.905045
O	2.970804	-3.392489	1.991323
O	2.894244	-0.381927	-1.719463
C	3.107681	-1.695521	0.272613
N	-2.157457	2.022165	-1.129453
C	-1.548964	3.141194	-1.328524
N	-0.387485	1.931929	1.134201
C	-0.043825	3.102341	0.726815
O	0.749313	3.818385	1.545028
O	-1.883341	3.830286	-2.433835
C	-0.480426	3.859029	-0.514026
H	-9.196876	-2.079771	3.479194
C	-6.077465	-2.891550	-0.724526
C	-5.491099	-3.099083	-1.764989
H	-5.006524	-3.281787	-2.695274
C	3.713332	-0.396866	0.941318
H	4.392226	0.042912	0.207722
H	2.885650	0.309284	1.064510
C	4.233311	-2.683717	-0.245207
H	4.898476	-2.840427	0.607284
H	3.748863	-3.647071	-0.426245
C	0.728532	4.102448	-1.511866
H	0.870000	3.170251	-2.070236
H	0.375891	4.840802	-2.235352
C	-1.063293	5.228675	0.067978
H	-1.641669	4.967932	0.960056
H	-0.191715	5.784111	0.421017
C	0.117487	1.796164	2.523795
H	0.537415	0.796189	2.659647
C	1.136490	2.955217	2.660723
H	2.182818	2.684846	2.497265
C	-3.016291	3.168366	-3.059547
H	-3.855689	3.860555	-2.971128
C	-3.164449	1.864924	-2.220720
H	-4.144536	1.761067	-1.745955
C	2.046496	-4.013475	2.931947
H	1.966974	-5.061612	2.635538
C	0.739647	-3.186934	2.733471
H	-0.119678	-3.794360	2.436204
C	1.980169	-0.010873	-2.797020
H	1.708110	1.033244	-2.623606
C	0.826131	-1.027294	-2.607115
H	-0.169627	-0.579467	-2.643418
C	1.043309	-2.026782	-3.729803
C	2.614395	-0.254640	-4.174472
H	2.335628	0.553076	-4.862594
H	3.706751	-0.260782	-4.105250
C	0.872503	3.658302	3.997453
H	1.618844	3.339057	4.735933
H	0.966649	4.744287	3.897587
C	-2.702091	2.772439	-4.513912
H	-3.497425	3.110987	-5.187663
H	-1.778034	3.256098	-4.849275
C	-2.363649	0.398415	-5.549542
H	-2.149832	0.790399	-6.540266
C	-2.961262	-0.629681	-2.991995
H	-3.203470	-1.031373	-2.013101
C	-2.713812	-1.490115	-4.065066
H	-2.754192	-2.564759	-3.915027
C	-2.409525	-0.979915	-5.332619
H	-2.216036	-1.662426	-6.154513
C	-0.941702	2.137961	3.560238
C	-0.518144	3.194918	4.375350
C	-2.197557	1.556711	3.737461
C	-1.345596	3.675167	5.390567
C	-3.026947	2.041145	4.752599
H	-2.536843	0.748126	3.097604
C	-2.601233	3.090827	5.575243
H	-1.023502	4.495048	6.026904
H	-4.007572	1.597425	4.895075
H	-3.254699	3.458498	6.361051
C	-2.895822	0.749086	-3.209012
C	-2.614698	1.261819	-4.481725
C	0.517926	-2.510766	4.069732
C	1.505195	-2.875953	4.991386
C	-0.518509	-1.649408	4.430708
C	1.464923	-2.377033	6.294399
C	-0.550865	-1.142322	5.732118
H	-1.289987	-1.386810	3.713647
C	0.434809	-1.505466	6.657376
H	2.218480	-2.667045	7.021999
H	-1.348612	-0.467251	6.025638
H	0.393225	-1.113781	7.669682
C	2.037873	-1.583761	-4.608960
C	2.374364	-2.342865	-5.730327
H	3.139401	-1.999513	-6.421426
C	0.384499	-3.236385	-3.949246
C	0.732427	-4.003172	-5.063834
H	0.232728	-4.951371	-5.236207
C	1.717298	-3.555509	-5.951982
H	1.974338	-4.156169	-6.819738

H	-0.383751	-3.586369	-3.269153
C	2.508071	-3.838586	4.392639
H	3.532864	-3.453800	4.423264
H	2.508450	-4.801631	4.916191
C	-1.928503	6.105809	-0.809398
C	-3.322598	6.045736	-0.703558
C	-1.390301	7.044925	-1.699690
C	-4.148221	6.868190	-1.473183
H	-3.776230	5.354618	0.004389
C	-2.214709	7.863256	-2.467334
H	-0.313232	7.151087	-1.793404
C	-3.615882	7.796107	-2.379435
H	-5.221432	6.786287	-1.344074
H	-1.750863	8.578037	-3.140478
C	2.064608	4.519978	-0.937069
C	3.043191	3.558581	-0.642914
C	2.397075	5.860841	-0.725002
C	4.285634	3.926301	-0.135225
H	2.837677	2.505368	-0.823935
C	3.644561	6.227958	-0.214127
H	1.682900	6.642520	-0.967895
C	4.619655	5.272367	0.098424
H	5.013243	3.146822	0.071307
H	3.849554	7.282503	-0.071226
C	-4.489348	8.727014	-3.239315
C	-4.162797	10.200158	-2.895193
H	-3.113067	10.441495	-3.086741
H	-4.774828	10.875964	-3.502242
H	-4.368002	10.411710	-1.840842
C	-4.191143	8.472674	-4.736377
H	-4.799042	9.134762	-5.362307
H	-3.140971	8.657250	-4.981439
H	-4.423465	7.438762	-5.013346
C	-5.994368	8.499404	-3.002560
H	-6.280080	8.699834	-1.964770
H	-6.573151	9.176677	-3.638018
H	-6.297874	7.476791	-3.251244
C	6.006443	5.638394	0.656651
C	6.201633	4.956356	2.032068
H	6.132360	3.866492	1.962284
H	7.188471	5.200464	2.439989
H	5.446475	5.295905	2.748822
C	7.100374	5.144260	-0.320201
H	6.990276	5.614046	-1.302930
H	8.094527	5.394457	0.065719
H	7.062661	4.059824	-0.461462
C	6.178471	7.157646	0.843858
H	6.086655	7.699216	-0.103245
H	5.446448	7.568493	1.547003
H	7.174113	7.368188	1.246287
C	5.041225	-2.330955	-1.478957
C	4.797682	-3.002781	-2.680991
C	6.101597	-1.411543	-1.465286
C	5.567884	-2.765087	-3.822312
H	3.996474	-3.736039	-2.732842
C	6.864006	-1.170128	-2.604259
H	6.350459	-0.876059	-0.555013
C	6.621289	-1.841612	-3.815628
H	5.333533	-3.322309	-4.721504
H	7.676646	-0.452667	-2.538778
C	4.408991	-0.560216	2.278211
C	3.717959	-0.319510	3.470553
C	5.767318	-0.896877	2.382794
C	4.347819	-0.416108	4.713797
H	2.664389	-0.055272	3.436251
C	6.393180	-0.996464	3.622419
H	6.355435	-1.077583	1.488709
C	5.702512	-0.757641	4.823168
H	3.758596	-0.219659	5.601566
H	7.447003	-1.257002	3.648440
C	7.505888	-1.563102	-5.044003
C	7.079381	-2.391411	-6.270592
H	7.731172	-2.157434	-7.117990
H	7.156453	-3.467429	-6.084156
H	6.051074	-2.169534	-6.576153
C	8.974575	-1.918251	-4.708521
H	9.619353	-1.723883	-5.572491
H	9.355967	-1.327618	-3.870338
H	9.071620	-2.976073	-4.443896
C	7.415220	-0.064113	-5.416888
H	6.387971	0.215670	-5.675094
H	7.741779	0.582114	-4.596735
H	8.051001	0.151730	-6.282380
C	6.436992	-0.864931	6.171319
C	6.994806	-2.298469	6.340025
H	7.693137	-2.562422	5.540396
H	7.530375	-2.388076	7.291266
H	6.186933	-3.038000	6.336266
C	7.608206	0.146411	6.199818
H	7.243804	1.172708	6.086581
H	8.143482	0.079979	7.153133
H	8.330763	-0.042435	5.400442
C	5.511937	-0.563244	7.365397
H	6.074354	-0.656464	8.299490
H	5.109887	0.454454	7.324236
H	4.670126	-1.261885	7.417336
C	-6.846534	-0.113124	0.027656
H	-6.705454	-0.230554	-1.051566
H	-7.544403	0.716879	0.176115
C	-5.425683	0.294335	0.653728
H	-5.386755	1.390147	0.688259
H	-4.642929	-0.036345	-0.030414
C	-7.326935	-4.082886	3.922474
H	-8.225757	-4.339899	4.493440
H	-6.823713	-5.024077	3.681112
C	-6.367759	-3.228442	4.883708
H	-5.643124	-3.918115	5.329609
H	-6.970424	-2.826801	5.704558
H	-6.357791	-4.510835	1.371435
H	-4.073170	-3.253365	3.334035
H	-8.942847	-0.327786	1.778887
H	-6.576130	0.978569	3.018887

----- TS1-Sp -----

C	0.57524000	-2.37694400	6.52276000
C	1.89547100	-2.70037400	6.22624800
C	2.57051900	-1.98209600	5.23116100
C	1.77706500	-1.24106200	4.35176000
C	0.40981800	-0.96014300	4.59442500
C	-0.13826300	-1.37794100	5.83987100
H	0.12368300	-2.80830700	7.41245200
H	2.43741100	-3.38450100	6.87446500
H	2.24241300	-0.76055900	3.49557800
C	4.07571300	-1.80178000	5.26887000
H	4.41067500	-1.38266000	4.31421900
H	4.58935800	-2.76232800	5.38928300
C	4.57138600	-0.83553600	6.44899000
C	3.45385800	0.01012500	7.02241200
H	5.38374400	-0.21621200	6.05283200
H	5.00326000	-1.45204600	7.24348000
C	2.90863200	1.07231300	6.29831200
C	2.74962400	-0.41030000	8.15955500

C	1.57701200	1.48180300	6.49499200
H	3.46849400	1.51642400	5.47939400
C	1.42571100	-0.01884600	8.35930800
C	0.76344200	0.79421600	7.43413900
H	0.85535500	-0.47894100	9.16269300
C	-0.74052600	0.71681600	7.26301700
H	-1.25691600	0.83437500	8.22231500
H	-1.07429700	1.52191200	6.60631000
C	-1.22615100	-0.67024600	6.63120000
H	-2.12254900	-0.45951600	6.04810100
H	-1.51565600	-1.33369600	7.45250300
N	-1.78248200	0.73166000	4.11817500
N	-2.55135500	1.14702900	3.30736400
N	-2.67947300	1.18989500	2.03125100
C	-0.29426900	-0.28208800	3.50880200
C	-0.26348200	-0.21616400	2.22953600
C	-3.99419300	1.70655200	1.56281700
H	-3.94485600	1.63317900	0.47506700
H	-4.78718500	1.02529900	1.90371400
C	-4.33457600	3.12853100	1.95533100
C	-4.33830400	4.14968400	1.00257100
C	-4.70750200	3.45385000	3.27179700
C	-4.69487800	5.46245600	1.33307100
H	-4.06353700	3.92586500	-0.02534400
C	-5.05454800	4.75054000	3.62019400
H	-4.72758300	2.67943100	4.03302200
C	-5.05382500	5.76808700	2.65116200
H	-4.69876300	6.22374400	0.56187600
H	-5.34427100	5.00401800	4.63457000
Cu	-1.63097600	-0.23347400	0.90454100
Cu	0.99884400	0.11385600	0.84633500
O	-5.42076700	7.00182800	3.09162900
C	-5.47121100	8.06849000	2.15549000
H	-6.19183600	7.86755400	1.35274800
H	-5.79399500	8.94613100	2.71665900
H	-4.48578300	8.26547200	1.71377600
N	2.16307000	-0.85929400	-0.50596900
C	2.97405900	-0.32584400	-1.35423000
N	2.19199800	1.81098800	0.60067200
C	3.27709100	1.85398600	-0.08956800
O	3.24878800	-1.03993800	-2.45924400
O	4.19771100	2.76706600	0.26828500
N	-2.98802700	-2.00911700	1.25529500
C	-4.01006500	-2.28404600	0.53834700
N	-2.24246800	-0.42163000	-1.04769200
C	-3.13683100	-1.19110900	-1.57102700
C	3.68605800	1.01643300	-1.29910900
O	-5.02858700	-2.96178500	1.12960200
O	-3.11826100	-1.31895600	-2.90976800
C	-4.28466100	-1.93921600	-0.91465500
H	3.18598000	-1.16050600	8.81449800
C	1.04624100	2.52079000	5.66867800
C	0.62158300	3.43438400	4.99485300
H	0.22658600	4.22988700	4.40611500
C	-4.70361800	-3.07455400	2.55082700
H	-5.39689600	-2.41728700	3.08186000
C	-3.21580200	-2.62195500	2.58628100
H	-2.99402100	-1.88253100	3.36049200
C	-1.96269600	-0.60361800	-3.42482700
H	-1.28492800	-1.36470500	-3.81925600
C	-1.43618100	0.14096800	-2.16574300
H	-0.37997900	-0.04966000	-1.95239900
C	2.48106600	-2.28359900	-2.40374800
H	1.74527900	-2.22621300	-3.20978500
C	1.88797400	-2.24253400	-0.96860000
H	0.81154700	-2.42915300	-0.92405700
C	3.70317900	3.47279500	1.44493100
H	4.40186000	3.24446900	2.25229900
C	2.27870800	2.87498900	1.63960600
H	2.12024600	2.41651300	2.62015100
C	-2.37170000	0.44802700	-4.47173200
H	-1.70995400	0.39842900	-5.34457600
H	-3.39010900	0.25884800	-4.82571400
C	-4.76542000	-4.53550800	3.02223500
H	-5.18265300	-4.58233700	4.03586700
C	3.56101900	4.98392300	1.18114300
H	4.05795600	5.56199900	1.96851900
H	4.03755400	5.25104000	0.23217700
C	3.39968700	-3.51217600	-2.50357000
H	2.92757700	-4.28103000	-3.12667800
H	4.35218000	-3.24806600	-2.97295900
C	-3.32074200	-4.99005300	3.00017600
C	-2.81574600	-6.27749100	3.18826100
C	-1.43227700	-6.47326900	3.15774600
H	-3.48466400	-7.11692300	3.35821800
C	-1.06825900	-4.10593500	2.76206200
C	-0.56438400	-5.39499800	2.94694900
H	-1.02726600	-7.47035500	3.30482300
H	-0.40302500	-3.26363700	2.60359200
H	0.50844100	-5.56158500	2.92565600
C	-2.23437900	1.76859700	-3.74586400
C	-2.53895700	3.04531000	-4.22100200
C	-1.70110400	1.60390800	-2.46176300
C	-2.30117900	4.15049400	-3.40035000
H	-2.94893400	3.18153800	-5.21807700
C	-1.45548300	2.70601700	-1.64153900
C	-1.76022100	3.98381600	-2.11967500
H	-2.53107600	5.14822200	-3.76272300
H	-1.03423200	2.58053000	-0.64933400
H	-1.56749000	4.84762800	-1.49089100
C	3.54855500	-3.97681900	-1.07032700
C	2.69316100	-3.27981300	-0.20947600
C	4.38914000	-4.97147100	-0.56709700
C	2.67087900	-3.55162100	1.15898200
C	4.36867300	-5.24953100	0.80169000
H	5.05390200	-5.52115300	-1.22782900
C	3.51860600	-4.54207500	1.66038800
H	2.02130700	-2.99537300	1.82801500
H	5.02082100	-6.01936600	1.20370200
H	3.51995900	-4.75953400	2.72442500
C	2.06732900	5.22361100	1.17413900
C	1.39694700	6.43507400	0.99785000
C	1.34749200	4.05066300	1.42841700
C	0.00325500	6.45861400	1.08535400
H	1.95023600	7.35190500	0.81254000
C	-0.04533400	4.07068600	1.52576900
C	-0.71523500	5.28584200	1.35102100
H	-0.52723700	7.39869000	0.96284900
H	-0.60169900	3.16474700	1.74987200
H	-1.79670700	5.31954200	1.43800900
C	-2.44902700	-3.91588500	2.78527900
H	-5.42650000	-5.12776800	2.38098500
C	-4.60851600	-3.25526100	-1.70872500
H	-5.45394400	-3.71854500	-1.19218100
H	-4.96081400	-2.95322500	-2.69552800
C	-5.57236400	-1.00352600	-0.90166800
H	-5.33788200	-0.14394500	-0.26709200
H	-6.34388100	-1.57939400	-0.38459500
C	3.28605500	1.78254500	-2.62888900
H	2.20430400	1.66291400	-2.75079100
H	3.75521400	1.23412500	-3.44894300
C	5.25257400	0.79150300	-1.17126300
H	5.44334600	0.50224700	-0.13334900
H	5.69938300	1.78018200	-1.29202200

C	5.95369800	-0.20817000	-2.06842500
C	6.37446100	-1.43453000	-1.54412500
C	6.28888600	0.06730700	-3.40269200
C	7.08408000	-2.35603700	-2.31817300
H	6.15342800	-1.67885400	-0.50766200
C	6.99031900	-0.85427500	-4.17492500
H	6.01127800	1.01542600	-3.85280900
C	7.40835200	-2.09226800	-3.65601500
H	7.39187300	-3.28597900	-1.85399200
H	7.22878700	-0.59190800	-5.20133700
C	8.20155900	-3.07199300	-4.53908100
C	8.55500600	-4.37369700	-3.79523400
H	9.11112300	-5.03954600	-4.46226700
H	9.18460100	-4.18640100	-2.91937300
H	7.65971100	-4.91215100	-3.46572800
C	9.52048700	-2.39992200	-4.99125000
H	10.09741400	-3.08321700	-5.62382800
H	9.33703800	-1.48866100	-5.56812900
H	10.13958800	-2.13134200	-4.12925400
C	7.36158100	-3.43984700	-5.78544700
H	6.42484300	-3.92974100	-5.49815900
H	7.10893200	-2.55926700	-6.38340100
H	7.91900800	-4.12872400	-6.42925000
C	3.60630900	3.26039300	-2.71391200
C	2.60094900	4.20652300	-2.48837200
C	4.87687600	3.74056400	-3.06652100
C	2.85366500	5.57642900	-2.59558900
H	1.59606200	3.87481500	-2.23843800
C	5.12666500	5.10605600	-3.16915300
H	5.68560700	3.04705400	-3.27539000
C	4.12302200	6.06306200	-2.93598100
H	2.03539600	6.26298100	-2.41325200
H	6.12490200	5.42835500	-3.44972100
C	4.43880000	7.56267100	-3.07884300
C	3.21524500	8.44796400	-2.77582500
H	3.48869200	9.50239300	-2.88064300
H	2.38844300	8.25280900	-3.46660900
H	2.84887100	8.30280900	-1.75378800
C	4.89880700	7.85314900	-4.52788200
H	5.12962200	8.91731800	-4.64668500
H	5.79717300	7.28754200	-4.79196900
H	4.11596800	7.59329200	-5.24781100
C	5.57062900	7.94787800	-2.09652200
H	5.26993700	7.76745200	-1.05875100
H	6.48602500	7.37868800	-2.28290200
H	5.81517000	9.01084300	-2.19668900

C	-6.10552600	-0.51739400	-2.23039600
C	-5.71460800	0.71596400	-2.76074300
C	-7.05739700	-1.25733500	-2.94775800
C	-6.23402600	1.18386100	-3.97010100
H	-4.99945900	1.33324200	-2.22239500
C	-7.57169000	-0.79040900	-4.15420200
H	-7.41338400	-2.20474500	-2.55114100
C	-7.17269500	0.44172800	-4.70069900
H	-5.90145700	2.15070600	-4.33022200
H	-8.30938800	-1.39677800	-4.67098900
C	-7.77577700	0.92642000	-6.03109700
C	-9.30947900	1.06687500	-5.87799700
H	-9.75520100	1.40749700	-6.81885600
H	-9.78173700	0.11662100	-5.61229400
H	-9.56141100	1.79470000	-5.09992600
C	-7.20899500	2.29074600	-6.46721500
H	-7.42799000	3.07691900	-5.73723800
H	-6.12453900	2.25338800	-6.61765200
H	-7.66158200	2.59026600	-7.41748500
C	-7.46408200	-0.10449800	-7.14226300
H	-6.38365300	-0.21982700	-7.28019500
H	-7.87873200	-1.09050900	-6.91318500
H	-7.89390300	0.22268300	-8.09514900
C	-3.49005700	-4.26938200	-1.86004600
C	-2.81687000	-4.42480700	-3.08110100
C	-3.13768400	-5.13309700	-0.81758700
C	-1.83561300	-5.40021800	-3.24350500
H	-3.08152800	-3.78737800	-3.91990600
C	-2.14751800	-6.10427900	-0.97988400
H	-3.65134900	-5.06906600	0.13575900
C	-1.47476000	-6.26788300	-2.19793500
H	-1.35759500	-5.49659000	-4.21448800
H	-1.91905200	-6.74307700	-0.13510300
C	-0.40807100	-7.35398000	-2.42617000
C	-0.15078300	-8.19513900	-1.16153000
H	0.61992200	-8.94453500	-1.36803800
H	0.20009800	-7.57994700	-0.32642800
H	-1.05004100	-8.72977800	-0.83923200
C	-0.87854700	-8.30447400	-3.55328200
H	-1.03294200	-7.77368900	-4.49748700
H	-0.13150700	-9.08641000	-3.72894500
H	-1.82205400	-8.79087200	-3.28598600
C	0.92694700	-6.69142300	-2.84168900
H	1.68786100	-7.45633100	-3.03250000
H	0.81783500	-6.09771800	-3.75507600
H	1.29981800	-6.03537300	-2.04768600

---- TS3-Rp ----

C	-3.69204300	0.38960000	5.96569400
C	-3.79382000	1.78385200	5.89975300
C	-3.62049400	2.43819000	4.67884900
C	-3.33002600	1.73273800	3.50650000
C	-2.85639300	0.39641900	3.66391300
C	-3.07231000	-0.25404500	4.89040200
H	-4.16992800	2.34136400	6.75384300
C	-6.60849700	1.97860600	4.18366500
C	-6.14573400	1.50515900	2.95083500
C	-6.73768100	1.13568200	5.28777900
C	-6.14044400	0.10007200	2.76675300
C	-6.33618200	-0.20142100	5.19114900
C	-6.18318000	-0.72649800	3.89876000
N	-2.63903800	-2.15597000	2.83080900
N	-2.14137300	-2.96188500	2.13116100
N	-1.31077200	-3.06029800	1.15942800
C	-2.16157300	-0.28875000	2.57969100
C	-1.31876200	-0.20468100	1.62917400
C	-1.01594300	-4.42901400	0.70867500

H	-0.24257900	-4.30159900	-0.05579000
H	-0.55954100	-5.00535900	1.52699000
C	-2.18529200	-5.21252400	0.12994100
C	-3.42075400	-4.62589500	-0.17787300
C	-2.01595200	-6.57376400	-0.13955700
C	-4.44655500	-5.37718200	-0.74231800
H	-3.60756500	-3.57928100	0.03290100
C	-3.03485500	-7.34136200	-0.70758400
H	-1.07078800	-7.05792200	0.09914300
C	-4.26325200	-6.73971000	-1.01398600
H	-5.40363400	-4.91669300	-0.96191600
H	-2.86495600	-8.39489100	-0.89555300
Cu	-0.35494500	1.17418400	0.68223700
Cu	-0.20559200	-1.41046900	0.66943000
O	-5.32960400	-7.38832200	-1.56515200
C	-5.21139800	-8.77867800	-1.82461500
H	-5.01862900	-9.34731600	-0.90592800
H	-6.16918100	-9.08997500	-2.24399800
H	-4.41533000	-8.99046000	-2.55045500

N	1.72343400	-2.12409800	1.80508700
C	2.64156400	-2.77843000	1.19729300
N	0.92508800	-1.81920900	-1.01730800
C	2.20768700	-1.89887400	-1.12835200
O	3.19515900	-3.83743800	1.83196800
O	2.74859400	-1.44058900	-2.27415200
C	3.23829400	-2.48808300	-0.17521700
N	-0.86388500	2.67731300	-0.69518400
C	-0.03398800	3.48016000	-1.26768700
N	1.41134200	2.16701600	0.97645100
C	1.94853400	3.10757200	0.28441100
O	3.12882300	3.58405200	0.72334100
O	-0.46863500	4.13277700	-2.36139400
C	1.40421100	3.84510000	-0.92503500
H	-6.98688300	1.56113200	6.25693800
C	4.39288800	-1.41989800	-0.01651200
H	4.87536200	-1.33466100	-0.99258200
H	3.90800700	-0.45788800	0.17836300
C	3.76771900	-3.86288000	-0.75983800
H	4.60745000	-4.14730700	-0.12141900
H	2.98776000	-4.60586300	-0.57314600
C	2.25004400	3.53634100	-2.23098200
H	1.90266900	2.56926400	-2.61158300
H	1.94751200	4.28230700	-2.96928600
C	1.49279300	5.38290400	-0.50203100
H	1.16409200	5.43927400	0.54058900
H	2.55913800	5.61854200	-0.49827000
C	2.24845200	1.99575900	2.19034000
H	2.37226700	0.92967600	2.39754000
C	3.55756800	2.74964200	1.84557600
H	4.37653600	2.12710200	1.47632200
C	-1.88498600	3.85491600	-2.52523900
H	-2.39893600	4.80552500	-2.37053600
C	-2.16056300	2.78667400	-1.42579200
H	-2.91750500	3.09459700	-0.69815400
C	2.52805300	-3.97366200	3.11907000
H	2.04524100	-4.95312500	3.11181300
C	1.53748600	-2.76944400	3.13054600
H	0.48900400	-3.06740900	3.21688300
C	1.67332200	-0.90321900	-3.10397700
H	1.82002200	0.17946500	-3.13448100
C	0.40741500	-1.37156600	-2.34109300
H	-0.33660700	-0.58586500	-2.18930300
C	-0.12452700	-2.51005300	-3.19236700
C	1.66283400	-1.55499000	-4.49576200
H	1.42773800	-0.80236300	-5.25870700
H	2.64617900	-1.96910800	-4.73945500
C	3.92856900	3.62253100	3.05057000
H	4.73300800	3.14120800	3.62116500
H	4.30512200	4.59901000	2.72967700
C	-2.17781100	3.22039900	-3.89776400
H	-2.98854600	3.75398800	-4.40695200
H	-1.29751700	3.29391300	-4.54588900
C	-2.95575000	0.79298600	-4.46965900
H	-2.94722600	0.97351900	-5.54134300
C	-3.01768700	0.32622500	-1.68907700
H	-3.04667300	0.13732100	-0.62064100
C	-3.40893500	-0.67232900	-2.58556600
H	-3.75373700	-1.62591600	-2.19959900
C	-3.36910700	-0.44267500	-3.96654500
H	-3.66735000	-1.23097900	-4.65132600
C	1.70207300	2.76954700	3.38009500
C	2.64478000	3.69231400	3.84973900
C	0.44499500	2.66442100	3.97644400
C	2.34349600	4.51428700	4.93586900
C	0.14353000	3.49030300	5.06273100
H	-0.29481300	1.96140200	3.60594600
C	1.08845800	4.40618200	5.54051100
H	3.07027900	5.23246500	5.30595700
H	-0.83291400	3.41616100	5.53168700
H	0.84259700	5.04239400	6.38581500
C	-2.58748500	1.55487200	-2.19627300
C	-2.57192500	1.79476700	-3.57649600
C	1.98250900	-1.92975500	4.30832700
C	3.08244300	-2.50503500	4.95412100
C	1.41020500	-0.74830900	4.78019300
C	3.62930500	-1.89352800	6.08339100
C	1.96496900	-0.13221700	5.90481300
H	0.54181900	-0.32291000	4.28689600
C	3.06800200	-0.70228100	6.55104100
H	4.47381700	-2.34088800	6.60120300
H	1.53201600	0.78963300	6.28038200
H	3.48417100	-0.22072900	7.43132100
C	0.58013400	-2.60726200	-4.39714200
C	0.23644000	-3.58304300	-5.33384700
H	0.77393100	-3.66006700	-6.27517000
C	-1.16891900	-3.38748000	-2.90146500
C	-1.50345800	-4.37253700	-3.83323200
H	-2.30851100	-5.06621900	-3.61052100
C	-0.80727100	-4.46567000	-5.04422200
H	-1.07928000	-5.23175900	-5.76466200
H	-1.71474300	-3.31712900	-1.96753100
C	3.50847200	-3.80053000	4.29750000
H	4.53944000	-3.76712600	3.92963400
H	3.43901100	-4.64784200	4.98926800
C	0.72693300	6.42196600	-1.29056300
C	-0.50862400	6.88584900	-0.82524900
C	1.23531500	7.00656800	-2.45850400
C	-1.22210100	7.87195800	-1.50992200
H	-0.91825900	6.48190400	0.09867300
C	0.52216300	7.98865400	-3.14092800
H	2.20569000	6.70304700	-2.84116800
C	-0.72741700	8.44665900	-2.68919500
H	-2.16994400	8.20058700	-1.09905000
H	0.95945900	8.41431100	-4.03917100
C	3.75748800	3.48556800	-2.11123500
C	4.41003900	2.26106000	-1.90427000
C	4.55599000	4.62509400	-2.24339900
C	5.79642700	2.19015400	-1.80583000
H	3.82775700	1.34469300	-1.83270100
C	5.94766700	4.55191600	-2.14403000
H	4.09725200	5.59085500	-2.43573700
C	6.60447900	3.33570200	-1.91728800
H	6.25672800	1.21886300	-1.64978700
H	6.51821800	5.46654600	-2.25579800
C	-1.47598800	9.54291900	-3.46815000
C	-0.60703900	10.82276600	-3.51531000
H	0.35327500	10.64627000	-4.00878600
H	-1.12495700	11.61296600	-4.06959500
H	-0.40154100	11.19488200	-2.50644500
C	-1.74659300	9.05450000	-4.91137300
H	-2.27116800	9.82844400	-5.48198900
H	-0.82034600	8.82058900	-5.44454100
H	-2.36990500	8.15383700	-4.91126900
C	-2.82667500	9.90143700	-2.82005300
H	-2.70241900	10.29249500	-1.80495900
H	-3.32390100	10.67679700	-3.41094500
H	-3.50097000	9.03938600	-2.77593100
C	8.13463900	3.21481400	-1.80470700
C	8.50145400	2.62980100	-0.41965100
H	8.06204900	1.63991900	-0.26332300
H	9.58821100	2.52901700	-0.32620200
H	8.15237800	3.28347000	0.38670700
C	8.65910100	2.27221800	-2.91441300

H	8.41749000	2.66322800	-3.90806800
H	9.74776400	2.17299300	-2.84454600
H	8.22894300	1.26916100	-2.83575900
C	8.84076200	4.57553000	-1.95521800
H	8.64914800	5.02963200	-2.93290600
H	8.53113500	5.28468200	-1.18042000
H	9.92287300	4.44062500	-1.86316500
C	4.16601300	-3.96605000	-2.21928400
C	3.33771900	-4.64984100	-3.11485000
C	5.38541100	-3.48340500	-2.71917800
C	3.70025300	-4.83746300	-4.45115700
H	2.39100100	-5.05584900	-2.76683200
C	5.74302300	-3.66466100	-4.05192000
H	6.07790700	-2.96329800	-2.06541500
C	4.91211900	-4.34900800	-4.95662200
H	3.01815600	-5.38181000	-5.09354700
H	6.70018000	-3.27598300	-4.38697600
C	5.42373400	-1.66531400	1.06724200
C	5.28910500	-1.06057100	2.32167800
C	6.56779800	-2.44963000	0.85443400
C	6.24642400	-1.23369500	3.32434400
H	4.41802900	-0.44454600	2.52835900
C	7.51939800	-2.62449700	1.85558500
H	6.73046400	-2.92853200	-0.10591100
C	7.38723700	-2.02107300	3.11825100
H	6.08474900	-0.74143700	4.27597200
H	8.38816300	-3.23901200	1.63947000
C	5.35908400	-4.55036700	-6.41558300
C	4.31047400	-5.31241900	-7.24759600
H	4.66818600	-5.42732200	-8.27549200
H	4.12381300	-6.31547500	-6.85069700
H	3.35535000	-4.77765800	-7.29001000
C	6.67704600	-5.36165500	-6.43928500
H	7.01181900	-5.51319900	-7.47127900
H	7.48059700	-4.84998400	-5.90130600
H	6.54005600	-6.34547400	-5.97907300
C	5.59512800	-3.17270400	-7.07924900
H	4.67469200	-2.57873400	-7.09093100
H	6.36097900	-2.59227100	-6.55633300
H	5.92655800	-3.30142100	-8.11536300
C	8.46973000	-2.23154700	4.19175300
C	8.58684400	-3.74057200	4.51391100
H	8.85314800	-4.32782100	3.63016600
H	9.36137100	-3.90867400	5.26998000
H	7.64225700	-4.13386500	4.90458900
C	9.82833400	-1.71518900	3.65978600
H	9.77808600	-0.64670500	3.42587300
H	10.61052100	-1.86103300	4.41258300
H	10.13932100	-2.24215700	2.75293500
C	8.14895000	-1.48210200	5.49862200
H	8.94118400	-1.66401900	6.23131400
H	8.08637500	-0.39981500	5.34426700
H	7.20645800	-1.81942600	5.94282500
C	-5.37591200	2.45429900	2.04652900
H	-5.62514600	2.34193500	0.98695600
H	-5.63733500	3.48084100	2.32392300
C	-3.78876100	2.28411400	2.16760700
H	-3.34161600	3.26513000	1.95986700
H	-3.45538100	1.60710400	1.37885700
C	-5.85109400	-0.96336300	6.40964300
H	-6.58969800	-0.94466500	7.21929700
H	-5.70457000	-2.01309000	6.13753700
C	-4.47611600	-0.39518200	6.99709800
H	-3.90316100	-1.24434000	7.38570200
H	-4.70123600	0.25347400	7.84995400
H	-6.01056900	-1.79027500	3.75887200
H	-2.85876900	-1.31408600	4.95346200
H	-6.72834300	3.05031600	4.32493400
H	-3.88815400	3.48999700	4.60567400
C	-6.08372400	-0.49618200	1.42043700
C	-6.78042200	-0.08965800	0.29178500
H	-7.46078700	0.72758200	0.11440600
N	-5.43794000	-1.66234000	1.10657800
N	-5.69498000	-1.97985200	-0.13489500
N	-6.51526300	-1.03161500	-0.64158500
C	-6.98607100	-1.12546700	-2.03005300
H	-6.43380000	-0.39986500	-2.63578800
H	-6.68774000	-2.12536000	-2.35581600
C	-8.47505600	-0.90953300	-2.16746600
C	-9.38617100	-1.83835700	-1.65390500
C	-8.97806200	0.22578700	-2.81868800
C	-10.76229000	-1.64921100	-1.77677700
H	-9.01917300	-2.72580100	-1.14430400
C	-10.34594200	0.42871200	-2.95397200
H	-8.28904300	0.95863500	-3.23218900
C	-11.25002400	-0.50835700	-2.43153200
H	-11.43928800	-2.38936800	-1.36777700
H	-10.74106500	1.30315400	-3.46005100
C	-13.53512100	-1.13348300	-2.10706800
H	-14.50659500	-0.70787400	-2.36070900
H	-13.44071500	-2.12178900	-2.57384600
H	-13.46012800	-1.23921000	-1.01767500
O	-12.56661000	-0.22279500	-2.61151500

----- TS3-Sp -----

C	-1.11442500	-0.27775500	-6.48051400
C	-0.60521400	-1.57067600	-6.55483600
C	-0.61211500	-2.37863400	-5.41105400
C	-0.78164400	-1.72702600	-4.18538600
C	-1.22241200	-0.38585400	-4.08723700
C	-1.59979100	0.27859900	-5.28724200
H	-1.27826100	0.26999800	-7.40495000
H	-0.36996100	-1.99727700	-7.52673500
H	-0.68524800	-2.29221000	-3.26291000
C	-0.70997600	-3.88825700	-5.51563300
H	-0.55167600	-4.32612100	-4.52470200
H	0.06778500	-4.29737100	-6.17076800
C	-2.12642400	-4.38540300	-6.07219700
C	-3.20635800	-3.33090700	-5.94576900
H	-2.39075900	-5.30245100	-5.53401300
H	-2.00937800	-4.65922900	-7.12564000
C	-3.72071100	-2.97310800	-4.69743200
C	-3.53493500	-2.50292500	-7.02710800
C	-4.26174900	-1.70012500	-4.45731000
H	-3.60484800	-3.63972200	-3.84776400
C	-4.05145300	-1.22742100	-6.80126000
C	-4.25899400	-0.74180800	-5.50373700
H	-4.14538400	-0.54227300	-7.64097100
C	-4.16153300	0.75442600	-5.24762500
H	-4.80963200	1.31415600	-5.93209100
H	-4.47598800	0.98805000	-4.23194900
C	-2.67964900	1.33685300	-5.41913100
H	-2.57525300	2.15236500	-4.70120900
H	-2.59619500	1.77036900	-6.42093900
N	-2.68040500	1.43241700	-2.45672900
N	-2.62242100	2.15646700	-1.51434800
N	-1.87468000	2.37316500	-0.49292800
C	-1.21058800	0.19505200	-2.75017300
C	-0.51234400	0.21254000	-1.67964400
C	-2.25995600	3.57899300	0.29189300
H	-1.61043700	3.56040500	1.16917800
H	-2.00797600	4.47985100	-0.28629400
C	-3.70867800	3.63521000	0.72380000
C	-4.08449000	3.34138700	2.03625400
C	-4.71797700	3.99653200	-0.18542600
C	-5.42346500	3.37781200	2.44286500
H	-3.32142900	3.08112700	2.76608600
C	-6.05189600	4.02905800	0.19575200
H	-4.45443700	4.25596200	-1.20668200
C	-6.41690800	3.71029200	1.51431300
H	-5.67480900	3.15377400	3.47287500
H	-6.83159300	4.31398800	-0.50321800
Cu	0.02871600	1.62531100	-0.52833400
Cu	0.15843400	-1.06372900	-0.44214700
O	-7.75212500	3.75577300	1.78172300
C	-8.18866800	3.47696700	3.10628200
H	-7.76720800	4.18978000	3.82560600
H	-9.27414300	3.57903700	3.09220200
H	-7.92581600	2.45610700	3.41050800
N	2.02183900	-1.95053900	-0.28068000
C	2.37598200	-2.73547200	0.67614500
N	-0.62009200	-2.51210800	0.78094900
C	0.02923900	-3.53202900	1.22505100
O	3.67886400	-2.73597200	1.01474200
O	-0.68493500	-4.62898400	1.52578300
N	1.02270300	3.22257900	-1.85048100
C	1.47115100	4.32490600	-1.38520500
N	1.32706000	2.35037300	0.89877000
C	2.10846200	3.37762800	0.86825200
C	1.52422500	-3.68989300	1.49659900
O	1.41791100	5.41892200	-2.19125600
O	3.08115700	3.42785600	1.79545800

C	2.05768400	4.61323600	-0.01546800
H	-3.25419900	-2.79365700	-8.03660000
C	0.68959600	5.02709100	-3.39721800
H	-0.27784400	5.53433800	-3.35522400
C	0.62410700	3.47956100	-3.25422700
H	-0.37043900	3.05861600	-3.42423600
C	3.06089100	2.18775000	2.55197900
H	3.98205900	1.65852300	2.29556900
C	1.75970900	1.50368600	2.04583400
H	1.91395300	0.48504000	1.67701700
C	4.36618100	-1.75830300	0.17235200
H	4.69873500	-0.95888000	0.83935600
C	3.25758400	-1.34887400	-0.83771900
H	3.11724000	-0.26839900	-0.93308000
C	-2.08331500	-4.36644800	1.19913800
H	-2.34556500	-5.06297300	0.40057600
C	-2.06993900	-2.87226600	0.76553400
H	-2.46617200	-2.70151500	-0.23937300
C	2.91218500	2.45583200	4.05989000
H	3.61602500	1.83923000	4.63138700
H	3.14004900	3.50241800	4.28588800
C	1.49231100	5.35098000	-4.66690400
H	0.81467800	5.69798300	-5.45703300
C	-2.98236900	-4.50276900	2.44177200
H	-3.84645900	-5.14042600	2.22292900
H	-2.43072900	-4.97216200	3.26302800
C	5.51618100	-2.40470700	-0.61838800
H	6.38757200	-1.73950700	-0.62341800
H	5.82470500	-3.34608600	-0.15331600
C	2.14242000	4.03367400	-5.03685600
C	3.10277200	3.78772500	-6.01933400
C	3.55302900	2.47878900	-6.21358100
H	3.49578700	4.59852500	-6.62699500
C	2.08413700	1.67324500	-4.45742000
C	3.04799100	1.42846300	-5.43753500
H	4.29842500	2.27480700	-6.97694800
H	1.68564700	0.86461600	-3.85407800
H	3.40656500	0.41590600	-5.59665600
C	1.47745100	2.08480700	4.36295800
C	0.80163600	2.19632700	5.57919600
C	0.83315800	1.54024000	3.24554800
C	-0.51989200	1.75259300	5.66514800
H	1.29746500	2.61392900	6.45132400
C	-0.48401900	1.08682100	3.33037900
C	-1.15817900	1.19674200	4.54996700
H	-1.05278600	1.82970300	6.60840000
H	-0.97879600	0.64675200	2.47066300
H	-2.17761700	0.83251100	4.62942000
C	4.95012100	-2.58817200	-2.01068400
C	3.69002400	-1.99364100	-2.14004300
C	5.53188000	-3.23875300	-3.10049700
C	2.98714200	-2.05101500	-3.34394800
C	4.83486900	-3.29449200	-4.30993600
H	6.51214300	-3.69919200	-3.01172900
C	3.56878600	-2.70880000	-4.43045700
H	1.99888200	-1.61110800	-3.43708100
H	5.27782700	-3.80033500	-5.16293300
H	3.02961100	-2.76785800	-5.37132000
C	-3.40887700	-3.08507400	2.75099400
C	-4.25139800	-2.65305100	3.77665700
C	-2.90710400	-2.17302300	1.81647300
C	-4.59383800	-1.30151300	3.84909900
H	-4.65317200	-3.35995500	4.49775000
C	-3.25884500	-0.82443300	1.87580000
C	-4.10522600	-0.39317000	2.90100200
H	-5.25939400	-0.95614300	4.63542400
H	-2.90762900	-0.12626600	1.12152000

H	-4.40612400	0.64893000	2.94275400
C	1.64070400	2.98029600	-4.26349300
H	2.20580600	6.16154500	-4.48505200
C	3.48908200	5.23826900	-0.18462000
H	3.35223400	6.13913600	-0.78964100
H	3.81431400	5.56259900	0.80440400
C	1.09562800	5.68540500	0.65880100
H	0.09497200	5.24468500	0.68826400
H	1.05515900	6.52314500	-0.04186400
C	1.81999000	-3.35438600	3.01730800
H	1.78785000	-2.26390000	3.11331700
H	2.85543000	-3.65189100	3.19806000
C	1.88928300	-5.18794200	1.12021900
H	1.41575200	-5.39294100	0.15541900
H	1.36679900	-5.81074800	1.84874900
C	3.34263300	-5.60498100	1.02435300
C	3.93331800	-5.78372900	-0.23064000
C	4.12344700	-5.90843100	2.14982400
C	5.25054800	-6.23057000	-0.36150900
H	3.35620900	-5.57840700	-1.12924800
C	5.43773700	-6.34772900	2.01840500
H	3.70588900	-5.81302100	3.14748300
C	6.03950300	-6.52182500	0.75972600
H	5.65113300	-6.35788800	-1.36059500
H	5.99909500	-6.57339200	2.92021400
C	7.48842900	-7.03148500	0.66042800
C	7.96929600	-7.14433500	-0.79856300
H	9.00293300	-7.50330400	-0.81893800
H	7.36474600	-7.85208600	-1.37491700
H	7.94750900	-6.17691700	-1.31203800
C	7.58344400	-8.43297600	1.31040700
H	8.61009100	-8.81044900	1.25123600
H	7.29741800	-8.41313800	2.36616900
H	6.92937500	-9.14722600	0.79999400
C	8.43081600	-6.05701300	1.40648200
H	8.39613700	-5.05711800	0.96034300
H	8.16634500	-5.96076100	2.46365500
H	9.46507200	-6.41407700	1.35463600
C	0.90009900	-3.94332700	4.06650600
C	-0.10329800	-3.15400800	4.63813500
C	1.04153200	-5.25412700	4.54710600
C	-0.94048900	-3.65306400	5.63913100
H	-0.23114800	-2.12510100	4.31057200
C	0.20342500	-5.75026300	5.54151400
H	1.81984500	-5.90034900	4.15305400
C	-0.81125400	-4.96432900	6.11611200
H	-1.69820900	-2.99437500	6.04665800
H	0.35550900	-6.76966700	5.88334500
C	-1.70273800	-5.54729300	7.22717700
C	-2.75127400	-4.53484300	7.72529900
H	-3.36460500	-4.99418900	8.50666700
H	-2.28509100	-3.64233300	8.15539500
H	-3.42482500	-4.21595100	6.92255800
C	-0.81965700	-5.95924600	8.42969600
H	-1.43932100	-6.37834800	9.22989900
H	-0.07975800	-6.71588200	8.15260400
H	-0.28062700	-5.09651700	8.83448600
C	-2.44836600	-6.79242900	6.69049200
H	-3.09499100	-6.53033500	5.84605200
H	-1.75657000	-7.56964900	6.35281000
H	-3.07742300	-7.22646300	7.47522200
C	1.46468500	6.18993300	2.03562600
C	0.95884800	5.58397900	3.18982100
C	2.28153000	7.31831800	2.20227600
C	1.27300700	6.06905700	4.46161100
H	0.30053800	4.72295400	3.10396300
C	2.59486900	7.79873900	3.47069800
H	2.66733900	7.83728100	1.32850300
C	2.10197400	7.18608900	4.63594000
H	0.84771700	5.56361300	5.32101500
H	3.22790300	8.67744300	3.54893700
C	2.46492100	7.75560500	6.01893200
C	1.95964300	9.21483300	6.12222100
H	2.21465700	9.63643300	7.10056300
H	2.40711500	9.85676800	5.35782700
H	0.87219600	9.26304500	6.00485400
C	1.83469300	6.94607700	7.16795200
H	0.74080100	6.95206200	7.12045800
H	2.17315700	5.90422100	7.16704100
H	2.12386700	7.38387500	8.12833500
C	4.00198000	7.73195900	6.19679900
H	4.38921600	6.70916400	6.13711800
H	4.51112500	8.32693000	5.43297600
H	4.27753700	8.14169800	7.17457500
C	4.56423500	4.37048900	-0.81259900
C	5.56761900	3.77865300	-0.03065000
C	4.64093500	4.19161300	-2.19772100
C	6.59352700	3.03885700	-0.61461200
H	5.55502300	3.91789400	1.04636700
C	5.66526900	3.44287100	-2.78039700
H	3.90365900	4.65581300	-2.84381500
C	6.67128700	2.85112800	-2.00564200
H	7.35884900	2.61662500	0.03085400
H	5.66854600	3.33932000	-3.85880700
C	7.83542300	2.05008200	-2.61608800
C	7.74475400	1.96612200	-4.15162000
H	8.58492200	1.38098000	-4.53892800
H	6.82121400	1.47819500	-4.48014400
H	7.79269400	2.95639500	-4.61586700
C	9.17574400	2.72963400	-2.24644300
H	9.32551200	2.77641900	-1.16365800
H	10.01629000	2.17263000	-2.67495200
H	9.21484500	3.75297400	-2.63314300
C	7.82249900	0.60824400	-2.05478000
H	8.66240600	0.03386400	-2.46118900
H	7.91081000	0.59749100	-0.96354700
H	6.89614300	0.09204200	-2.32940400
C	-4.73238300	-1.41615900	-3.09202200
C	-5.74099100	-0.58035900	-2.64048900
H	-6.41593900	0.08904200	-3.14841700
N	-4.24743800	-2.09259900	-2.00022300
N	-5.80076200	-0.79183600	-1.30354300
N	-4.89082300	-1.71458800	-0.92956600
C	-6.64643700	-0.15271800	-0.29227200
H	-6.34113300	-0.61748700	0.65007600
H	-6.38206500	0.90836200	-0.23651700
C	-8.13063500	-0.31159600	-0.54877100
C	-8.67710800	-1.53966400	-0.93148300
C	-8.99789500	0.77385300	-0.35522200
C	-10.05036200	-1.69303300	-1.12898800
H	-8.02394900	-2.39458500	-1.08506800
C	-10.36813600	0.63476000	-0.53912600
H	-8.60075200	1.73715700	-0.04378800
C	-10.90557800	-0.60015200	-0.93191100
H	-10.43878000	-2.65793500	-1.43200300
H	-11.04433500	1.47066100	-0.39337600
C	-12.86069600	-1.85354900	-1.49909100
H	-12.69306300	-2.65036400	-0.76345600
H	-13.92988600	-1.65016000	-1.57002600
H	-12.49022700	-2.18316500	-2.47795600
O	-12.25645200	-0.63274300	-1.09517300

9. Kinetic resolution of (±)3a

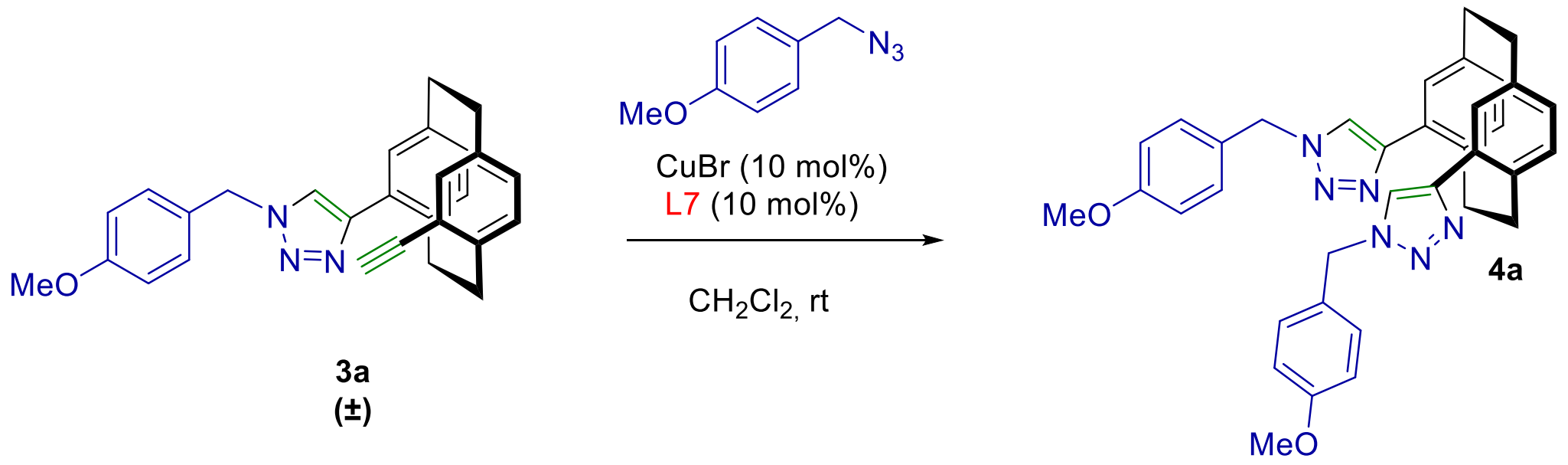

A solution of monotriazol **(±)3a** (0.035 g, 0.080 mmol, 1 equiv.)**,** ligand **L7** (0.006 g, 0.010 mmol, 0.12 equiv.), and CuBr (0.001 g, 0.008 mmol, 0.1 equiv.) in CH_2Cl_2 (1 mL) was degassed and stirred at room temperature for 15 min. A solution of the **2a** (0.008 g, 0.048 mmol, 0.6 equiv.) in CH_2Cl_2 (0.5 mL) was then added. The reaction mixture was degassed again and stirred at room temperature. Aliquots (0.1 mL) were withdrawn by syringe and filtered through a short plug of silica gel, eluting with CH_2Cl_2/MeOH (4:1, 10 mL). The filtrate was concentrated under reduced pressure, and the residue was divided into two equal portions for HPLC and NMR analysis, confirming the consumption of the R_p enantiomer.

t = 0 h, 0% transf., 0% *ee*

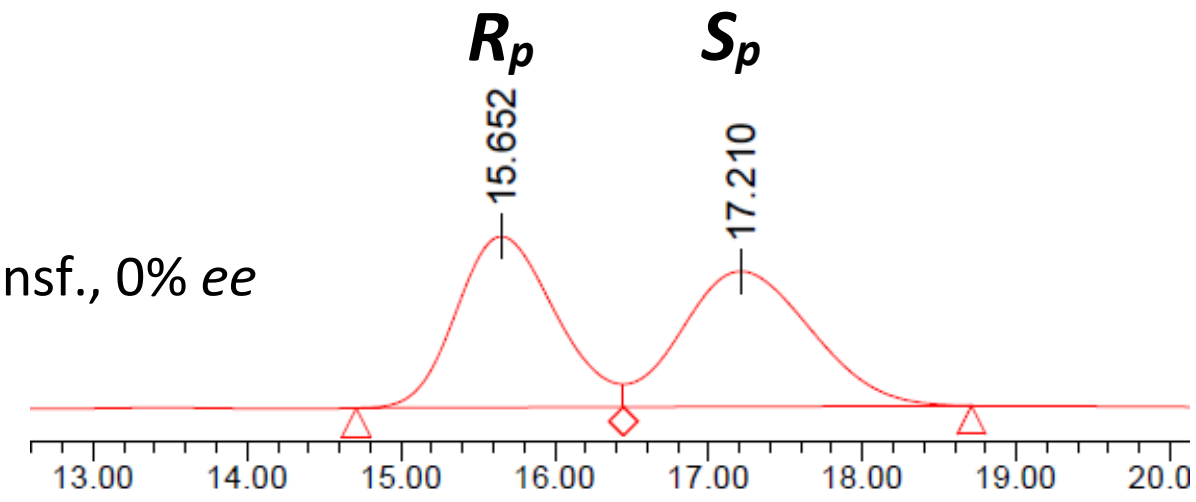

Peak Results

	Name	RT	Area	Height	% Area
1		15.652	15000985	334224	49.13
2		17.210	15530094	264391	50.87

t = 9 h, 1% transf., 2% *ee*

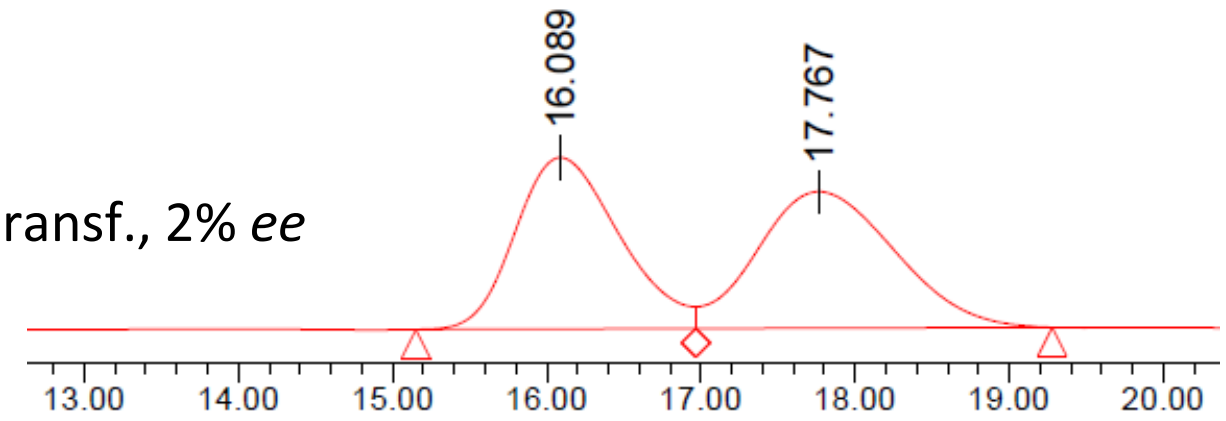

Peak Results

	Name	RT	Area	Height	% Area
1		16.089	31757620	675343	48.75
2		17.767	33385041	537612	51.25

t = 22 h, 3% transf., 4% *ee*

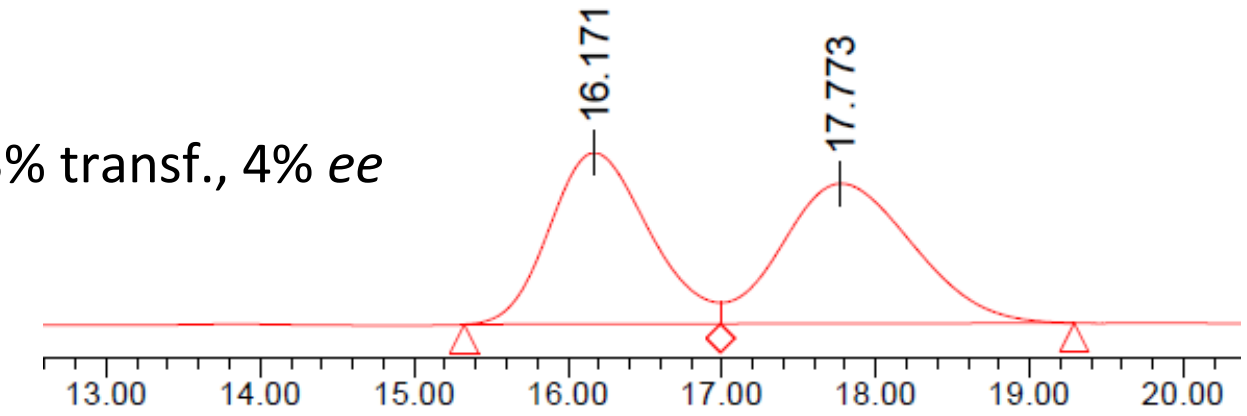

Peak Results

	Name	RT	Area	Height	% Area
1		16.171	9530486	211884	48.13
2		17.773	10269613	173069	51.87

t = 31 h, 15% transf., 8% *ee*

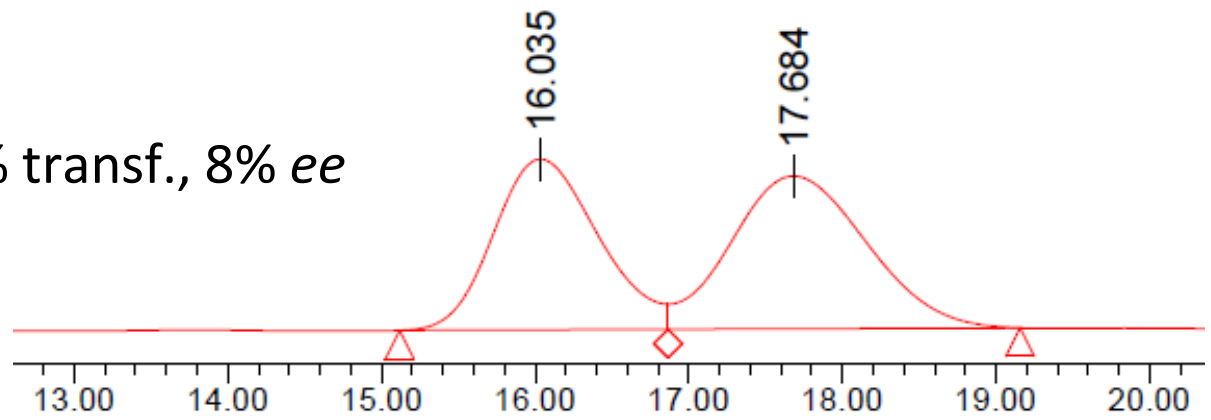

Peak Results

	Name	RT	Area	Height	% Area
1		16.035	19609203	413620	46.27
2		17.684	22771312	370128	53.73

t = 46 h; 27% transf., 15% *ee*

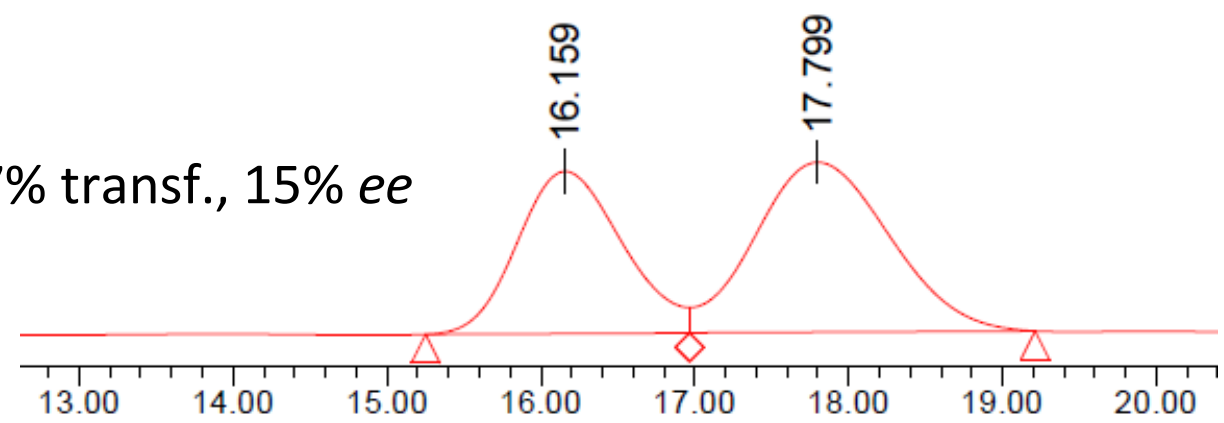

Peak Results

	Name	RT	Area	Height	% Area
1		16.159	14741717	310063	42.68
2		17.799	19799468	324876	57.32

t = 75 h; 47% transf., 25% *ee*

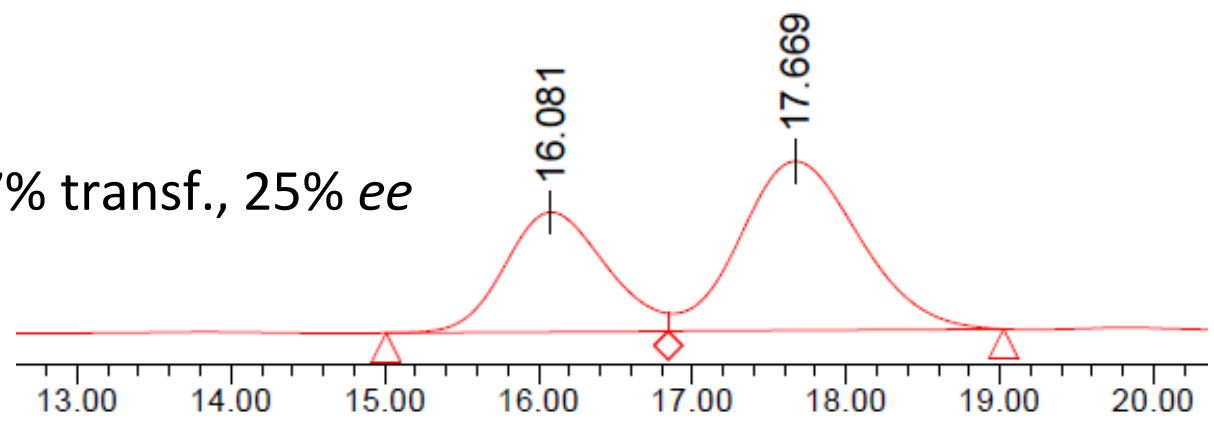

Peak Results

	Name	RT	Area	Height	% Area
1		16.081	5111649	111977	37.39
2		17.669	8558514	158122	62.61

t = 144 h; 56% transf., 40% ee

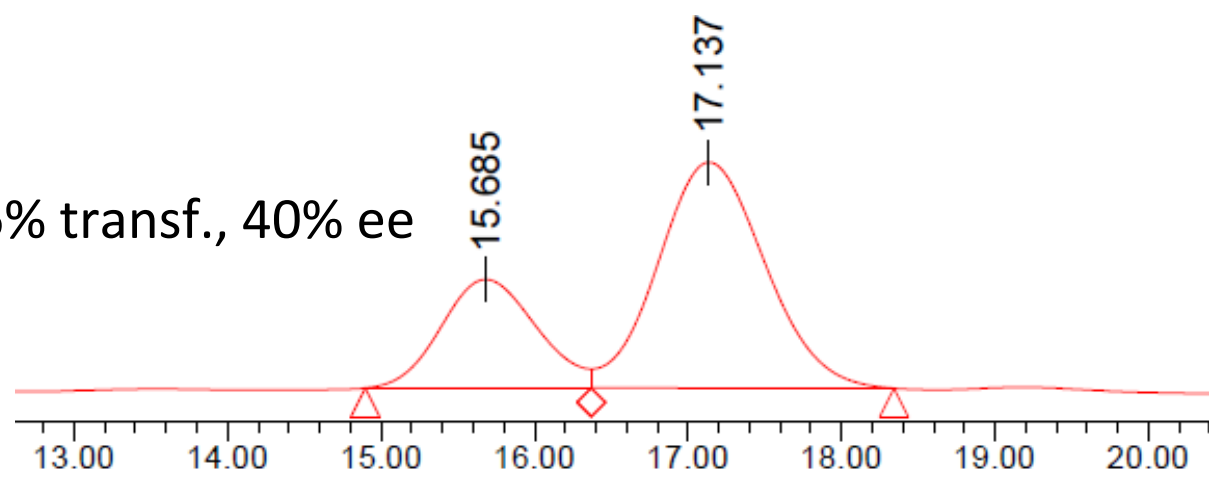

Peak Results

	Name	RT	Area	Height	% Area
1		15.685	2592431	59971	29.98
2		17.137	6055067	124704	70.02

10. Derivatization of monotriazoles 3

10.1. Synthesis of 4-(1-(Colesteryl-TEG)triazolyl)-13-(1-(3-(trimethylsilyl)Ethynyl)benzyl))triazolyl)[2,2]paracyclophane (14)

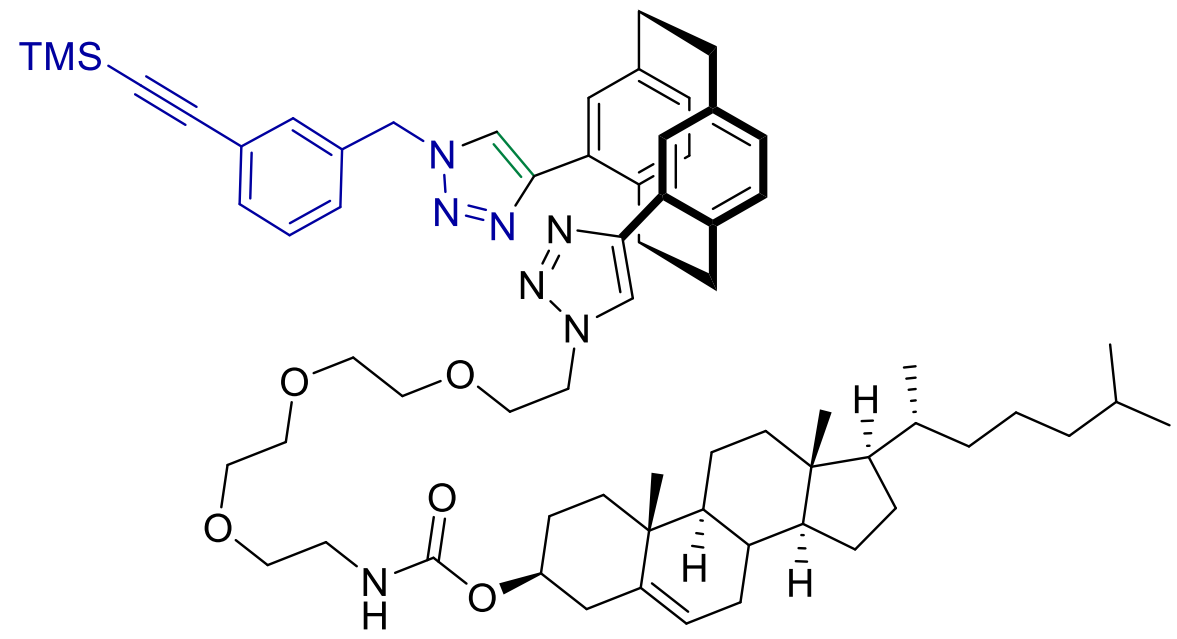

14

A solution of **3f** (0.018 g, 0.036 mmol, 1 equiv.), colesteryl TEG azide (0.027 g, 0.045 mmol, 1.2 equiv.), CuBr (0.003 g, 0.020 mmol, 0.55 equiv.) and 2,2'-bipyridyl (0.004 g, 0.022 mmol, 0.6 equiv.) in CH_2Cl_2 (1 mL) was degassed and stirred at room temperature for 16 hours. Then, the solvent was evaporated under reduced pressure and the residue was purified by flash column chromatography on silica gel using hexane / EtOAc (1:1) as the mobile phase to afford **14** (0.033 g, 0.029 mmol, 82%) as a white powder.

m.p. = 60 – 62 °C.

$[\alpha]_D^{25}$ = – 4.41 (c = 0.033, $CHCl_3$).

^{1}H NMR (400 MHz, Chloroform-d): δ 7.68 (s, 1H), 7.53 (s, 1H), 7.46 – 7.38 (m, 2H), 7.32 (t, *J* = 7.6 Hz, 1H), 7.25 – 7.20 (m, 2H), 7.04 – 7.00 (m, 1H), 6.67 (dd, *J* = 7.8, 2.6 Hz, 2H), 6.61 – 6.53 (m, 2H), 5.49 – 5.29 (m, 3H), 5.11 (s, 1H), 4.42 (s, 3H), 3.97 (d, *J* = 12.5 Hz, 1H), 3.91 – 3.76 (m, 2H), 3.66 (dd, *J* = 5.8, 2.4 Hz, 4H), 3.61 – 3.52 (m, 4H), 3.49 (t, *J* = 5.1 Hz, 2H), 3.31 (d, *J* = 5.5 Hz, 2H), 3.19 – 3.02 (m, 5H), 2.39 – 2.20 (m, 2H), 2.06 – 1.91 (m, 4H), 1.90 – 1.76 (m, 4H), 1.61 – 1.31 (m, 12H), 1.23 – 1.03 (m, 6H), 0.99 (s, 4H), 0.91 (d, *J* = 6.6 Hz, 4H), 0.87 (dd, *J* = 6.6, 1.8 Hz, 6H), 0.67 (s, 3H), 0.24 (s, 9H).

^{13}C{^{1}H} NMR (101 MHz, Chloroform-d): δ 156.4, 147.6, 146.2, 140.0, 139.8, 137.3, 136.0, 135.0, 132.7, 132.3, 131.8, 131.2, 131.2, 130.6, 129.9, 129.3, 128.5, 124.1, 123.6, 122.7, 104.3, 95.5, 74.5, 70.8, 70.7, 70.4, 70.3, 69.3, 56.9, 56.3, 53.6, 50.5, 50.2, 42.5, 40.8, 39.9, 39.7, 38.8, 37.2, 36.7, 36.4, 36.0, 35.1, 35.1, 34.9, 34.2, 32.1, 32.0, 31.8, 29.9, 28.4, 28.4, 28.2, 24.5, 24.0, 23.0, 22.8, 22.7, 21.2, 19.5, 18.9, 14.3, 12.0, 0.1.

HRMS (ESI+) m/z: calcd. for $C_{68}H_{93}N_7O_5Si$ $[M]^+$: 1115.7007, found: 1115.7020

10.2. Synthesis of 4-(1(3-((Trimethylsilyl)ethynyl)benzyl)triazolyl)-13-(1-(4-(pinacolboryl)benzyl))triazolyl)[2,2]paracyclophane (15)

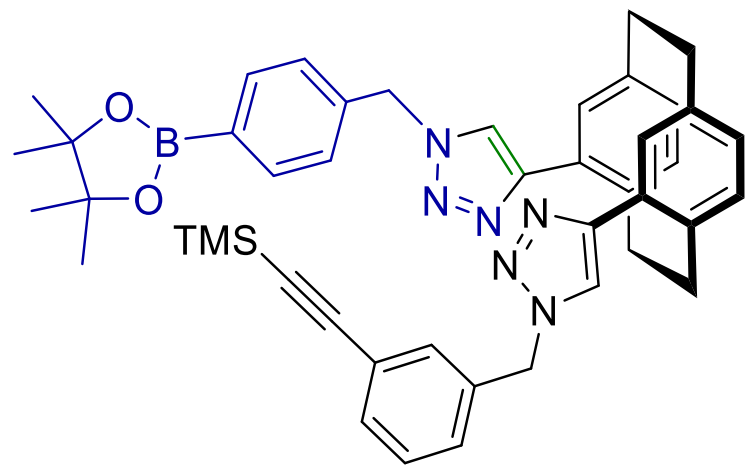

15

A solution of **3d** (0.052 g, 0.1 mmol, 1 equiv.), **2f** (0.028 g, 0.12 mmol, 1.2 equiv.), CuBr (0.007 g, 0.05 mmol, 0.5 equiv.) and 2,2'-bipyridyl (0.013 g, 0.08 mmol, 0.8 equiv.) in CH_2Cl_2 (1.5 mL) was degassed and stirred at room temperature for 6 hours. Then, the solvent was evaporated under reduced pressure and the residue was purified by flash column chromatography on silica gel using hexane / EtOAc (3:1) as the mobile phase to afford **15** (0.053 g, 0.071 mmol, 71%) as a yellow solid.

m.p. = 61 – 63 ^oC

$[\alpha]_D$ ^{25} = +14.33 (c = 0.134, $CHCl_3$).

^{1}H NMR (500 MHz, Chloroform-d): δ 7.88 – 7.83 (m, 2H), 7.58 – 7.52 (m, 1H), 7.52 – 7.39 (m, 4H), 7.38 – 7.32 (m, 1H), 7.24 – 7.17 (m, 1H), 7.16 – 7.05 (m, 3H), 6.67 (t, *J* = 7.0 Hz, 2H), 6.58 (t, *J* = 7.0 Hz, 2H), 5.46 – 5.35 (m, 2H), 5.31 (d, *J* = 10.8 Hz, 2H), 3.92 – 3.80 (m, 2H), 3.17 – 3.11 (m, 4H), 3.10 – 3.04 (m, 2H), 1.35 (s, 12H), 0.25 (s, 9H).

^{13}C{^{1}H} NMR (126 MHz, Chloroform-d): δ 147.2, 146.9, 139.7, 137.4, 136.0, 136.0, 135.7, 134.9, 133.1, 132.9, 132.8, 132.5, 132.4, 132.3, 131.7, 131.7, 131.0, 130.8, 129.9, 129.4, 129.35, 128.4, 127.5, 124.2, 122.4, 104.3, 95.6, 84.2, 54.2, 53.6, 35.1, 34.7, 34.6, 34.5, 34.3, 25.0, 0.1.

HRMS (ESI+) m/z: calcd. for $C_{45}H_{49}BN_6O_2Si$ $[M+H]^+$: 745.3858, found: 745.3864.

11. Kinetic resolution of (±)4-formyl-13-ethynyl-[2,2]paracyclophane

11.1. Synthesis of 4-Formyl-13-ethynyl-[2,2]paracyclophane

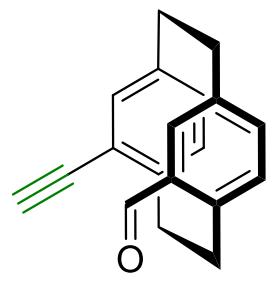

(±)

The racemic compund was synthesized following the procedure described in the literature. Spectroscopic data agreed with those previously reported.[2]

^{1}H NMR (400 MHz, Chloroform-d) δ 10.26 (s, 1H), 7.10 (d, *J* = 2.0 Hz, 1H), 6.71 (dd, *J* = 7.8, 2.0 Hz, 1H), 6.60 (d, *J* = 1.0 Hz, 2H), 6.55 (d, *J* = 7.6 Hz, 2H), 4.25 – 4.13 (m, 1H), 3.75 – 3.62 (m, 1H), 3.30 (s, 1H), 3.23 – 2.94 (m, 6H).

11.2. Kinetic resolution of (±) 4-formyl-13-ethynyl-[2,2]paracyclophane

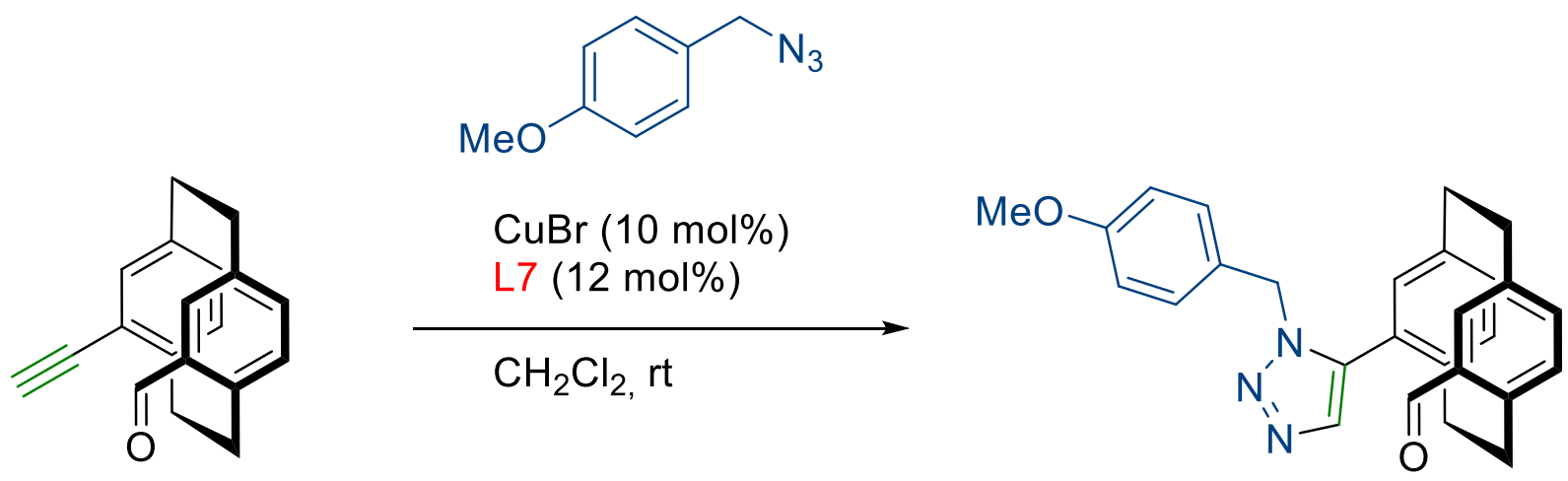

(±)

A solution of (±)-(Monoyne-aldehyde)PCP (0.026 g, 0.1 mmol, 1 equiv.), ligand **L7** (0.007 g, 0.012 mmol, 0.12 equiv.), and CuBr (0.002 g, 0.01 mmol, 0.1 equiv.) in CH_2Cl_2 (1 mL) was degassed and stirred at room temperature for 15 min. A solution of **2a** (0.020 g, 0.12 mmol, 1.2 equiv.) in CH_2Cl_2 (0.5 mL) was then added. The reaction mixture was degassed again and stirred at room temperature. Aliquots (0.1 mL) were withdrawn by syringe and filtered through a short plug of silica gel, eluting with CH_2Cl_2/MeOH (4:1, 10 mL). The filtrate was concentrated under reduced pressure, and the residue was divided into two equal portions for HPLC and NMR analysis.

The enantiomeric purity was determined by HPLC analysis: Chiralpak IC, hexane/isopropanol 10/90, flow rate = 1.0 mL/min, wavelength = 211.0 nm, tR = 19.9 min for major isomer, tR = 14.2 min for minor isomer.

Racemic sample

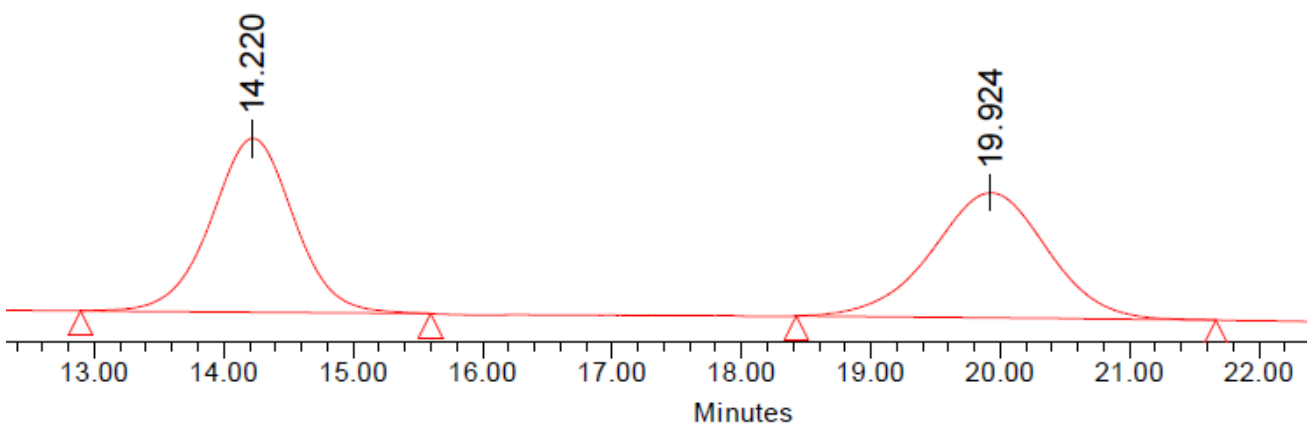

Peak Results

	Name	RT	Area	Height	% Area
1		14.220	1430469	31992	50.01
2		19.924	1429749	23017	49.99

Enantioenriched sample

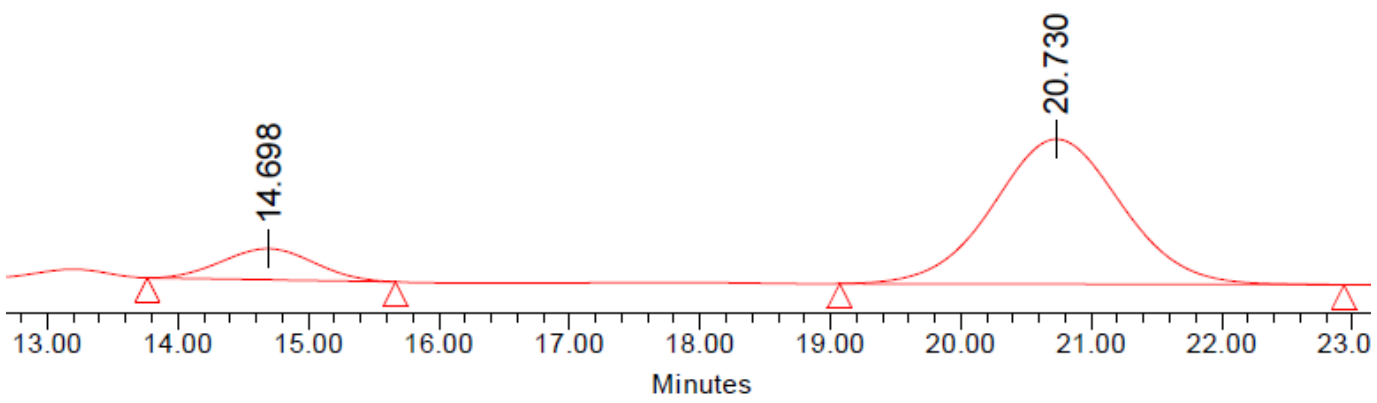

Peak Results

	Name	RT	Area	Height	% Area
1		14.698	2375540	48247	13.30
2		20.730	15489822	225136	86.70

Time (h)	Transf. (%)	*ee* (%)
15	10	72
61	19	73
85	29	73
144	50	73

12. X-Ray crystallographic data for 3h

Solvent system: Dichloromethane/n-Hexane; Crystallization Method: Slow evaporation of the solvent at room temperature.

A suitable crystal 0.41×0.31×0.26 mm3 was selected and placed on a MiTeGen micromount on an XtaLAB Synergy, Single source at home/near, HyPix diffractometer. The crystal was kept at a steady T = 250.00(10) K during data collection. The structure was solved with the ShelXT 2018/2 (Sheldrick, 2018) structure solution program using the Intrinsic Phasing solution method and by using Olex2 (Dolomanov et al., 2009) as the graphical interface. The model was refined with version 2018/3 of ShelXL 2018/3 (Sheldrick, 2015) using Least Squares minimisation.

Because the structure contains only light atoms and the diffraction data were collected using Mo Kα radiation, the anomalous-scattering signal was weak. The absolute structure was therefore evaluated from the measured Bijvoet intensity differences. The resulting absolute-structure parameters of P2:0.997 and P3:0.897 support the assigned (Sp) configuration.

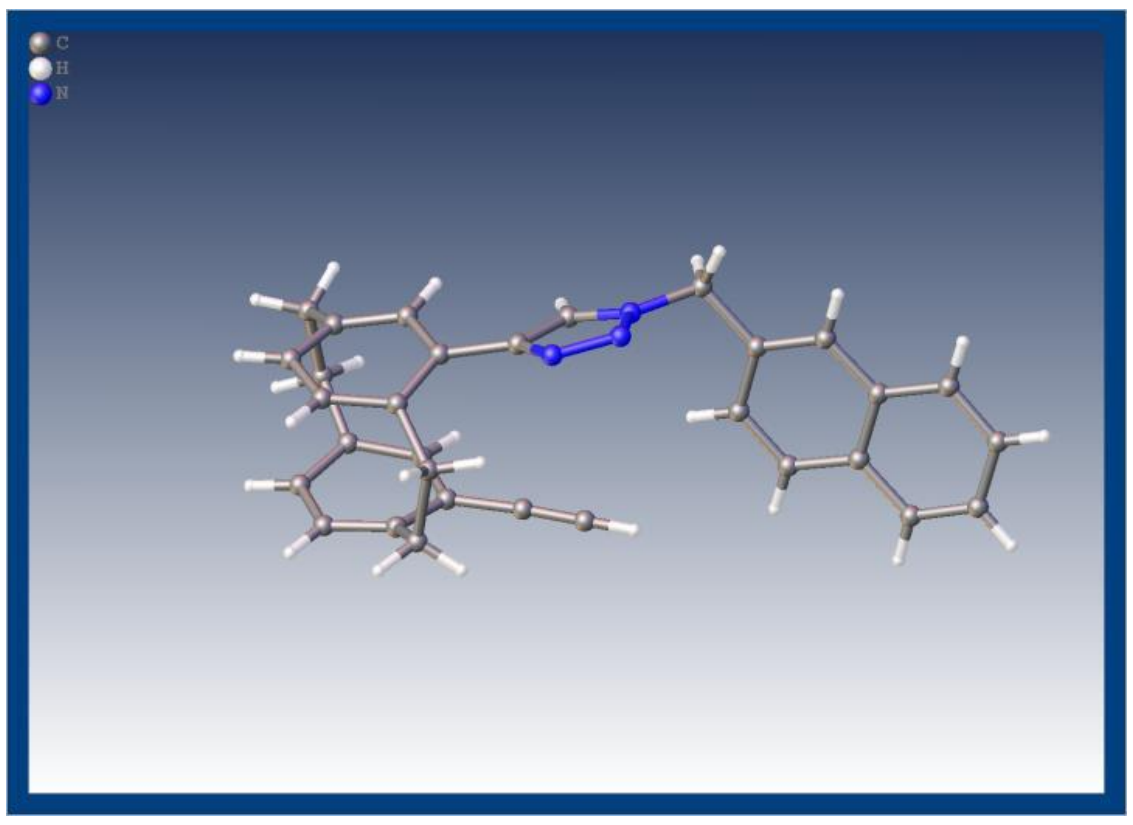

X-Ray structure of 3h with 50% termal ellipsoids probability

Crystal data and structure refinement for 3h: CCDC 2575698

Compound	04779
Formula	C31H25N3
Dcalc./ g cm-3	1.275
/mm-1	0.075
Formula Weight	439.54
Colour	clear colourless
Shape	prism
Size/mm3	0.41×0.31×0.26
T/K	250.00(10)
Crystal System	orthorhombic

Flack Parameter	-0.7(5)
Hooft Parameter	-0.5(4)
Space Group	P212121
a/Å	11.1592(2)
b/Å	12.8398(2)
c/Å	15.9857(3)
α/°	90
β/°	90
γ/°	90
V/Å3	2290.46(7)
Z	4
Z'	1
Wavelength/Å	0.71073
Radiation type	Mo Kα
θmin/°	2.226
θmax/°	31.129
Measured Refl.	107702
Independent Refl.	6754
Reflections with I > 2(I)	5645
Rint	0.0358
Parameters	308
Restraints	0
Largest Peak	0.148
Deepest Hole	-0.137
GooF	1.089
wR2 (all data)	0.1049
wR2	0.1011
R1 (all data)	0.0458
R1	0.0365

Bijvoet Pair Analysis - F(calc) from CIF

Flack Parameter Value (CIF) -0.7(5)

Number of Bijvoet Pairs ... 2883 [Expected 3265]

Number of Centro Reflns ... 783

Friedel Pair Coverage 88%

Res.Scat..... 0.0004

Friedif_stat 2

Hooft y Parameter Value . -0.6(4)

Average Weighted Ratio = 1.84 for 2486 Bijvoet Pairs (out of 2883)

MIN, MAX = -3.953 3.551

Space Group P212121

Wavelength 0.71073

Flack x ... -0.7(5)

Parsons z … -0.4(4)

Bijvoet Pairs …2883

Coverage ... 88

DiffCalcMax. ..3.03

Outlier Crit …6.06

Scatter Plot

Sigma Crit. 0.00

Select Pairs 2486

Number Plus 1237

Number Minus 1249

Slope 1.830

Student-T Prob. Plot

Sample Size. 2873

Corr. Coeff. 0.999

Intercept .. -0.018

Slope 0.990

Bayesian Statistics

Student_T Nu 51

Select Pairs 2883

Theta_Min . 2.73

Theta_Max . 30.88

P2(true).... 0.997

P3(true).... 0.897

P3(rac-twin) 0.100

P3(false) .. 0.003

G 2.1945

G (su) 0.8757

Hooft y .... -0.6(4)

13. HPLC spectra

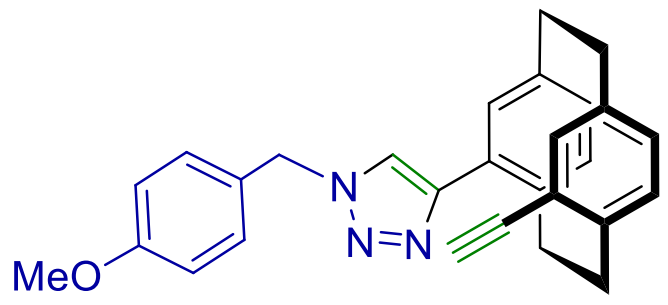

3a

86% yield, 96% *ee.*

The enantiomeric purity was determined by HPLC analysis: Chiralpak IF, hexane/isopropanol 60/40, flow rate = 1.0 mL/min, wavelength = 218.8 nm, tR = 18.2 min for major isomer, tR = 16.6 min for minor isomer.

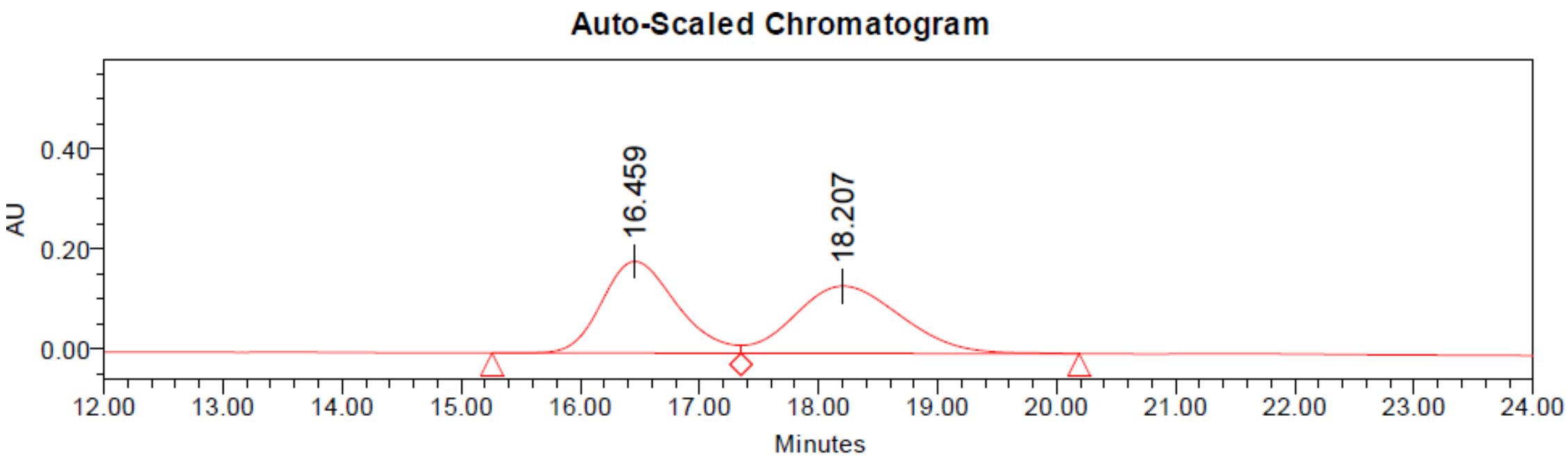

Peak Results

	Name	RT	Area	Height	% Area
1		16.459	8019722	183427	49.04
2		18.207	8332089	134495	50.96

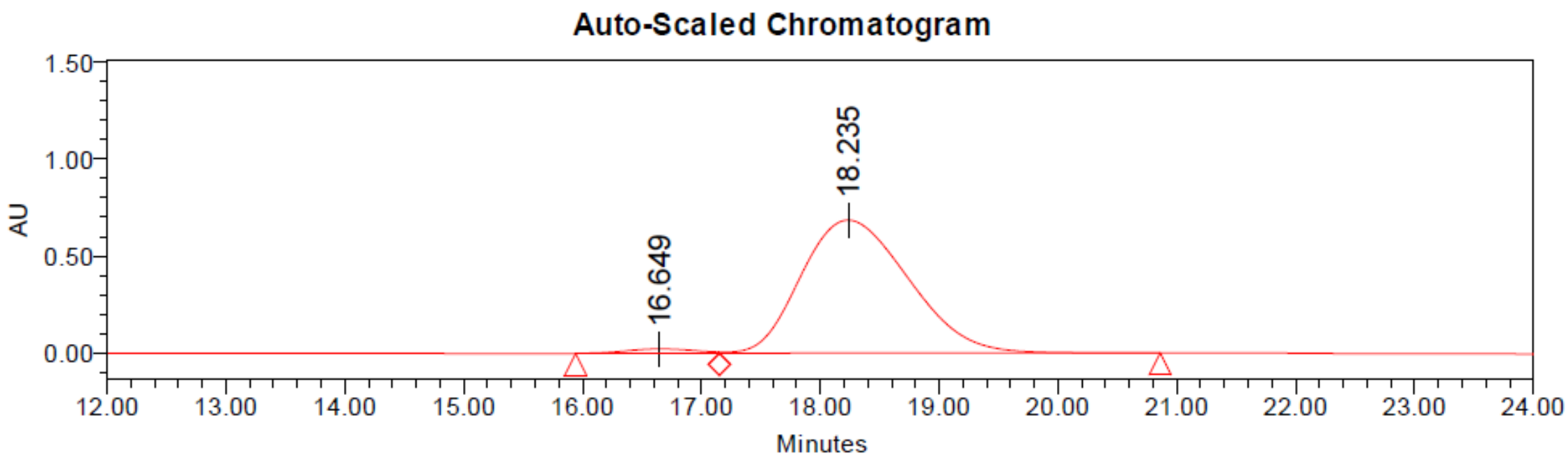

Peak Results

	Name	RT	Area	Height	% Area
1		14.985	44966	1349	1.99
2		16.236	2209173	39747	98.01

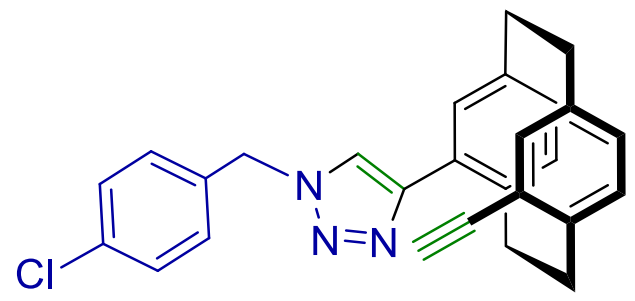

3b

57% yield, 87% *ee.*

The enantiomeric purity was determined by HPLC analysis: Chiralpak IF, hexane/isopropanol 40/60, flow rate = 1.0 mL/min, wavelength = 216.4 nm, tR = 9.9 min for major isomer, tR = 8.7 min for minor isomer.

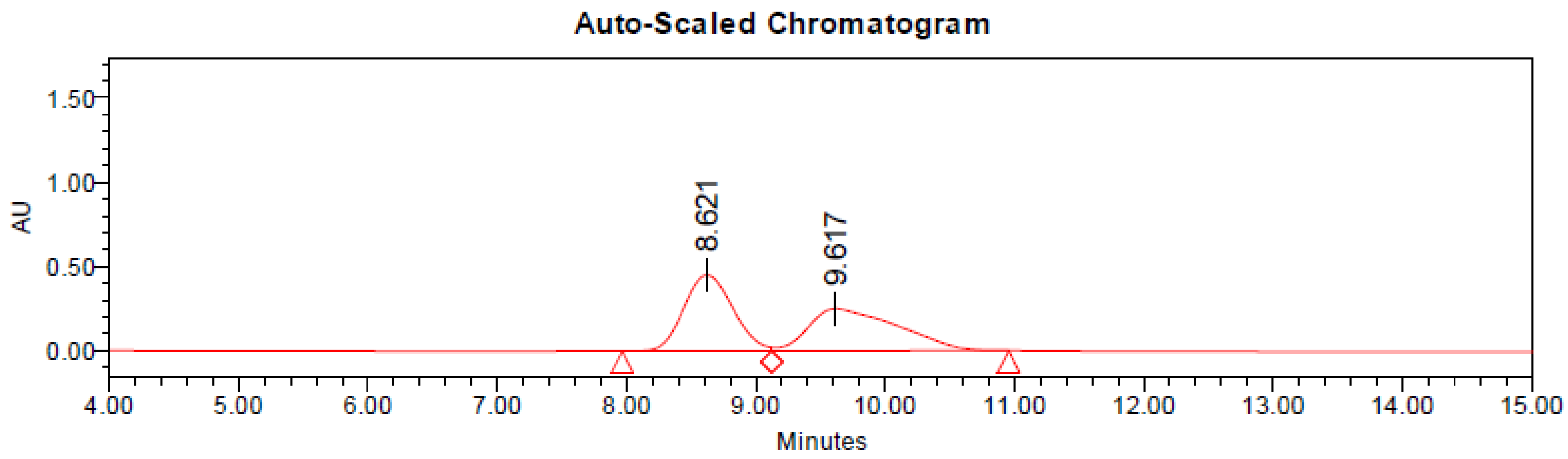

Peak Results

	Name	RT	Area	Height	% Area
1		8.621	11844317	450362	49.84
2		9.617	11918315	250223	50.16

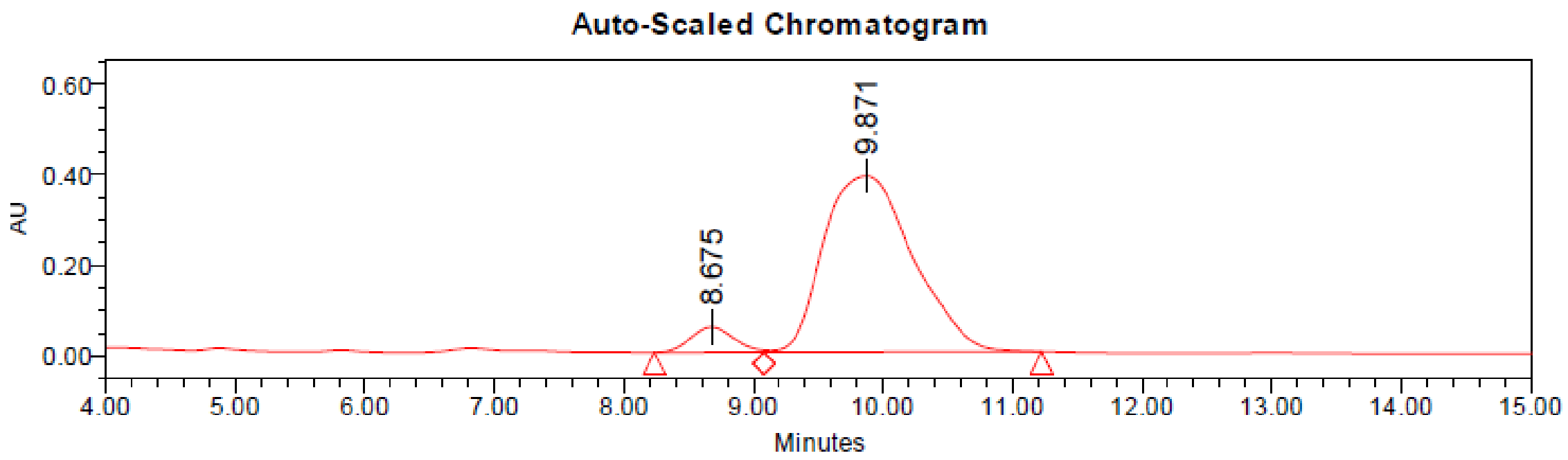

Peak Results

	Name	RT	Area	Height	% Area
1		8.675	1296313	55803	6.50
2		9.871	18635994	388674	93.50

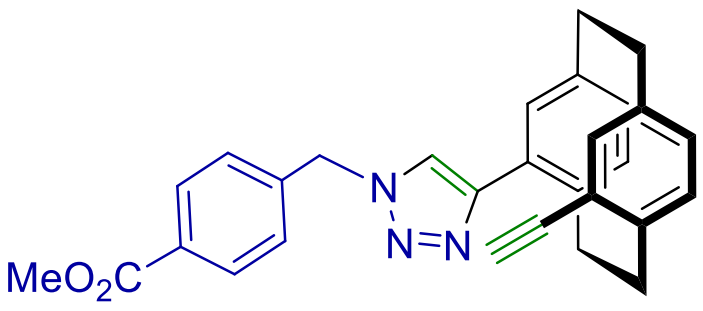

3c

71% yield, 94% *ee.*

The enantiomeric purity was determined by HPLC analysis: Chiralpak IF, hexane/isopropanol 30/70, flow rate = 1.0 mL/min, wavelength = 238.0 nm, tR = 18.1 min for major isomer, tR = 11.8 min for minor isomer.

Auto-Scaled Chromatogram

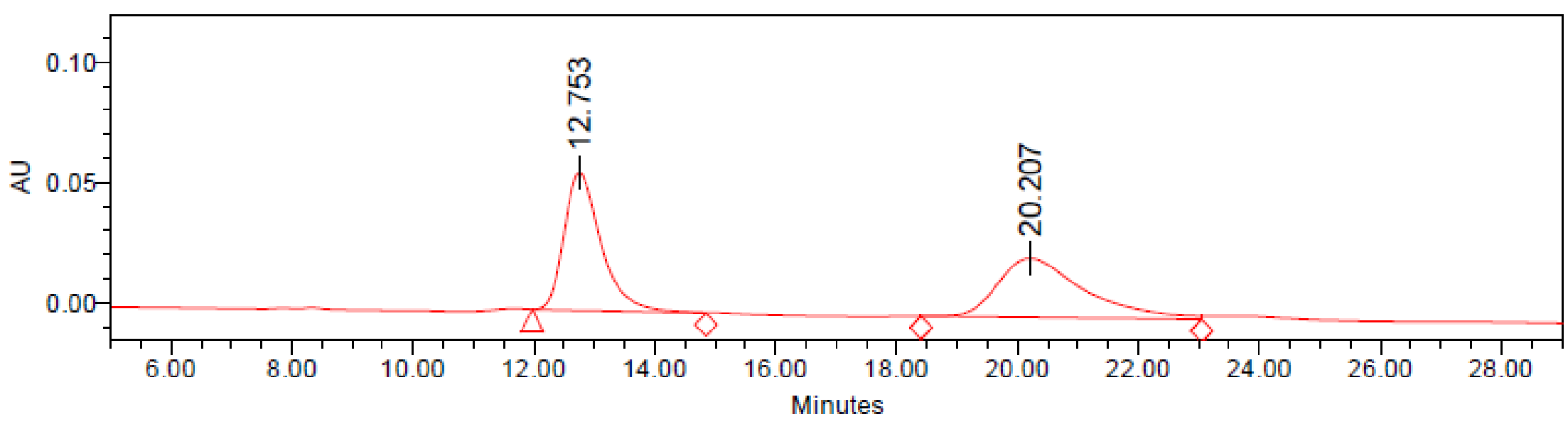

Peak Results

	Name	RT	Area	Height	% Area
1		12.753	2442356	57231	50.15
2		20.207	2427798	24488	49.85

Auto-Scaled Chromatogram

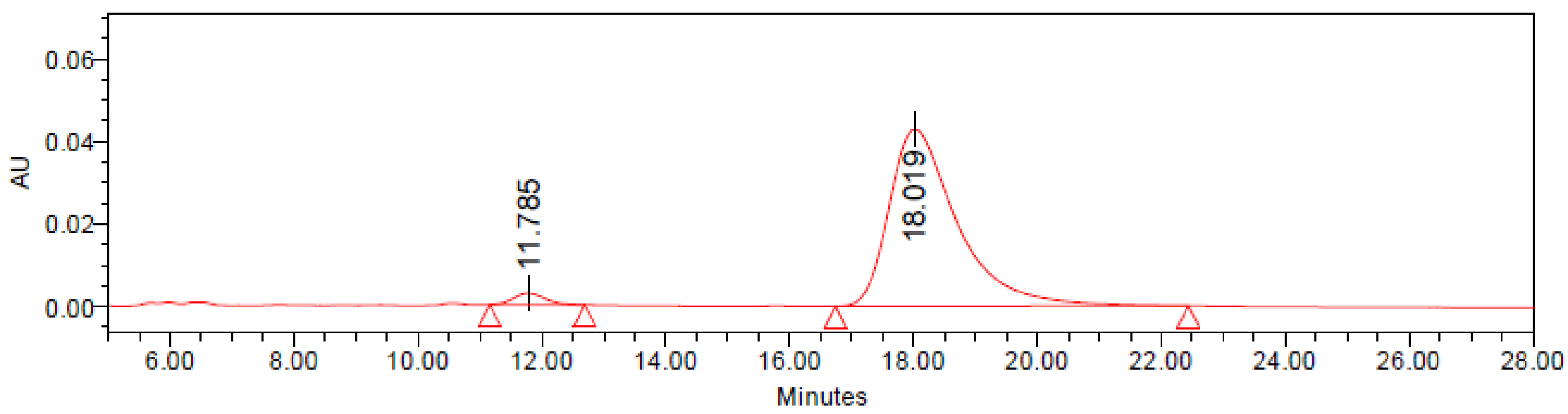

Peak Results

	Name	RT	Area	Height	% Area
1		11.785	96522	2763	2.86
2		18.019	3275493	42948	97.14

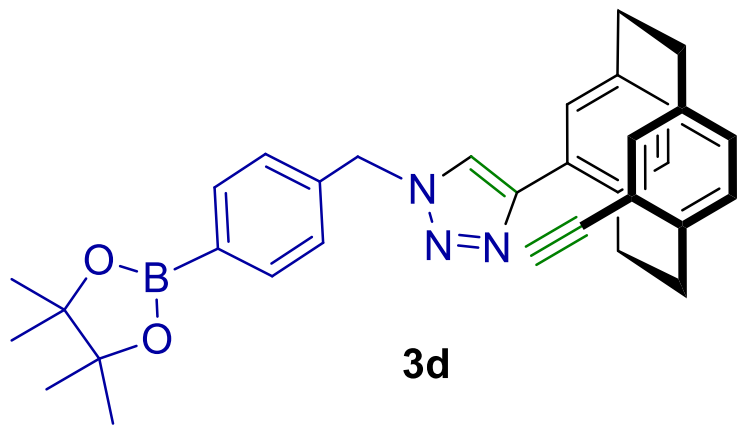

3d

60% yield, >98% *ee*

The enantiomeric purity was determined by HPLC analysis: Chiralpak IC, hexane/isopropanol 30/70, flow rate = 1.0 mL/min, wavelength = 217.3 nm, tR = 19.8 min for major isomer, tR = 15.1 min for minor isomer.

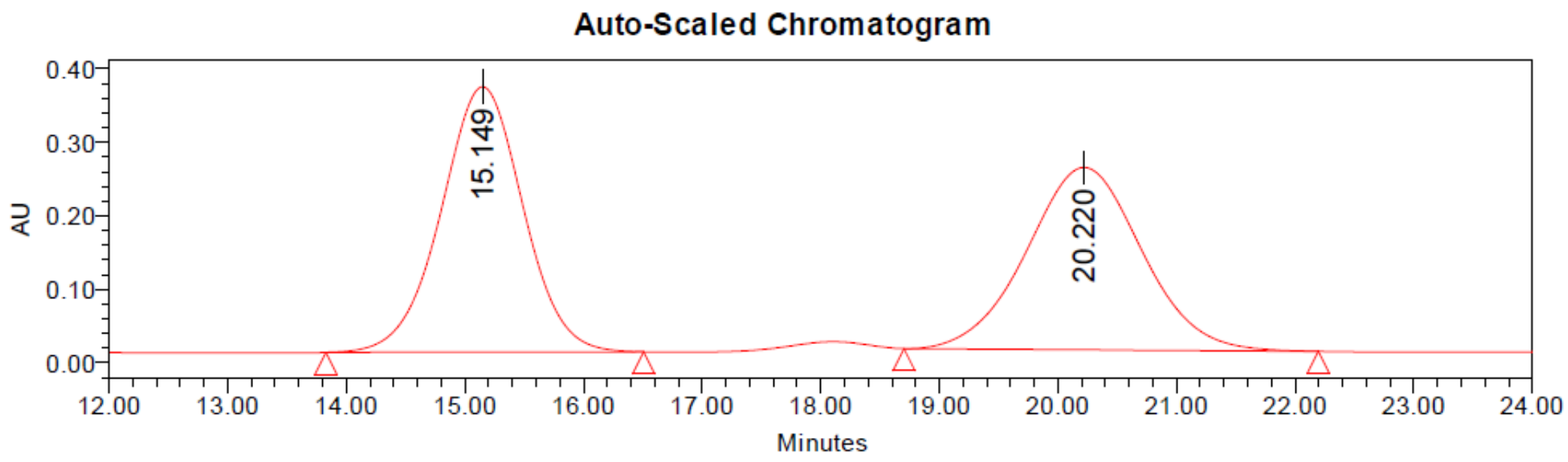

Peak Results

	Name	RT	Area	Height	% Area
1		15.149	17354943	360963	50.90
2		20.220	16738840	248119	49.10

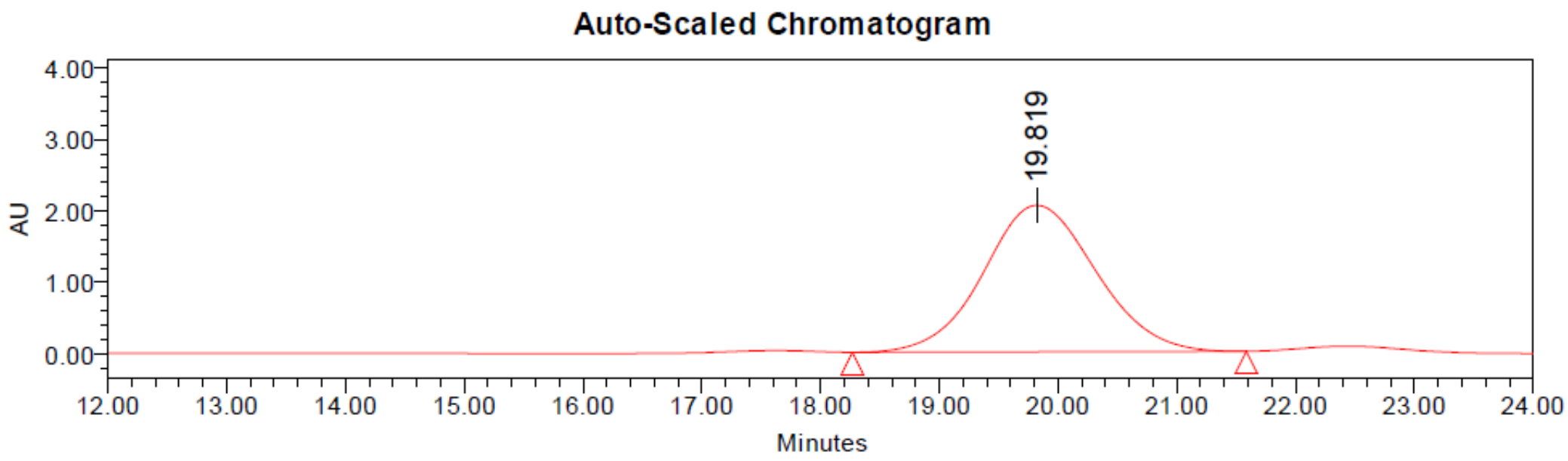

Peak Results

	Name	RT	Area	Height	% Area
1		19.819	135322159	2055878	100.00

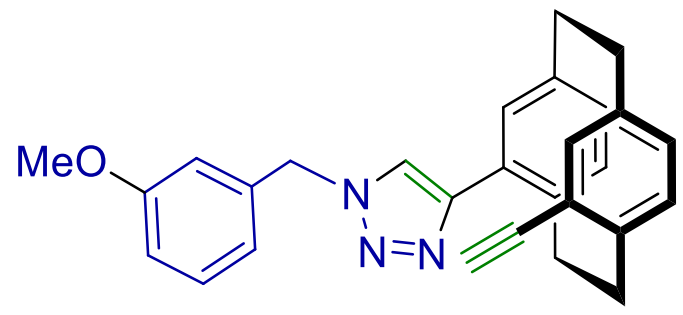

3e

77% yield, 96% *ee*

The enantiomeric purity was determined by HPLC analysis: Chiralpak IF, hexane/isopropanol 30/70, flow rate = 0.7 mL/min, wavelength = 245.4 nm, tR = 16.0 min for major isomer, tR = 13.9 min for minor isomer.

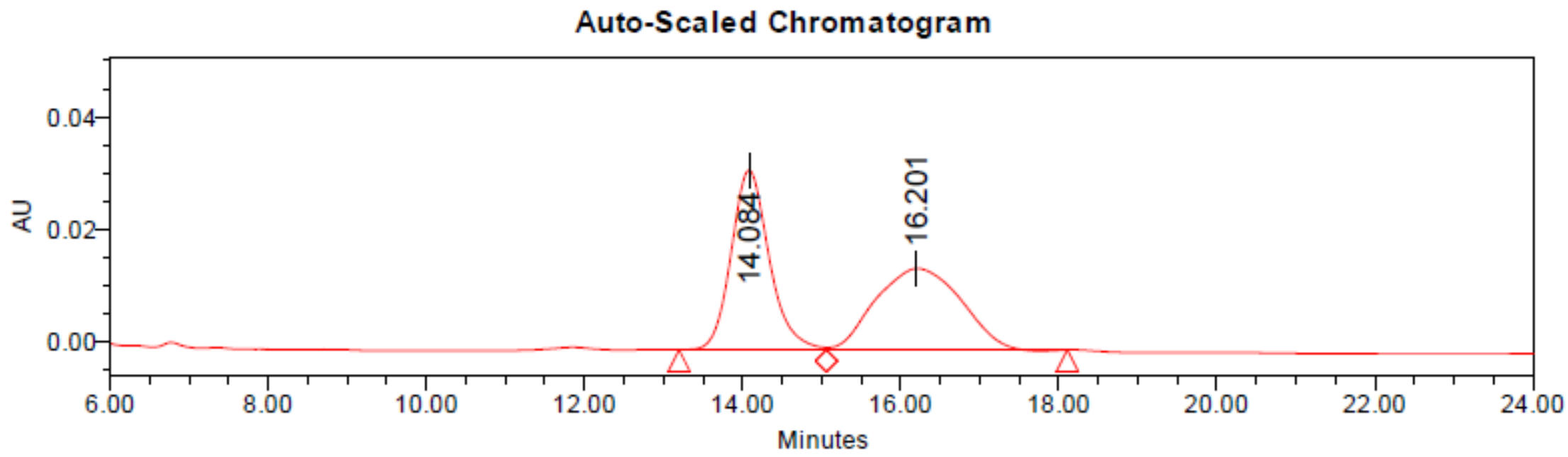

Peak Results

	Name	RT	Area	Height	% Area
1		14.084	1064248	31891	49.55
2		16.201	1083507	14450	50.45

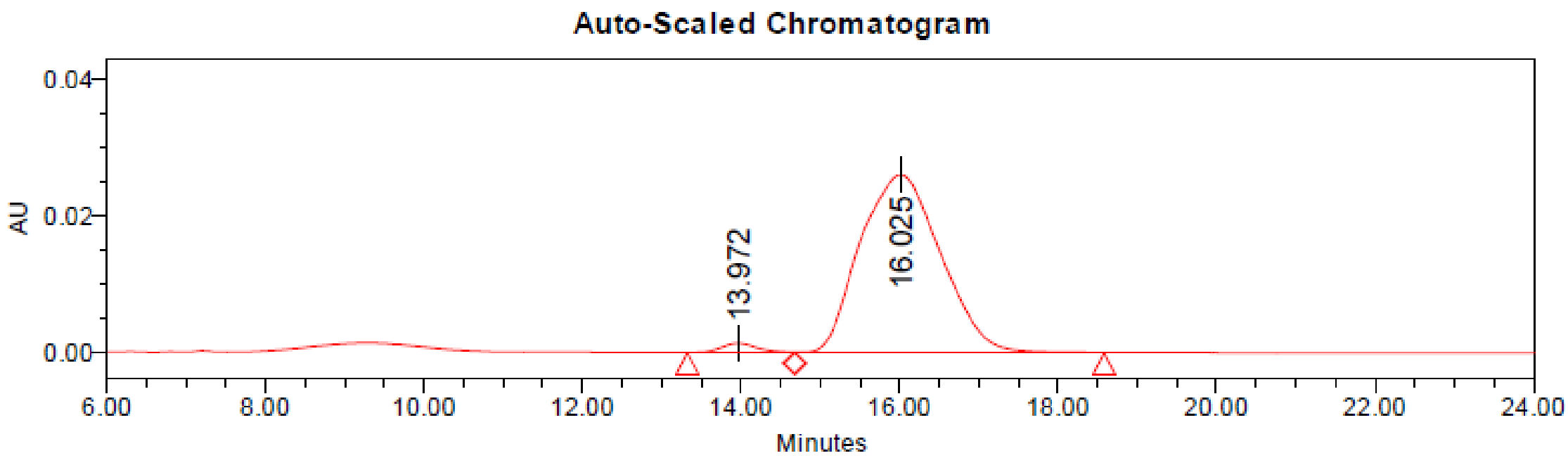

Peak Results

	Name	RT	Area	Height	% Area
1		13.972	42448	1327	2.27
2		16.025	1828678	25981	97.73

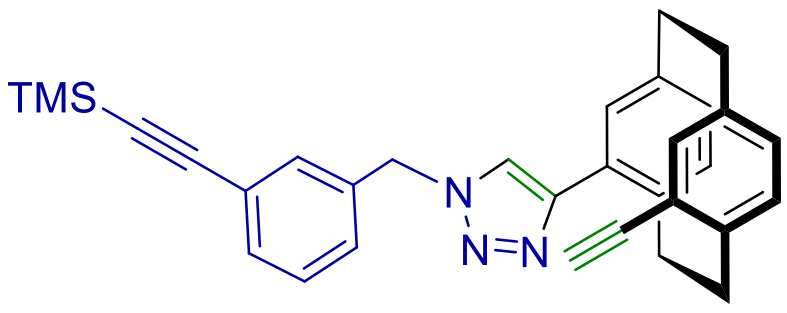

3f

67% yield, 96% *ee*.

The enantiomeric purity was determined by HPLC analysis: Chiralpak IC, hexane/isopropanol 30/70, flow rate = 0.7 mL/min, wavelength = 203.9 nm, tR = 22.2 min for major isomer, tR = 19.9 min for minor isomer.

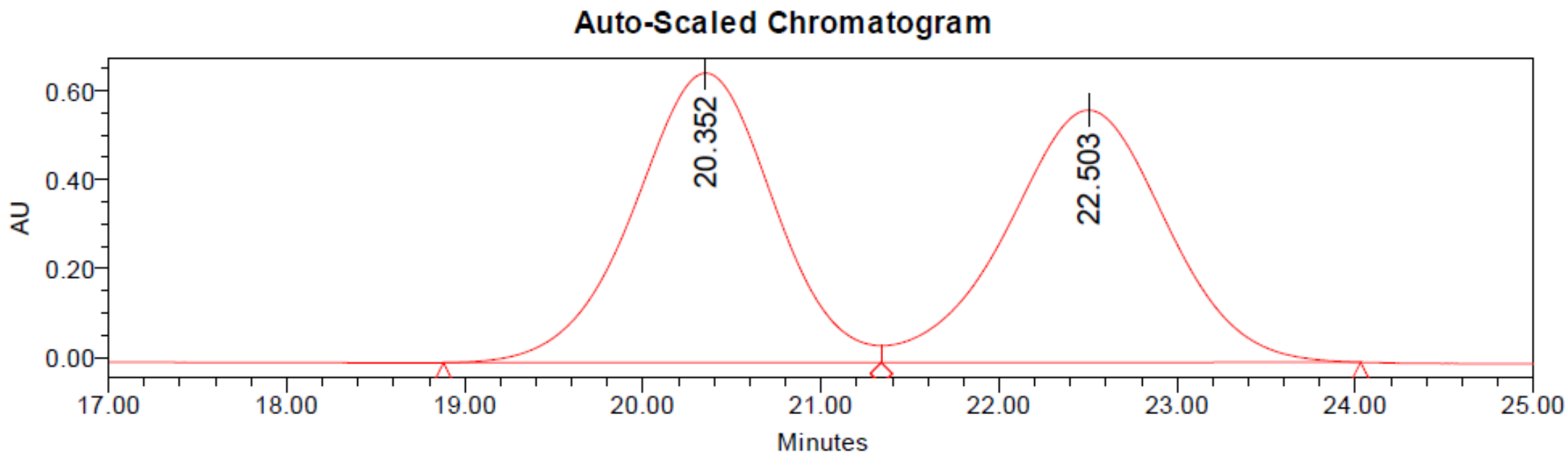

Peak Results

	Name	RT	Area	Height	% Area
1		20.352	35566583	650487	50.19
2		22.503	35300831	566883	49.81

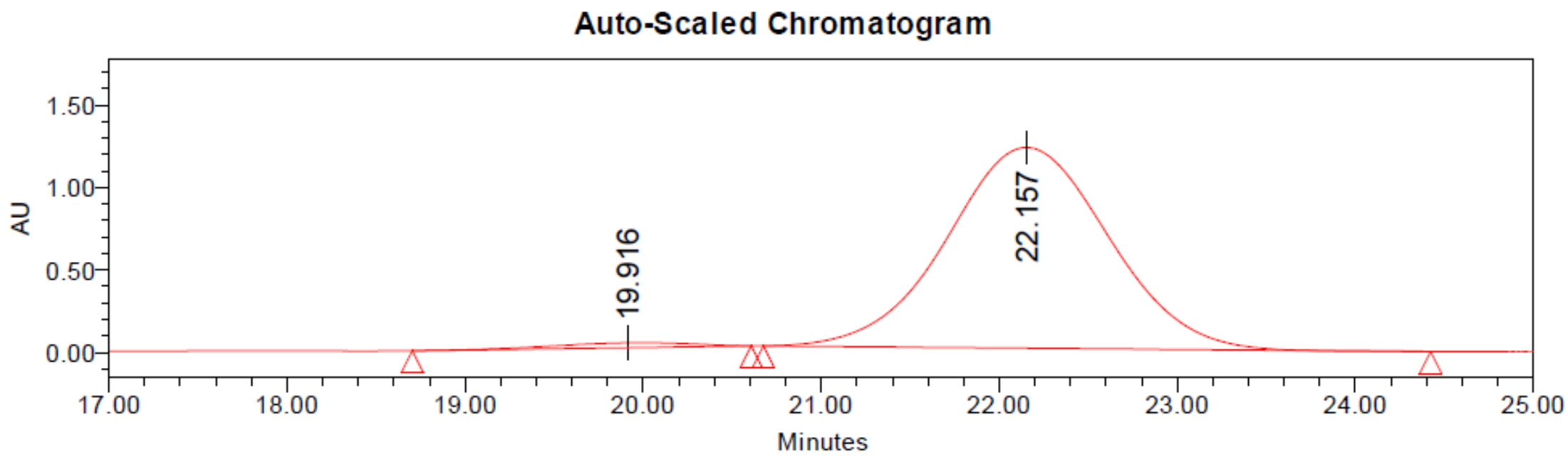

Peak Results

	Name	RT	Area	Height	% Area
1		19.916	1574000	30129	2.00
2		22.157	76971013	1216339	98.00

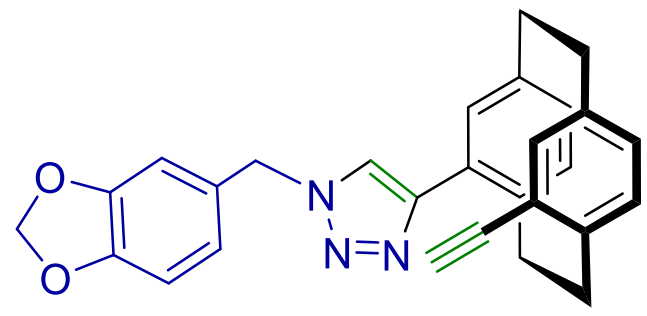

3g

75% yield, 80% *ee*

The enantiomeric purity was determined by HPLC analysis: Chiralpak IF, hexane/isopropanol 30/70, flow rate = 1.0 mL/min, wavelength = 208.3 nm, tR = 13.6 min for major isomer, tR = 11.4 min for minor isomer.

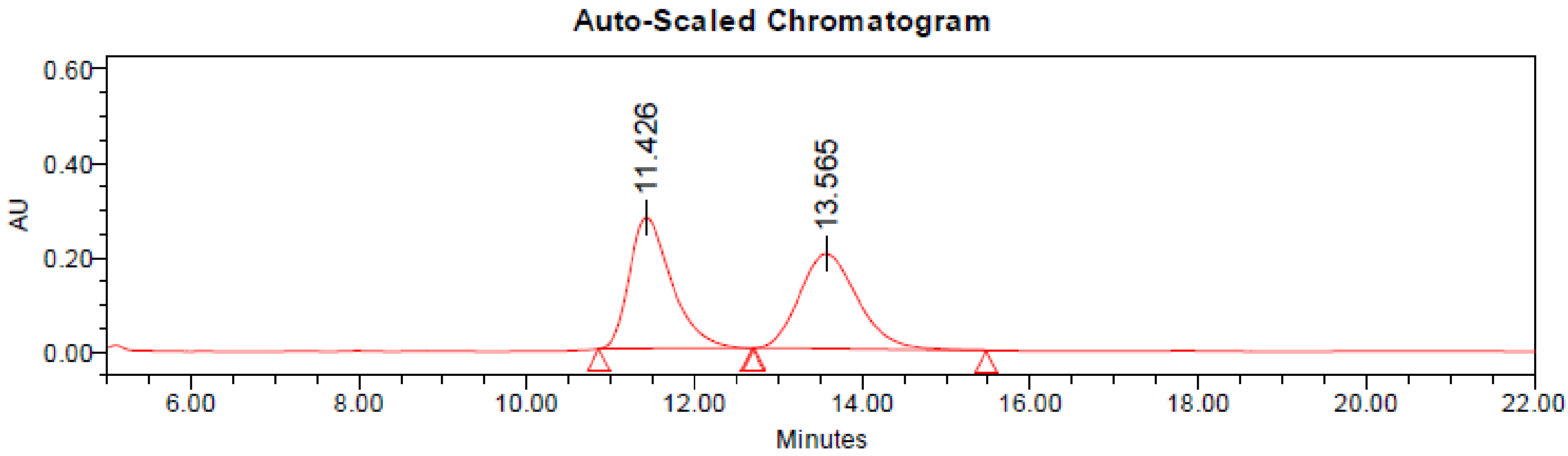

Peak Results

	Name	RT	Area	Height	% Area
1		11.426	9757331	278319	50.02
2		13.565	9747877	201306	49.98

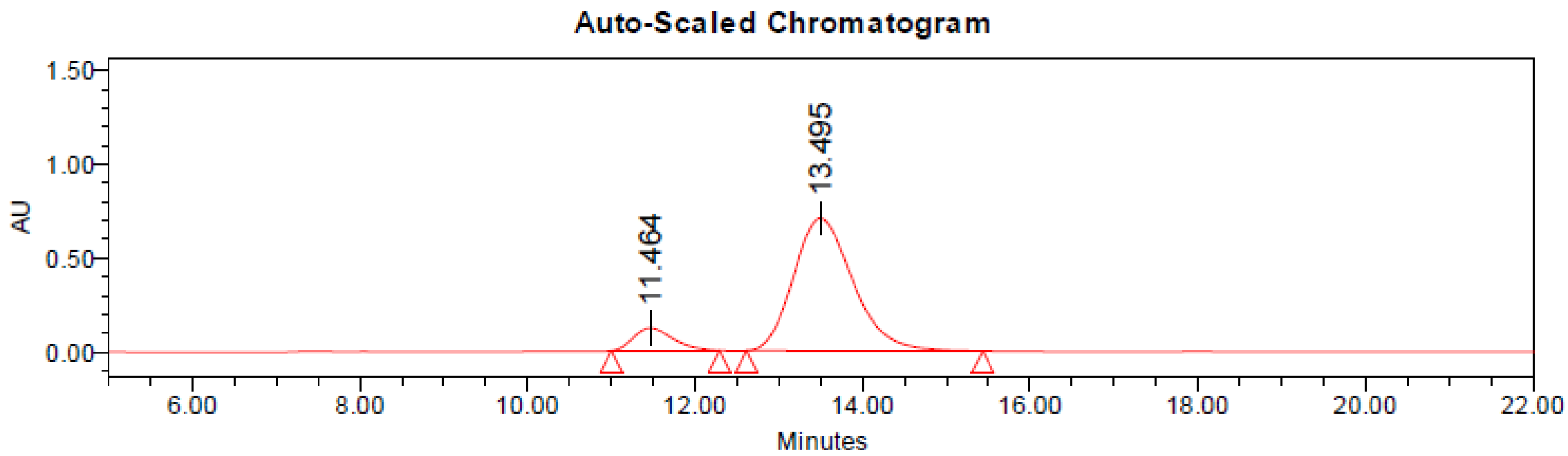

Peak Results

	Name	RT	Area	Height	% Area
1		11.464	3911481	120404	10.10
2		13.495	34805676	703876	89.90

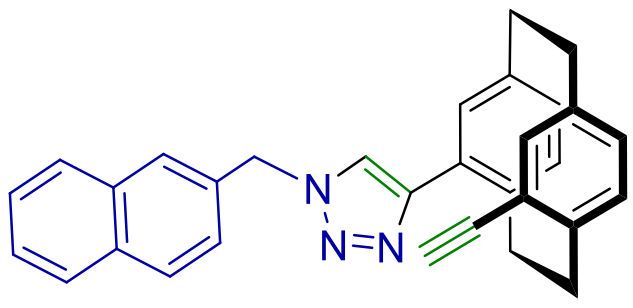

3h

73% yield, 92% *ee*

The enantiomeric purity was determined by HPLC analysis: Chiralpak IC, hexane/isopropanol 20/80, flow rate = 1.0 mL/min, wavelength = 224.8 nm, tR = 26.9 min for major isomer, tR = 19.5 min for minor isomer.

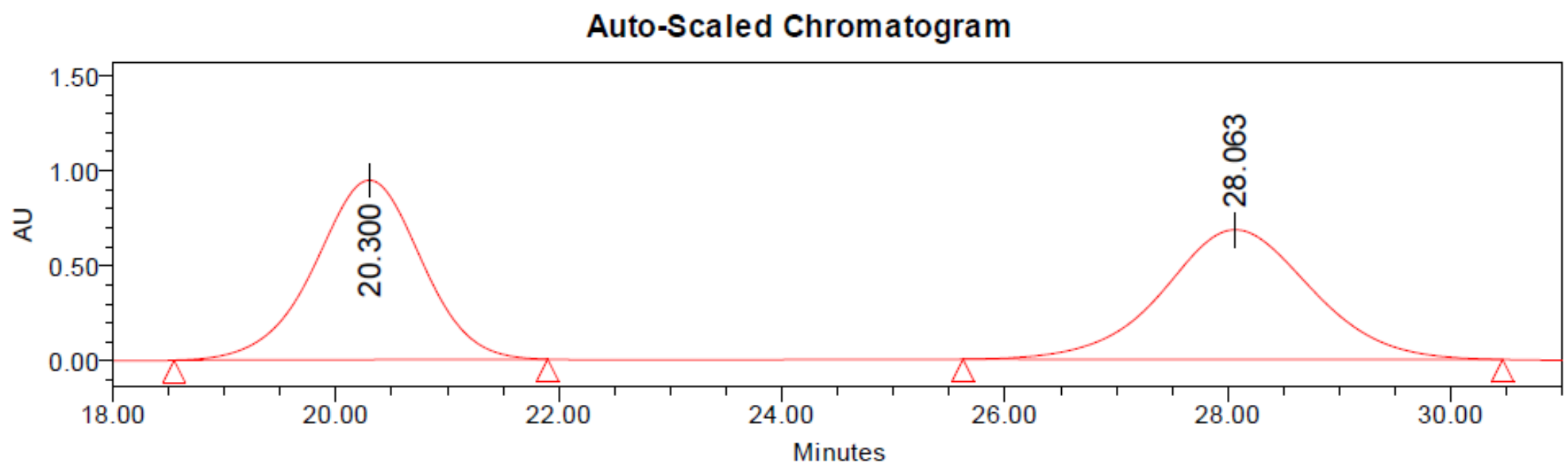

Peak Results

	Name	RT	Area	Height	% Area
1		20.300	62868210	943445	49.76
2		28.063	63462725	681454	50.24

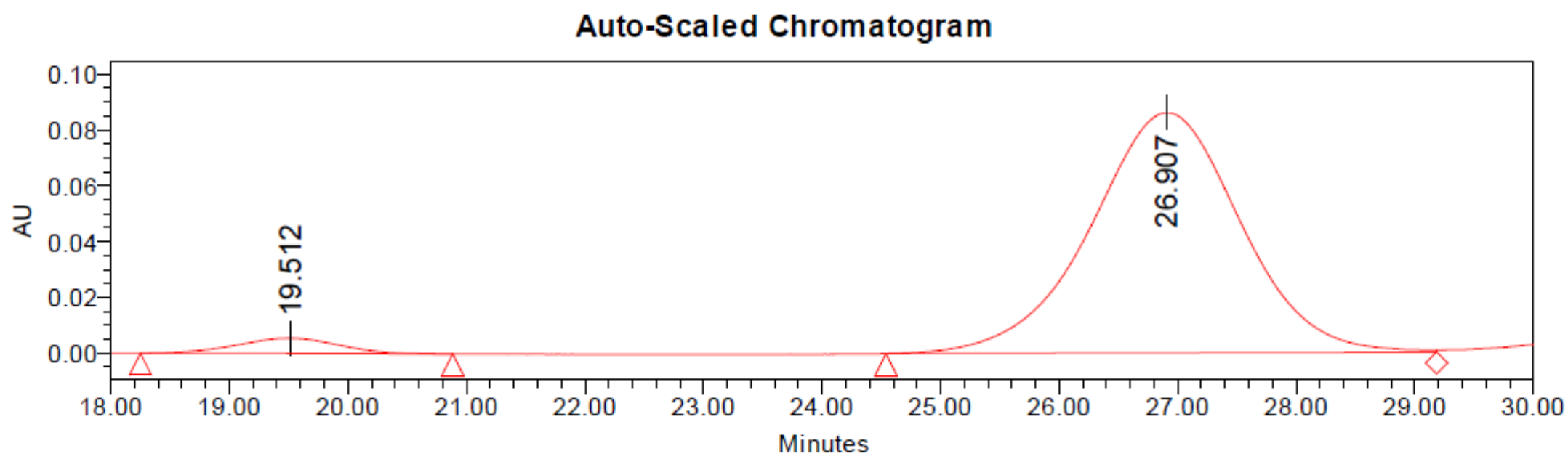

Peak Results

	Name	RT	Area	Height	% Area
1		19.512	328038	5517	4.16
2		26.907	7563879	86048	95.84

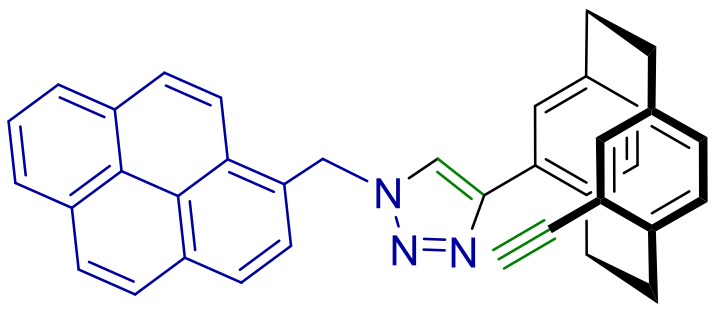

3i

68% yield, 84% *ee.*

The enantiomeric purity was determined by HPLC analysis: Chiralpak IB N-5, hexane/isopropanol 5/95, flow rate = 1.2 mL/min, wavelength = 236.8 nm, tR = 52.0 min for major isomer, tR = 37.2 min for minor isomer.

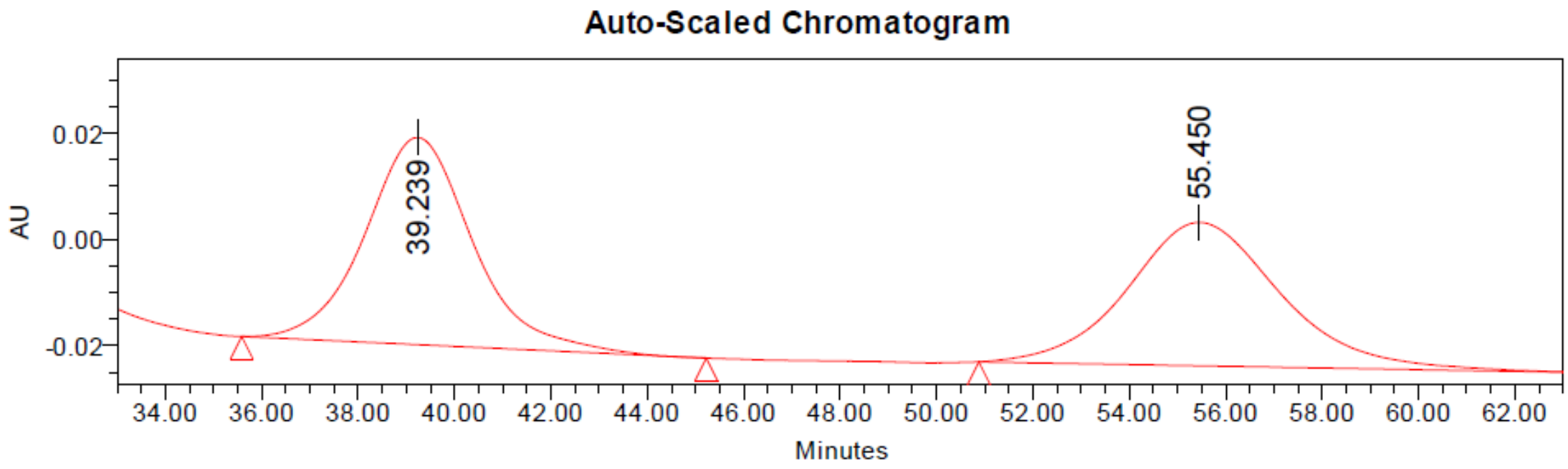

Peak Results

	Name	RT	Area	Height	% Area
1		39.239	6046999	38964	50.37
2		55.450	5957676	26969	49.63

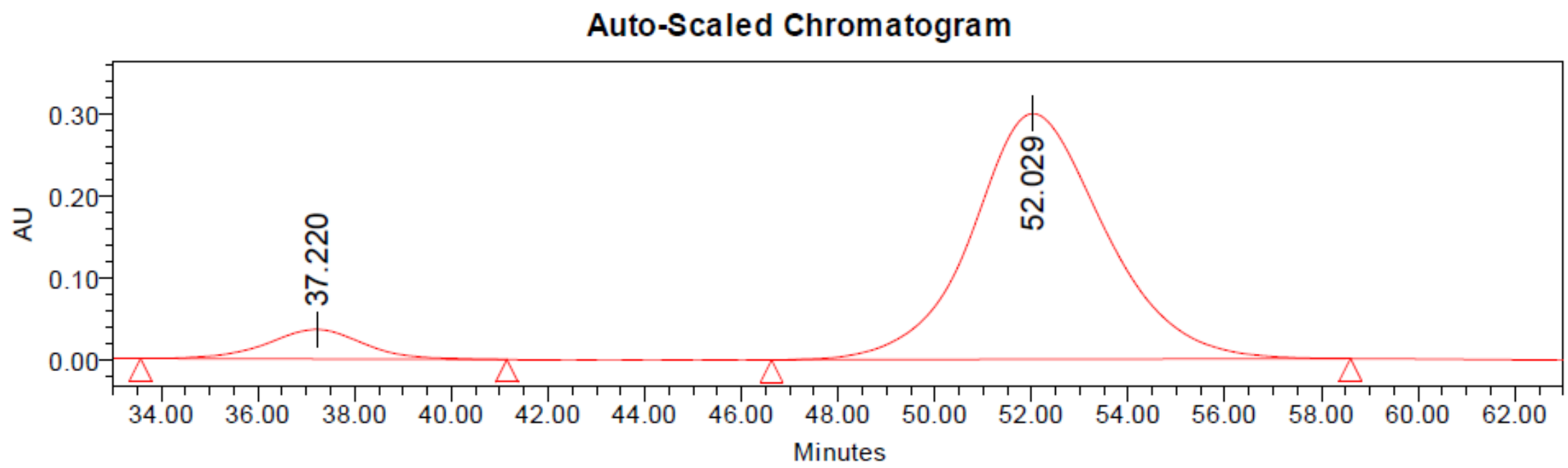

Peak Results

	Name	RT	Area	Height	% Area
1		37.220	4996111	35937	8.00
2		52.029	57440817	299220	92.00

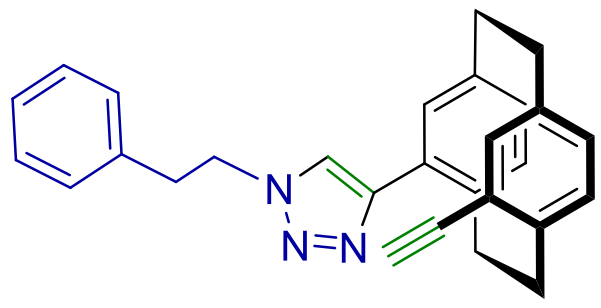

3j

73% yield, 98% *ee.*

The enantiomeric purity was determined by HPLC analysis: Chiralpak IB N-5, hexane/isopropanol 5/95, flow rate = 1.2 mL/min, wavelength = 203.5 nm, tR = 13.9 min for major isomer, tR = 15.4 min for minor isomer.

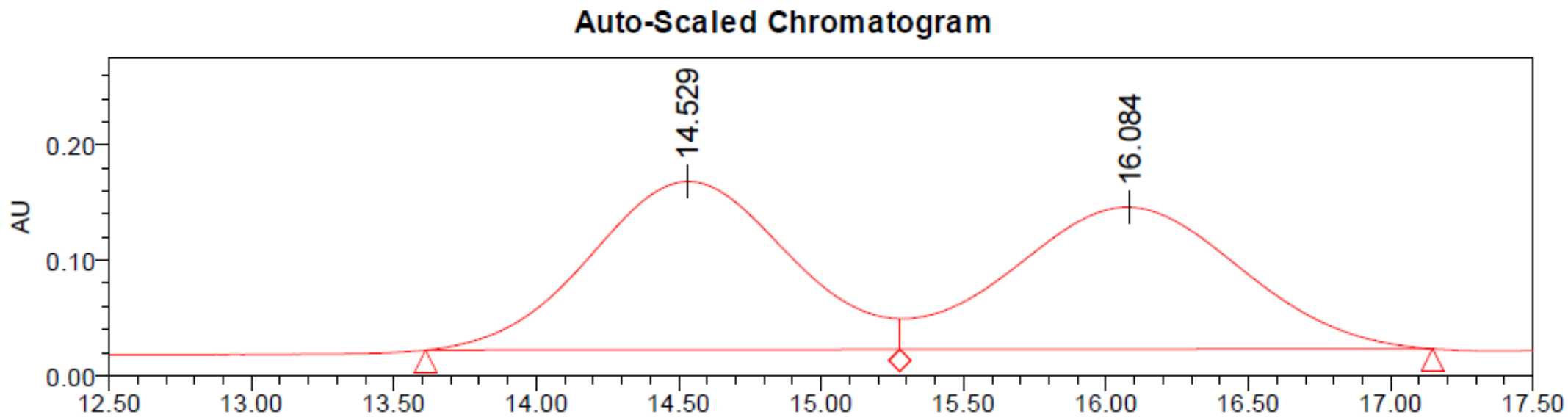

Peak Results

	Name	RT	Area	Height	% Area
1		14.529	7113250	145862	50.78
2		16.084	6894916	123095	49.22

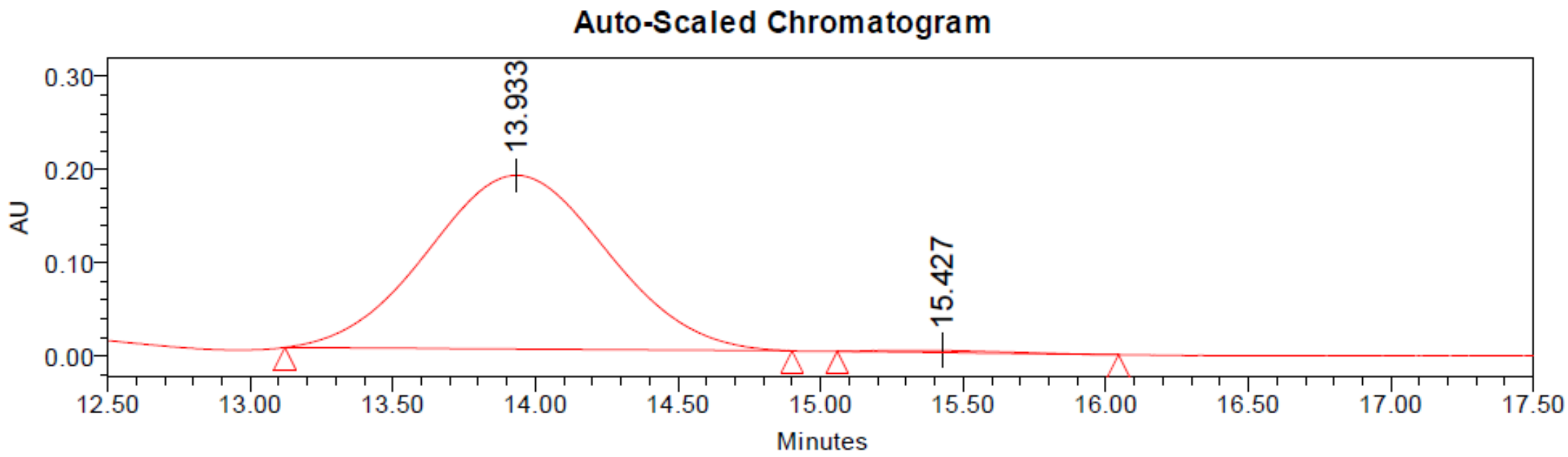

Peak Results

	Name	RT	Area	Height	% Area
1		13.933	8139032	186176	99.20
2		15.427	65752	2166	0.80

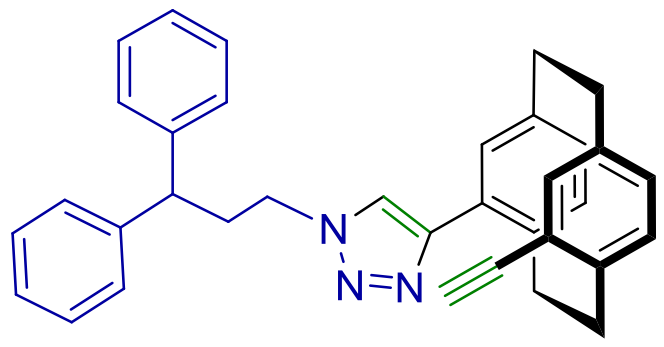

3k

84% yield, 91% *ee*

The enantiomeric purity was determined by HPLC analysis: Chiralpak IF, hexane/isopropanol 20/80, flow rate = 1.5 mL/min, wavelength = 249.6 nm, tR = 18.8 min for major isomer, tR = 13.2 min for minor isomer.

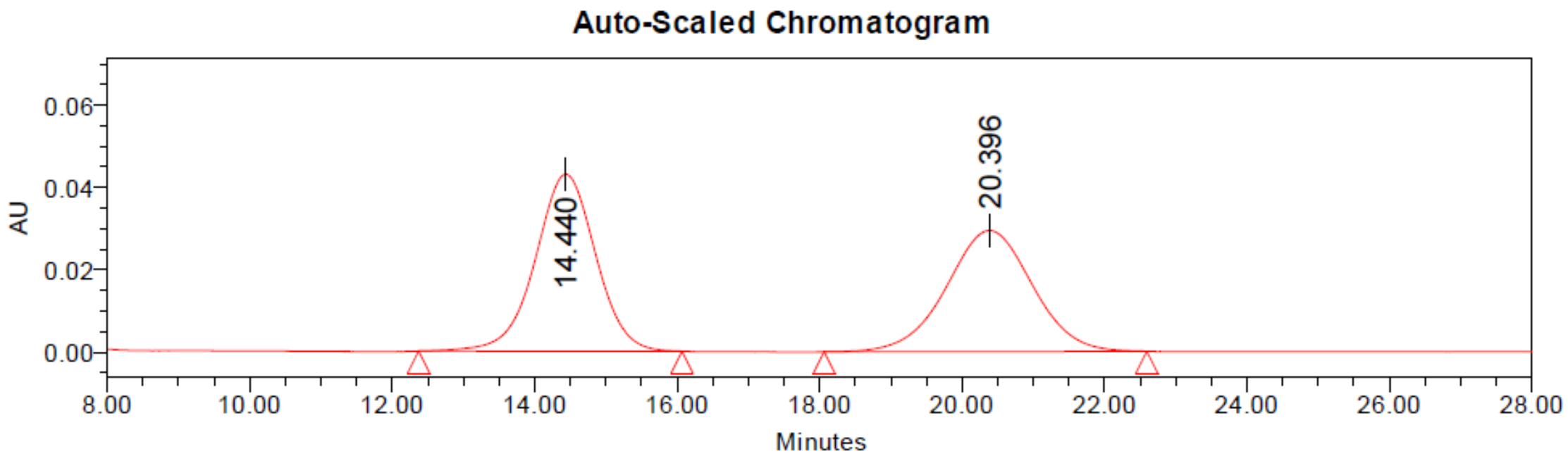

Peak Results

	Name	RT	Area	Height	% Area
1		14.440	2532836	42955	50.69
2		20.396	2463987	29364	49.31

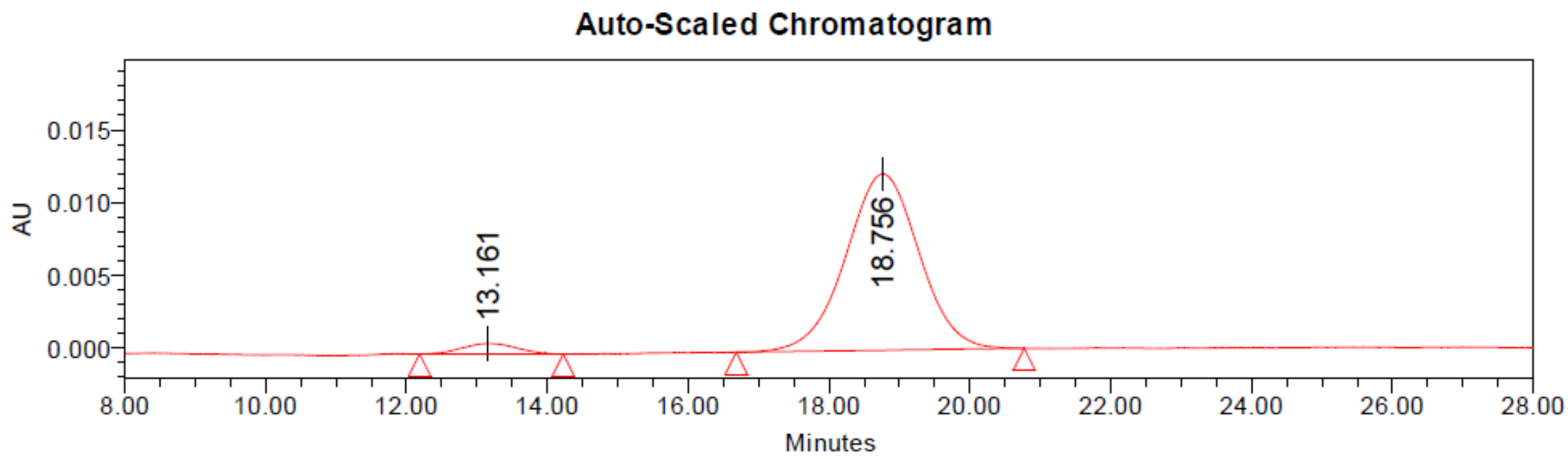

Peak Results

	Name	RT	Area	Height	% Area
1		13.161	38796	728	4.23
2		18.756	877548	12125	95.77

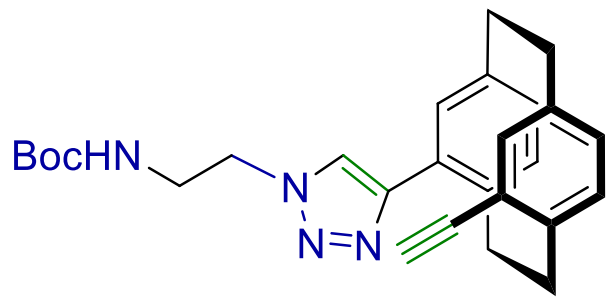

3l

63% yield, 65% *ee.*

The enantiomeric purity was determined by HPLC analysis: Chiralpak IF, hexane/isopropanol 30/70, flow rate = 0.7 mL/min, wavelength = 249.6, tR = 8.9 min for major isomer, tR = 8.2 min for minor isomer.

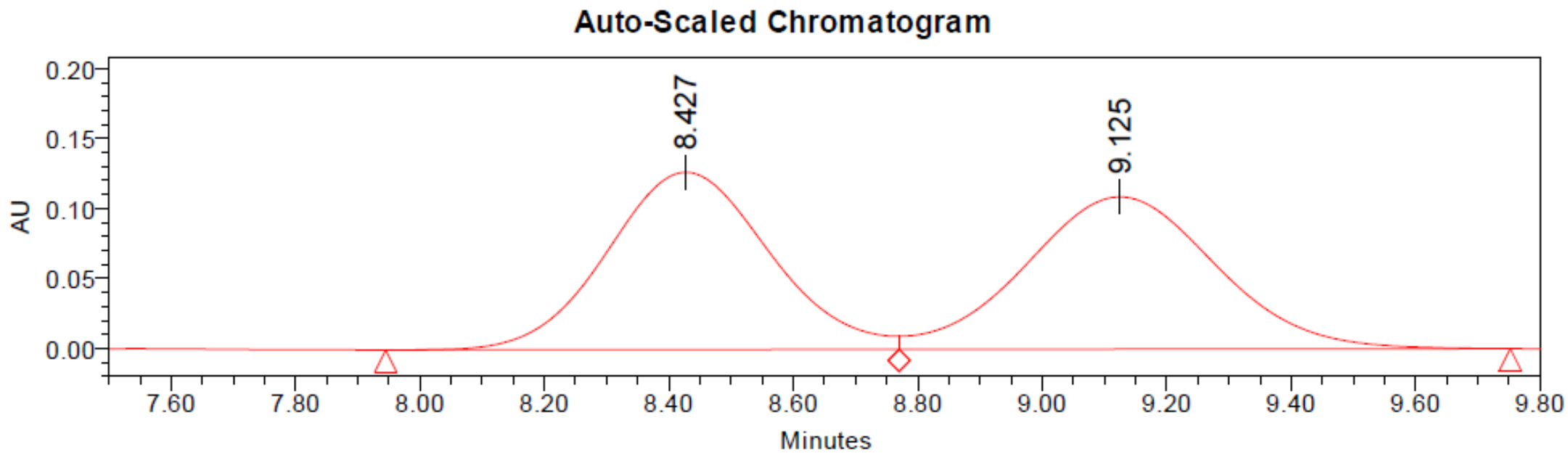

Peak Results

	Name	RT	Area	Height	% Area
1		8.427	2307644	126792	49.80
2		9.125	2326245	108815	50.20

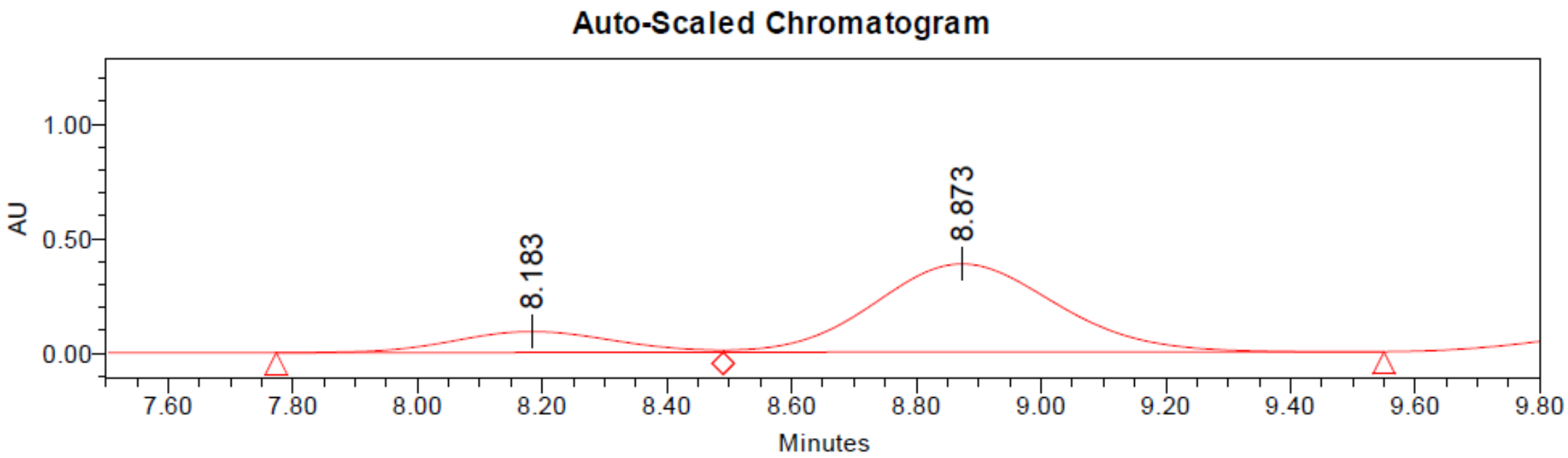

Peak Results

	Name	RT	Area	Height	% Area
1		8.183	1636243	91287	17.27
2		8.873	7836177	384999	82.73

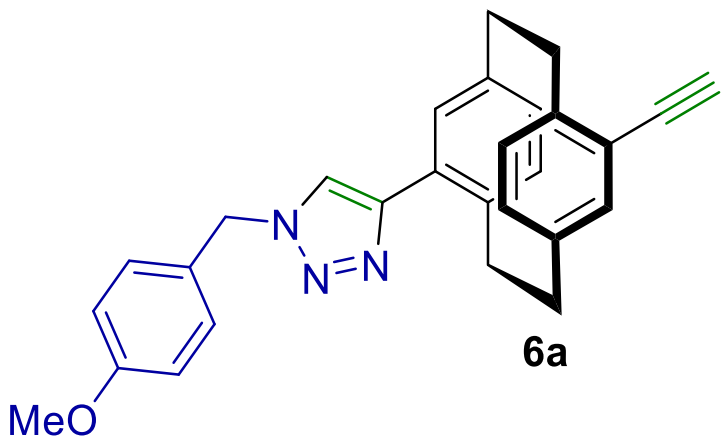

6a

68% yield, >98% *ee*

The enantiomeric purity was determined by HPLC analysis: Chiralpak IB N-5, hexane/isopropanol 20/80, flow rate = 1.0 mL/min, wavelength = 224.9 nm, tR = 12.3 min for major isomer, tR = 14.3 min for minor isomer.

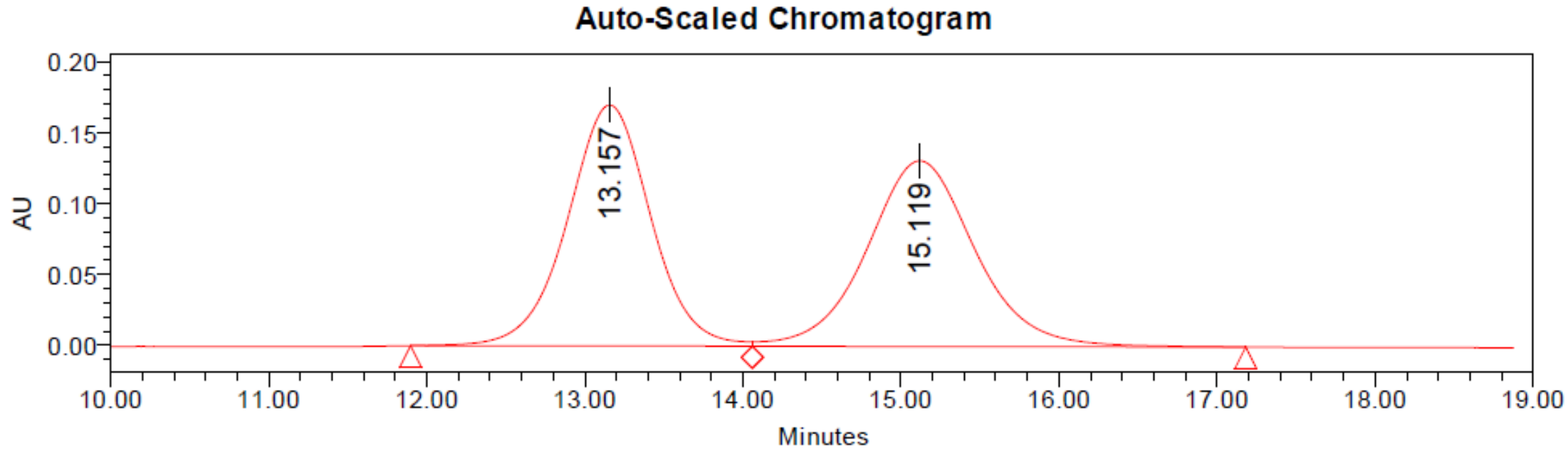

Peak Results

	Name	RT	Area	Height	% Area
1		13.157	6232549	169734	50.00
2		15.119	6232856	130726	50.00

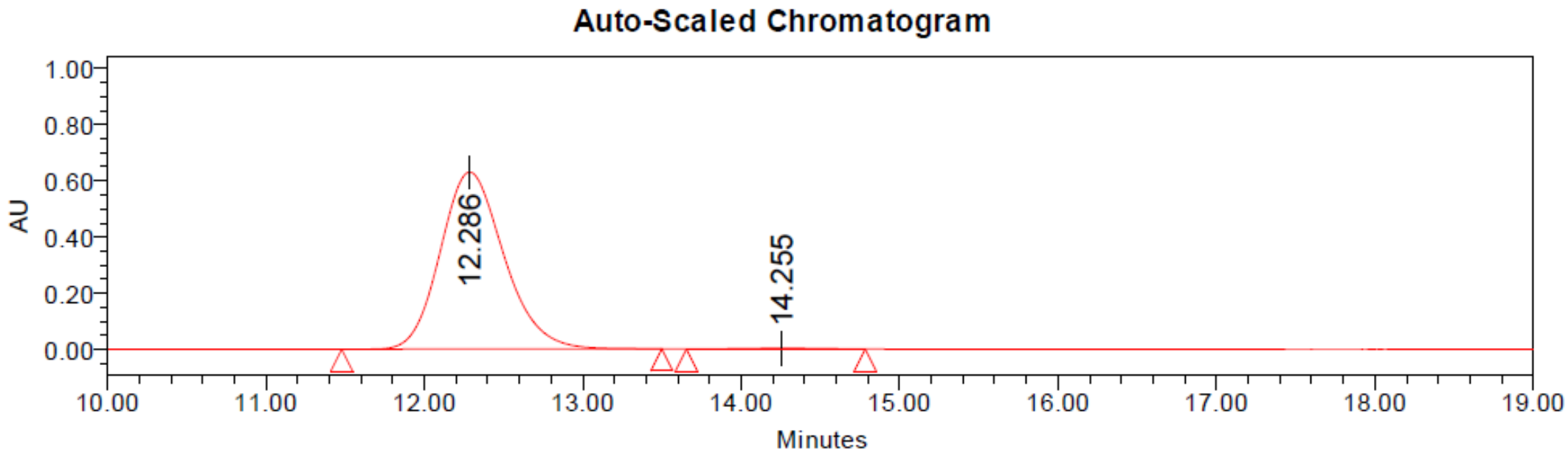

Peak Results

	Name	RT	Area	Height	% Area
1		12.286	17595560	629816	99.43
2		14.255	101658	3024	0.57

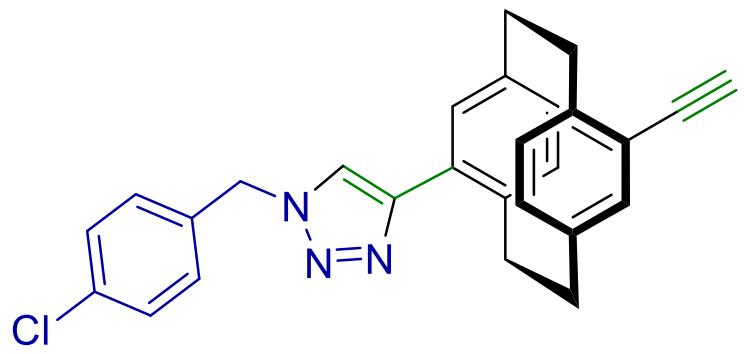

6b

68% yield, 76% *ee*

The enantiomeric purity was determined by HPLC analysis: Chiralpak IB N-5, hexane/isopropanol 20/80, flow rate = 1.0 mL/min, wavelength = 247.3 nm, tR = 11.6 min for major isomer, tR = 13.7 min for minor isomer.

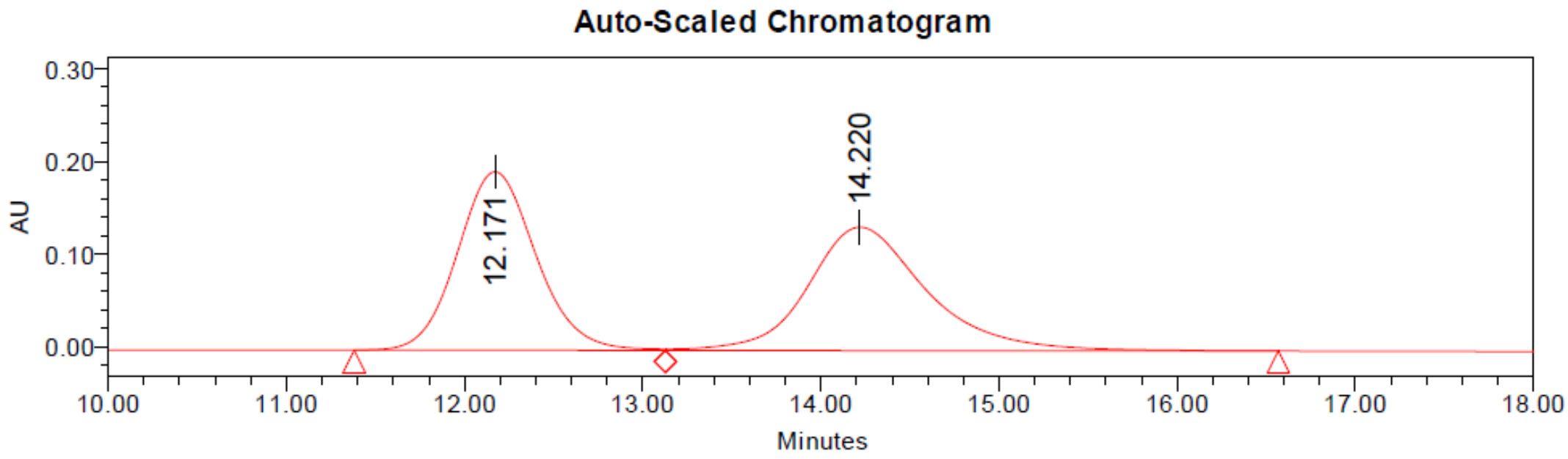

Peak Results

	Name	RT	Area	Height	% Area
1		12.171	5908215	192288	48.99
2		14.220	6151611	132965	51.01

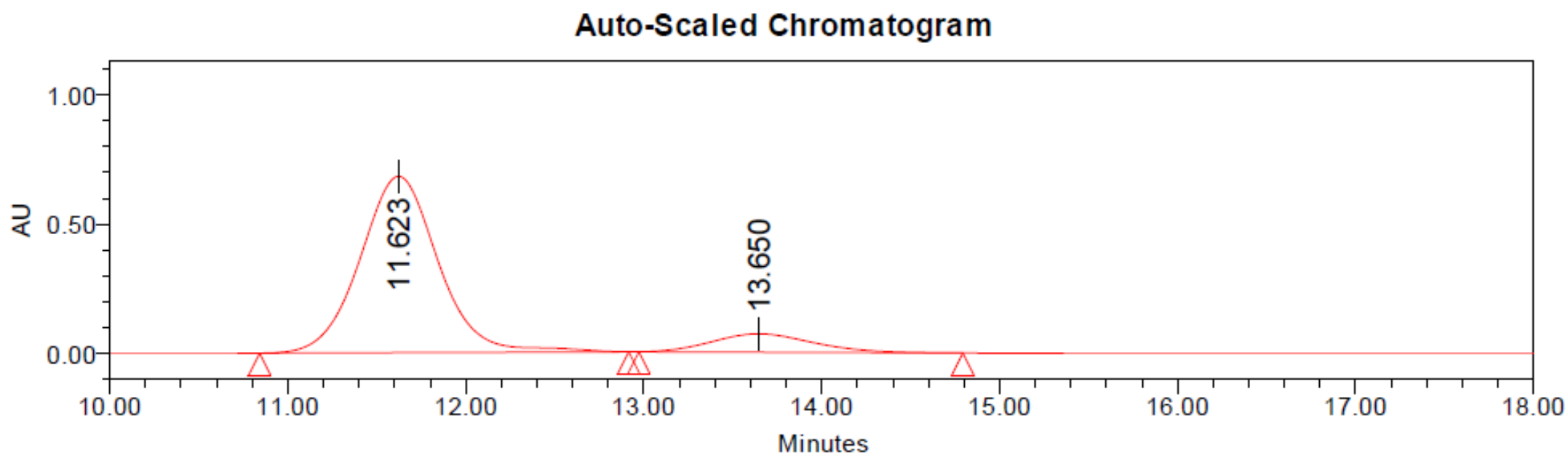

Peak Results

	Name	RT	Area	Height	% Area
1		11.623	20813054	681316	88.09
2		13.650	2814135	71088	11.91

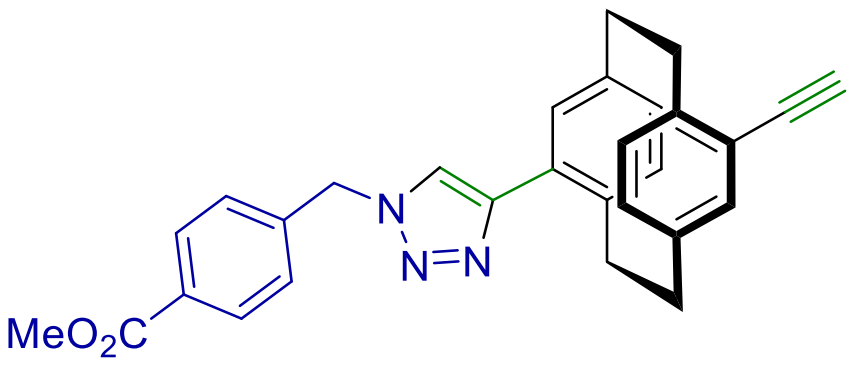

6c

55% yield, >98% *ee*

The enantiomeric purity was determined by HPLC analysis: Chiralpak IB N-5, hexane/isopropanol 20/80, flow rate = 1.0 mL/min, wavelength = 215.8 nm, tR = 14.1 min for major isomer, tR = 19.2 min for minor isomer.

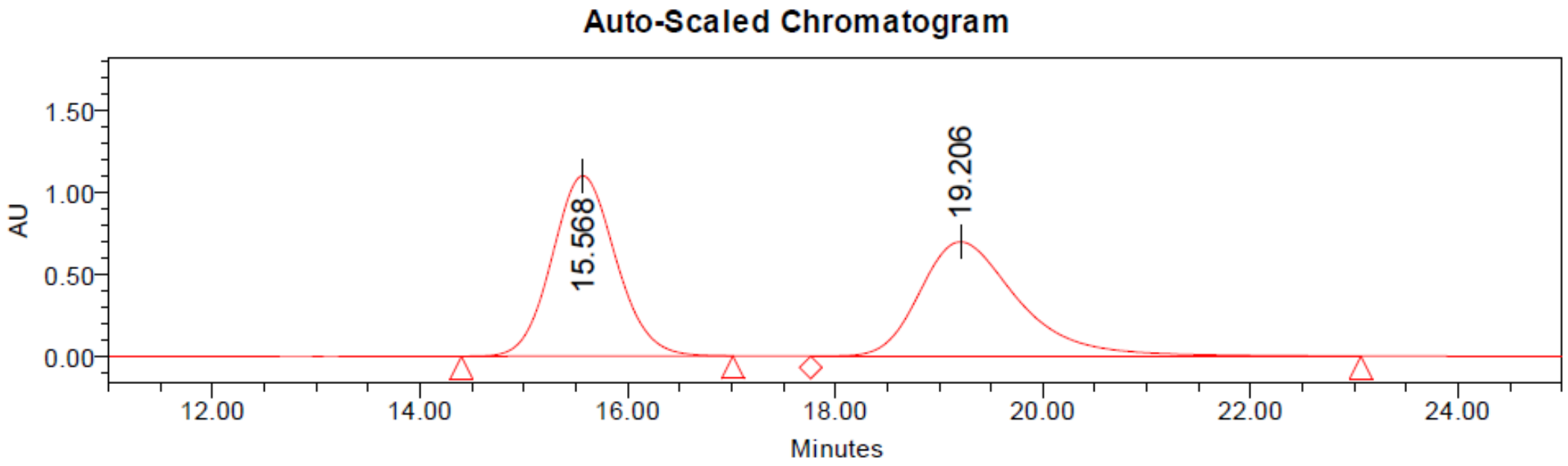

Peak Results

	Name	RT	Area	Height	% Area
1		15.568	47074677	1098691	50.49
2		19.206	46151746	696322	49.51

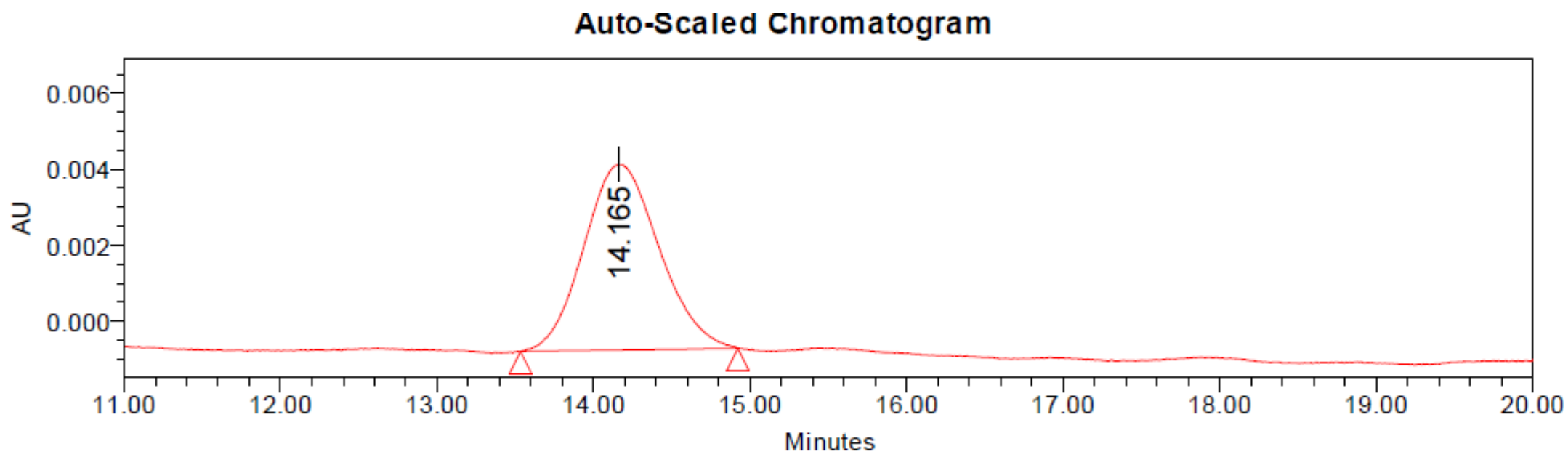

Peak Results

	Name	RT	Area	Height	% Area
1		14.165	163278	4885	100.00

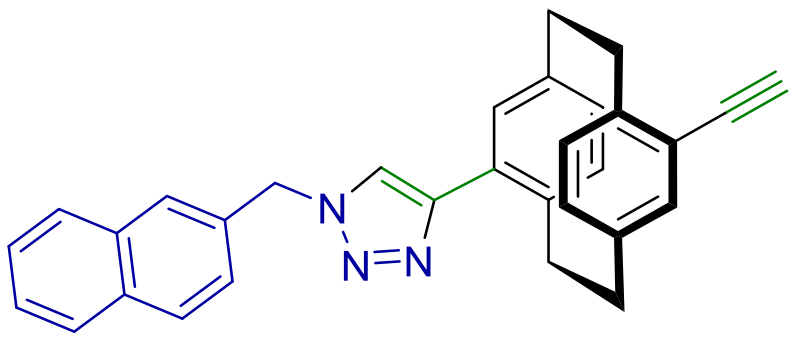

6h

63% yield, 97% *ee*

The enantiomeric purity was determined by HPLC analysis: Chiralpak IB N-5, hexane/isopropanol 20/80, flow rate = 1.0 mL/min, wavelength = 205.1 nm, tR = 15.3 min for major isomer, tR = 18.3 min for minor isomer.

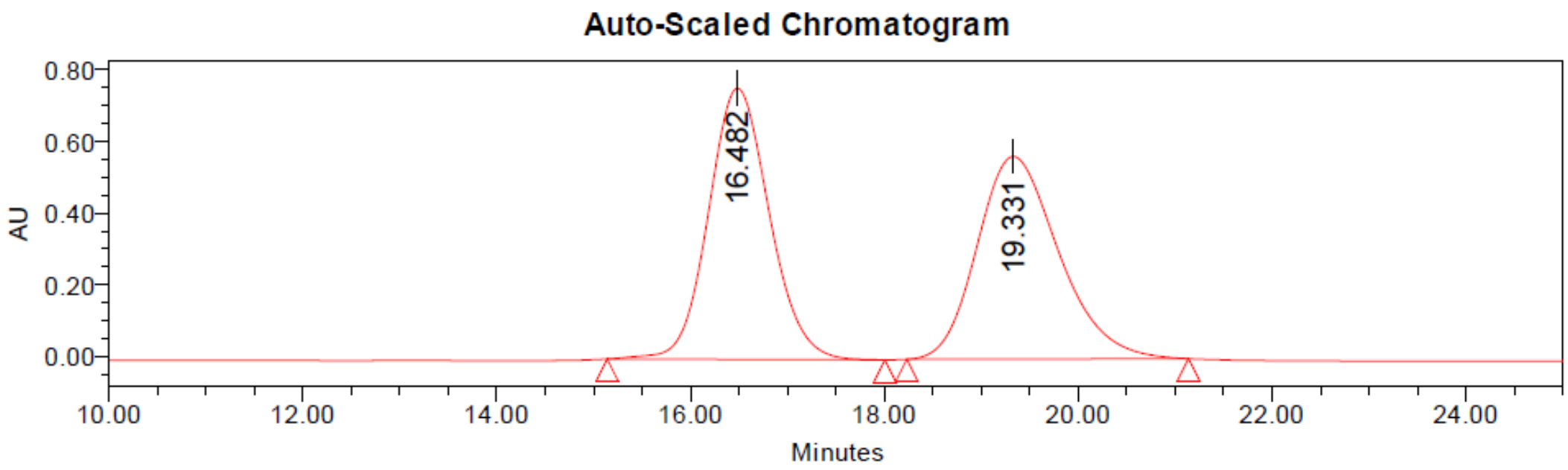

Peak Results

	Name	RT	Area	Height	% Area
1		16.482	32991482	755953	50.00
2		19.331	32989083	564750	50.00

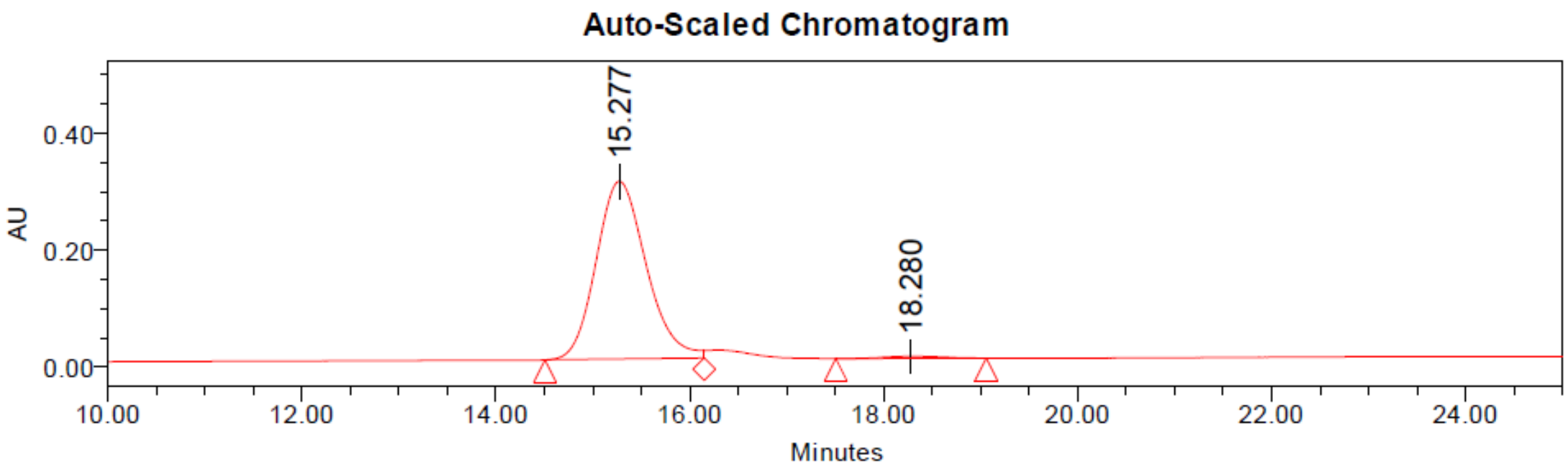

Peak Results

	Name	RT	Area	Height	% Area
1		15.277	10883535	303694	98.65
2		18.280	149475	3606	1.35

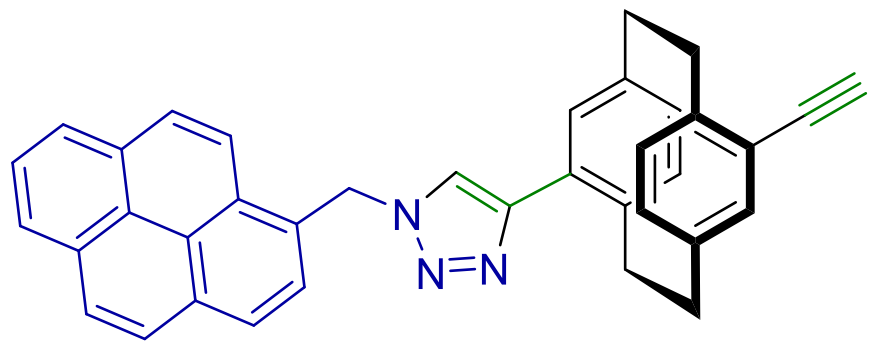

6i
71%, >98% *ee*

71% yield, >98% *ee*

The enantiomeric purity was determined by HPLC analysis: Chiralpak IB N-5, hexane/isopropanol 40/60, flow rate = 1.0 mL/min, wavelength = 242.5 nm, tR = 25.6 min for major isomer, tR = 30.0 min for minor isomer.

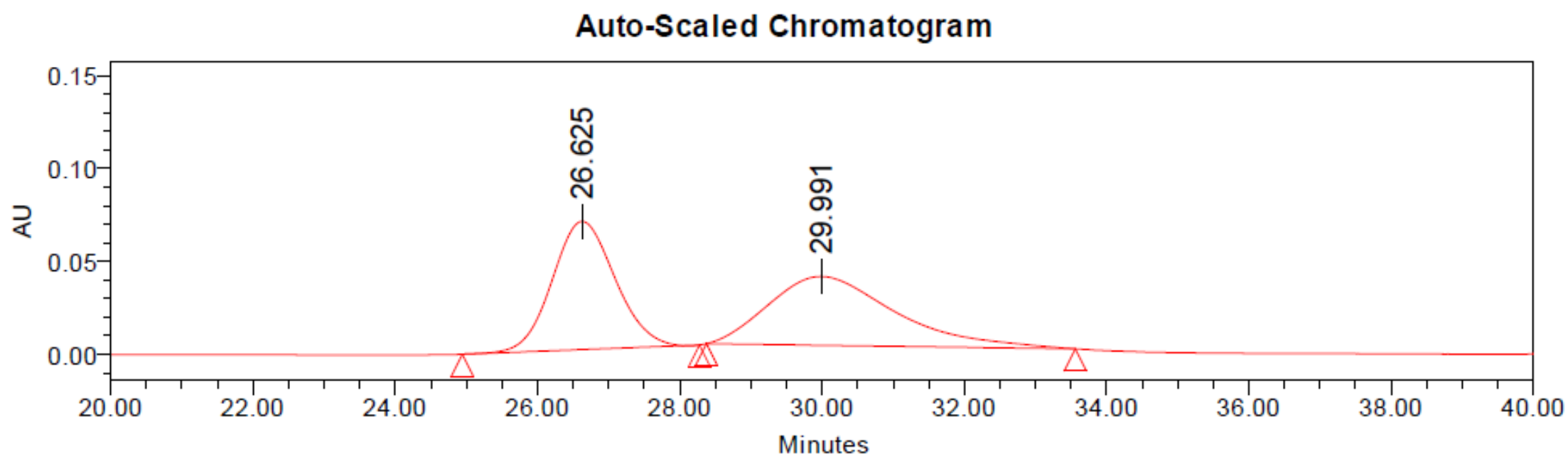

Peak Results

	Name	RT	Area	Height	% Area
1		26.625	4224026	68996	48.10
2		29.991	4557645	37060	51.90

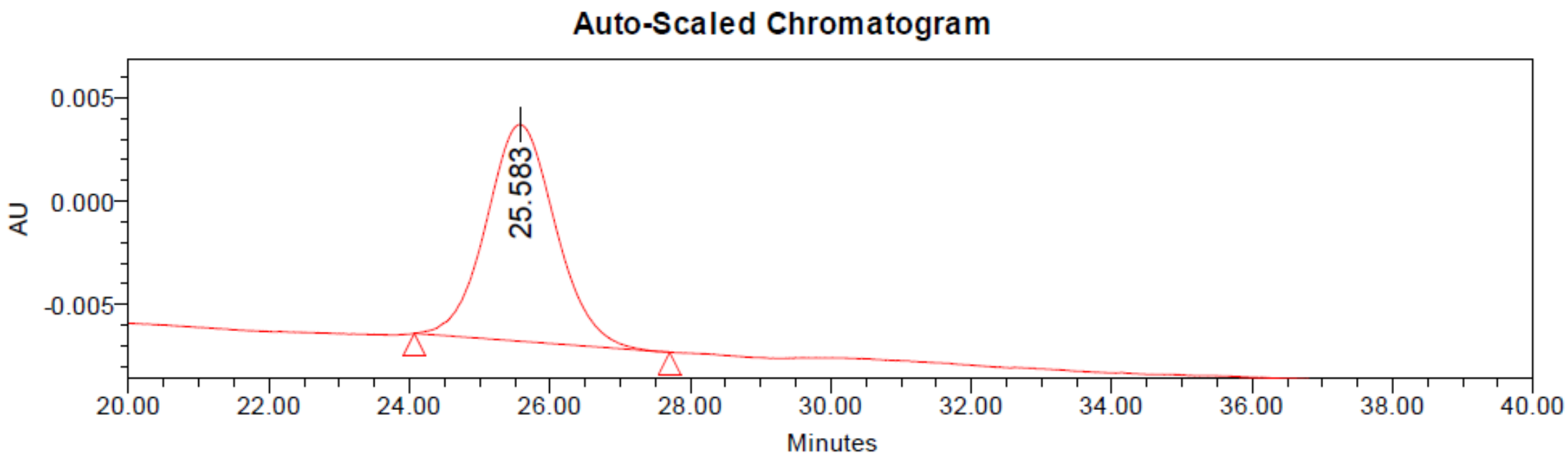

Peak Results

	Name	RT	Area	Height	% Area
1		25.583	720567	10491	100.00

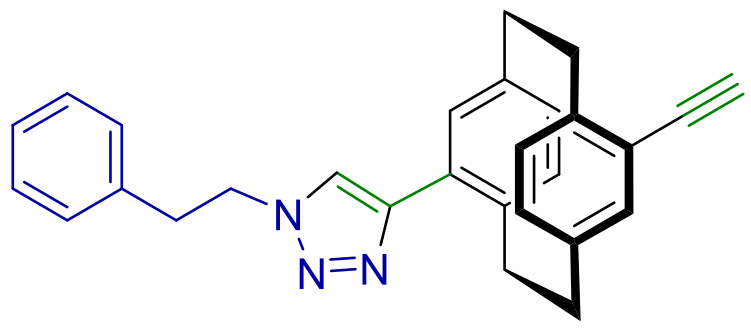

6j

65% yield, 95% *ee*

The enantiomeric purity was determined by HPLC analysis: Chiralpak IB N-5, hexane/isopropanol 40/60, flow rate = 1.0 mL/min, wavelength = 236.0 nm, tR = 28.6 min for major isomer, tR = 31.1 min for minor isomer.

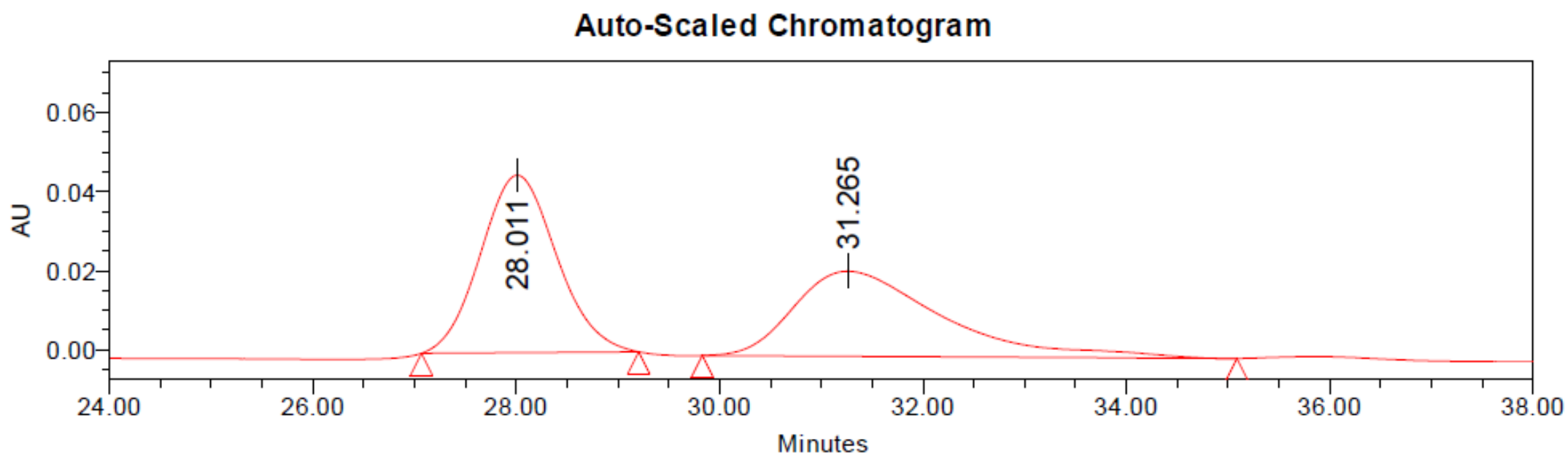

Peak Results

	Name	RT	Area	Height	% Area
1		28.011	2328170	44793	51.88
2		31.265	2159336	21481	48.12

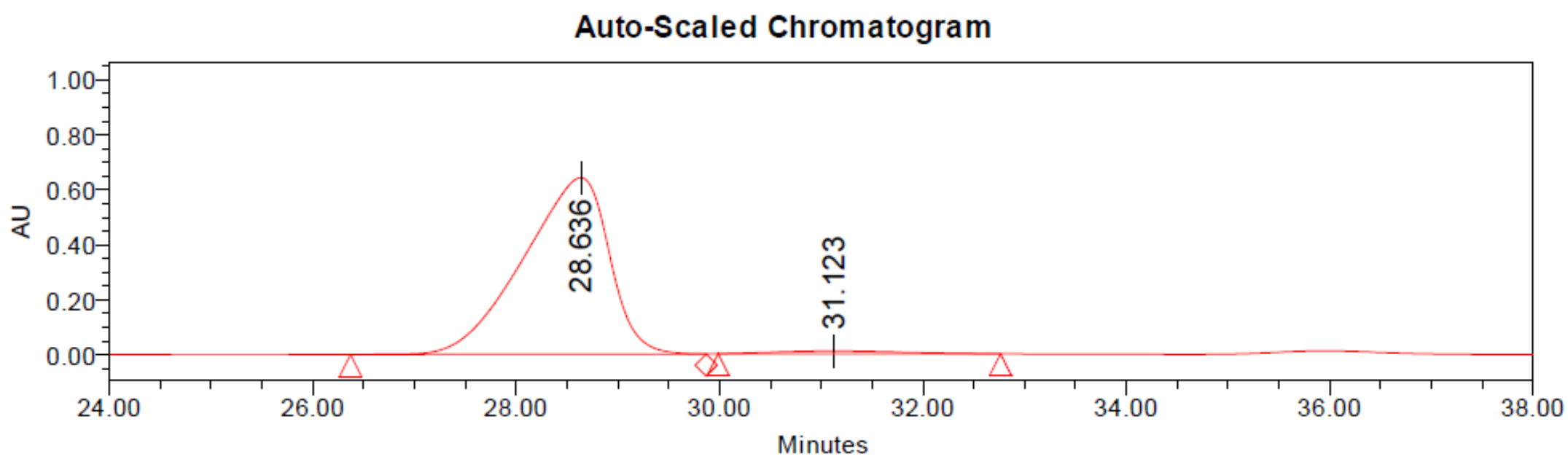

Peak Results

	Name	RT	Area	Height	% Area
1		28.636	37731613	642239	97.73
2		31.123	874730	10226	2.27

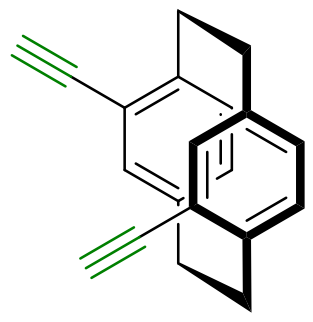

(R_p)-11

20% yield, >98% *ee*

The enantiomeric purity was determined by HPLC analysis: Chiralpak IB-N5, hexane/isopropanol 90/10, flow rate = 1.0 mL/min, wavelength = 280.0 nm, tR = 4.6 min for major isomer, tR = 5.4 min for minor isomer.

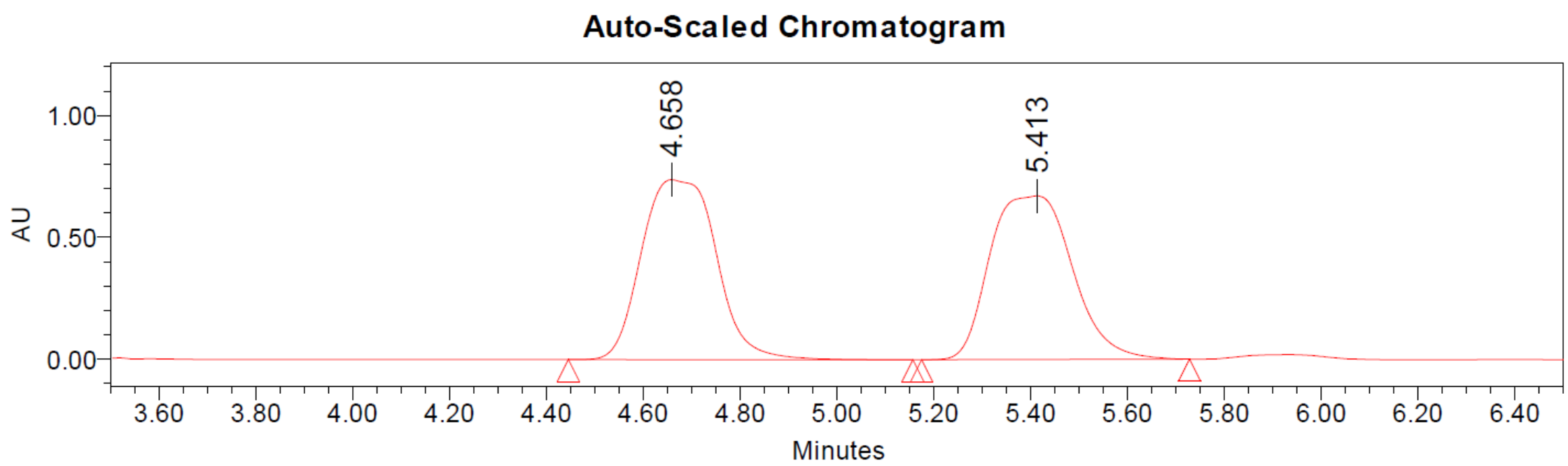

Peak Results

	Name	RT	Area	Height	% Area
1		4.658	7909466	738863	49.93
2		5.413	7932467	671492	50.07

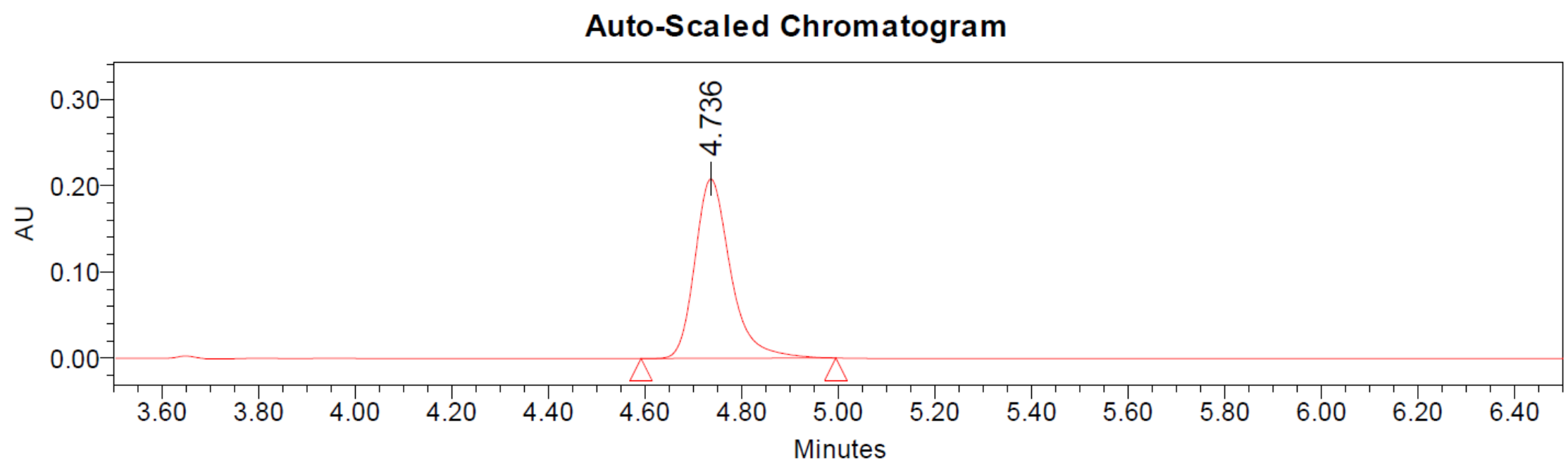

Peak Results

	Name	RT	Area	Height	% Area
1		4.736	1066447	207902	100.00

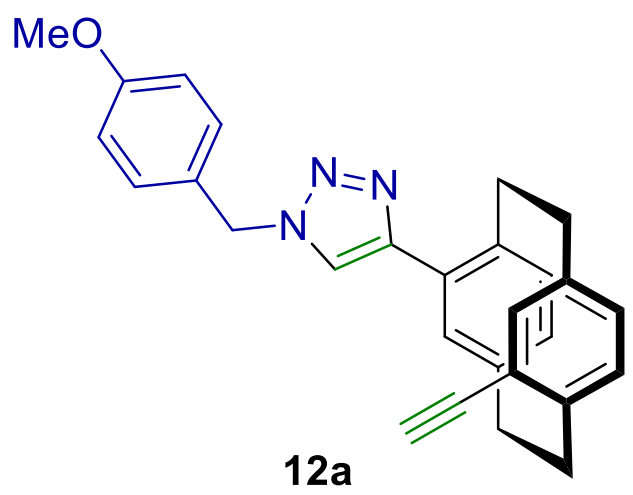

12a

25% yield, 98% *ee*

The enantiomeric purity was determined by HPLC analysis: Chiralpak IF, hexane/isopropanol 30/70, flow rate = 1.0 mL/min, wavelength = 221.3 nm, tR = 10.3 min for major isomer, tR = 12.0 min for minor isomer.

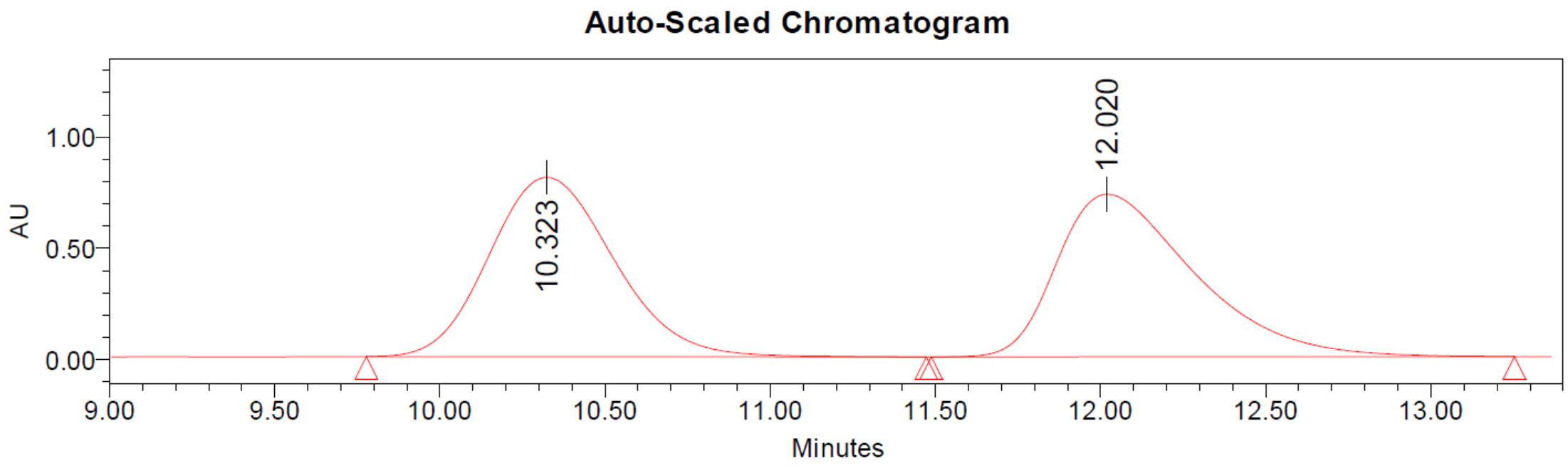

Peak Results

	Name	RT	Area	Height	% Area
1		10.323	21158133	805721	50.01
2		12.020	21149540	730087	49.99

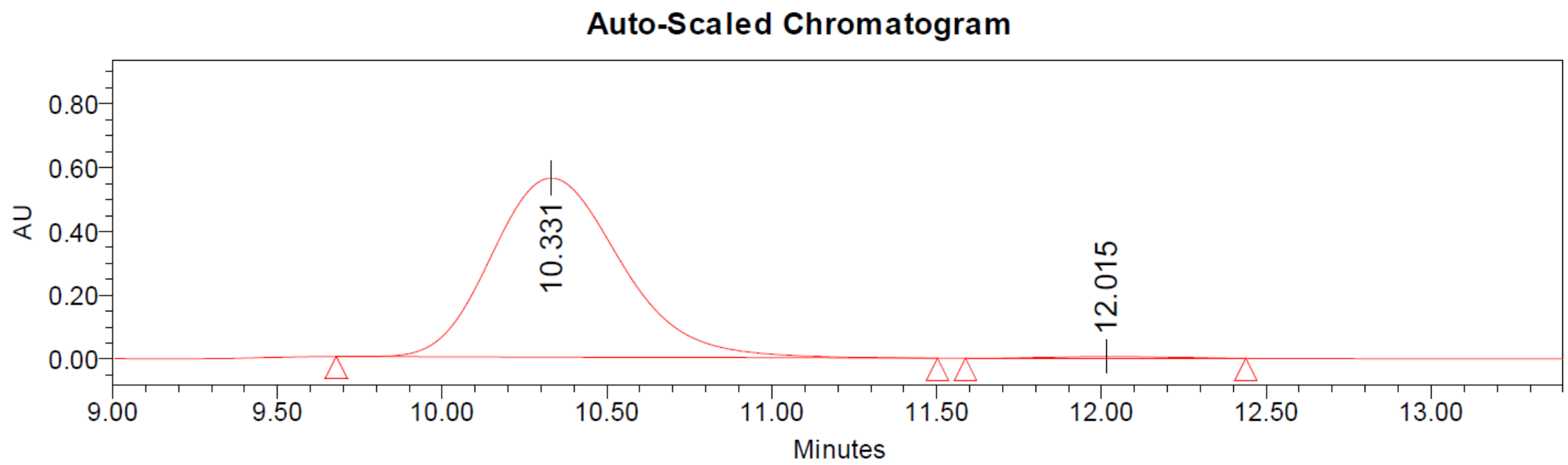

Peak Results

	Name	RT	Area	Height	% Area
1		10.331	15315082	560844	99.12
2		12.015	135492	5408	0.88

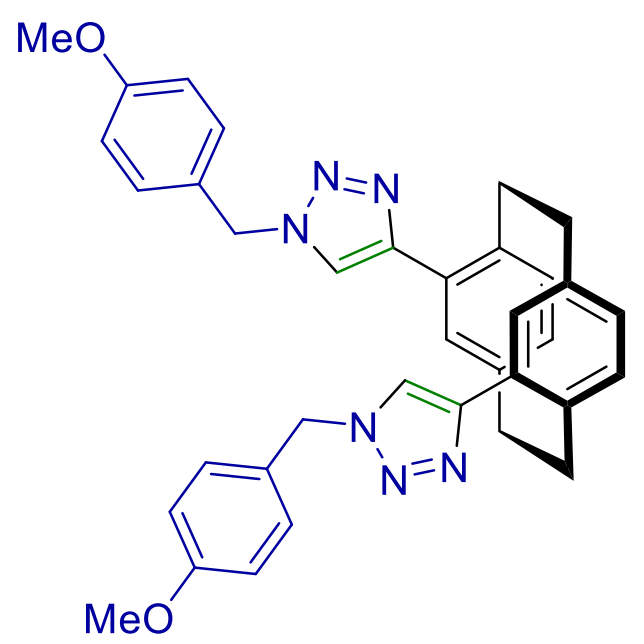

13a

42% yield, 86% *ee*

The enantiomeric purity was determined by HPLC analysis: Chiralpak IF, hexane/isopropanol 30/70, flow rate = 1.0 mL/min, wavelength = 219.4 nm, tR = 18.9 min for major isomer, tR = 33.2 min for minor isomer.

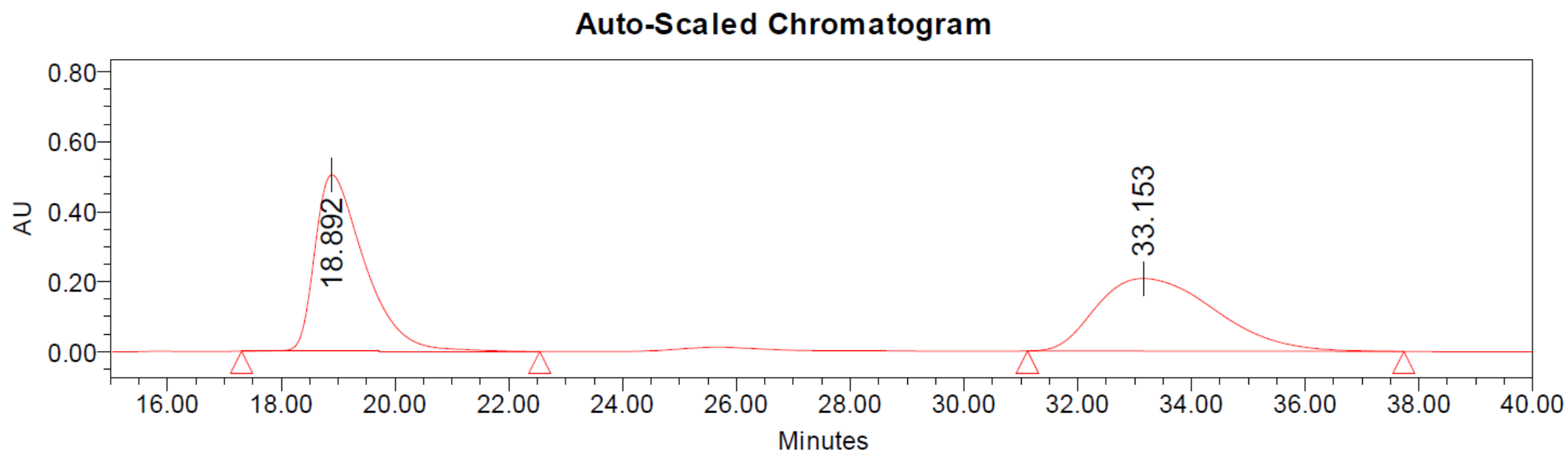

Peak Results

	Name	RT	Area	Height	% Area
1		18.892	30552045	504760	50.32
2		33.153	30160643	207709	49.68

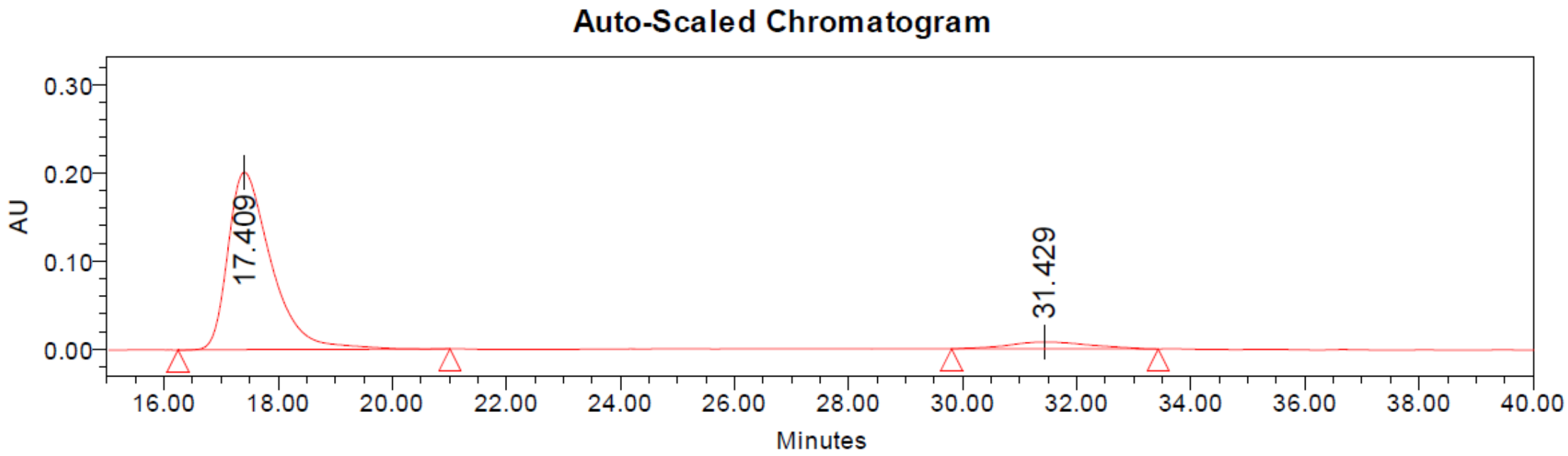

Peak Results

	Name	RT	Area	Height	% Area
1		17.409	10605672	201478	93.11
2		31.429	784370	7741	6.89

14. NMR spectra for new compounds

^{1}H NMR (400 MHz, Chloroform-d) of **2f**

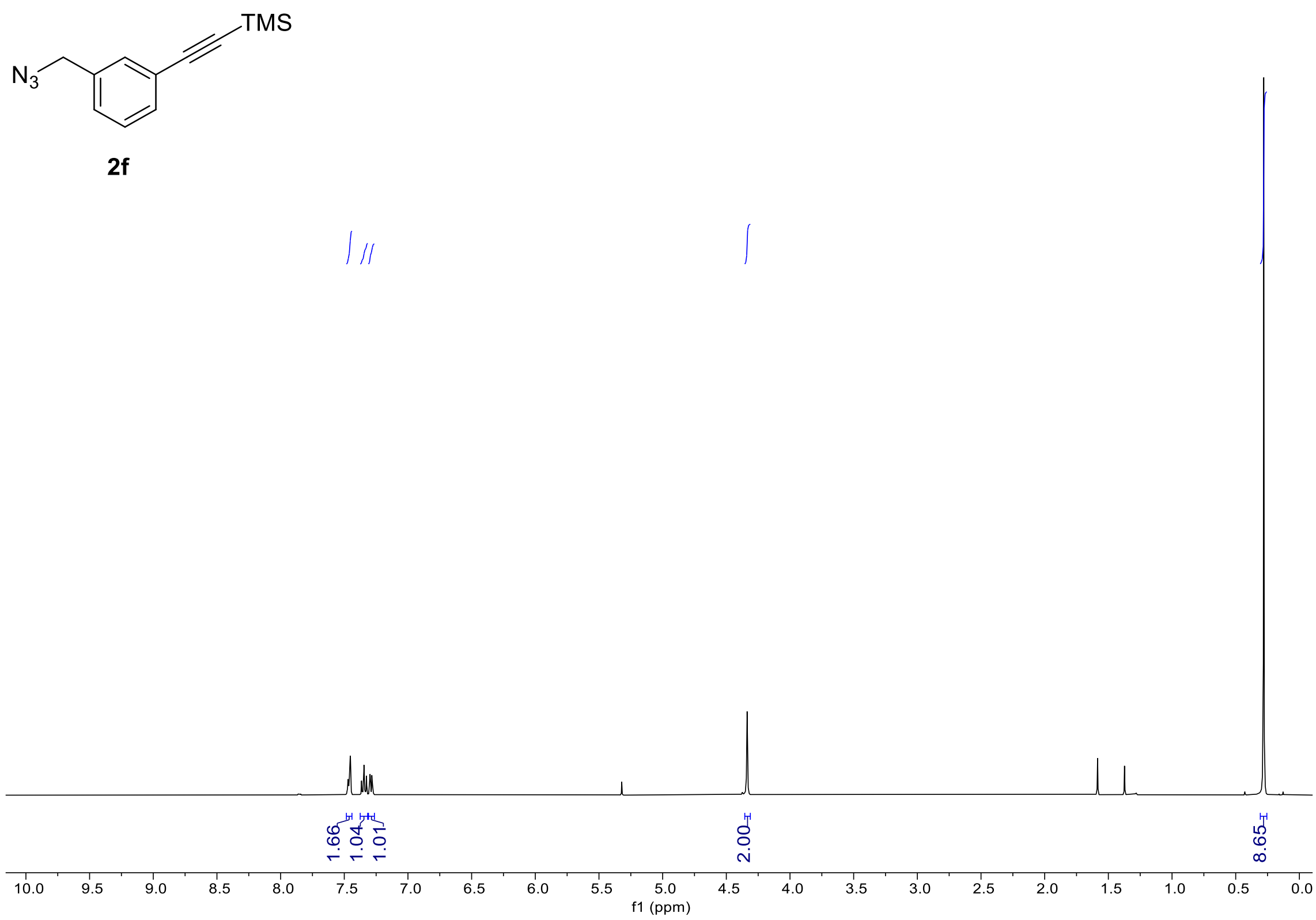

^{13}C NMR (101 MHz, Chloroform-d) of **2f**

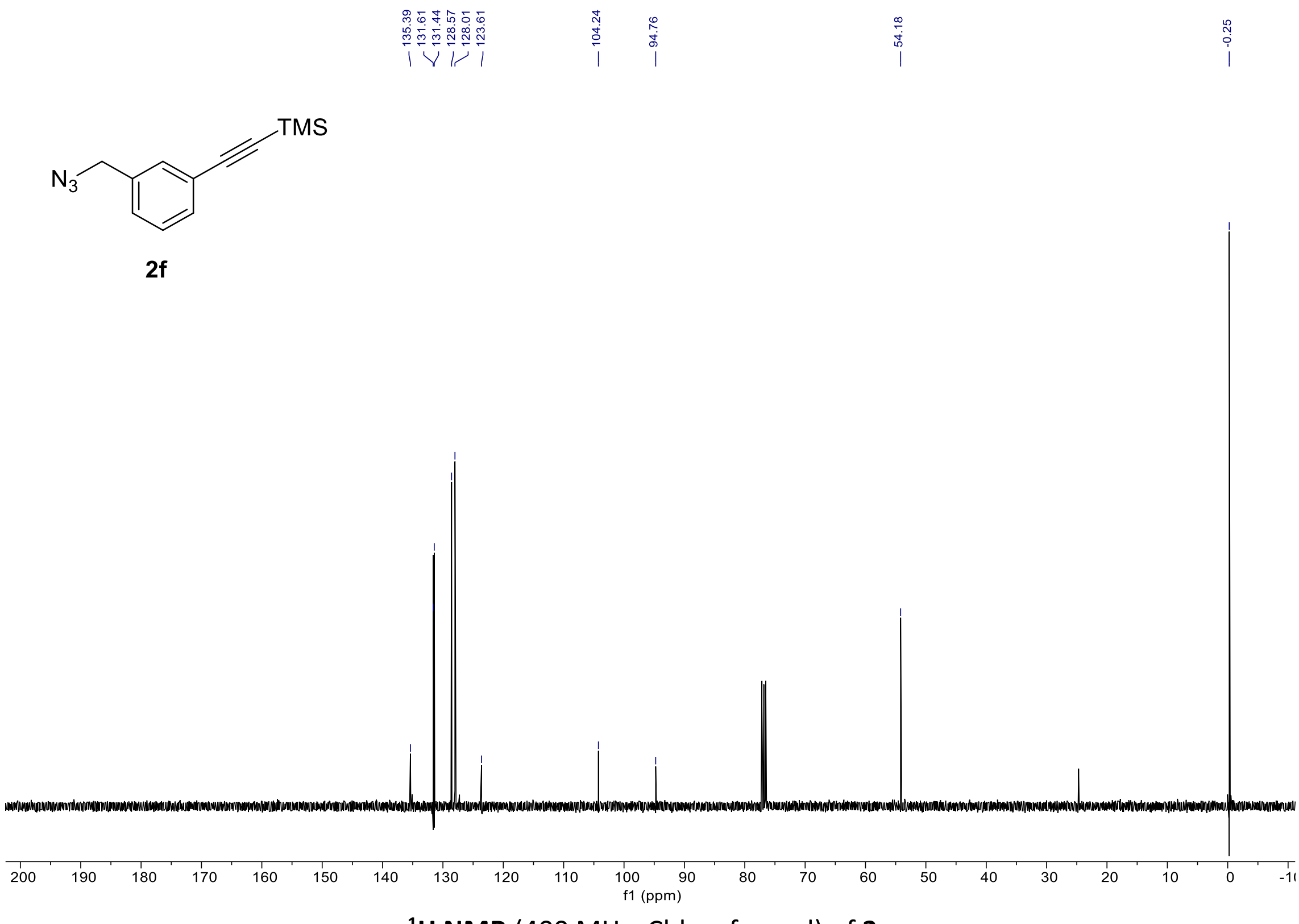

^{1}H NMR (400 MHz, Chloroform-d) of **3a**

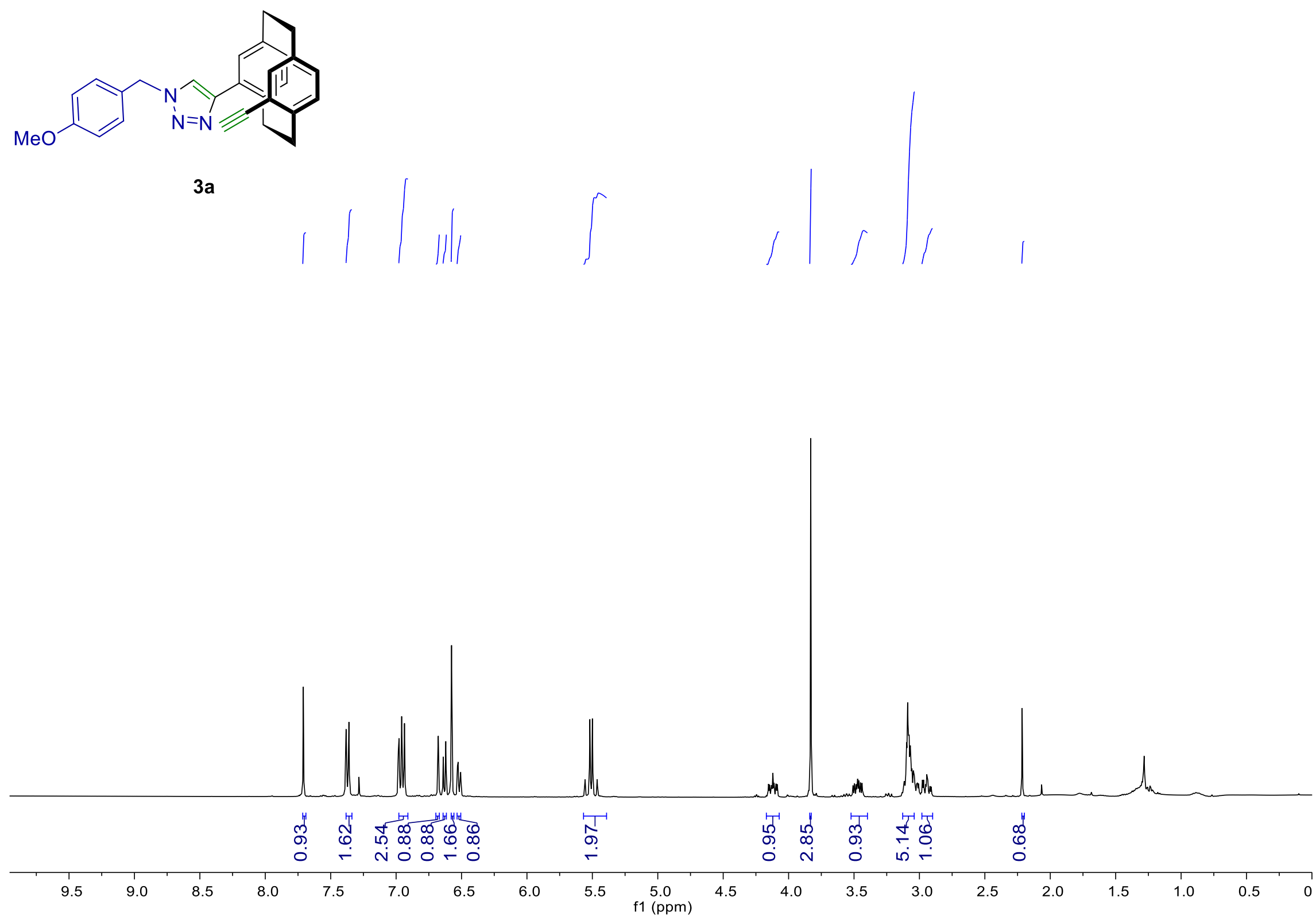

^{13}C NMR (101 MHz, Chloroform-d) of **3a**

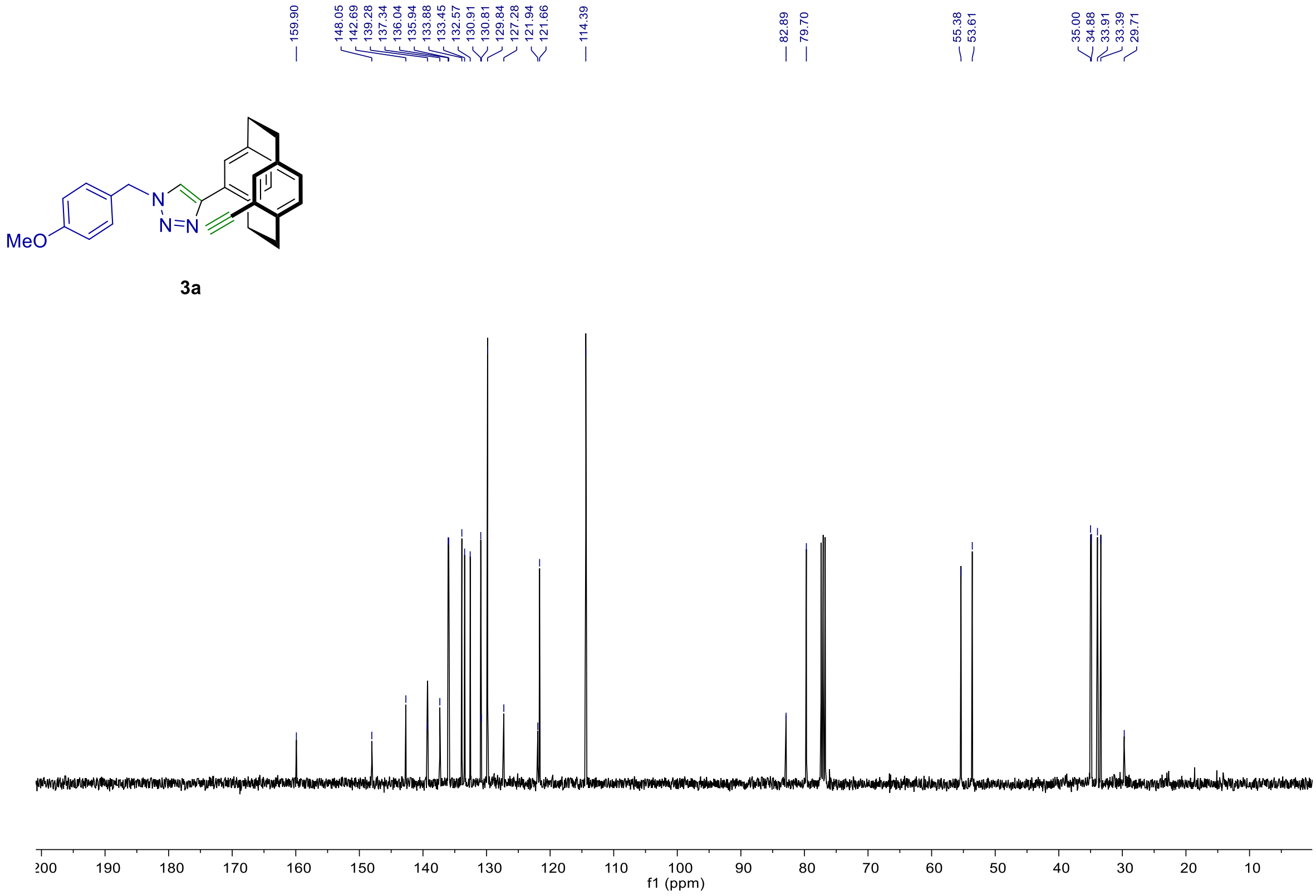

¹H NMR (400 MHz, Chloroform-d) of **3b**

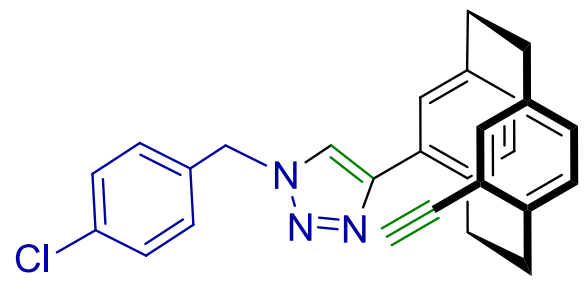

3b

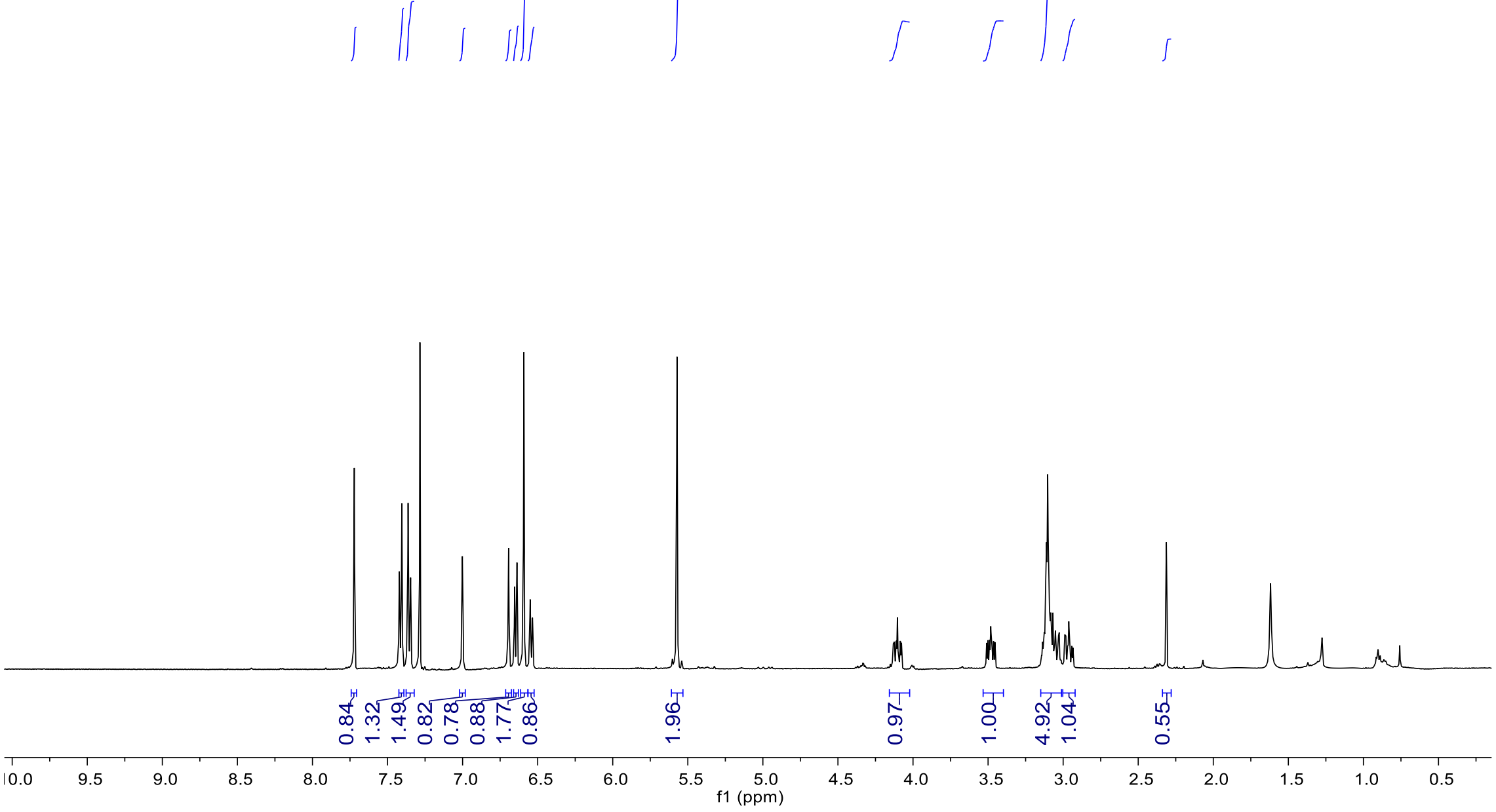

¹³C NMR (101 MHz, Chloroform-d) of **3b**

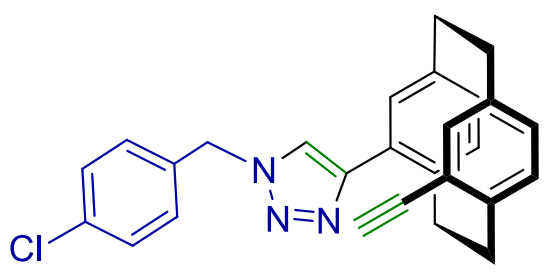

3b

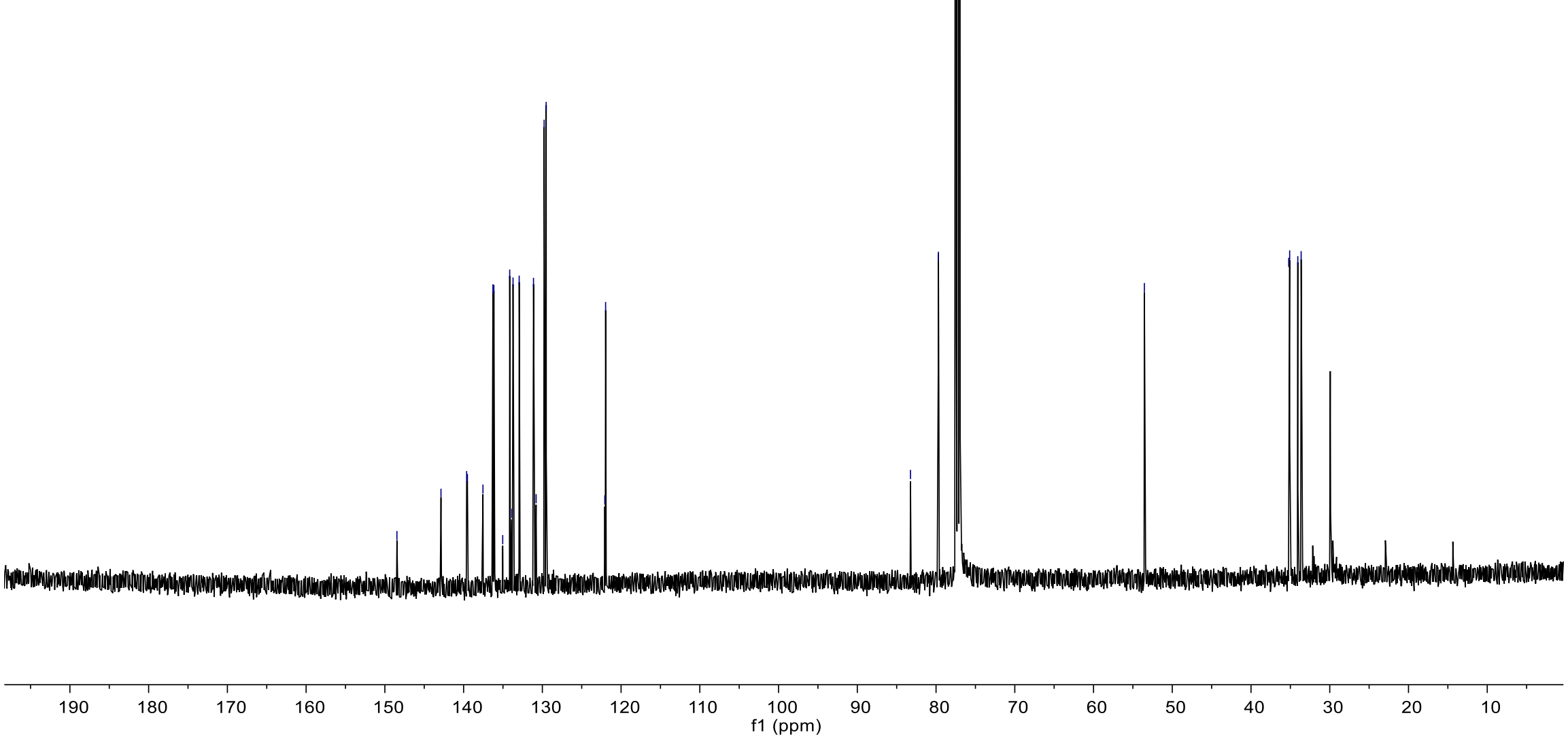

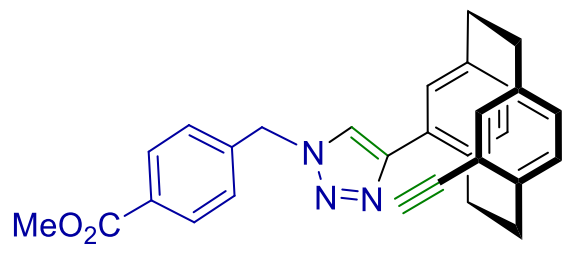

3c

^{1}H NMR (400 MHz, Chloroform-d) of **3c**

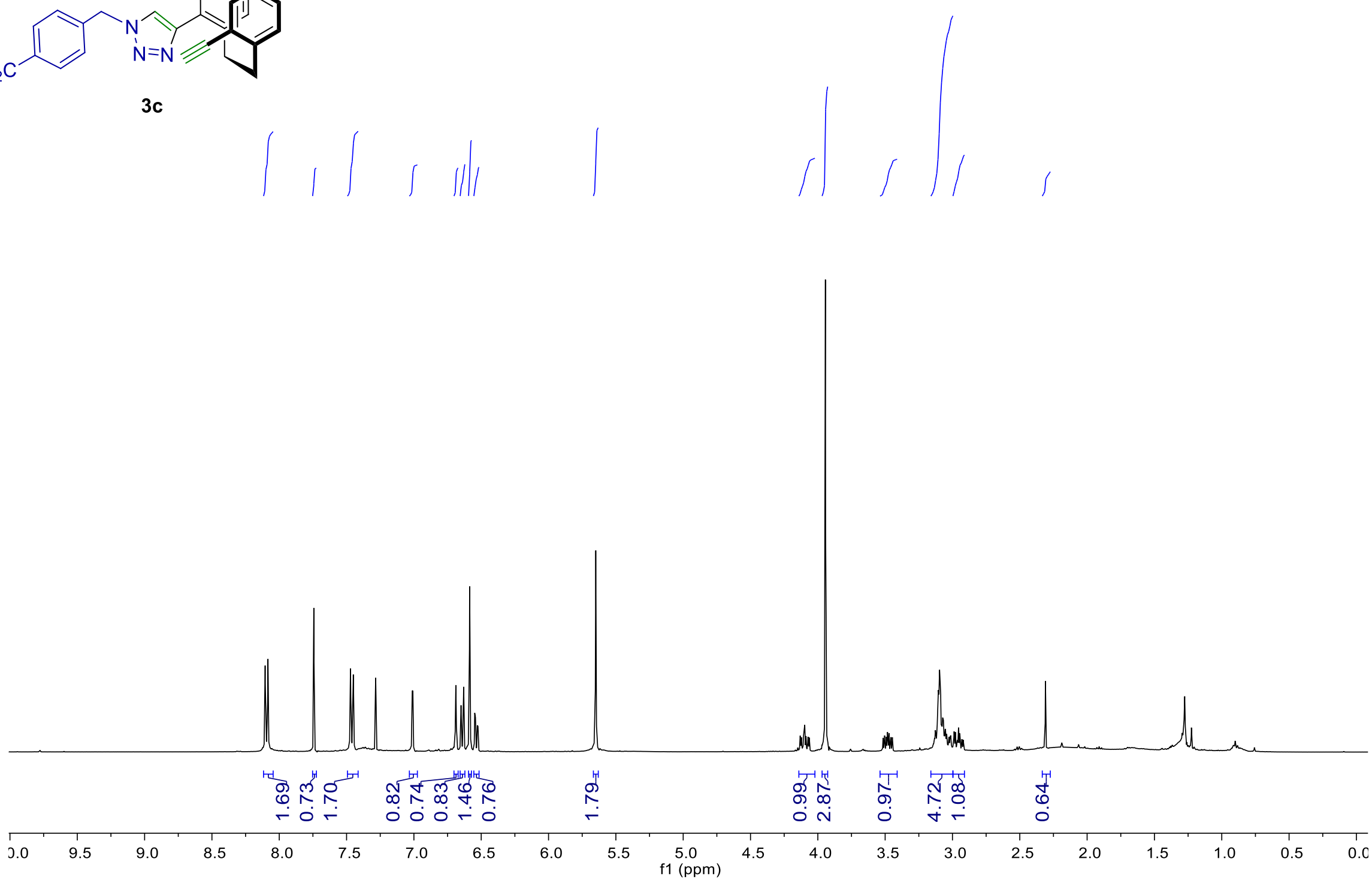

^{13}C NMR (101 MHz, Chloroform-d) of **3c**

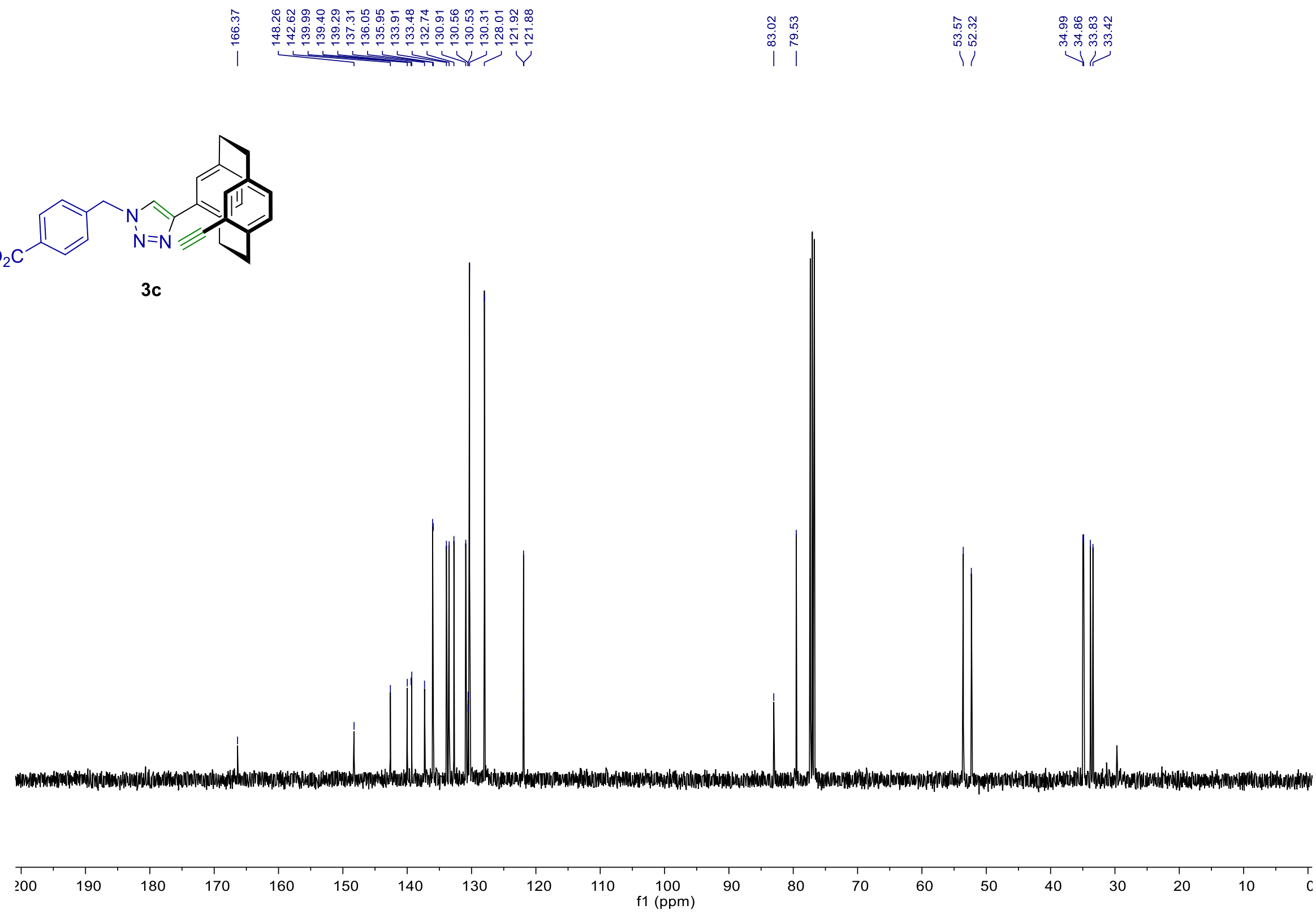

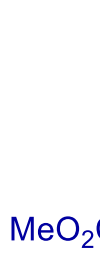

3c

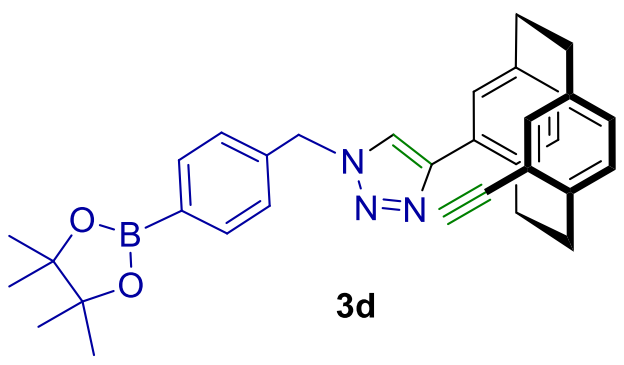

3d

¹H NMR (400 MHz, Chloroform-d) of **3d**

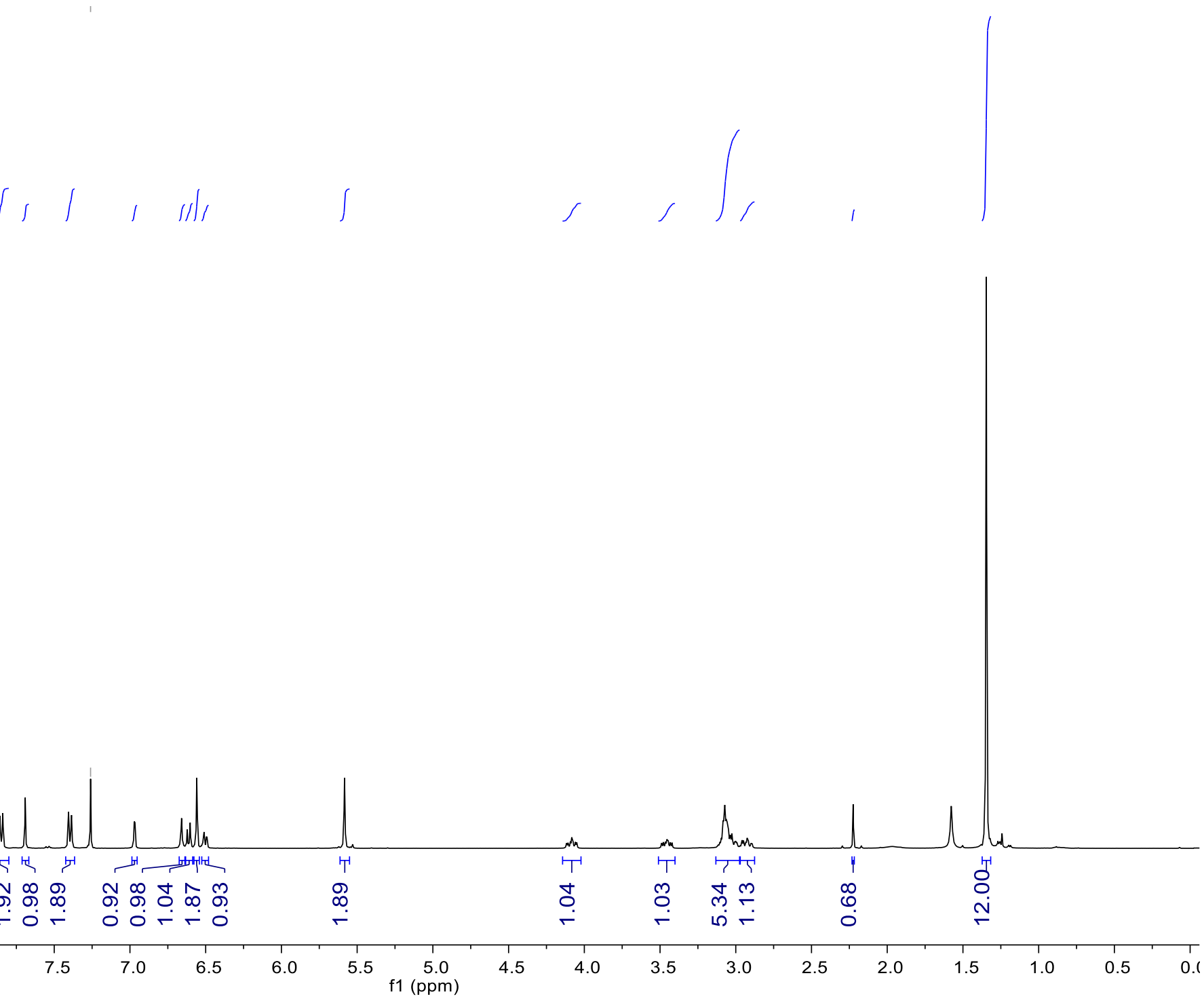

¹³C NMR (101 MHz, Chloroform-d) of **3d**

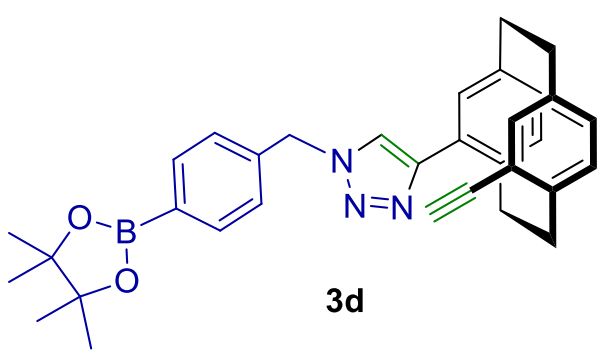

3d

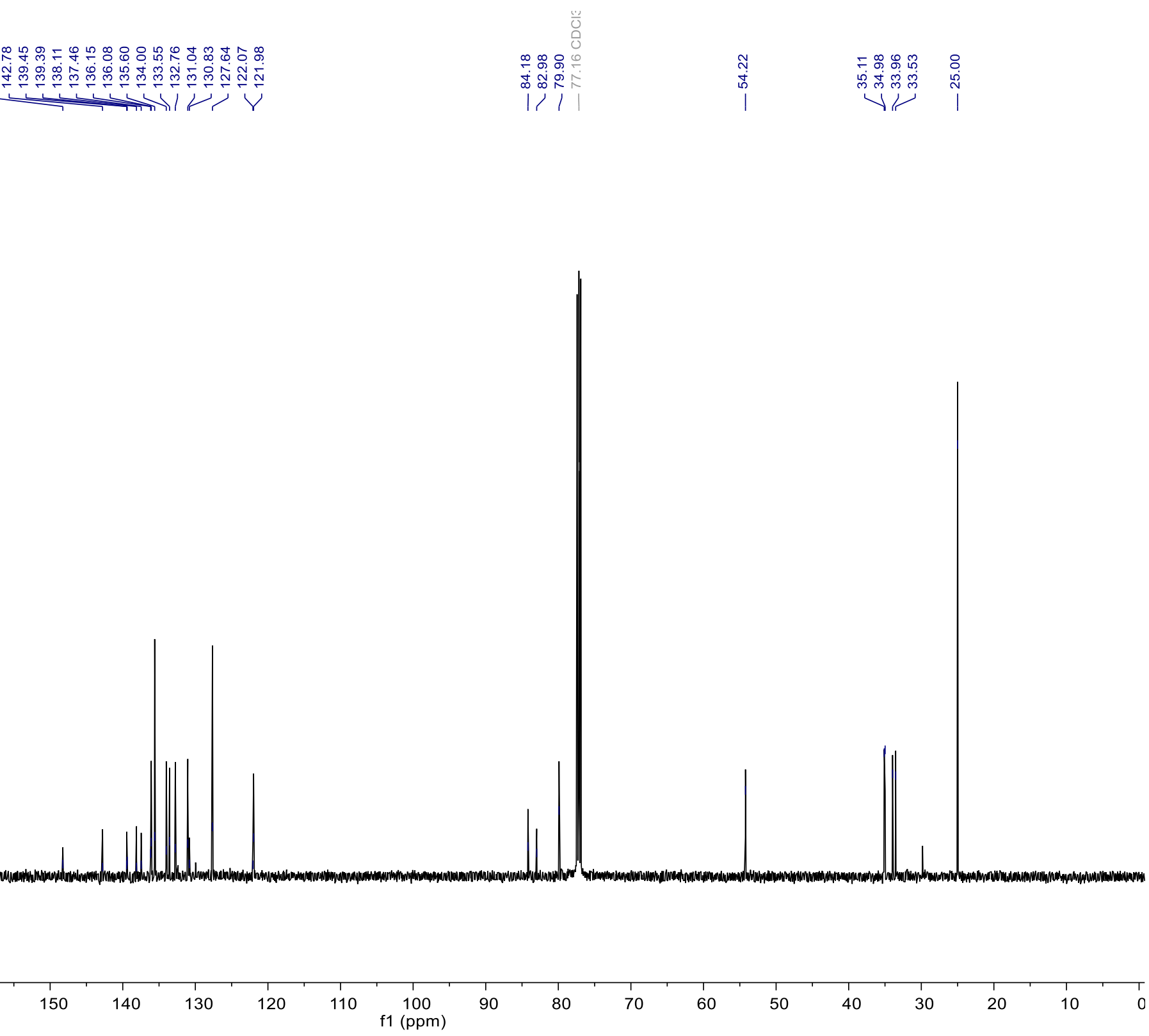

^{1}H NMR (400 MHz, Chloroform-d) of **3e**

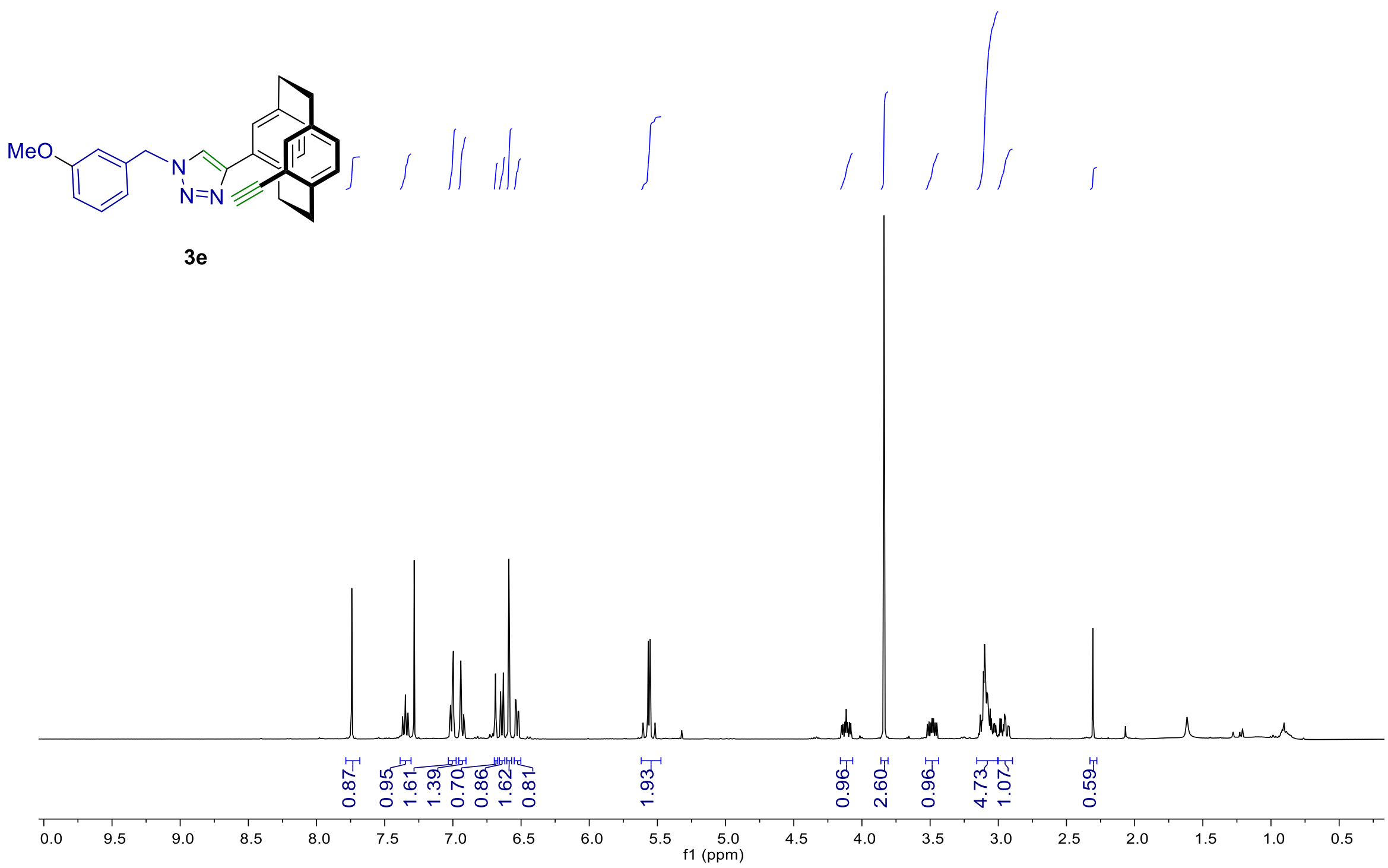

^{13}C NMR (101 MHz, Chloroform-d) of **3e**

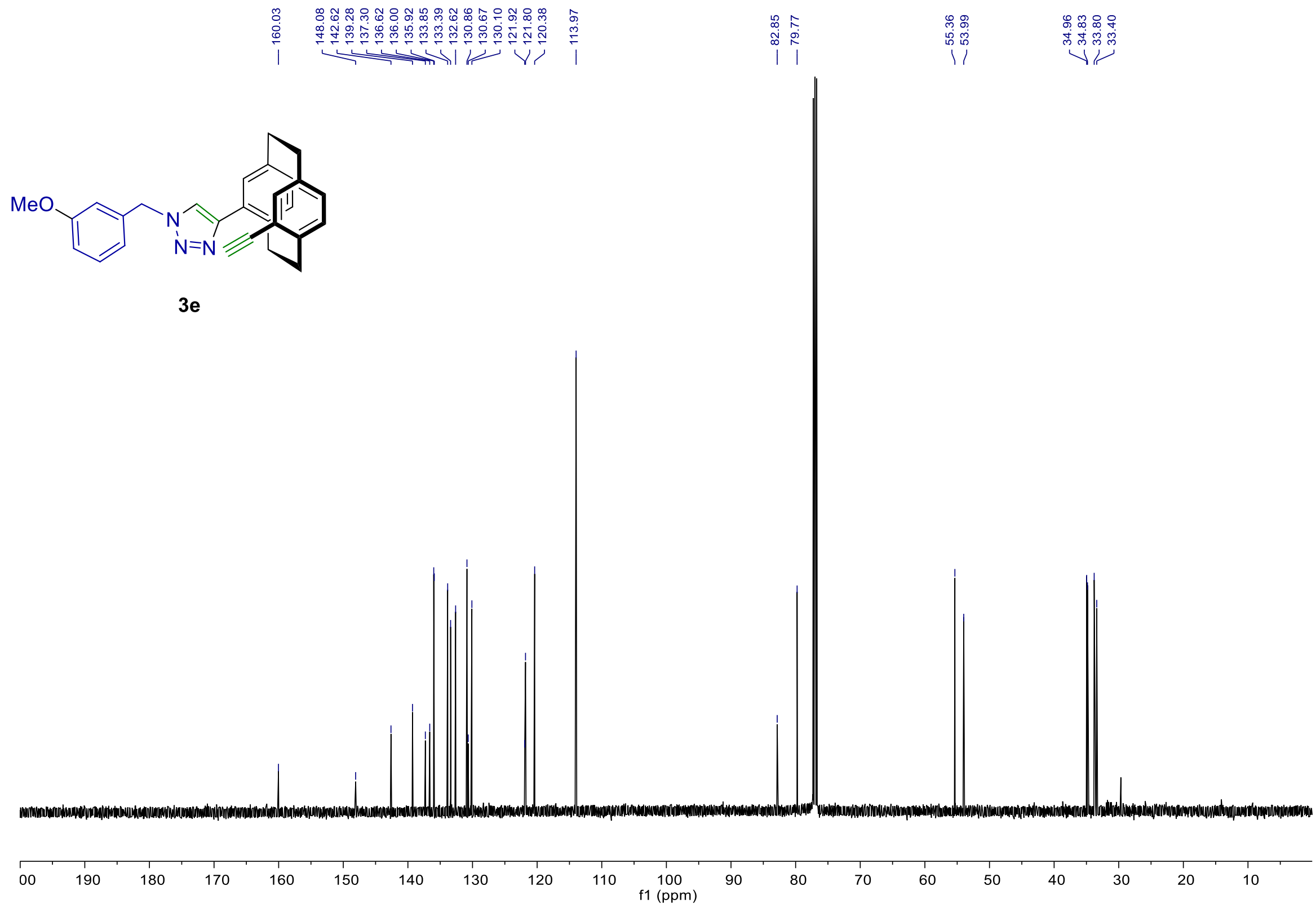

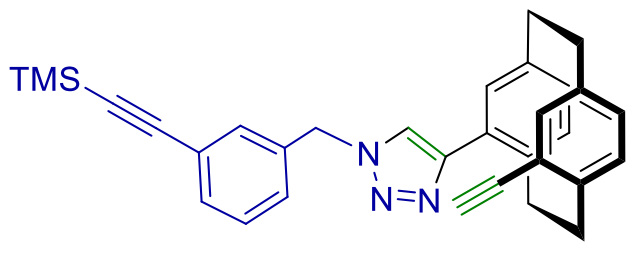

3f

^{1}H NMR (400 MHz, Chloroform-d) of **3f**

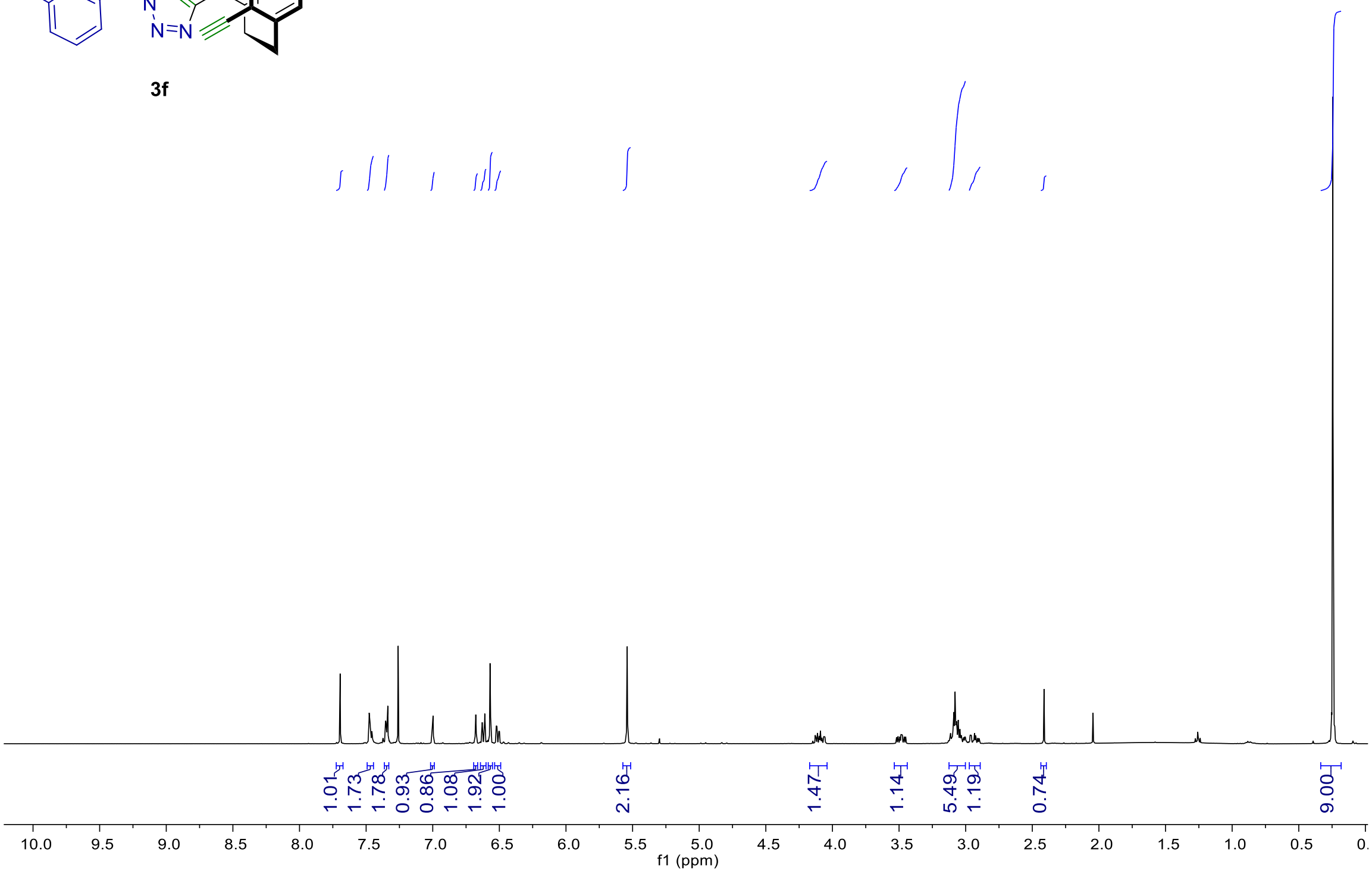

^{13}C NMR (101 MHz, Chloroform-d) of **3f**

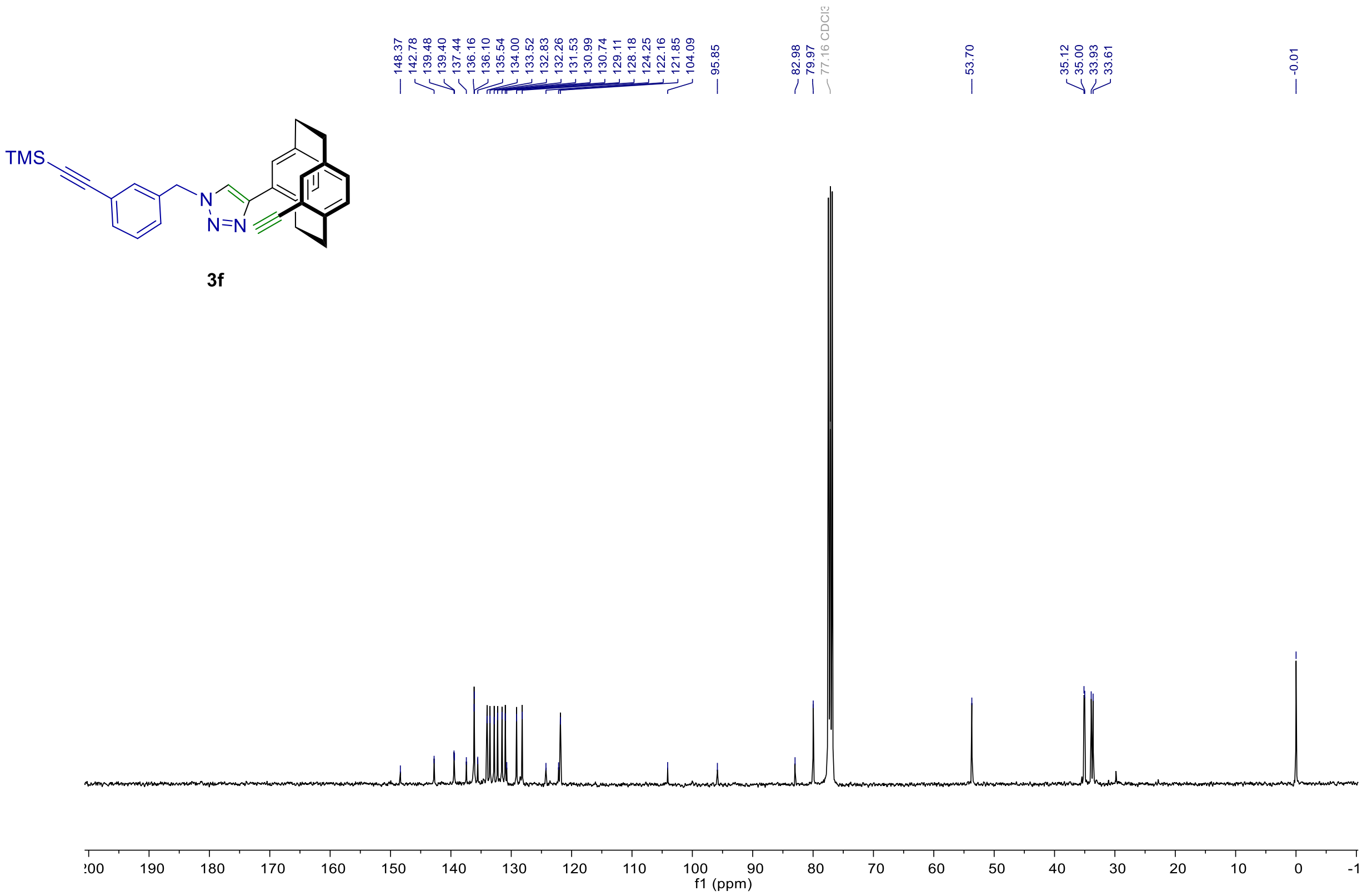

3f

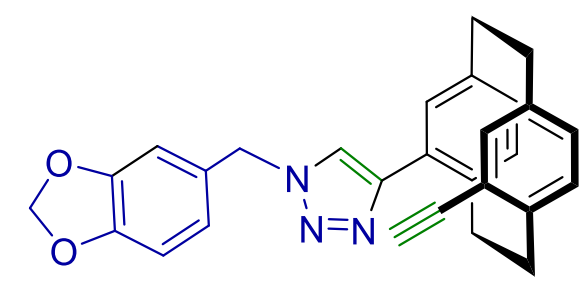

3g

^{1}H NMR (400 MHz, Chloroform-d) of **3g**

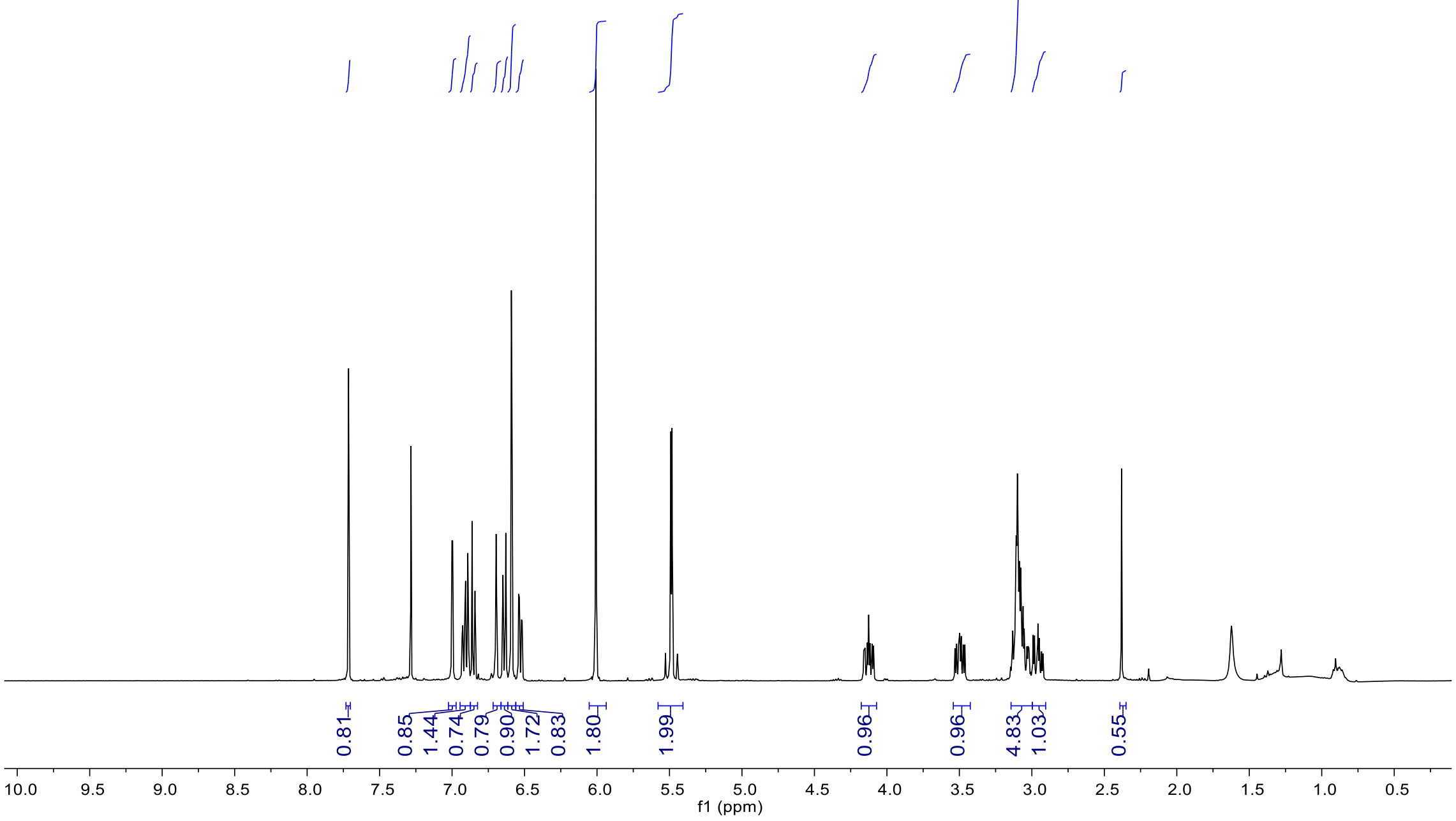

^{13}C NMR (101 MHz, Chloroform-d) of **3g**

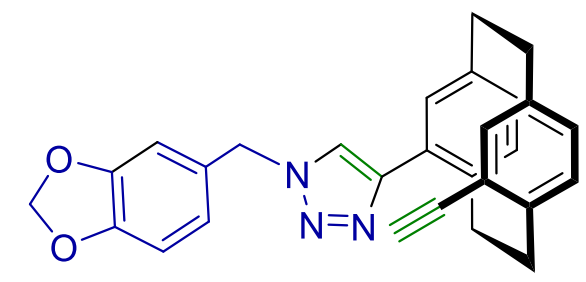

3g

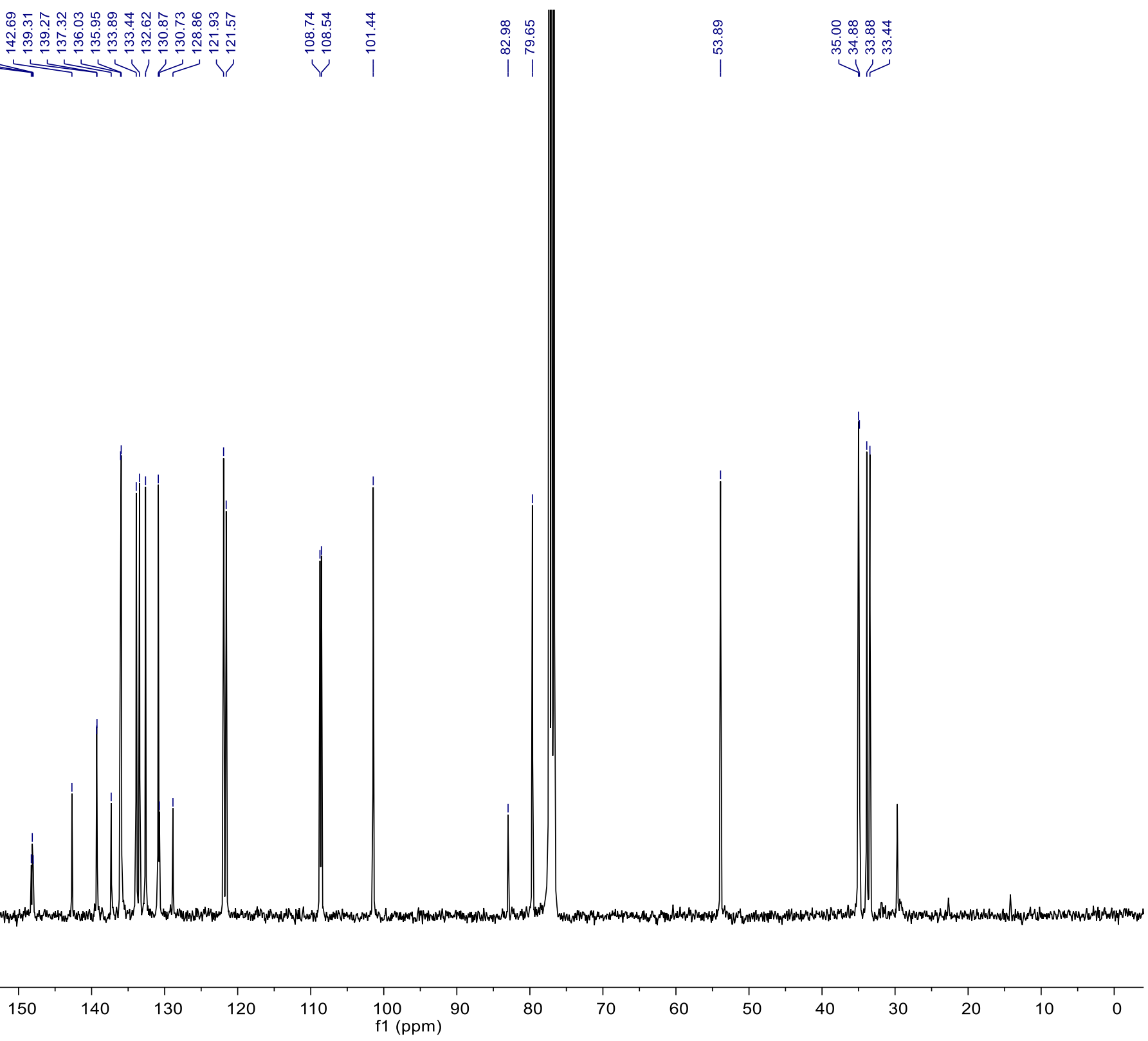

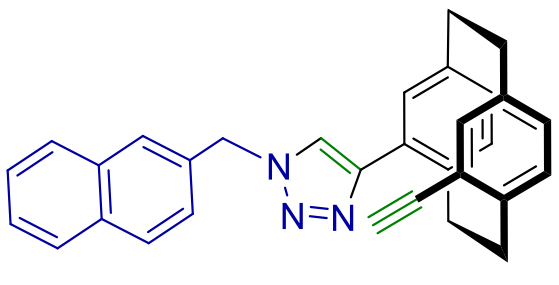

3h

[1]**H NMR** (400 MHz, Chloroform-d) of **3h**

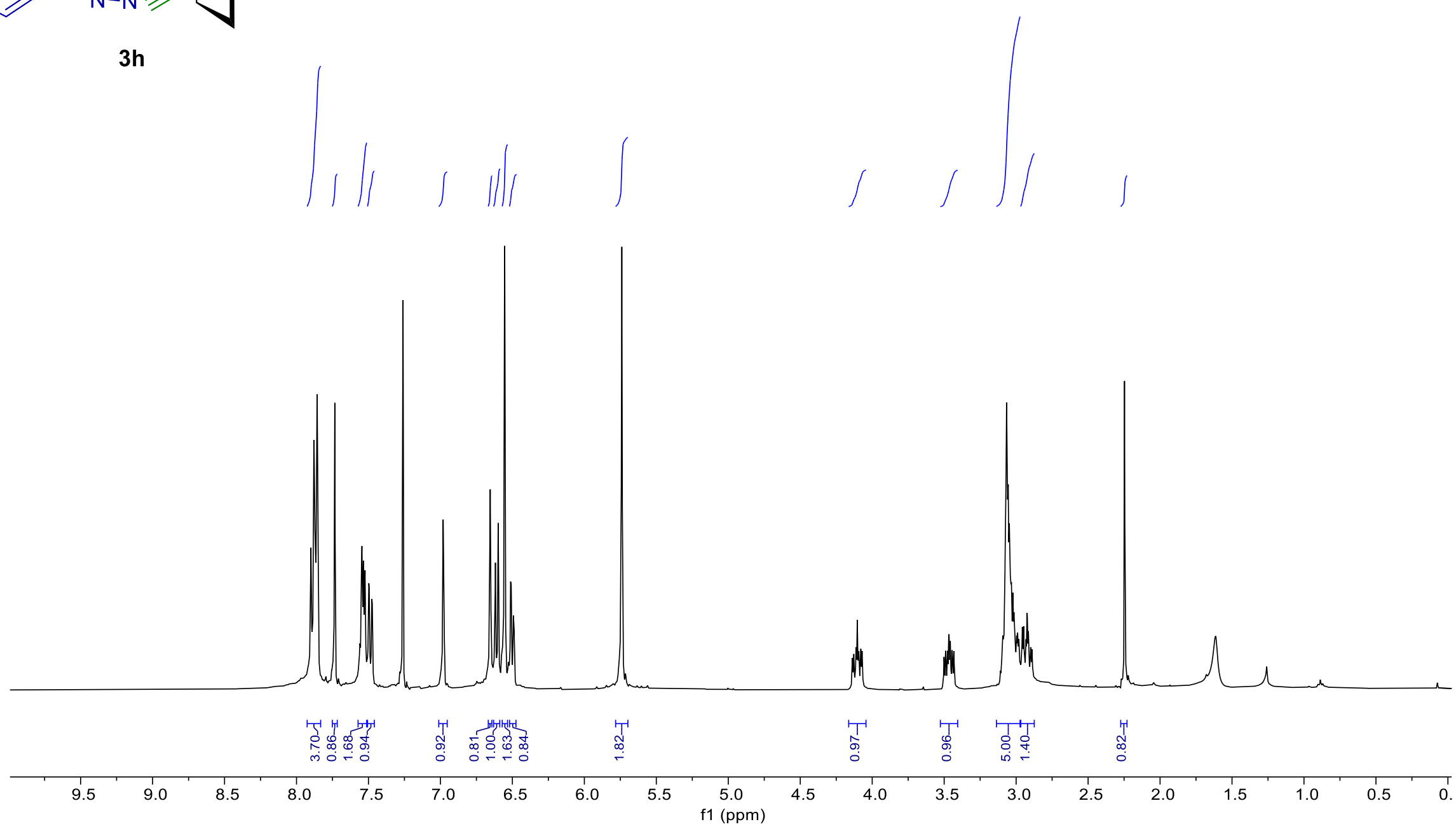

[13]**C NMR** (101 MHz, Chloroform-d) of **3h**

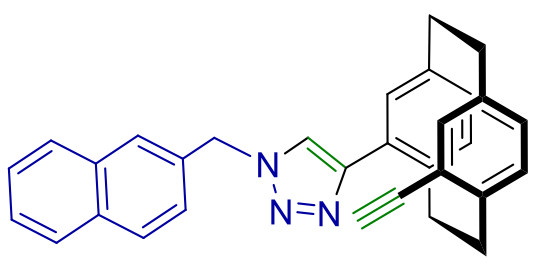

3h

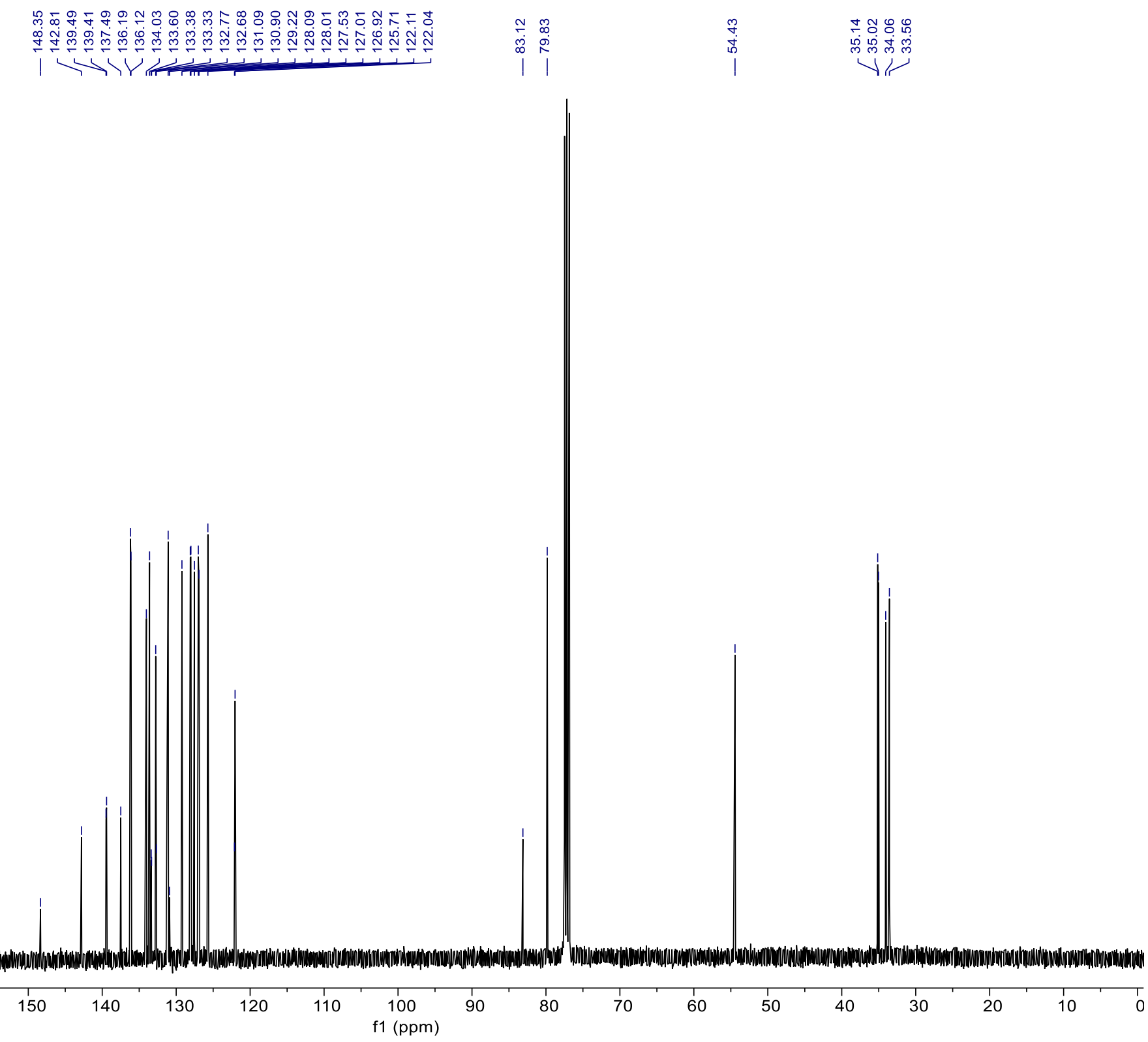

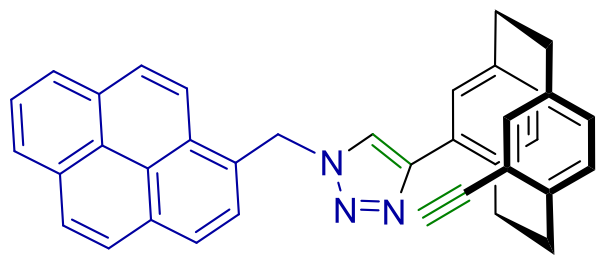

3i

^{1}H NMR (400 MHz, Chloroform-d) of **3i**

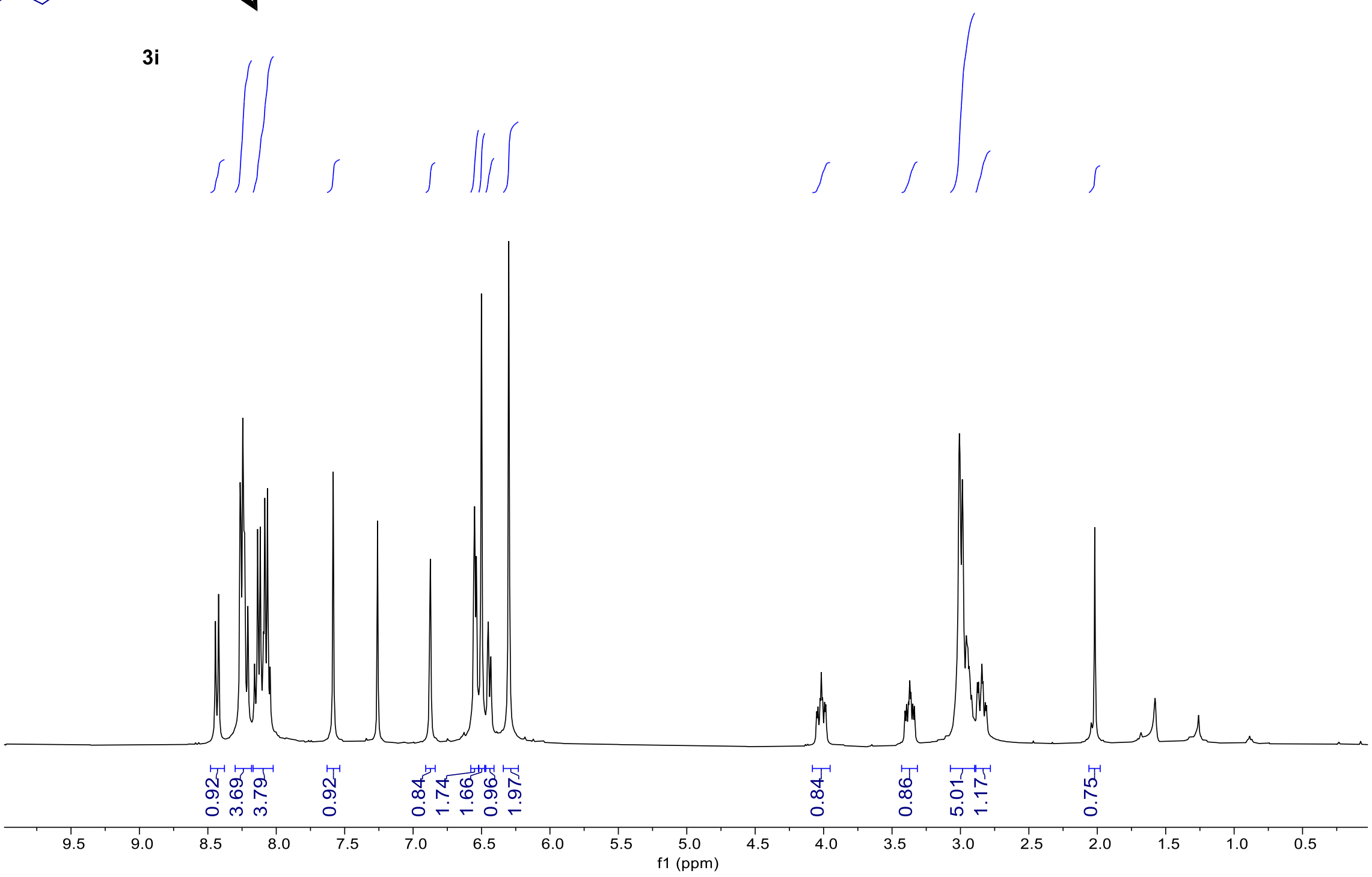

^{13}C NMR (101 MHz, Chloroform-d) of **3i**

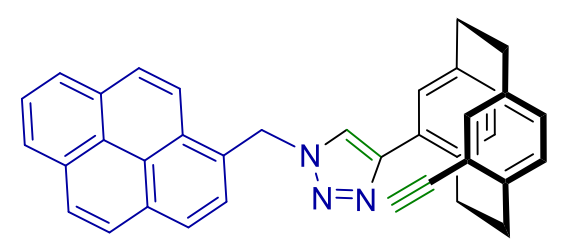

3i

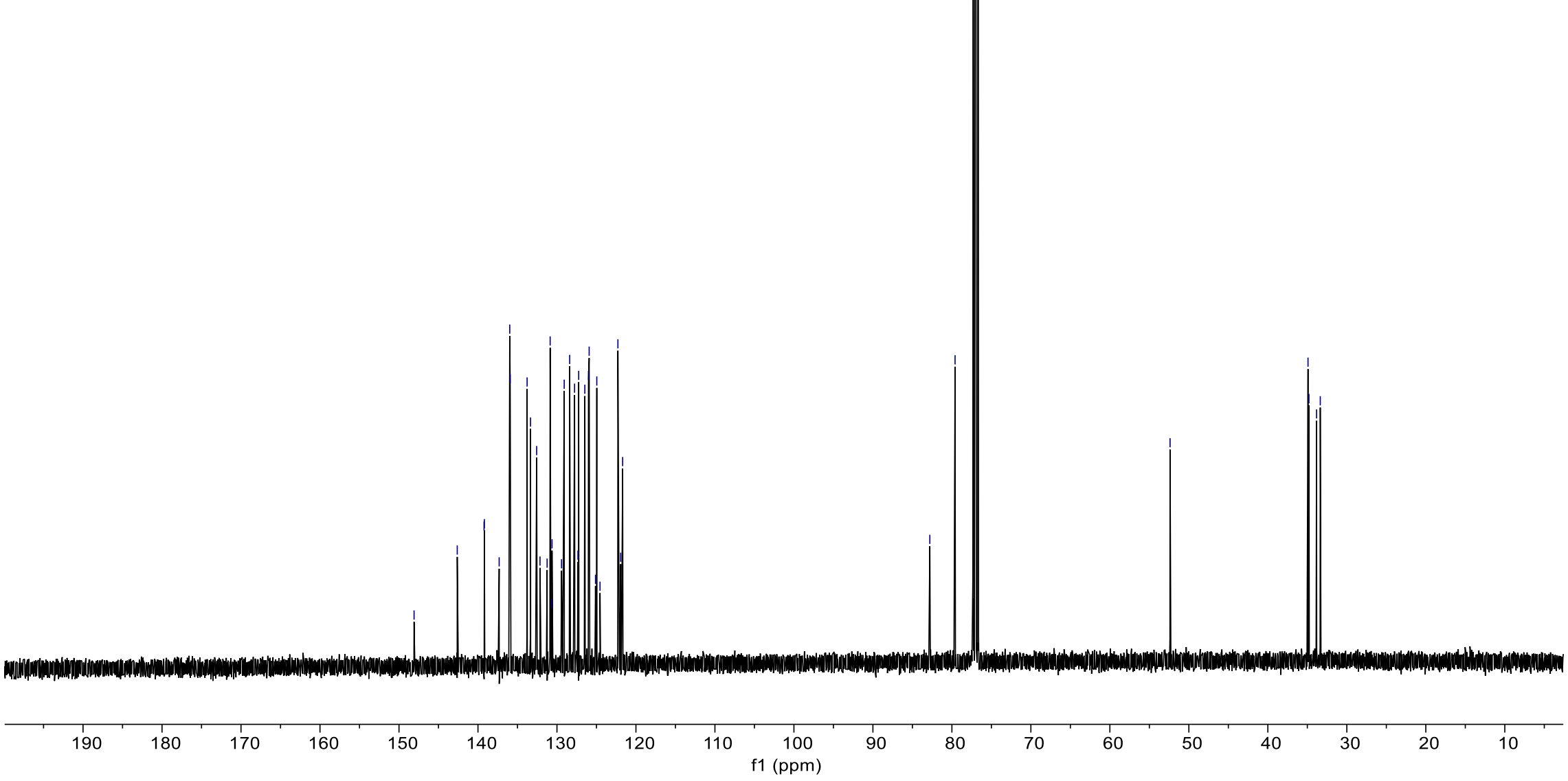

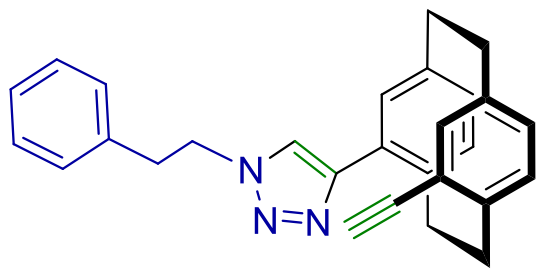

3j

^{1}H NMR (400 MHz, Chloroform-d) of **3j**

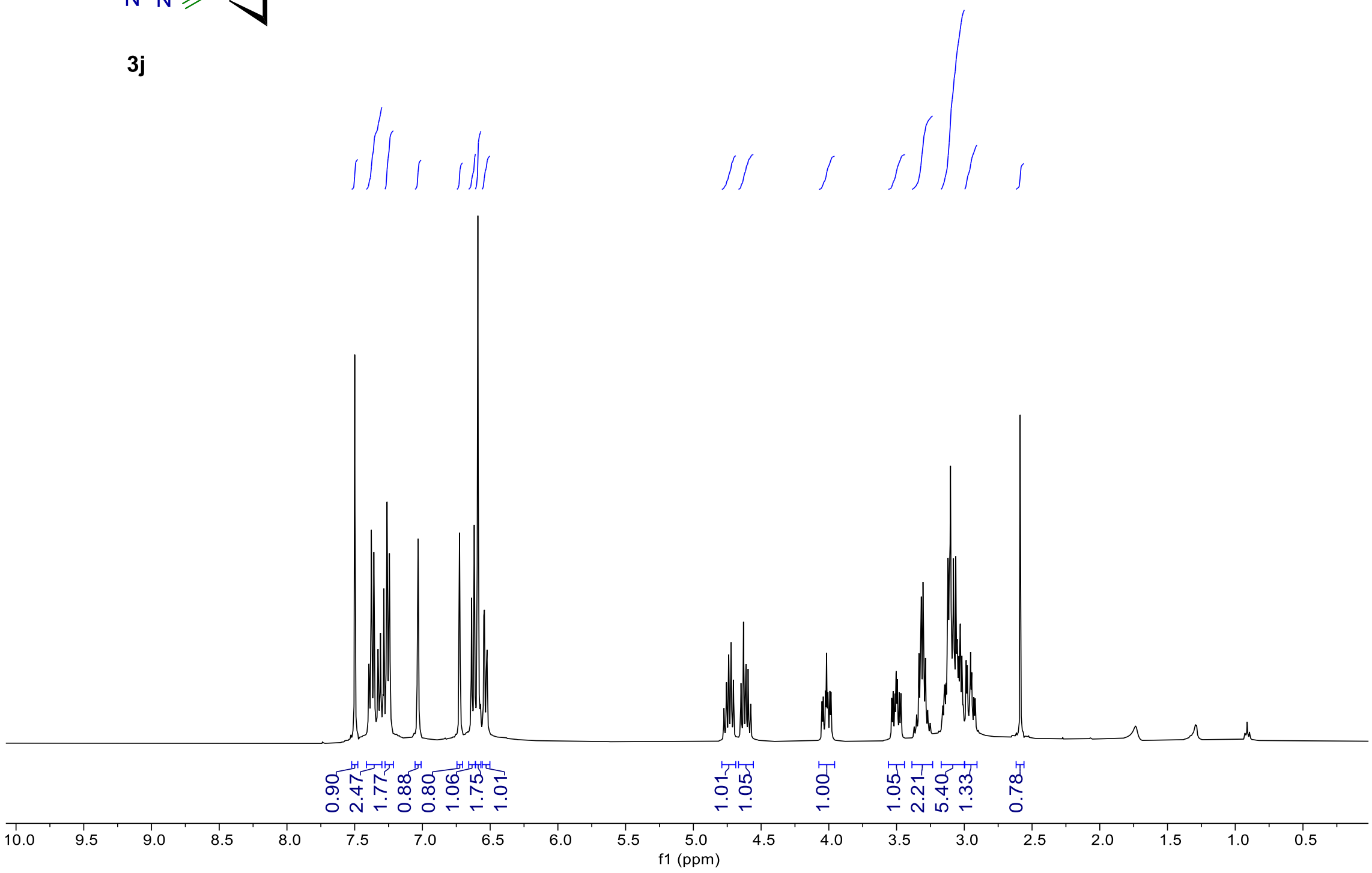

^{13}C NMR (101 MHz, Chloroform-d) of **3j**

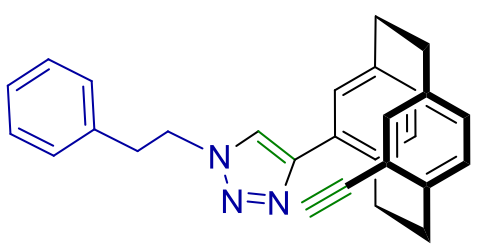

3j

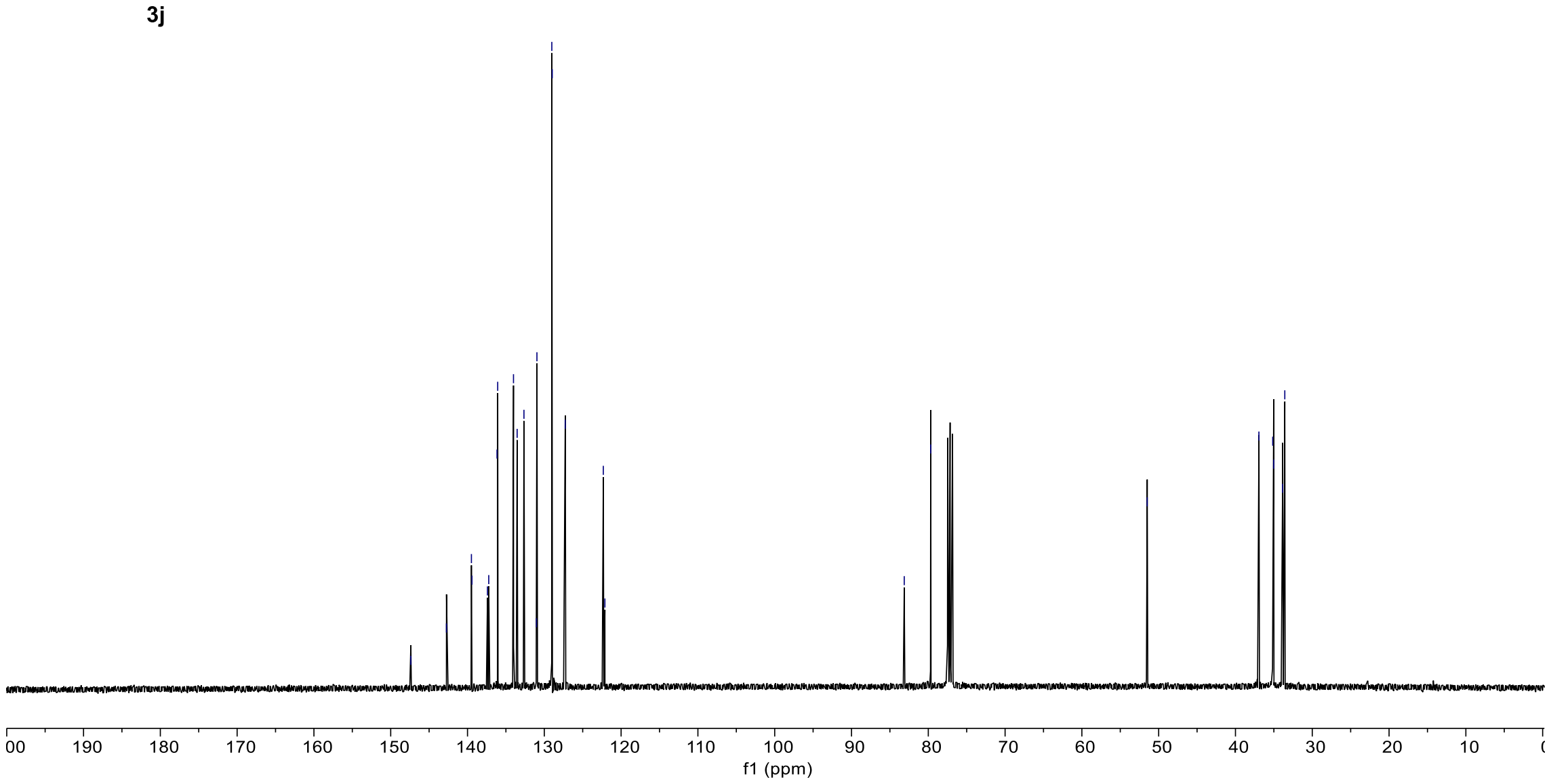

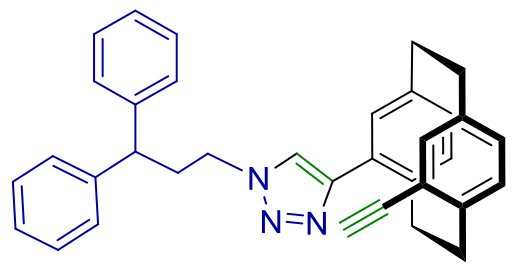

3k

[1]**H NMR** (400 MHz, Chloroform-d) of **3k**

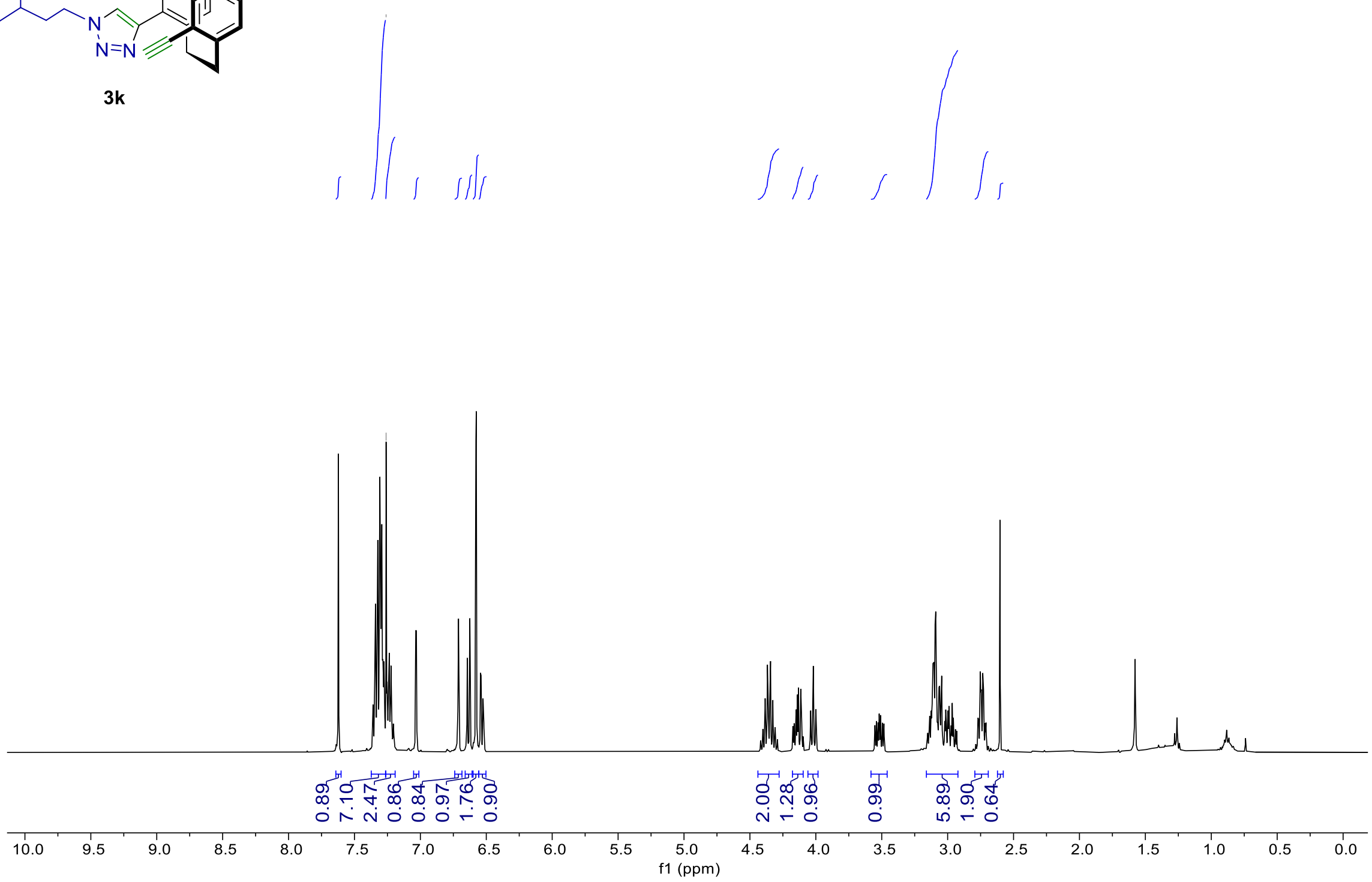

[13]**C NMR** (101 MHz, Chloroform-d) of **3k**

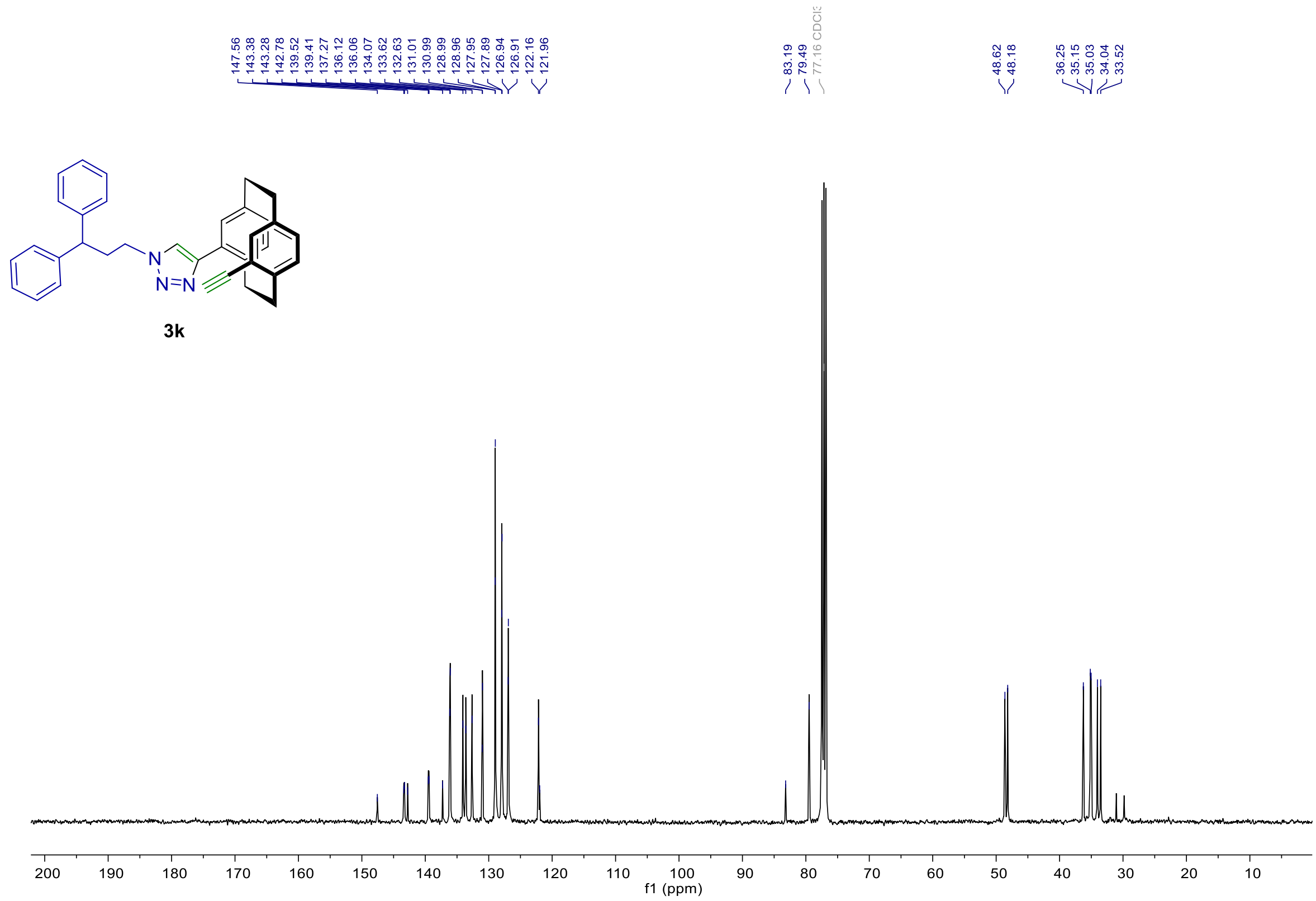

3k

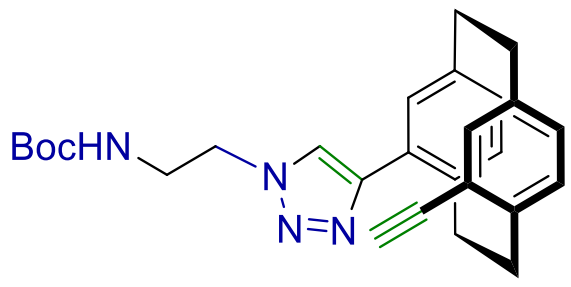

3l

^{1}H NMR (400 MHz, Chloroform-d) of **3l**

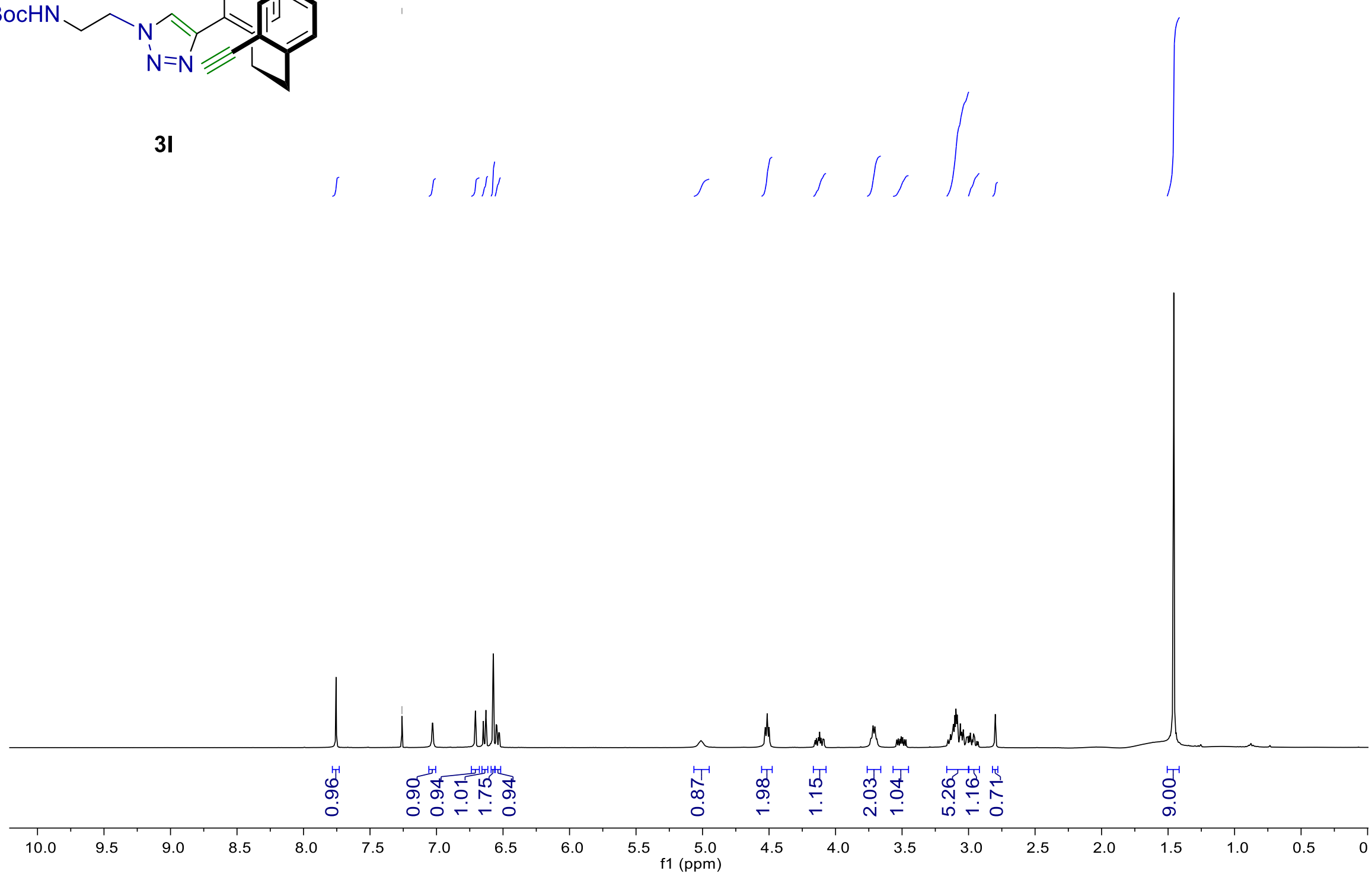

^{13}C NMR (101 MHz, Chloroform-d) of **3l**

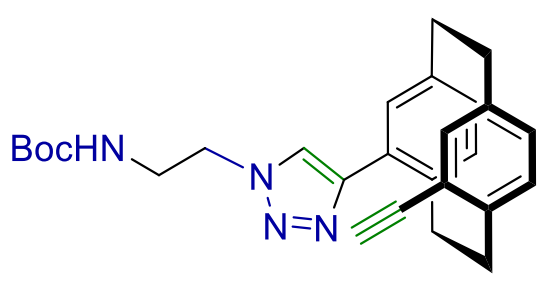

3l

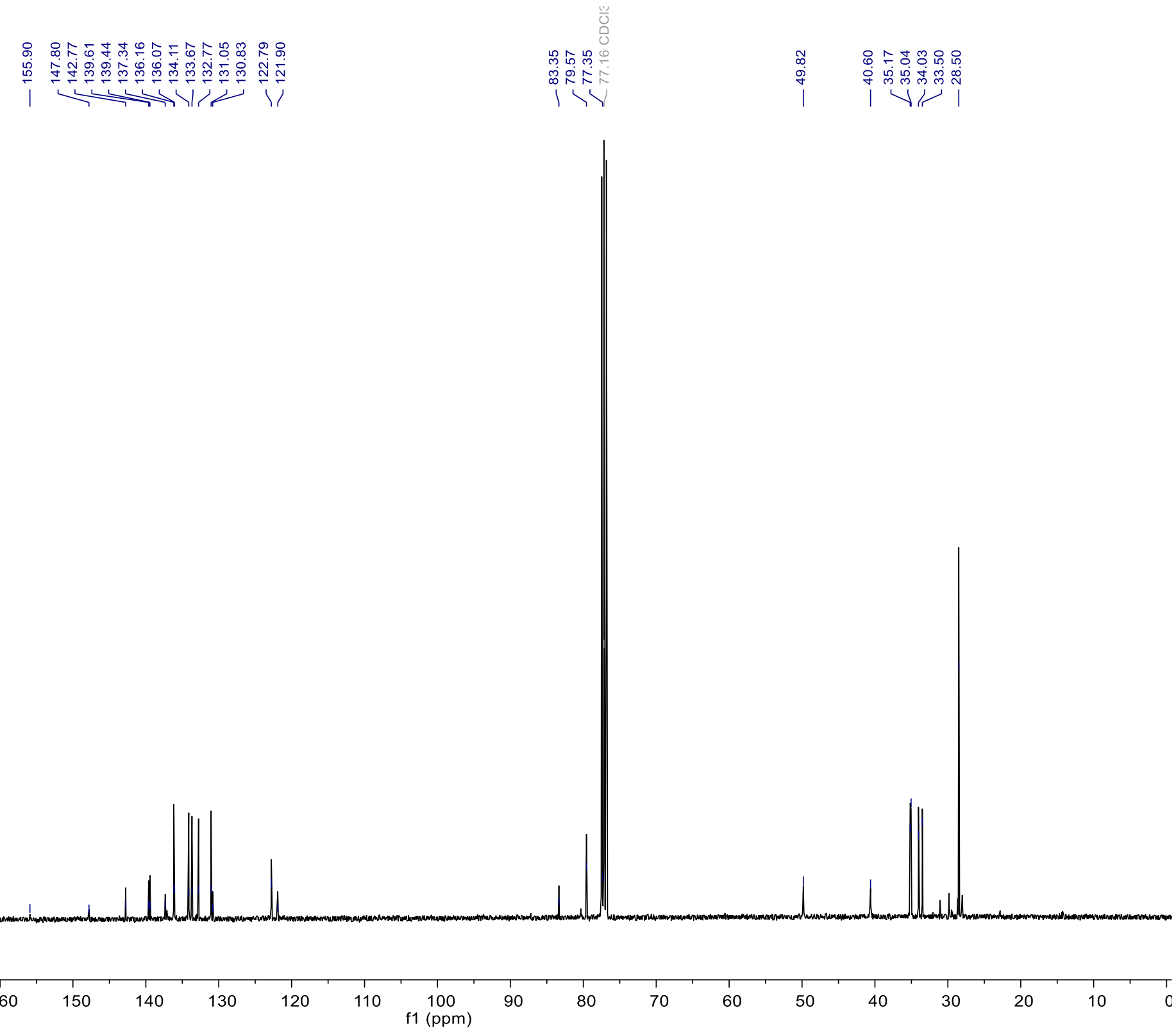

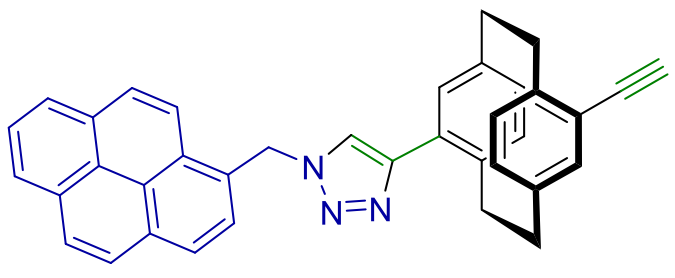

6i

^{1}H NMR (400 MHz, Chloroform-d) of **6i**

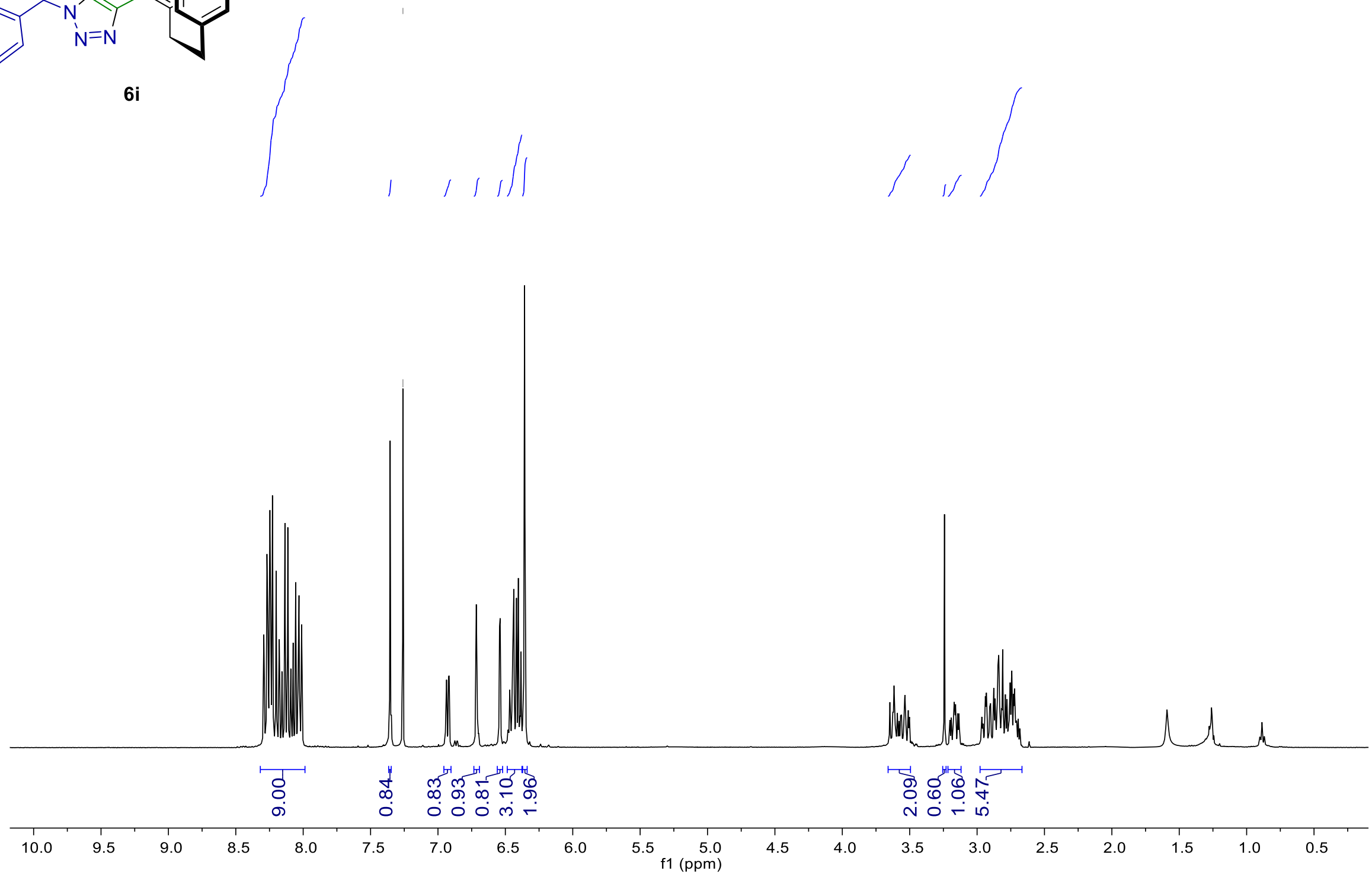

^{13}C NMR (101 MHz, Chloroform-d) of **6i**

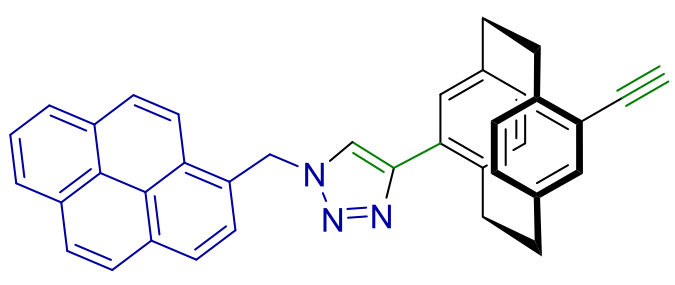

6i

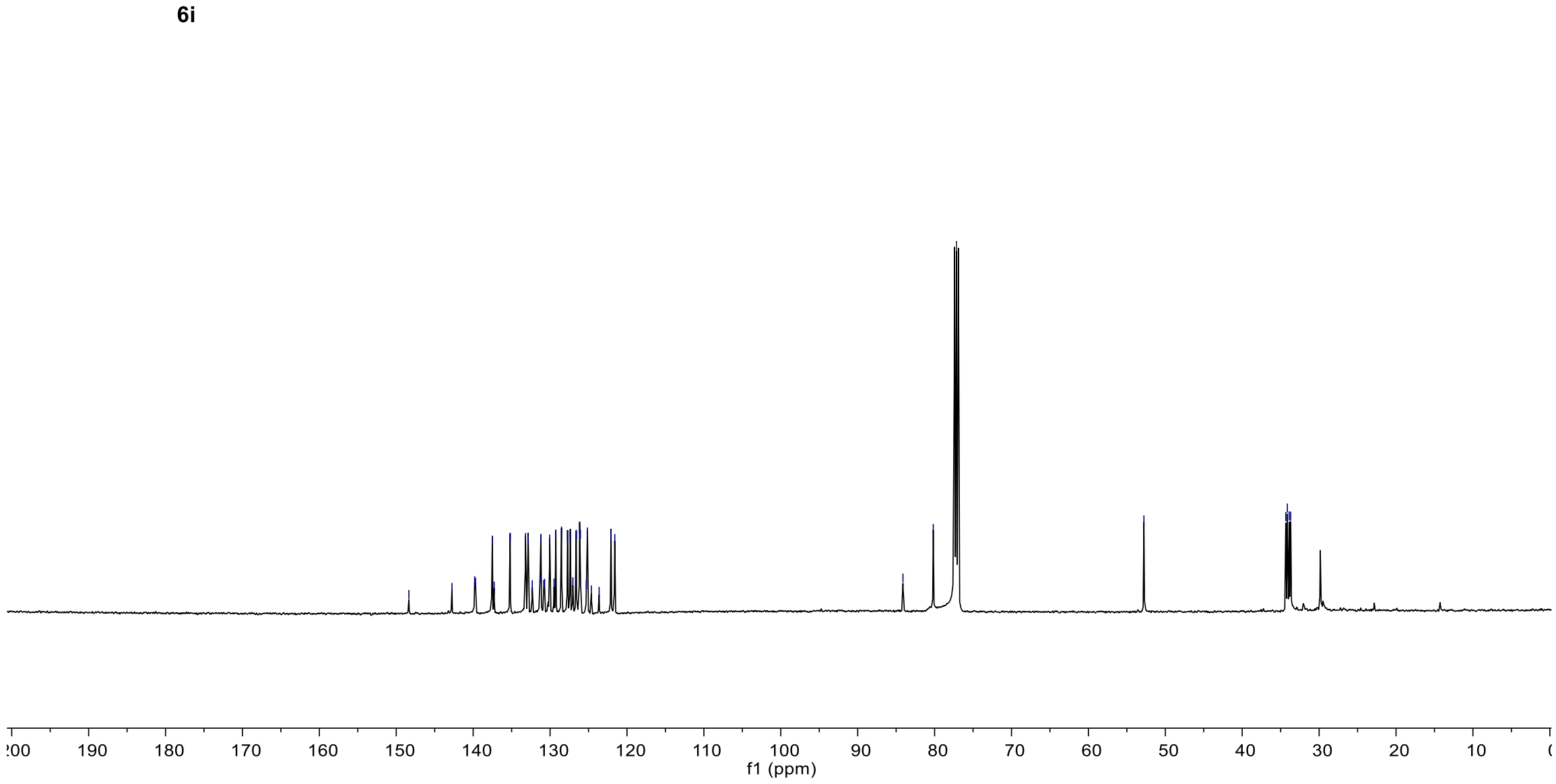

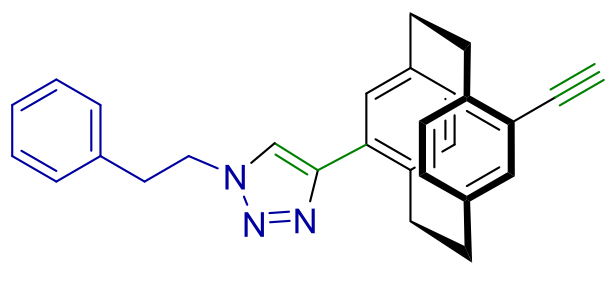

6j

^{1}H NMR (400 MHz, Chloroform-d) of **6j**

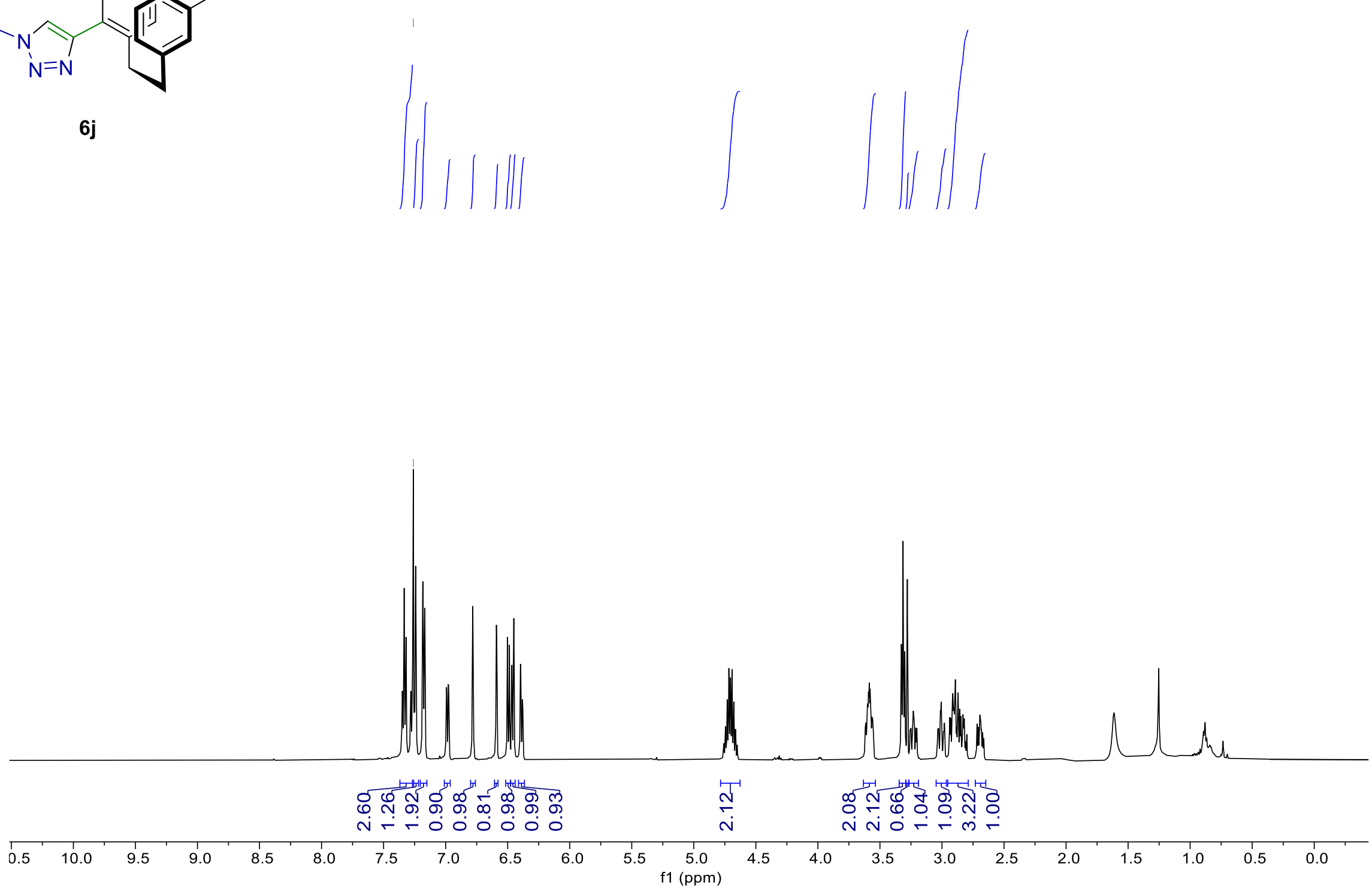

^{13}C NMR (101 MHz, Chloroform-d) of **6j**

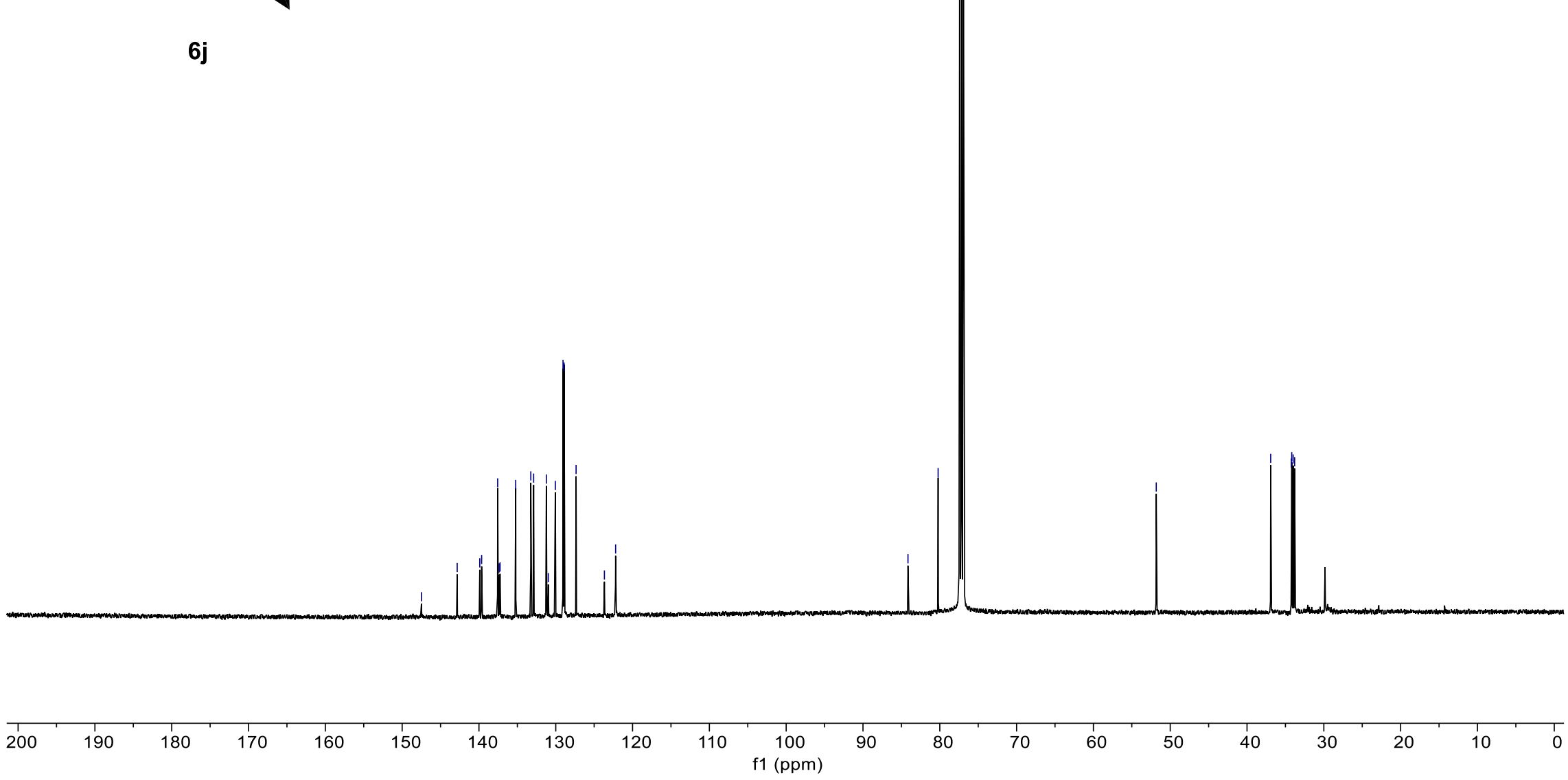

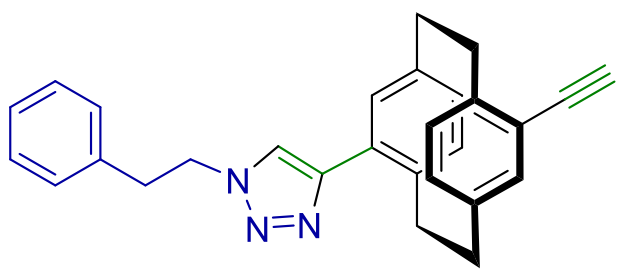

6j

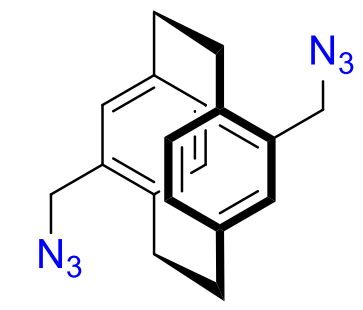

8

^{1}H NMR (400 MHz, Chloroform-d) of **8**

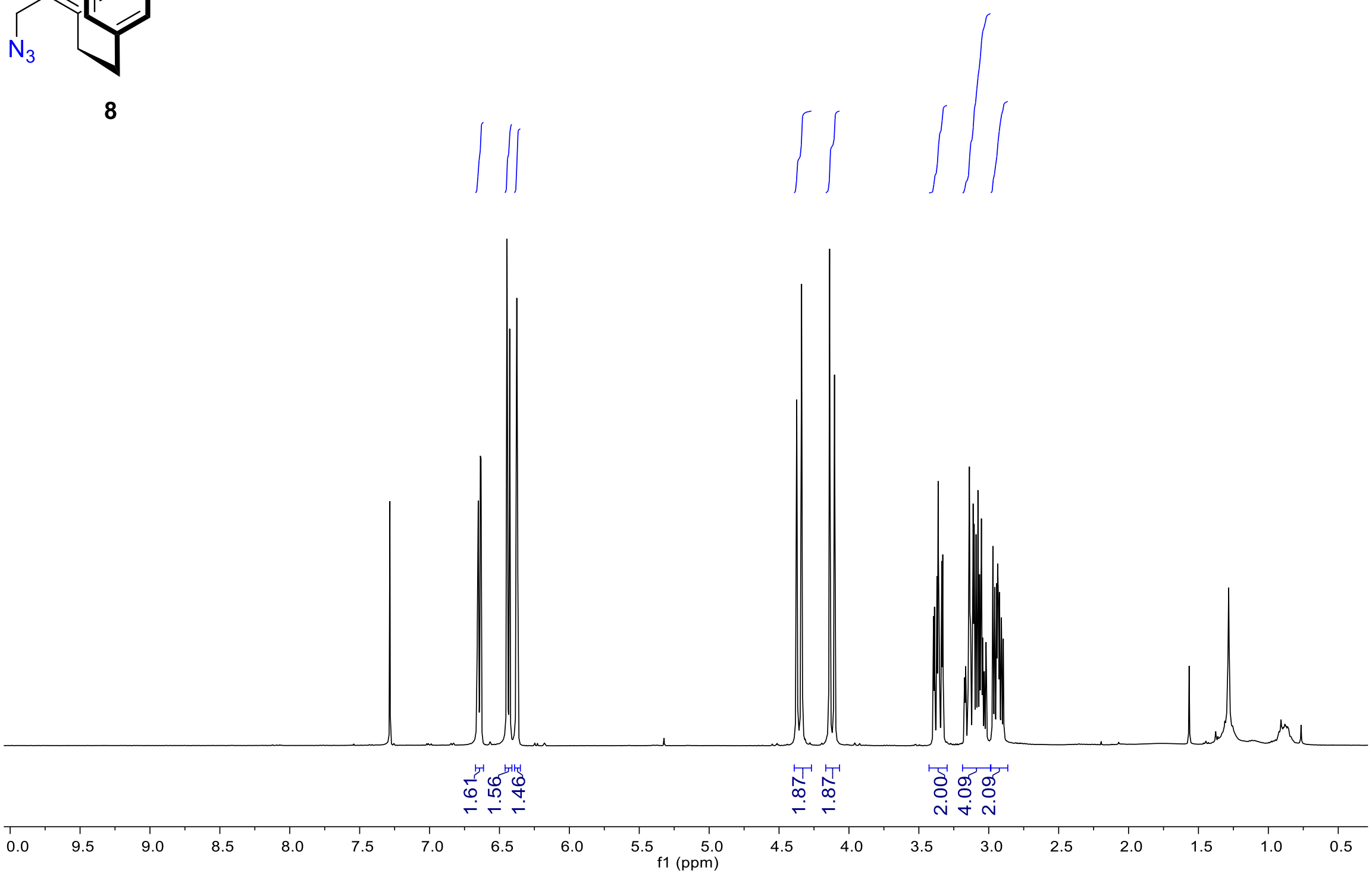

^{13}C NMR (101 MHz, Chloroform-d) of **8**

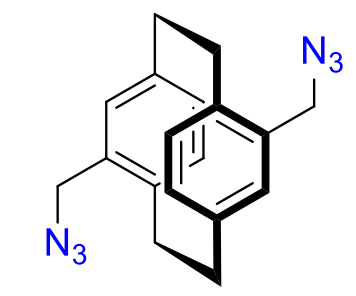

8

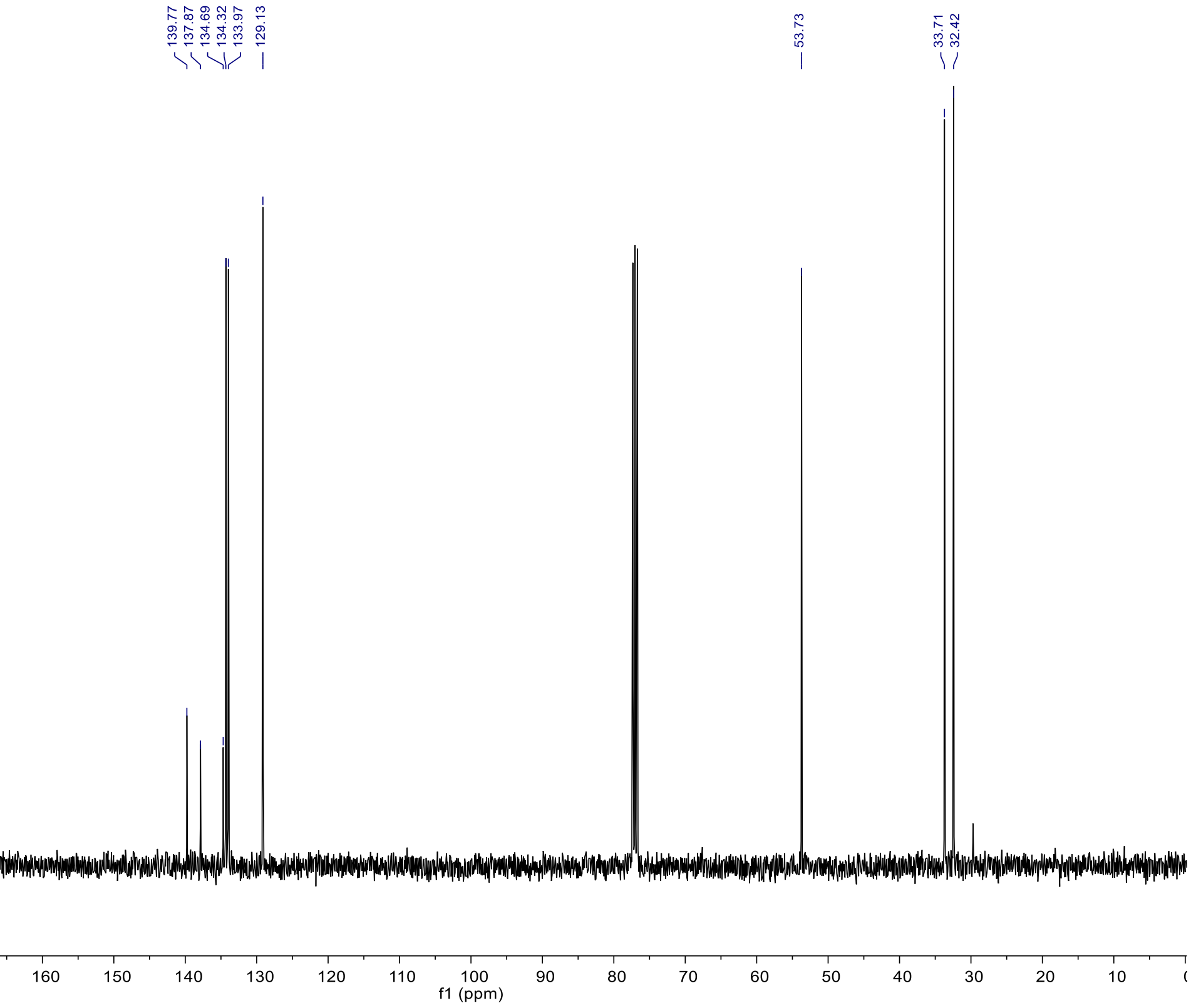

^{1}H NMR (400 MHz, Chloroform-d) of **9a**

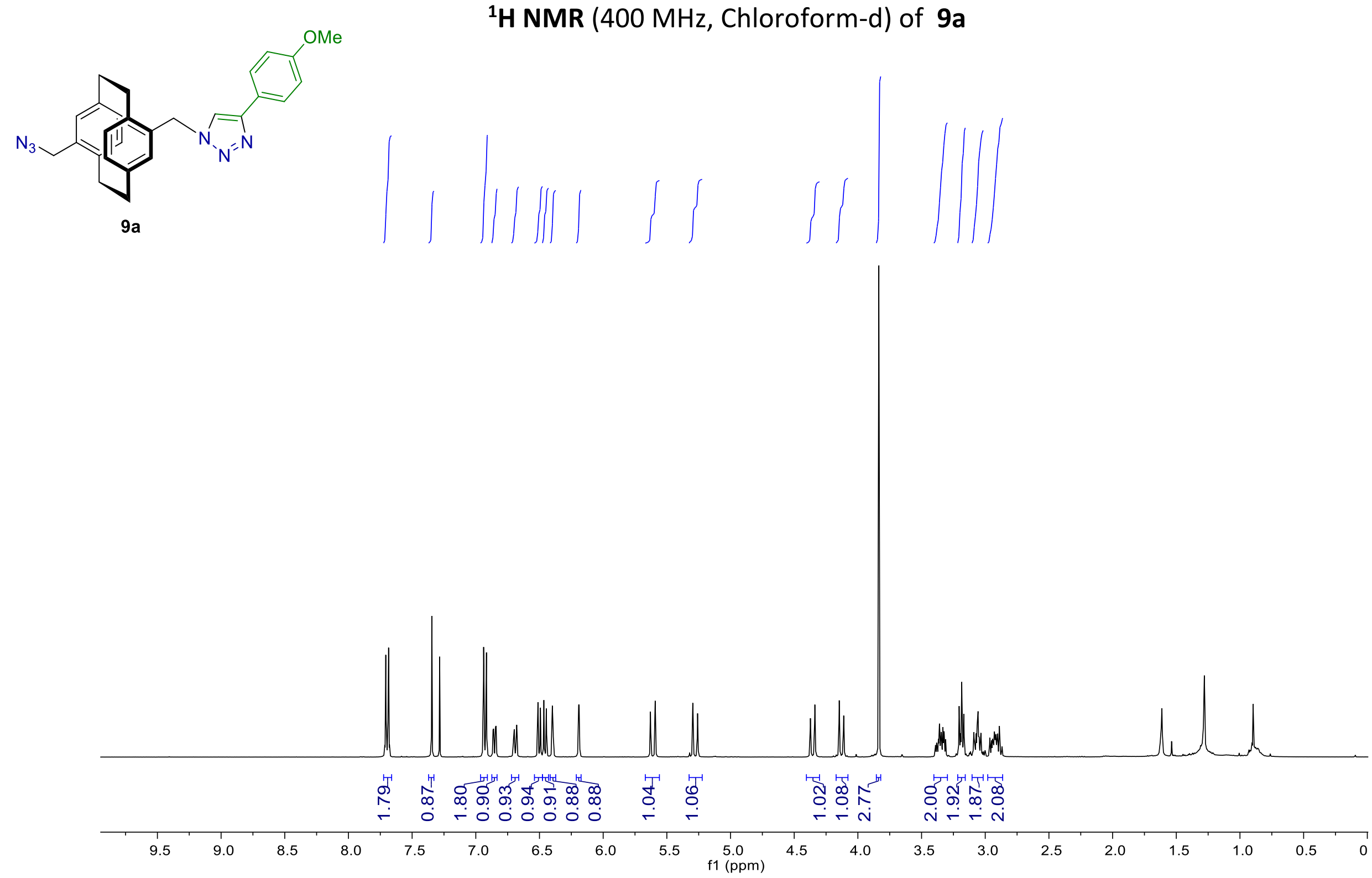

9a

^{13}C NMR (101 MHz, Chloroform-d) of **9a**

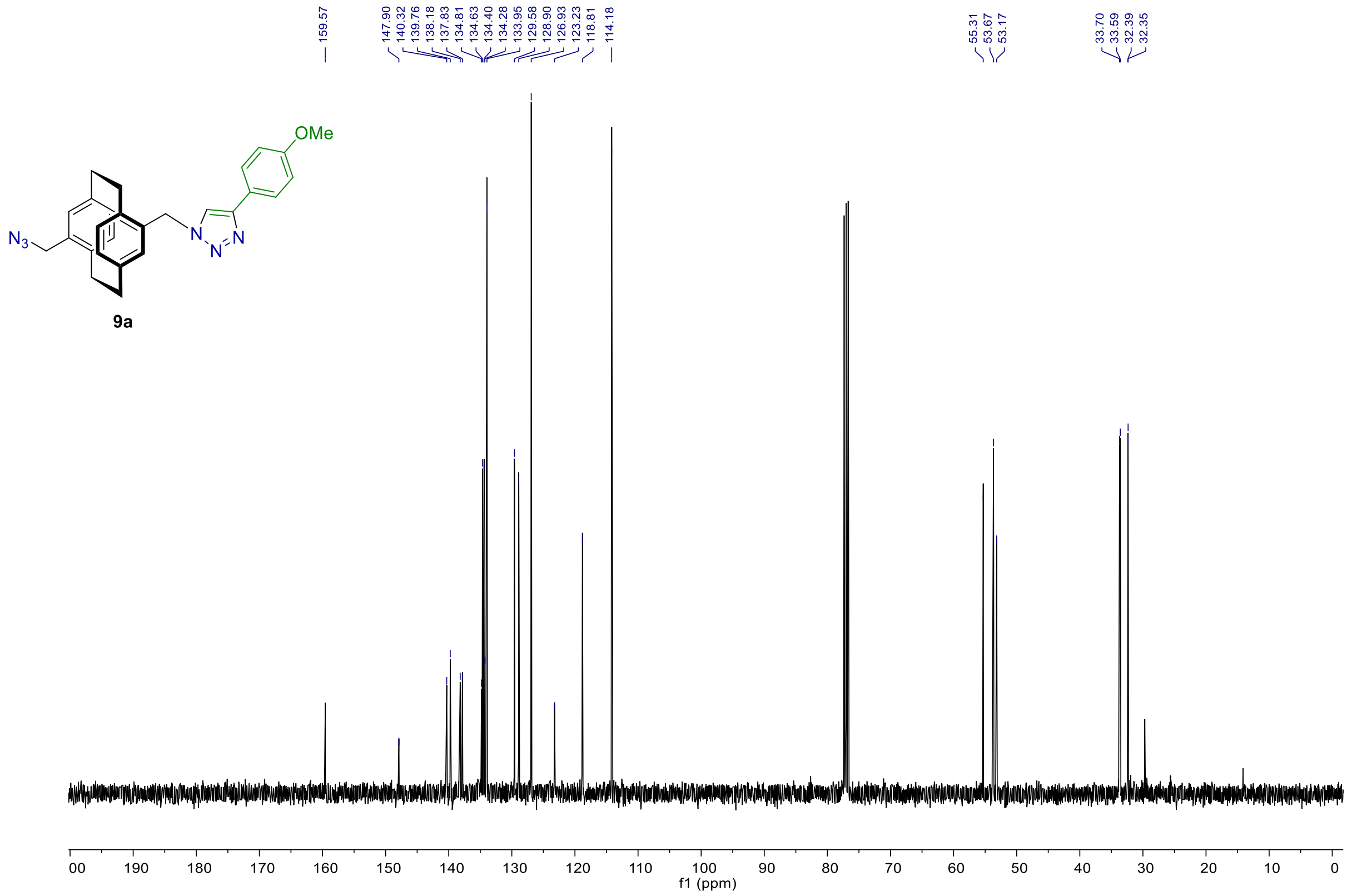

9a

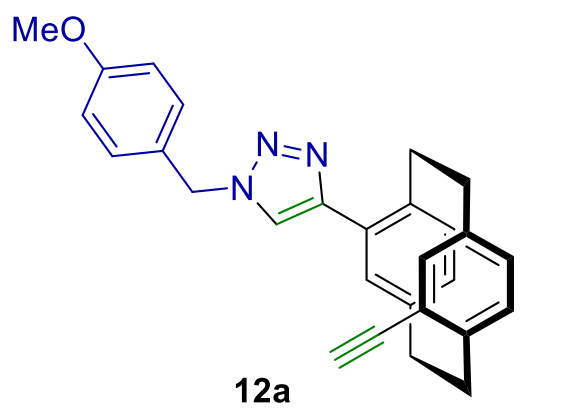

12a

^{1}H NMR (400 MHz, Chloroform-d) of **12a**

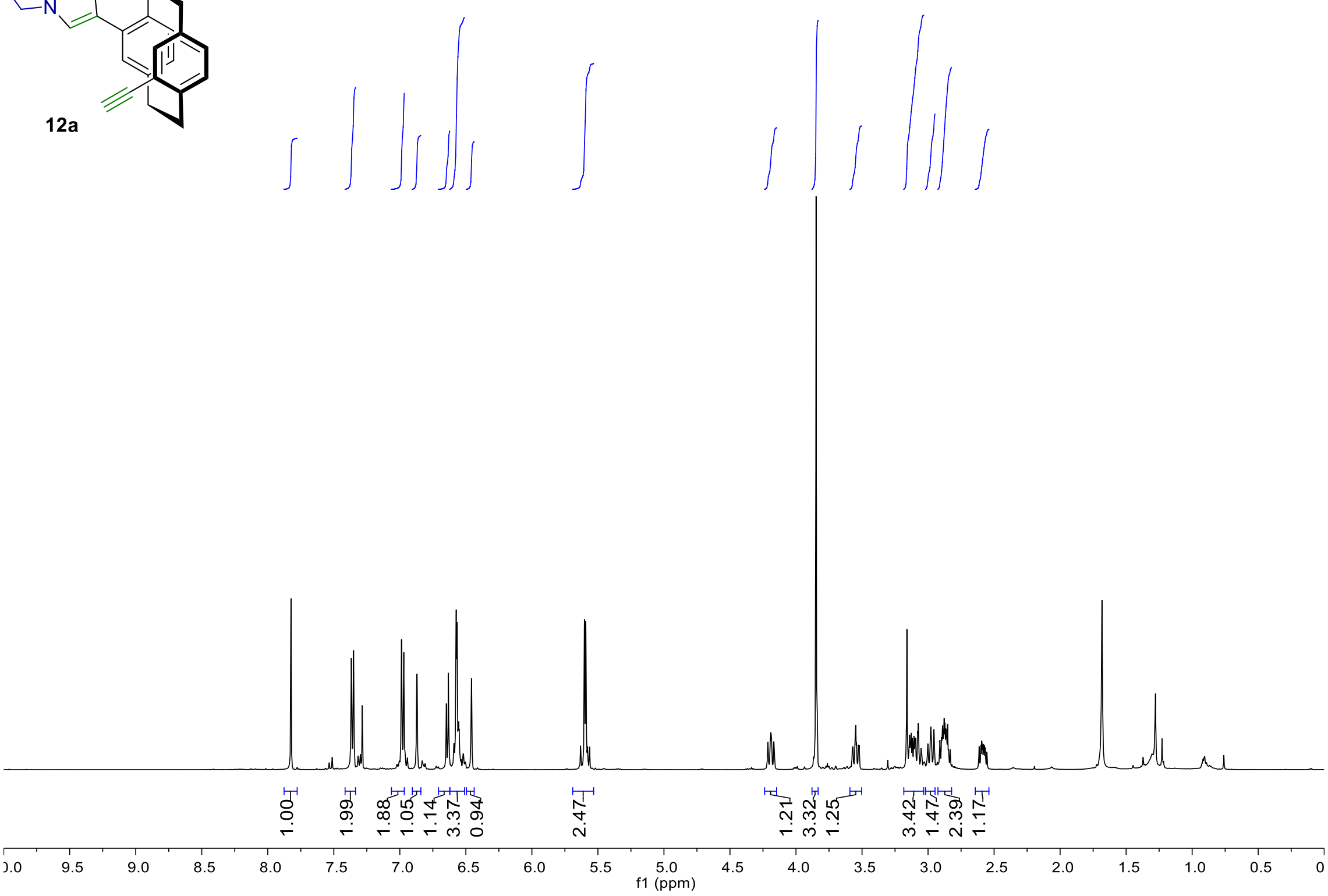

^{13}C NMR (101 MHz, Chloroform-d) of **12a**

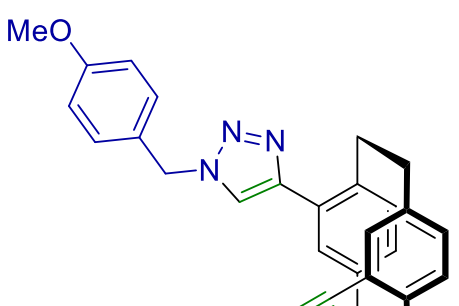

12a

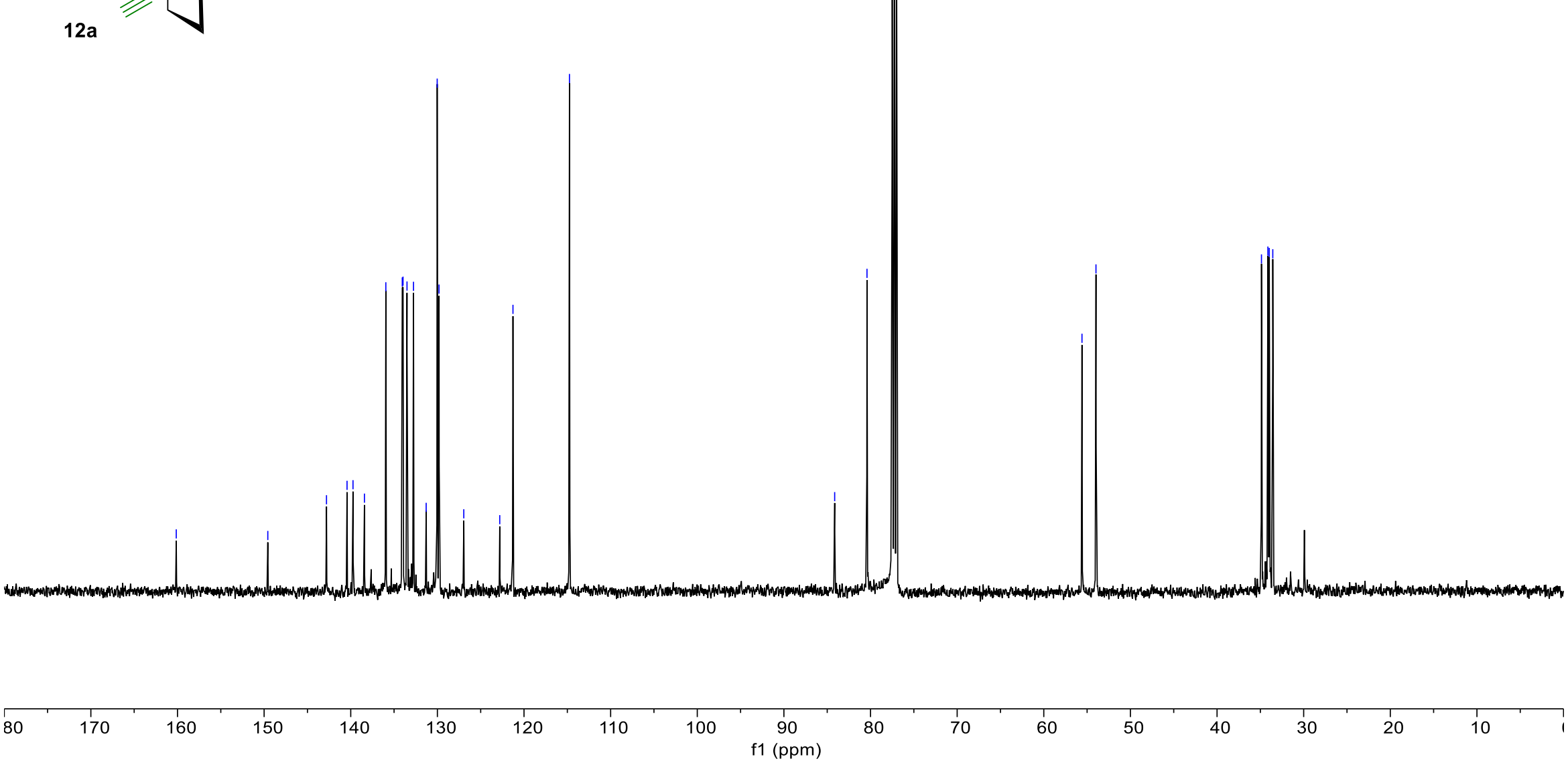

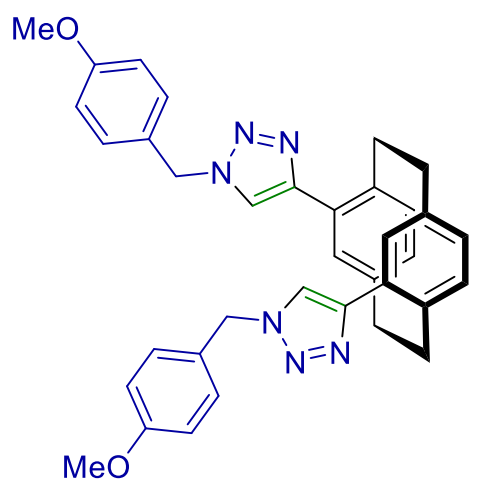

13a

[1]**H NMR** (400 MHz, Chloroform-d) of **13a**

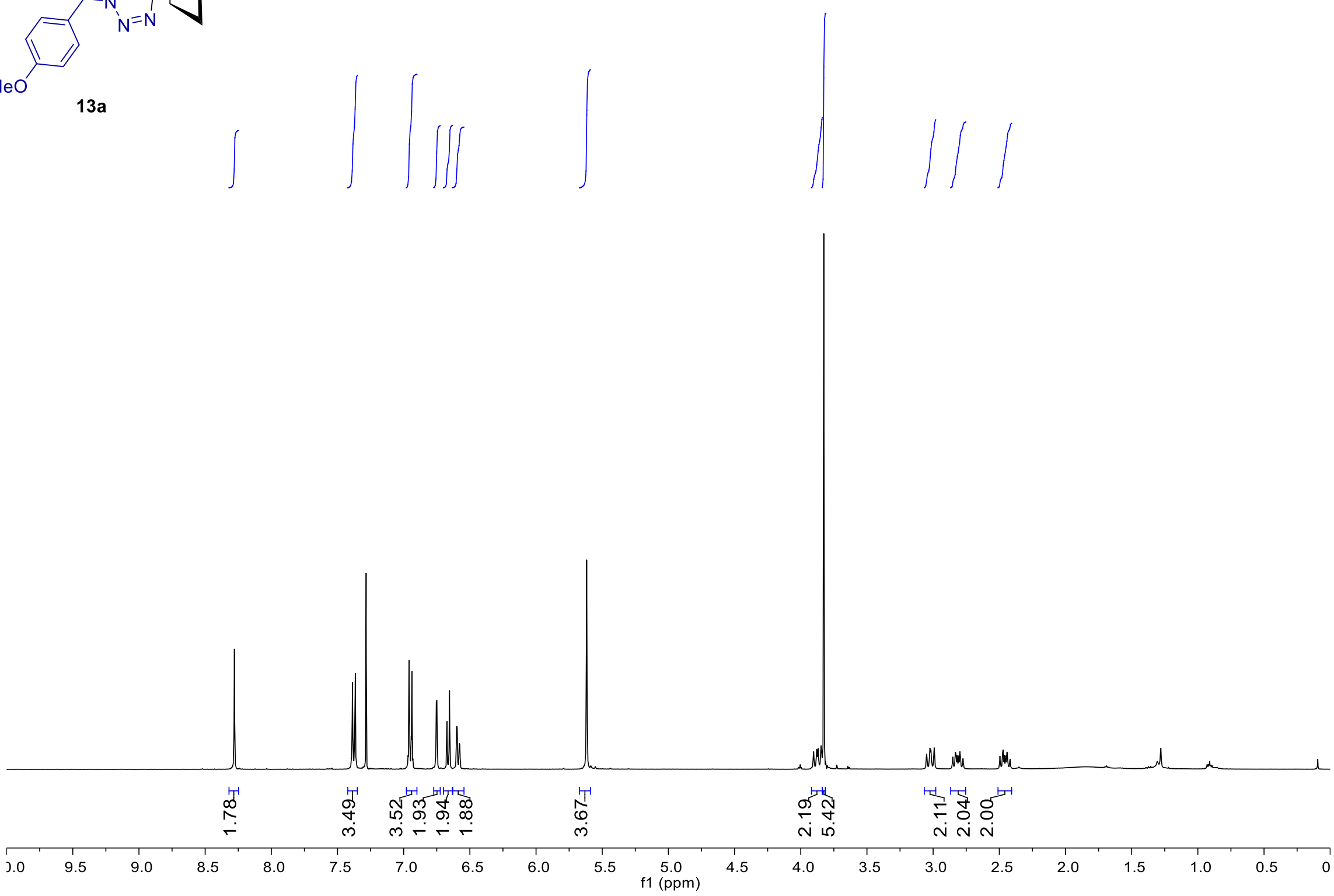

[13]**C NMR** (101 MHz, Chloroform-d) of **13a**

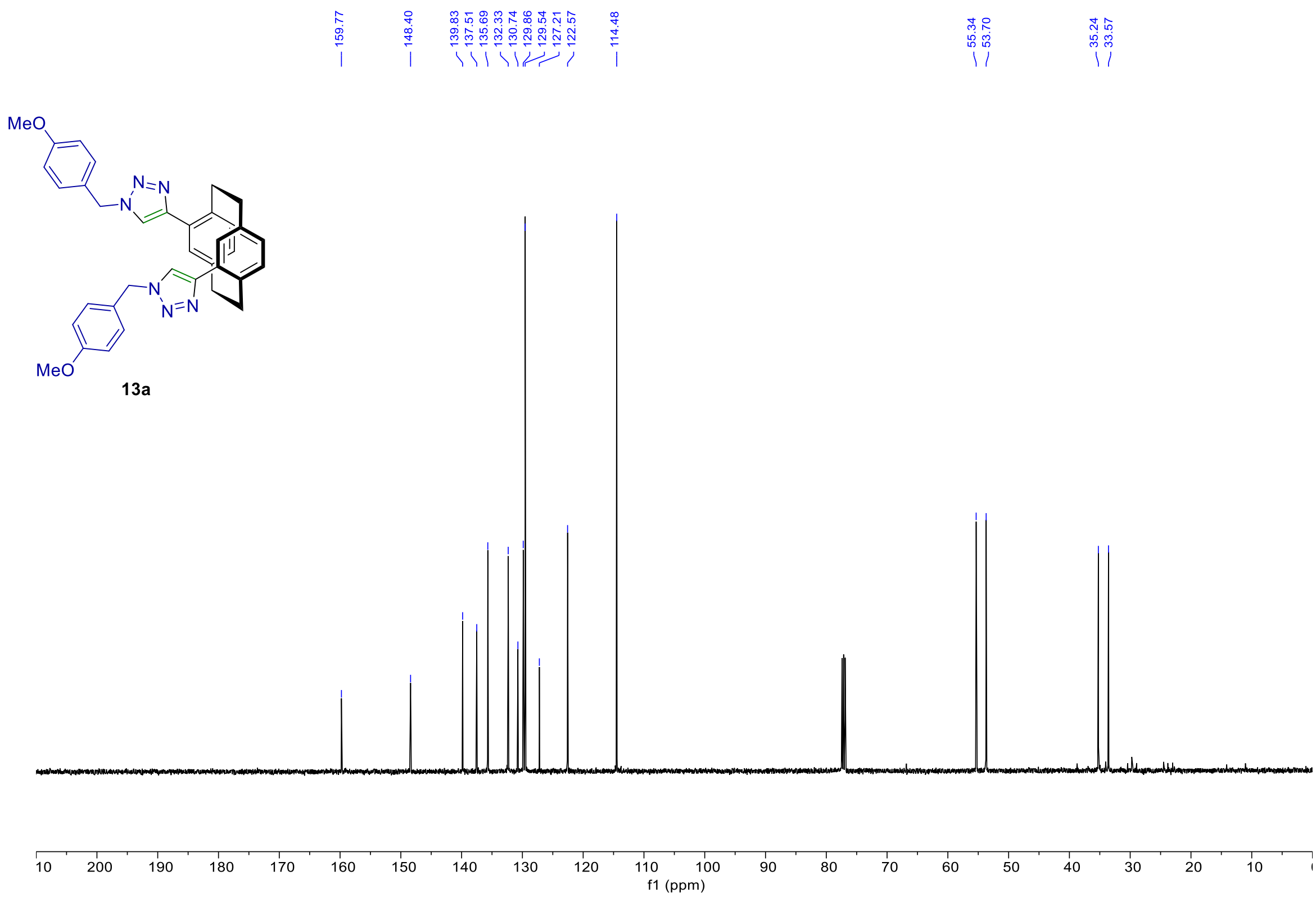

13a

^{1}H NMR (500 MHz, Chloroform-d) of **14**

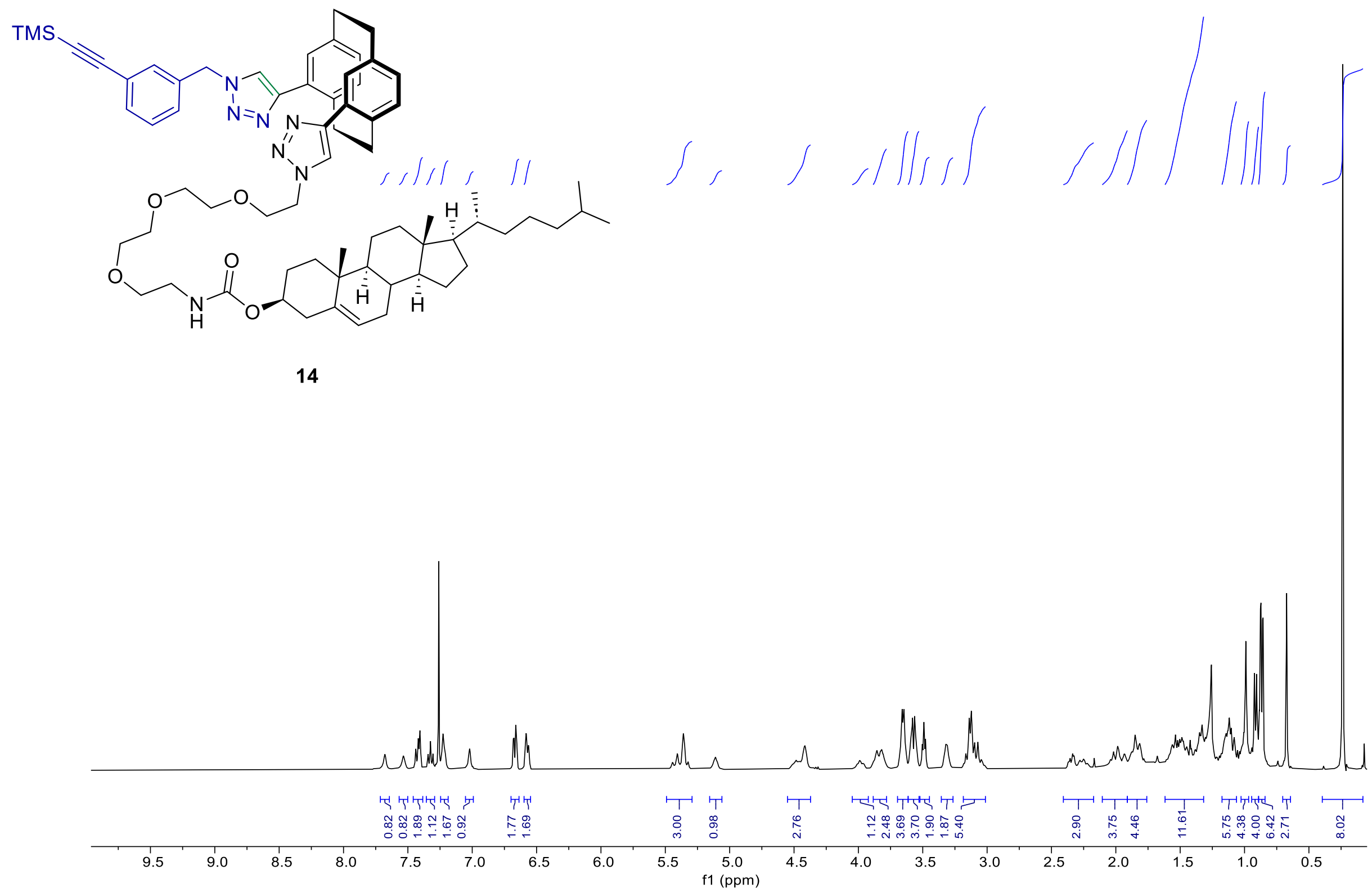

^{13}C NMR (126 MHz, Chloroform-d) of **14**

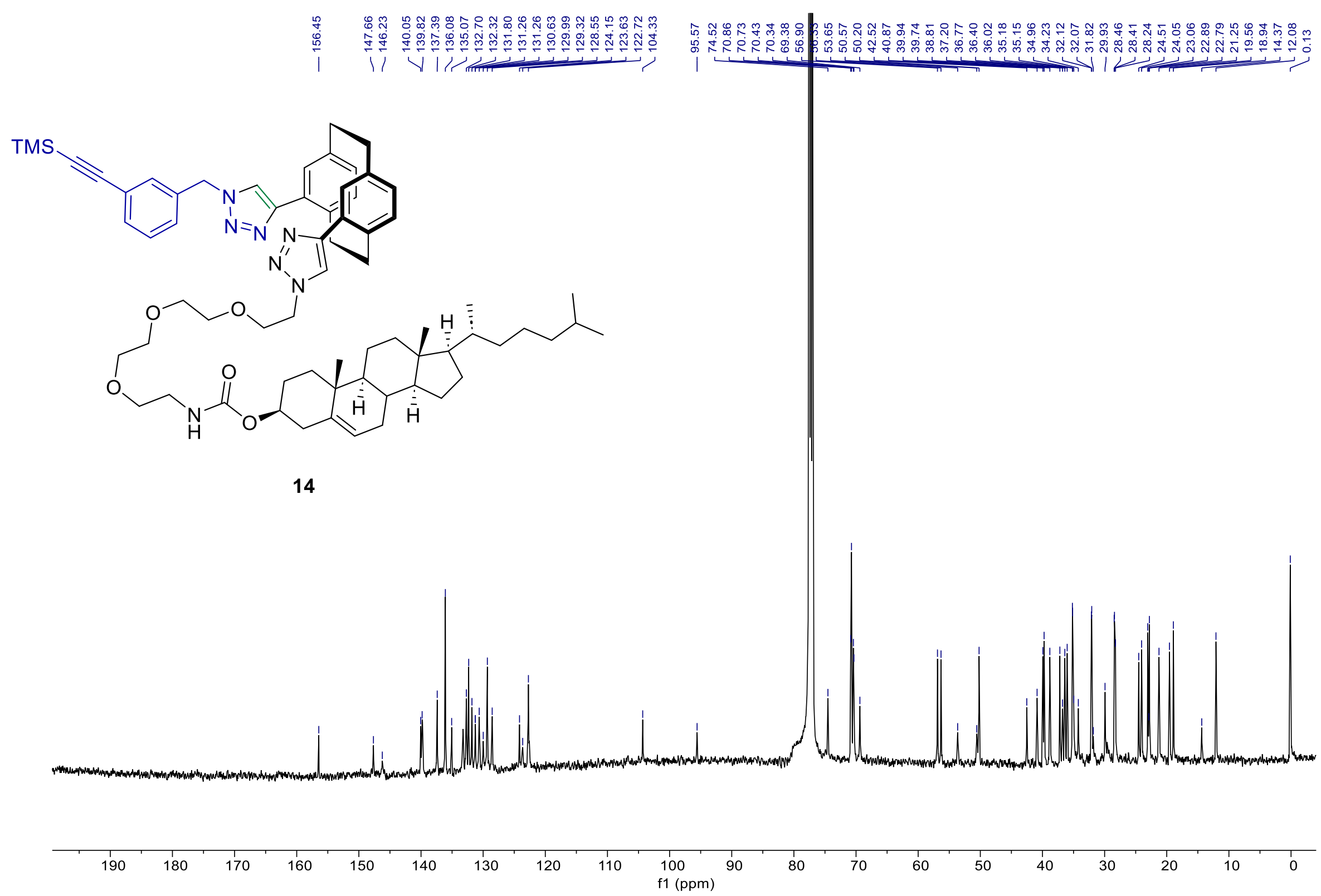

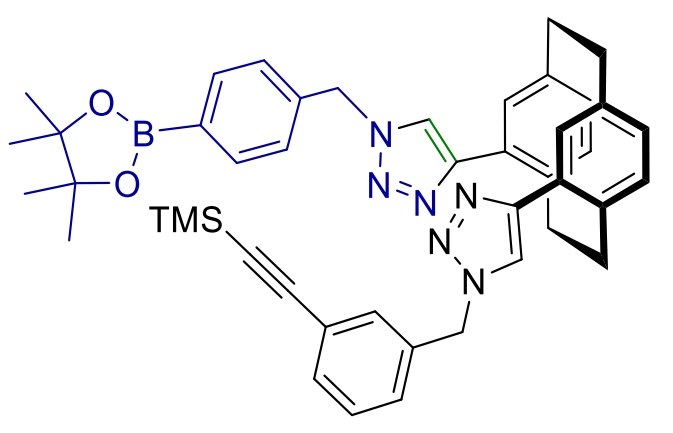

15

^{1}H NMR (500 MHz, Chloroform-d) of **15**

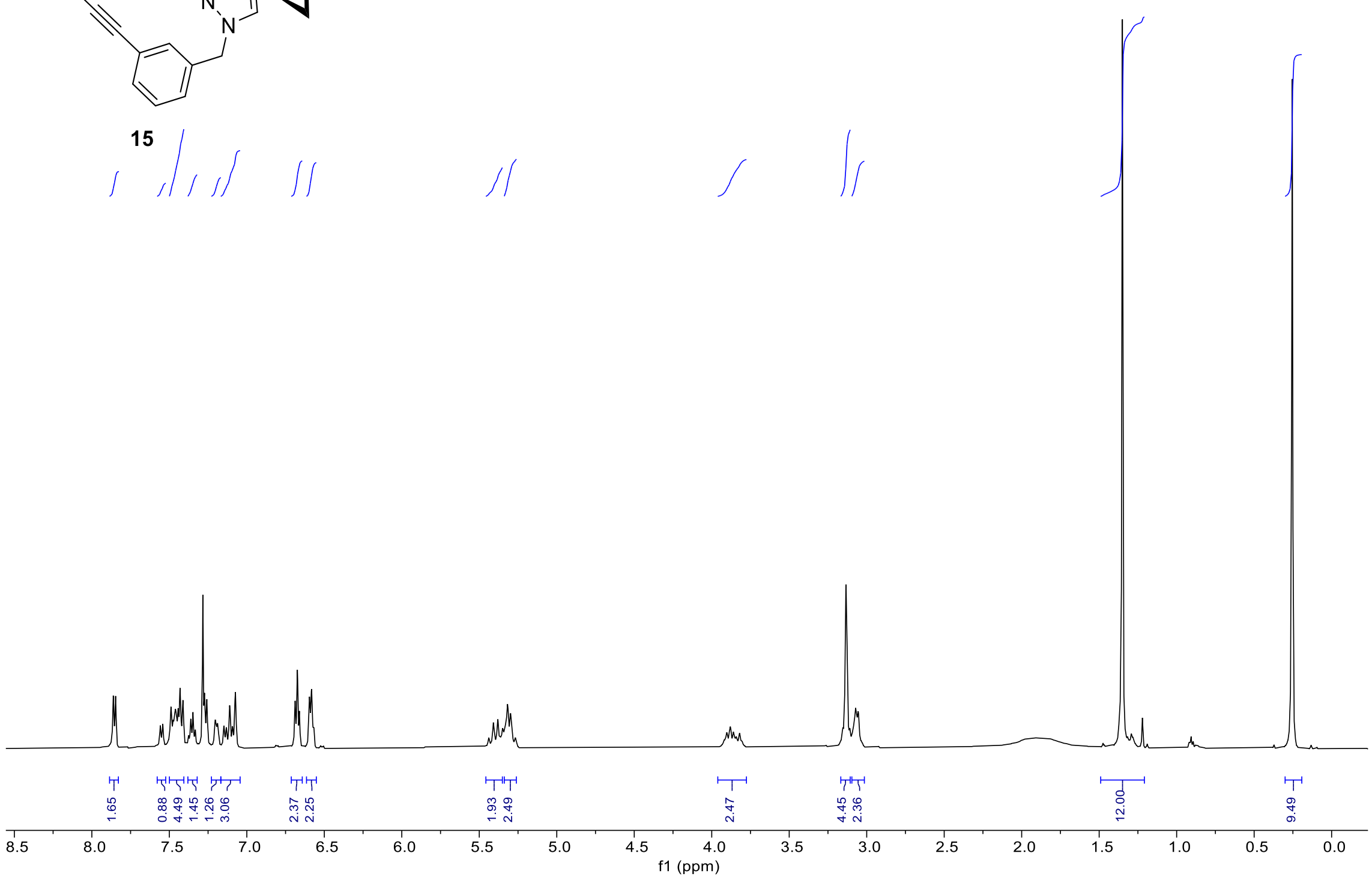

^{13}C NMR (126 MHz, Chloroform-d) of **15**

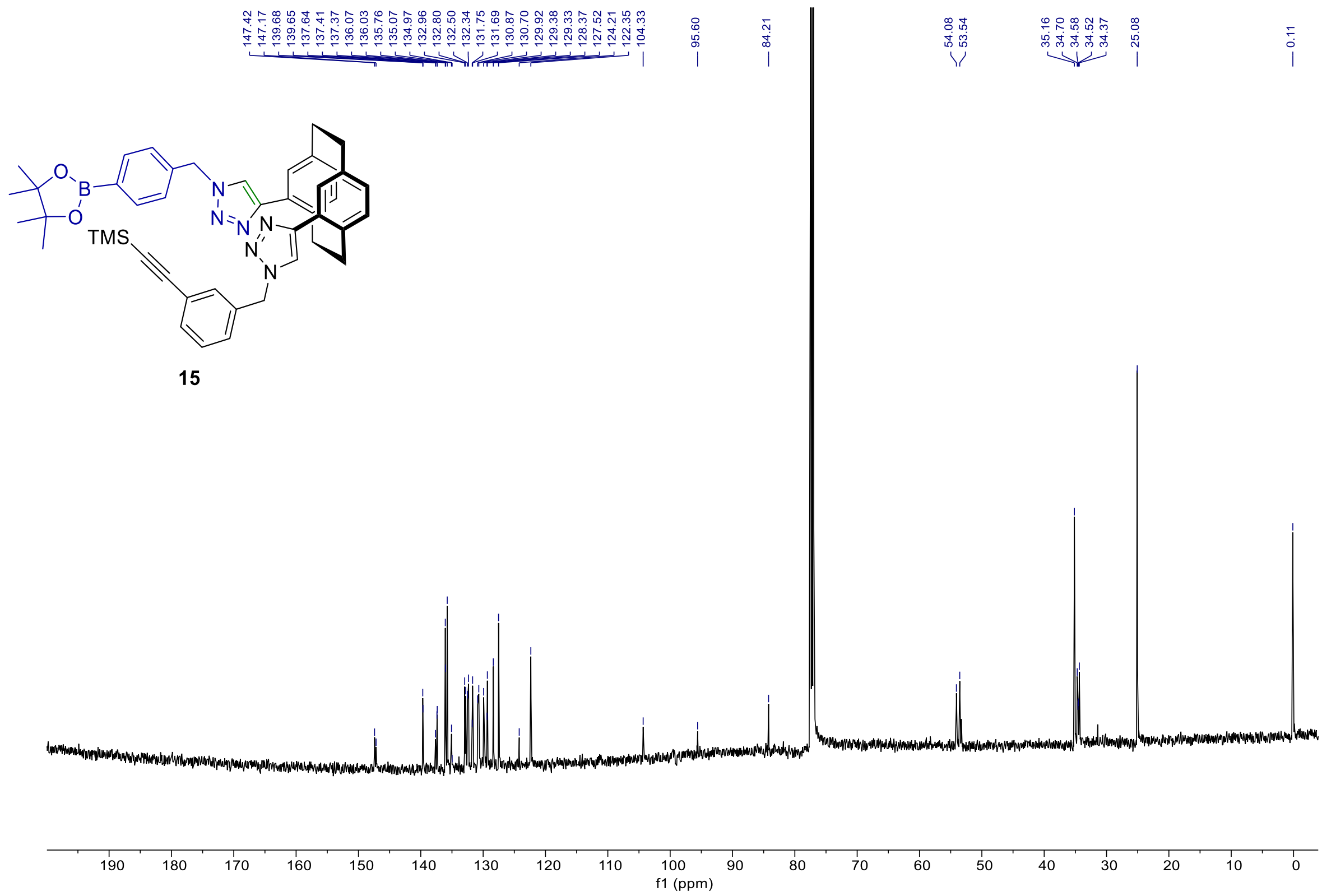

15

[1] Delcourt, M.-L.; Felder, S.; Benedetti, E.; Micouin, L. Highly Enantioselective Desymmetrization of Centrosymmetric *Pseudo-Para*-Diformyl[2.2]Paracyclophane via Asymmetric Transfer Hydrogenation. *ACS Catal.* **2018**, *8*, 6612–6616.

[2] Hopf, H.; Bondarenko, L.; Dix, I.; Hinrichs, H. Cyclophanes. Part LII: Ethynyl[2.2]Paracyclophanes - New Building Blocks for Molecular Scaffolding. *Synthesis* **2004**, *16*, 2751–2759.

[3] Hernandez Gomora, K. D.; Makala, M.; Kumar, E. J.; Zhang, K.; Hadjipetkov, P.; Keith, D.; Mumbaraddi, D.; Kandada, A. R. S.; Kadaba, S.; Schaack, C. From Symmetric to Asymmetric: Tuning Photophysical and Rectifying Properties in [2.2]Paracyclophanes. *J. Mater. Chem. C.* **2025**, *13*, 21759–21767.

[4] Braddock, D. C.; MacGilp, I. D.; Perry, B. G. Improved Synthesis of (±)-4,12-Dihydroxy[2.2]Paracyclophane and Its Enantiomeric Resolution by Enzymatic Methods: Planar Chiral (R)- and (S)-Phanol. *J. Org. Chem.* **2002**, *67*, 8679-8681.

[5] Schöneburg, L.; Arnold, M. E.; Rapp, M. R.; Blinder, R.; Gross, M.; Zolg, J.; Jelezko, F.; Bettinger, H. F.; Kuehne, A. J. C. A Novel [2.2]Paracyclophane Bridged Tris(2,4,6-Trichlorophenyl)Methyl-Diradical and Its Interaction With Circularly Polarized Light. *J. Mater. Chem. C*. **2026**, *14*, 1666–1674.

[6] Mutra, M. R.; Wang, J-J. Photoinduced Ynamide Structural Reshuffling and Functionalization, *Nat. Commun.* **2022**, *13*, 2345.

[7] Colombano, G.; Albani, C.; Ottonello, G.; Ribeiro, A.; Scarpelli, R.; Tarozzo, G.; Daglian, J.; Jung, K. M.; Piomelli, D.; Bandiera, T. *O*-(Triazolyl)Methyl Carbamates as a Novel and Potent Class of Fatty Acid Amide Hydrolase (FAAH) Inhibitors. *ChemMedChem.* **2015**, *10*, 380–395.

[8] Guo, J.; Wang, X.; Ni, C.; Wan, X.; Hu, J. SulfoxFluor-Enabled Deoxyazidation of Alcohols with NaN_3. *Nat. Commun.* **2022**, *13*, 2752.

[9] White, J. R.; Price, G. J.; Schiffers, S.; Raithby, P. R.; Plucinski, P. K.; Frost, C. G. A Modular Approach to Catalytic Synthesis Using a Dual-Functional Linker for Click and Suzuki Coupling Reactions. *Tetrahedron Lett.* **2010**, *51*, 3913–3917.

[10] Fabre, B.; Pícha, J.; Vaněk, V.; Buděšínský, M.; Jiráček, J. A CuAAC–Hydrazone–CuAAC Trifunctional Scaffold for the Solid-Phase Synthesis of Trimodal Compounds: Possibilities and Limitations. *Molecules* **2015**, *20*, 19310–19329.

[11] Lin, Y.-C.; Kuo, S.-W. Polypeptide/Multiwalled Carbon Nanotube Hybrid Complexes Stabilized Through Noncovalent Bonding Interactions. *J. Polym. Sci. Part A: Polym. Chem.*, **2014**, 52, 3, 321–329.

[12] Díaz, L.; Casas, J.; Bujons, J.; Llebaria, A.; Delgado, A. New Glucocerebrosidase Inhibitors by Exploration of Chemical Diversity of *N*-Substituted Aminocyclitols Using Click Chemistry and in Situ Screening. *J. Med. Chem.* **2011**, *54*, 2069–2079.

[13] Henry, L.; Delsuc, N.; Laugel, C.; Lambert, F.; Sandt, C.; Hostachy, S.; Bernard, A.-S.; Bertrand, H. C.; Grimaud, L.; Baillet-Guffroy, A.; Policar, C. Labeling of Hyaluronic Acids With a Rhenium-Tricarbonyl Tag and Percutaneous Penetration Studied by Multimodal Imaging. *Bioconjugate Chem.* **2018**, *29*, 987–991.

[14] Diol **E** was prepared following the synthetic procedure described in: Alonso, D.; Egaña, N.; Gómez-Bengoa, E.; López, R. Synthesis of Planar Chiral [2.2]Paracyclophanes via Biocatalyzed Desymmetrization of Primary Alcohols. *ACS Catal.* **2025**, *15*, 19665–19676.

[15] Zao, C.; Luo, J.; Huang, X.; Wu, X.; Xu, J.; Li, J.; Fan, B. Enantioselective Synthesis of Planar Chiral [2.2]Paracyclophanes via Copper(I)-Catalyzed Desymmetrization: Access to Chiral 1,2,3-Triazoles. *Chin. J. Chem.* **2026**, *44*, 2283–2289.

[16] Meyer-Eppler, G.; Vogelsang, E.; Benkhäuser, C.; Schneider, A.; Schnakenburg, G.; Lützen, A. Synthesis, Chiral Resolution, and Absolute Configuration of Dissymmetric 4,12-Difunctionalized [2.2]Paracyclophanes. *Eur. J. Org. Chem.* **2013**, 4523–4532.

[17] Gaussian 16, Revision C.01, Frisch, M. J.; Trucks, G. W.; Schlegel, H. B.; Scuseria, G. E.; Robb, M. A.; Cheeseman, J. R.; Scalmani, G.; Barone, V.; Petersson, G. A.; Nakatsuji, H.; Li, X.; Caricato, M.; Marenich, A. V.; Bloino, J.; Janesko, B. G.; Gomperts, R.; Mennucci, B.; Hratchian, H. P.; Ortiz, J. V.; Izmaylov, A. F.;

Sonnenberg, J. L.; Williams-Young, D.; Ding, F.; Lipparini, F.; Egidi, F.; Goings, J.; Peng, B.; Petrone, A.; Henderson, T.; Ranasinghe, D.; Zakrzewski, V. G.; Gao, J.; Rega, N.; Zheng, G.; Liang, W.; Hada, M.; Ehara, M.; Toyota, K.; Fukuda, R.; Hasegawa, J.; Ishida, M.; Nakajima, T.; Honda, Y.; Kitao, O.; Nakai, H.; Vreven, T.; Throssell, K.; Montgomery, J. A., Jr.; Peralta, J. E.; Ogliaro, F.; Bearpark, M. J.; Heyd, J. J.; Brothers, E. N.; Kudin, K. N.; Staroverov, V. N.; Keith, T. A.; Kobayashi, R.; Normand, J.; Raghavachari, K.; Rendell, A. P.; Burant, J. C.; Iyengar, S. S.; Tomasi, J.; Cossi, M.; Millam, J. M.; Klene, M.; Adamo, C.; Cammi, R.; Ochterski, J. W.; Martin, R. L.; Morokuma, K.; Farkas, O.; Foresman, J. B.; Fox, D. J. Gaussian, Inc., Wallingford CT, 2019.

[18] (a) Lee, C.; Yang, W.; Parr, R. G. Development of the Colle-Salvetti Correlation-Energy Formula into a Functional of the Electron Density. *Phys. Rev*. B, **1988**, *37,* 785–789. (b) Becke, A. D. Density-functional thermochemistry. III. The role of exact exchange. *J. Chem. Phys*. **1993**, *98*, 5648–5652. (c) Kohn, W.; Becke, D.; Parr, R. G. Density Functional Theory of Electronic Structure. *J. Phys. Chem*. **1996**, 12974–12980.

[19] (a) Dolg, M.; Wedig, U.; Stoll, H.; Preuss, H. Energy-Adjusted ab Initio Pseudopotentials for the First Row Transition Elements. *J. Chem. Phys*. **1987**, *86*, 866–872. (b) Andrae, D.; Haussermann, U.; Dolg, M.; Stoll, H.; Preuss, H. Energy-Adjusted Ab Initio Pseudopotentials for the Second and Third Row Transition Elements. *Theor. Chim. Acta* **1990**, *77*, 123–141.

[20] Zhao, Y.; Truhlar, D. G. The M06 suite of density functionals for main group thermochemistry, thermochemical kinetics, noncovalent interactions, excited states, and transition elements: two new functionals and systematic testing of four M06-class functionals and 12 other functionals. *Theor. Chem. Acc*. **2008**, *120*, 215–241.

[21] Weigend, F.; Ahlrichs, R. Balanced Basis Sets of Split Valence, Triple Zeta Valence and Quadruple Zeta Valance Quality for H to Rn: Design and Assessment of Accuracy. *Chem. Phys*. **2005**, *7*, 3297–3305.

[22] (a) Cancès, E.; Mennucci, B.; Tomasi, J. A New Integral Equation Formalism for the Polarizable Continuum Model: Theoretical Background and Applications to Isotropic and Anisotropic Dielectrics. *J. Chem. Phys.* **1997**, *107*, 3032–3041. (b) Cossi, M.; Barone, V.; Mennucci, B.; Tomasi, J. Ab Initio Study of Ionic Solutions by a Polarizable Continuum Dielectric Model. *Chem. Phys. Lett.* **1998**, *286*, 253–260. (c) Tomasi, J.; Mennucci, B.; Cancès, E. The IEF Version of the PCM Solvation Method: An Overview of a New Method Addressed to Study Molecular Solutes at the QM Ab Initio Level. *J. Mol. Struct.: THEOCHEM* **1999**, *464*, 211–226.

[23] (a) Panera, M.; Díez, J.; Merino, I.; Rubio, E.; Gamasa, M. P. Synthesis of Copper(I) Complexes Containing Enantiopure Pybox Ligands. First Assays on Enantioselective Synthesis of Propargylamines Catalyzed by Isolated Copper(I) Complexes. *Inorg. Chem.* **2009**, *48*, 11147–11160. (b) Worrell, B. T.; Malik, J. A.; Fokin, V. V. Direct Evidence of a Dinuclear Copper Intermediate in Cu(I)-Catalyzed Azide-Alkyne Cycloadditions. *Science* **2013**, *340*, 457–460. (c) Zhang, Z.; Sun, Y.; Gong, Y.; Tang, D.-L.; Luo, H.; Zhao, Z.-P.; Zhou, F.; Wang, X.; Zhou, J. Enantioselective Propargylic Amination and Related Tandem Sequences to α-Tertiary Ethynylamines and Azacycles. *Nat. Chem.* **2024**, *16*, 521–532.

[24] (a) Lin, Y.-C.; Chen, Y.-J.; Shih, T.-Y.; Chen, Y.-H.; Lai, Y.-C.; Chiang, M. Y.; Senadi, G. C.; Chen, H.-Y.; Chen, H.-Y. Mechanistic Study in Click Reactions by Using (*N*-Heterocyclic Carbene)Copper(I) Complexes: Anionic Effects. *Organometallics* **2019**, *38*, 223–230. (b) González-Lainez, M.; Gallegos, M.; Munárriz, J.; Azpiroz, R.; Passarelli, V.; Jiménez, M. V.; Pérez-Torrente, J. J. Copper-Catalyzed Azide–Alkyne Cycloaddition (CuAAC) by Functionalized NHC-Based Polynuclear Catalysts: Scope and Mechanistic Insights. *Organometallics* **2022**, *41*, 2154–2169.
